# Carbon‐Centered Reactivity in Carbodiphosphorane‐Based Ligands Allowing for Redox‐Non‐Innocent Ligand/Ligand Dual Bond‐Activation

**DOI:** 10.1002/anie.202419786

**Published:** 2025-01-21

**Authors:** Philipp Schatz, Weiqin Xu, Sebastian Rynek, Leon Maser, Niels Heise, Olaf Fuhr, Dieter Fenske, Haleh Hashemi Haeri, Dariush Hinderberger, Matthias Vogt, Robert Langer

**Affiliations:** ^1^ Institute of Chemistry, Faculty of Natural Science II Martin Luther University Halle-Wittenberg Kurt-Mothes-Str. 2 06120 Halle(Saale) Germany; ^2^ Chemistry Department Guangdong University of Education Guangzhou 510303 PR China; ^3^ Institut für Nanotechnologie (INT) and Karlsruhe Nano Micro Facility (KNMFi) Karlsruher Institut für Technologie (KIT) Kaiserstraße 12 76131 Karlsruhe Germany; ^4^ Lehn Institute of Functional Materials (LIFM), School of Chemistry Sun Yat-Sen University Guangzhou 510000 PR China

**Keywords:** carbodiphosphoranes, homogeneous catalysis, redox noninnocence, ligand reactivity, metal-ligand-cooperation

## Abstract

A pronounced nucleophilicity in combination with a distinct redox non‐innocence is a unique feature of a coordinated ligand, which in the current case, leads to unprecedented carbon‐centered reactivity patterns: A carbodiphosphorane‐based (CDP) pincer‐type rhodium complex allows to cleave two C−Cl‐bonds of geminal dichlorides via two consecutive S

## Graphical abstract

**Carbon does the Job** Carbodiphosphoranes are often described as ligand‐stabilized carbon(0) compounds (L_2_C), which in our contribution are demonstrated to be highly nucleophilic and non‐innocent in the coordination sphere of rhodium. These properties allow for unprecedented types of bond activation and new pathways in catalysis.

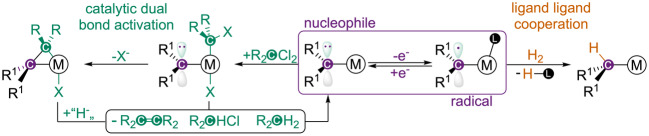

## Introduction

**Figure 1:**
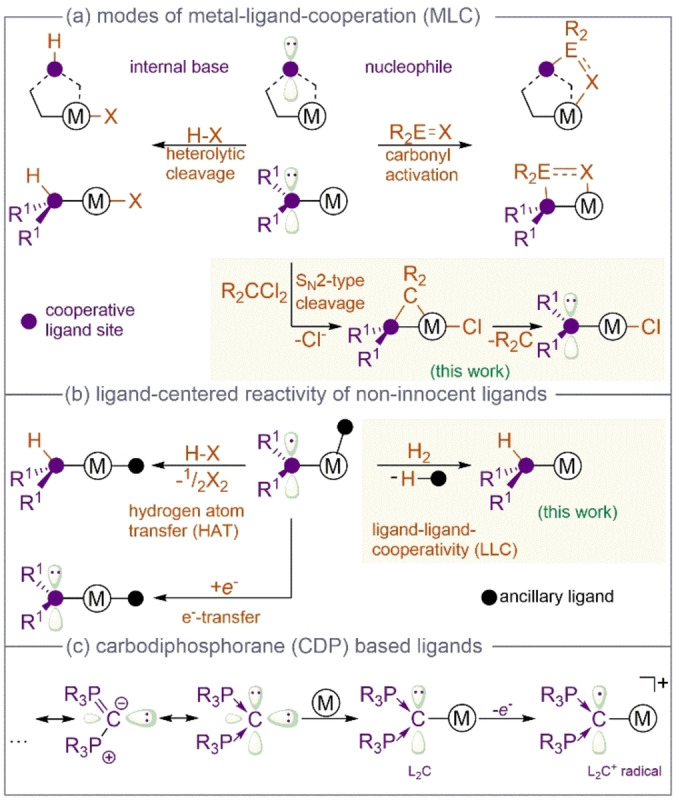
(a) Known and novel modes of MLC. (b) Typical reactivity of non‐innocent ligands vs. the reported LLC in this manuscript. (c) Selected resonance structures of CDPs, their metal coordination and subsequent oxidation.

The progress in the design of functional ligands, capable to assist elementary steps in catalytic‐ or bond activation reactions, has led to a significant development of novel and more sustainable reactions that address urgent problems in chemistry with implication to global societal issues.^[1]^ This metal‐ligand‐cooperative behaviour is most commonly observed for ligands, acting as internal base (Figure 1a) that assists the heterolytic cleavage of an element‐hydrogen bond without the need for a change in the metal oxidation state and act as hydrogen bond donor in its protonated form.[^1^, ^2^] In more rare cases, these cooperative sites can act as nucleophiles and activate unsaturated functional groups (SO_2_, CO_2_, aldehydes, nitriles, isonitriles etc.), which in a few cases can be used to convert the activated, ligand‐bound substrate and close a catalytic cycle.^[3]^ Examples of cooperative sites, being a ligating group or a remote functional group, are documented for numerous transition metal complexes, as well for metallo‐enzymes, such as hydrogenases.[^3^, ^4^, ^5^, ^6^, ^7^, ^8^] Although the basicity, the nucleophilicity and the redox potential of coordinated ligands are interconnected properties, a preferential reactivity pattern for a given ligand set is often observed. In this context, the ability of ligands or donor groups to adopt different redox states in the coordination sphere of transition metals can facilitate alternative reaction types (Figure 1b) and is a well‐established concept in enzymes such as galactose oxidase.[^9^, ^10^, ^11^, ^12^] Besides typical redox‐active ligand motifs, such as NO, O_2_, 1,2‐dithiolenes, 1,2‐dioxolens, 1,2‐diketones, 1,2‐diimines, or iminopyridines,[^6^, ^7^, ^8^, ^13^, ^14^, ^15^, ^16^, ^17^] also ligands, which are commonly used as spectator ligands in organometallic chemistry and homogenous catalysis, became subject of the discussion regarding their redox non‐innocent properties.^[18]^ In many cases the reactivity of these ligand centred radicals is limited to hydrogen atom transfer, redox steps, or an internal electron transfer to a reactant in the metal coordination sphere. Notably, most examples for metal‐ligand‐cooperative and redox non‐innocent ligands are based on hetero atoms. Although carbon‐based ligands, such as carbenes, are versatile ligand motifs in homogeneous catalysis, coordination‐ and organometallic chemistry, which can also be used for the stabilization of unusual and elusive compounds in main group chemistry,[^19^, ^20^, ^21^, ^22^, ^23^, ^24^] their electronic structure is different and they usually do not act as internal base or coordinated nucleophile, however their redox non‐innocence has been repeatedly discussed.[^25^, ^26^, ^27^, ^28^, ^29^]

Carbon‐based ligands which are more related to the general cooperative and redox non‐innocent ligand types depicted in Figure 1a are carbones, which are characterized by two lone pairs at the central carbon atom and two stabilizing donors, such as phosphanes in carbodiphosphoranes (CDPs) or two carbene substituents in case of carbodicarbenes (CDCs).[^30^, ^31^, ^32^, ^33^, ^34^, ^35^, ^36^, ^37^] They are often described as carbon(0) compounds[^38^, ^39^, ^40^, ^41^] and act as neutral ligands with σ/π‐donor ability and extremely strong electron donating properties (Figure 1c).[^32^, ^42^, ^43^, ^44^, ^45^, ^46^, ^47^, ^48^, ^49^, ^50^, ^51^] These binding properties lead for example to highly reactive and catalytically active CDP main group element compounds.[^52^, ^53^]

In analogy to the pioneering work by Grützmacher and co‐workers on aminyl radical ligands^[54]^ and the recently demonstrated redox non‐innocence of CDCs in the coordination sphere of highly oxidized metal centres,[^55^, ^56^] we herein report an exclusive and preferential ligand‐based reactivity of carbon‐centred radical in a CDP‐moiety that allows for the cleavage of non‐polar reactants by cooperation of the CDP‐radical with an ancillary ligand. Moreover, the facile ligand oxidation is in line with strong nucleophilic character of the coordinated (non‐oxidized) CDP carbon atom. We report in the following the combination of a metal‐based bond activation followed by a ligand‐based, second bond activation step. Such a dual bond activation is without precedence in the literature and is demonstrated to yield different olefination and hydrodehalogenation products under catalytic conditions.

## Results and Discussion

**Scheme 1:**
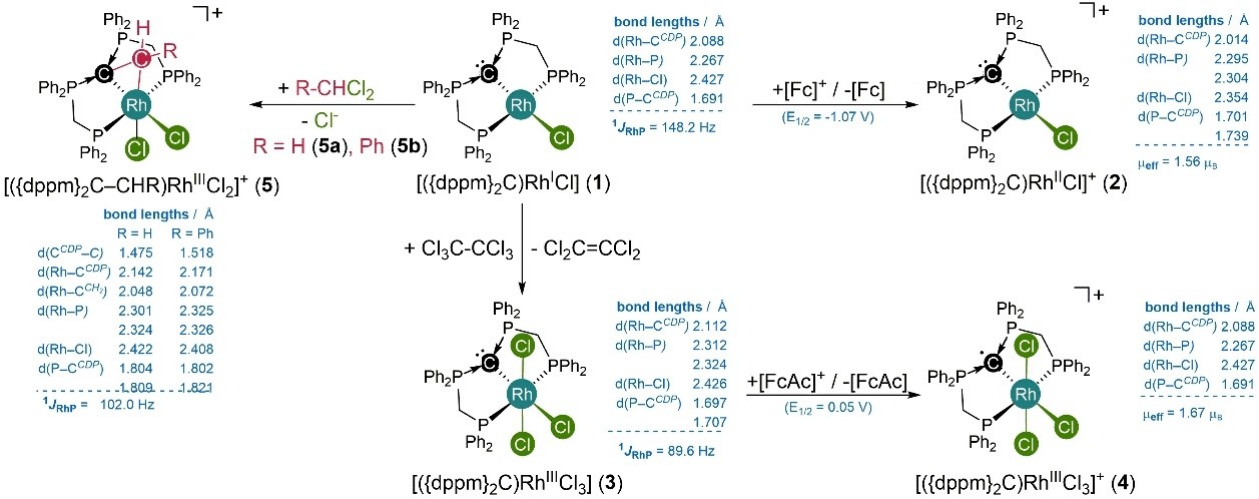
Synthesis of carbodiphosphorane‐based rhodium pincer‐type complexes and their reactivity, with structural and spectroscopic key‐features of the fully characterized complexes **1**–**5**.

**Synthesis and Redox Chemistry** Inspired by the seminal work on rhodium complexes bearing tridentate ligand with a central aminyl radical moiety,[^57^, ^58^, ^59^, ^60^] we synthesized the rhodium complex **1** with a CDP‐based pincer‐type ligand, using a strategy previously established for other transition metals.[^61^, ^62^, ^63^, ^64^] The rhodium(I) complex [({dppm}_2_C)RhCl] (**1**) is straightforwardly oxidized by ferrocenium salts in THF to give the cationic complex [({dppm}_2_C)RhCl]^+^ (**2**, Scheme 1), as evident from a quasi‐reversible one‐electron oxidation wave at low potential (−1.07 V vs. ferrocene/ferrocenium Fc/Fc^+^ redox couple, Figure S21) in the cyclic voltammogram. A two‐electron oxidation of **1** can be achieved by utilization of hexachloroethane, which selectively yields the diamagnetic trichlorido rhodium(III) complex [({dppm}_2_C)RhCl_3_] (**3**) and tetrachloroethylene. To our surprise the cyclic voltammogram of **3** shows a reversible oxidation wave at rather low potentials (+0.05 V vs. Fc/Fc^+^, Figure S31) and the utilization of a suitable oxidant such as an acetyl ferrocenium salt indeed results in a clean one‐electron oxidation to give complex [({dppm}_2_C)RhCl_3_]^+^ (**4**).

**Figure 2:**
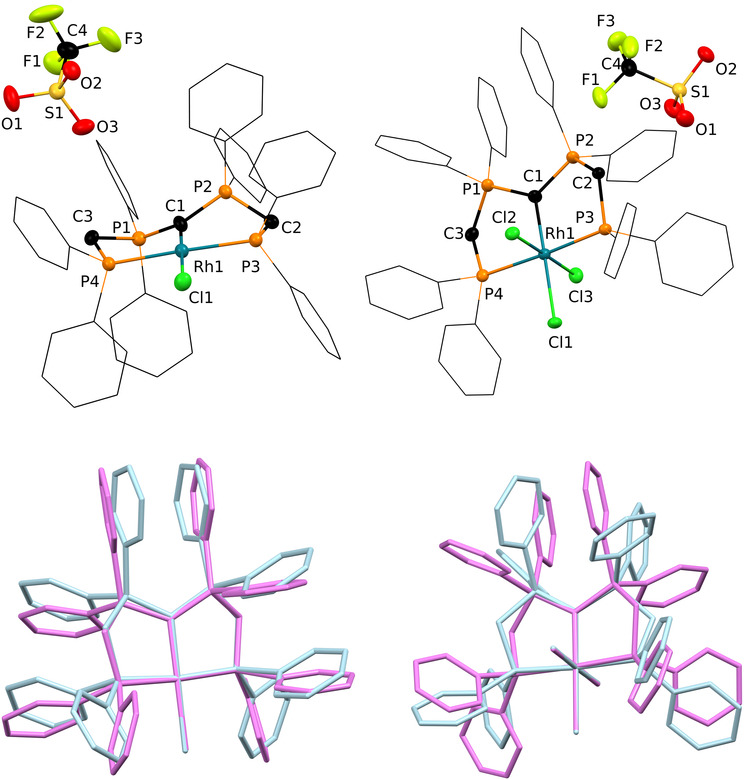
Top: Molecular structures of **2** (left) and **4** (right) in the solid state (thermal ellipsoids are drawn with 50 % probability; hydrogen atoms are omitted for clarity and phenyl‐rings are display as wire frame). Bottom left: Overlayed molecular scaffolds of **1** (violet) and **2** (blue). Bottom right: **3** (violet) and **4** (blue). Counter ions and hydrogen atoms are omitted for clarity.

The molecular structures of complexes **1**‐**5** were characterized by single crystal X‐ray diffraction analysis (scXRD), which confirmed that the principal connectivity of all atoms within the respective molecular scaffolds is retained upon one‐electron oxidation of **1** and **2** to give **3** and **4**, respectively (Figure 2). In both cases the oxidation results in shortening of the Rh–C and the Rh–Cl‐bond as well as an elongation of the two P–C^*CDP*^‐bonds, indicating a reduced charge delocalization of the CDP‐backbone after oxidation. Notably, the CDP carbon atom maintains in a strict trigonal planar environment in all complexes (**1**‐**4,** see overlayed structures in Figure 2, bottom).

The terminal PPh_2_‐groups in **1** give rise to doublet of triplets resonance in the ^31^P{^1^H} NMR spectrum with a ^1^ *J*_RhP_ coupling constant of 148.4 Hz, which represents a typical value for rhodium(I) complexes, whereas complex **3** gives rise to a smaller coupling constant of 89.6 Hz, reflecting the smaller s‐orbital contribution in rhodium(III) complexes.^[65]^ The oxidized complexes **2** and **4** did not show any resonance in the ^31^P{^1^H} NMR spectra and display broad, paramagnetically shifted resonances in the ^1^H NMR spectrum. Determination of the magnetic moments using Evans’ NMR spectroscopic method in solution of THF‐*d*_8_ (**3**) and DCM‐*d*_2_ (**4**) gave magnetic moments of 1.54 μ_b_ for **2** and 1.67 μ_b_ for **4**, which is in agreement with one unpaired electron and is close to the value, expected for the spin only approximation.

**Figure 3:**
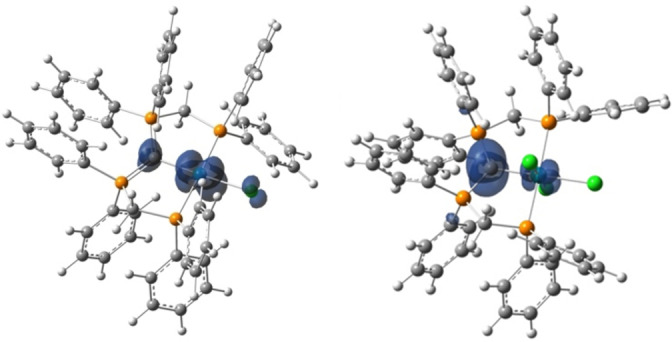
Calculated spin density plot of complexes **2** (left) and **4** (right, S=1/2, the isosurface value is 0.005, ωB97X‐D/def2‐TZVPP).

**EPR Spectroscopy and Quantum Chemical Investigations** Further insights into the structural identity of complexes **2** and **4**, as well as the nature of the radicals in these compounds, are provided by quantum chemical investigations, using density functional theory (DFT) on ωB97X‐D / def2‐TZVPP level of theory. The structural changes observed upon oxidation of **1** and **3** are well reproduced in the optimized geometries derived from the calculations. Inspection of the highest occupied molecular orbitals (HOMOs) in **1** and **3** reveals that they are best described as an antibonding π‐type interaction of the p‐orbitals at the CDP carbon atom and the chlorido ligand in *trans*‐position with a d‐orbital of the central rhodium atom. The single occupied molecular orbital (SOMO) after oxidation essentially exhibits a similar shape as the respective HOMOs in **1** and **3**, however significant dissimilarities can be noticed for **2** versus **4** with respect to the contribution of different atomic orbitals. In case of complex **2** a spin density of ρ=0.09 is calculated at the CDP carbon atom, whereas the spin density at the central rhodium atom is calculated to be much larger at ρ=0.81, which allows for an overall assignment of a rhodium(II) metallo‐radical complex. In sharp contrast, the ligating carbon atom of the CDP ligand in complex **4** hosts the major contribution of the spin density with ρ=0.62, whereas the spin density at the central rhodium atom was calculated to be only ρ=0.18 and 0.01 for the adjacent chlorido ligand. From the calculated spin density values and the corresponding spin density plot (Figure 3 and Table S13) it becomes apparent that the unpaired electron is mainly localized at the ligating C−atom with a minor charge delocalization to the attached phosphine substituents (Σρ=0.14). These findings are in line with the description of complex **4** as a rhodium(III) complex with a ligand‐centered carbon based radical.

**Figure 4:**
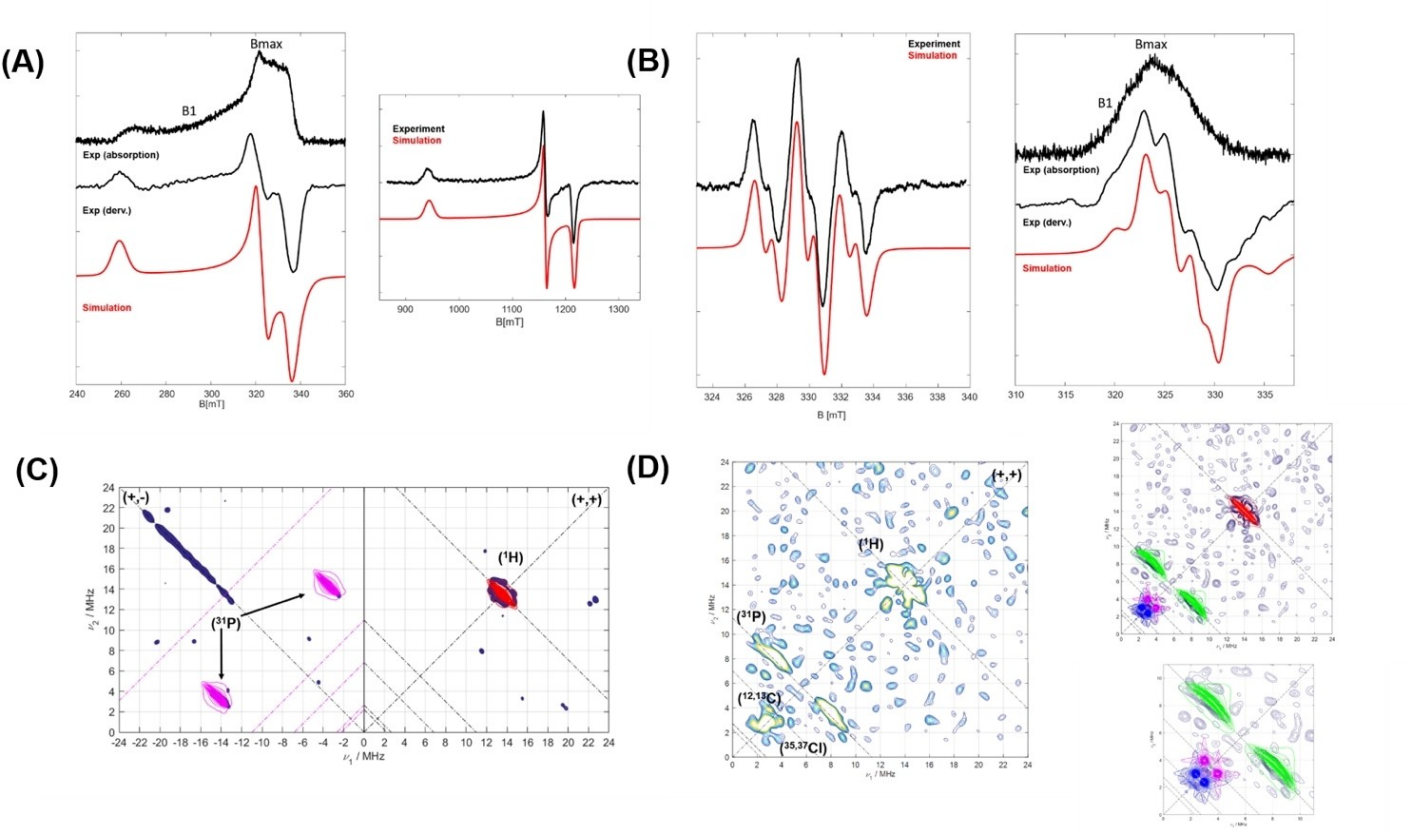
(**A**) Low temperature continuous wave (CW) EPR spectra of complex **2** at different frequencies (left: X‐band (9.4 GHz) at 20 K / right: Q‐band (33.9 GHz) at 50 K). Corresponding field positions for ESEEM and HYSCORE experiments are shown as B_max_ and B_1_. (**B**) X‐band CW EPR spectrum of **4** at ambient temperature (left) and Echo detected spectrum at 50 K (right). (**C**) HYSCORE spectrum of **2** at B_max_ at 20 K (experimental data is shown in marine blue, simulations in red for ^1^H and magenta for ^31^P nuclei). **(D)** HYSCORE spectrum of **4** measured at 30 K at B_1_, shown on the left and corresponding simulation of spectrum on the right (upper panel) with a magnified representation for low frequency nuclei (lower panel). Simulations for ^1^H, ^31^P, ^13^C, ^35,37^Cl nuclei are shown in red, green, magenta and blue, respectively.

EPR spectroscopic measurements were performed to gain further experimental insights into the nature of the radical complexes **2** and **4**. The X‐band absorption spectrum of complex **2** shows a broad spectral feature (~120 mT), which is indicative of localized spin density on the central rhodium atom (Figure 4A left). EPR measurements at higher frequency (34 GHz, Q‐band, Figure 4A right) resulted in a highly resolved spectrum with a clear rhombicity.

The CW‐EPR X‐band spectrum of complex **4** at room temperature, displays a clear splitting (Figure 4B left) couplings from two ^31^P nuclei (A(^31^P)=74 and 75 MHz) as well as coupling to the central rhodium atom (A(^103^Rh)=27 MHz), which is in line with the calculated spin delocalization (Figure 3). The low temperature X‐band EPR measurement of the electron spin echo (ESE) of **4** (30 K, Figure 4B right), resulted in a spectrum of ~20 mT width, significantly narrower compared to that of complex **2**. The narrow signal in the EPR spectra of **4** in combination with the small ^103^Rh hyperfine coupling constant (27 MHz), strongly suggesting the presence of an organic, partially delocalized radical in **4**.

Electron Spin Echo Envelope Modulation (ESEEM) measurements of **2** and **4** were performed on two different field positions B_max_ and B_1_ (high field and intermediate, corresponding to g_x_ and g_y_, planar contributions, see Figure 4) to detect couplings of the unpaired electron with different types of nuclei, which was then further analyzed by 2D Hyperfine Sub‐level Correlation (HYSCORE) experiments.

The highly resolved HYSCORE spectrum of **2**, obtained on the B_max_ position, shows the presence of both weakly and strongly coupled nuclei (Figure 4C): At quadrant (+,+),indicating weak couplings, matrix protons are observed, for which simulations showed the presence of two inequivalent protons with hyperfine couplings of A=[1,−3,1] and [−3,1,1] MHz. The observed strongly coupled signals at the (+,−) quadrant around the ^31^P Larmor frequency could be well simulated with a rather isotropic hyperfine coupling of A=[16,18,21] MHz. The ridge along the diagonal line of (+,−) quadrant is a result of imperfect phase cycling. The HYSCORE on B1 position represented only the matrix protons (Figure S11). The HYSCORE experiment of **4** at B_max_ confirmed the presence of matrix protons, phosphorous, and chlorine nuclei in the weak coupling quadrant (Figure S13). In addition, weakly coupled phosphorous nuclei with considerably reduced A(^31^P) of [1,8,1] MHz are observed. Also, the presence of a chlorine nucleus with small A(^35,37^Cl)=[0.1,1.0,−0.10] MHz and quadrupolar coupling of Q(^35,37^Cl)=[4,−1,−1] MHz is well reproduced by simulations. Moving toward B_1_ position (in‐plane position), the measured HYSCORE (Figure 4D) showed a signal arising from ^13^C nucleus, in addition to corresponding signals from ^1^H, ^31^P and ^35,37^Cl. The observation of signals arising from ^13^C nuclei correlates with the predominant organic nature of the radical in **4** and implies a significant spin density on the ligating CDP−carbon nucleus, considering the low natural abundance of ^13^C (1.07 %).

**Scheme 2:**
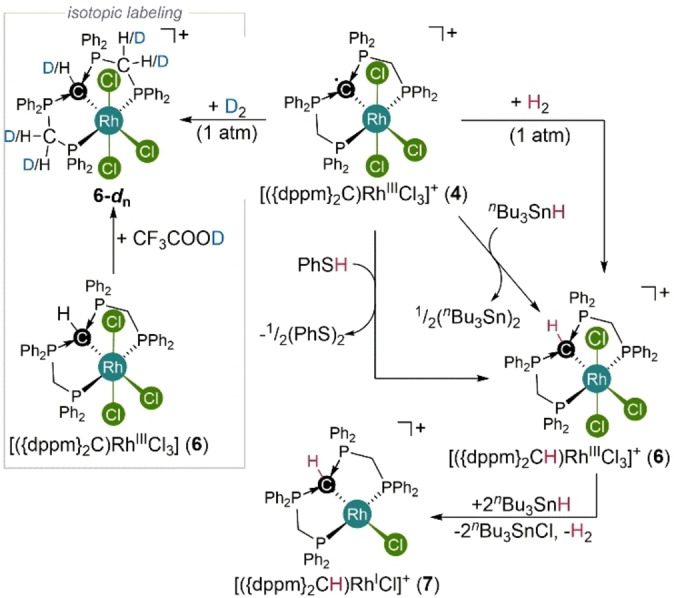
Right: ligand‐ and subsequent metal‐based reactivity of rhodium(III) complex **4** with an oxidized carbodiphosphorane‐based pincer‐type ligand. Left: Isotopic labeling experiments.

**Reactivity Study** Based on the spectroscopic and quantum chemical investigations, we examined whether the concluded ligand‐based radical character is reflected in the reactivity of complex **4** (Scheme 2). The reaction of complex **4** with thiophenol (PhSH) results in the selective hydrogen atom transfer (HAT) and formation of the rhodium(III) complex **6** and can be closely followed by ^31^P{^1^H} NMR spectroscopy (Figure S67). The accordingly formed disulfide PhS−SPh was clearly detected by high resolution mass spectrometry (HR‐MS) (Figure S68). Notably, the closed‐shell rhodium(III) complex **3** does not react with PhSH, clearly underlining the ligand based reactivity. Hence, with respect to their redox non‐innocence and the resulting reactivity, the CDP ligand motif may be regarded as neutral counterpart of the carbanion‐based pincer‐type complexes, reported by Iluc.[^66^, ^67^] Concerning the HAT using PhSH, similar observations are made in course of the reaction of **4** and ^*n*^Bu_3_SnH, which initially leads to the formation of complex **6** and ^*n*^Bu_3_Sn−Sn^*n*^Bu_3_ as inferred from ^31^P{^1^H} and ^119^Sn NMR spectroscopy (Figure S70 and S71). Unexpectedly, complex **6** slowly reacts with excess ^*n*^Bu_3_SnH to give the closed‐shell rhodium(I) complex **7**. The formation of **7** is best explained by an initial HAT to give complex **6**, which subsequently undergoes chlorido/hydrido ligand exchange, followed by a final reductive elimination of dihydrogen to give complex **7**. As complex **3** does not react with ^*n*^Bu_3_SnH, an initial reduction of **4** can be ruled out as pathway for the formation of **6** and **7**. Overall, the observed reactivity patterns clearly indicate that even in the presence of comparably reactive ancillary ligands like chlorido ligands, the CDP‐based reactivity dominates and is observed prior to a reaction in the coordination sphere of the metal center. Notably, complex **4** reacts selectively with H_2_ to complex **6**, which is, to the best of our knowledge, an unprecedented reactivity for a non‐innocent steering ligand. In this context it is worth noting that certain organic radicals as well as uncoordinated carbenes are able to activate H_2_.[^68^, ^69^]

**Scheme 3:**
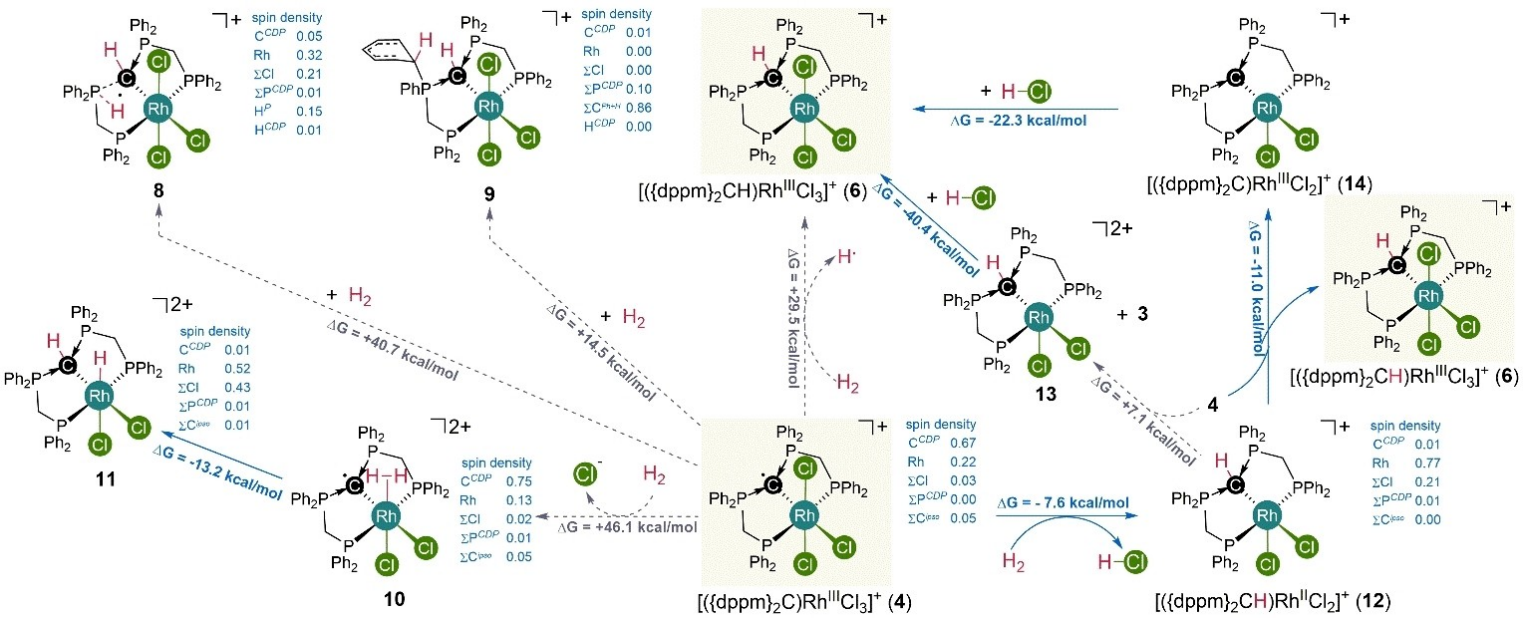
Relative stability of potential intermediates for the activation of H_2_ by complex **4** using density functional theory (DFT) on B97D3/def2‐TZVP/SMD(CH_2_Cl_2_) level of theory.

**Mechanistic Study ‐ L/L Cooperation** To gain further insight into this unusual reactivity, different pathways for the activation of H_2_ by **4** were considered in quantum chemical investigations using density functional theory (B97D3/def2‐TZVP/SMD{CH_2_Cl_2_}). The overall H_2_‐activation (2 x **4** + H_2_ → 2 x **6**) is thermodynamically favorable by ΔG=−40.9 kcal/mol, but the initial generation of one equivalent **6** and a solvated hydrogen atom is thermodynamically uphill (ΔG=+29.5 kcal/mol) and does not seem to be a viable pathway (Scheme 3). Similar results were calculated for the initial cleavage of H_2_ across the P→ C‐bond (**8**, ΔG=+40.7 kcal/mol) as well as for the H_2_‐cleavage and hydrogen atom transfer (HAT) to one of the phosphorous‐bound phenyl rings (ΔG=+14.5 kcal/mol), which was found to be a viable H_2_‐activation pathway for boron‐based radicals.^[70]^ The initial substitution of one chlorido ligand in **4** by a dihydrogen ligand to the dicationic complex **10** is unfavorable as well (ΔG=+46.1 kcal/mol), although the following activation step is favorable by ΔG=−13.2 kcal/mol (**10** → **11**).

Based on these findings, isotopic labeling experiments were performed to illuminate the accessibility of the identified most favorable, calculated pathways (Scheme 2). Notably, the reaction of complex **4** with D_2_ instead of H_2_ results in the selective formation **6‐*d***_**n**_ with partial deuterium incorporation at the protonated CDP‐bound hydrogen atom as well as at the CH_2_‐groups of the pincer arms, but no deuterium incorporation was observed by ^2^H NMR spectroscopy for phenyl‐groups. Resonances corresponding to hydrido‐ or deuterido ligands were absent in the corresponding ^1^H and ^2^H NMR spectra. Based on these observations, the involvement of species **9** can be ruled out. Furthermore, the deuterated solvent CD_2_Cl_2_ used in some of these experiments did not lead to any deuterium incorporation in control experiments.

As the NMR spectra of complex **6** did not indicate chemical exchange (e. g. line broadening) and no exchange correlations could be observed in ^1^H EXSY NMR spectra, we investigated the deuterium incorporation in **6** in the presence of different acids. Whereas no deuteration of **6** is observed in the presence of slight excess CD_3_OD and CD_3_COOD, the reaction with CF_3_COOD leads to partial deuteration of the CDP‐bound hydrogen atom as well as the P‐bound CH_2_‐groups. On the other hand the reaction of [({dppm}_2_C)RhCl_3_] (**3**) with CD_3_OD, CD_3_COOD or [Et_2_O ⋅ D](BArF) resulted in deuteration to **6‐*d***_**n**_ with the same deuterium incorporation pattern (Figure S81). Overall, these findings corroborate a possible formation of an acid such as HCl during the H_2_‐/D_2_‐activiation by **4**, which would be the case for the formation of **12** as an intermediate (ΔG=−7.6 kcal/mol). Continuing the activation sequence from **12**, the observed H/D‐exchange could either be explained by exchange of the product complex **6** with the formed acid or by protonation/deuteration of intermediates such as [({dppm}_2_C)RhCl_3_] (**3**) or [({dppm}_2_C)RhCl_2_]^+^ (**14**). In summary, both pathways involving either the formation of **3** and **13** (via e^−^ transfer) or the formation of **6** and **14** (via HAT) from **12** and **4**. The initial activation step is identical in both pathways and involves the cooperation of a non‐innocent CDP ligand with an ancillary chlorido ligand, leading to the cleavage of the homolytic H−H‐bond. A transition state was calculated for the H_2_ cleavage by the CDP‐radical ligand (ΔG=−35.8 kcal/mol). Remarkably, the formation of rhodium hydridic intermediates were not observed. This principle mode of ligand‐ligand‐cooperation is essentially not known and provides a powerful approach for bond activation chemistry, especially when considering that a suitable support for the redox‐active centers, may not necessarily be a transition metal center.

**Scheme 4:**
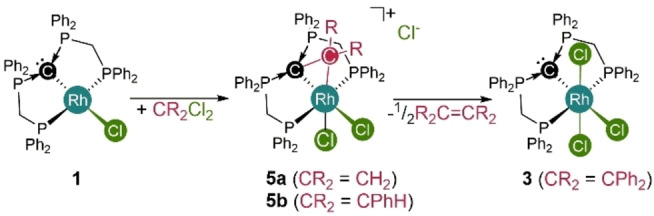
Reactivity of complex **1** towards geminal dichlorides.

**Figure 5:**
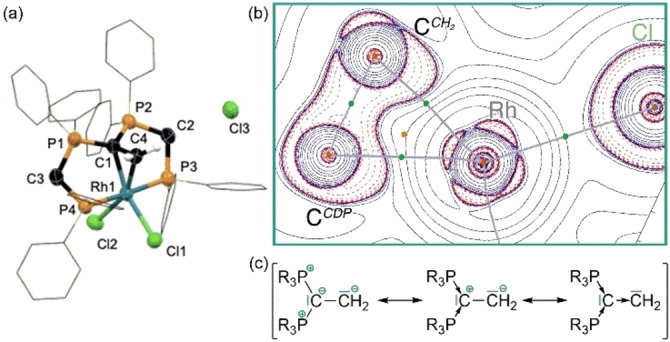
(a) Molecular structure of **5 a** in the solid state hydrogen atoms are omitted for clarity (except C−CH_2_−Rh), phenyl‐rings are shown as wire frame, the remaining atoms are drawn with thermal ellipsoids at 50 % probability. (b) Calculated contour line diagram of the Laplacian distribution of the electron density in **5 a** (dashed red lines indicate areas of charge concentration, solid blue lines show areas of charge depletion; bond paths are displayed in gray with bond critical points in green). (c) Possible resonance structures in line with C−C‐single bond, indicating the double donor ability.

**Investigation of a Dual Reactivity Mode** Based on the unexpected low potential for the one electron oxidation of complexes **1** and **3**, we hypothesized that classic elementary steps of organometallic chemistry involving formal two‐electron redox steps may occur with the participation of the coordinated CDP entity. In line with the oxidation of complex **1** by Cl_3_C−CCl_3_ (Scheme 1) an immediate reaction is observed with methylene chloride resulting in the selective formation of the cationic complex **5 a** (Scheme 4), which was fully characterized and its structural identity was confirmed by scXRD (Figure 5). The central rhodium atom in **5 a** is coordinated by the initial pincer‐type ligand, two chlorido ligands and one CH_2_‐fragment, which is additionally stabilized by the coordinated CDP carbon atom, giving rise to an overall distorted octahedral coordination sphere around a formal Rh^III^ center. The C^*CDP*^–C^*carbene*^‐bond length in **5 a** is with d_C1−C4_=1.475 Å close to the value for a C−C‐single bond. The tentative assignment as a rhodium(III) complex is further supported by the ^1^ *J*_RhP_ coupling constant of 102 Hz in the ^31^P{^1^H} NMR spectrum.

A similar ligand entity was recently reported in a platinum complex by Frenking and Petz,^[71]^ where the bonding situation was described as a CDP‐stabilized carbene that acts as four‐electron σ‐donor entity. A closer look into the bonding situation in **5 a** using quantum theory of atoms in molecules (QTAIM) provides a similar picture: the Laplacian distribution of the electron density (∇ ^2^ρ, Figure 5) displays two areas of charge concentration (∇ ^2^ρ > 0) for the donation from each carbon atom to the central rhodium atom along two bond paths. Hence, the bonding situation in **5 a** is best described by the resonance structures in Figure 5c.

The corresponding reaction with benzal chloride PhCHCl_2_ instead of CH_2_Cl_2_ results in the formation of the analogous dual C−Cl‐bond activation product **5 b** as the minor product together with [({dppm}_2_C)RhCl_3_] (**3**) as major product (**5 a**:**3**=1:2). The formation of complex **3** can formally be explained by elimination of the stabilized carbene fragment in **5 b**. As both the Rh−C (2.072 Å) and the C^*CDP*^–C^carbene^‐bond (1.518 Å) in **5 b** are longer with respect to **5 a**, the assumption of a weakly bound carbene fragment in **5 b** might be valid (scXRD details in Figure S6). The reaction of complex **1** with Ph_2_CCl_2_ selectively results in the formation complex **3** and no stabilized carbene complex of type **5** could be detected. Based on ^1^H NMR and ^13^C{^1^H} NMR spectra, as well as GC traces and HR‐MS spectra tetraphenylethylene Ph_2_C=CPh_2_ was identified as a further product of the reaction, which suggests that a Ph_2_C carbene fragment is released from **5** and subsequent dimerization gives rise to the corresponding olefin.

Turning again to quantum chemical investigations using density functional theory (DFT) on B97D3/def2‐TZVP/SMD(CH_2_Cl_2_) level of theory, the difference in Gibbs energy for the formation of CDP‐stabilized carbene complexes of type **5** vs. the formation of complex **3** and the corresponding carbene / olefin were calculated. In case of CH_2_Cl_2_, olefin formation is only slightly more favored by ΔΔG=−5.6 kcal/mol with respect to **5 a**, but the initial release of the carbene is strongly disfavored (ΔG=+59.5 kcal/mol). For benzal chloride, PhCHCl_2_, formation of **3** and (formally) half equivalent of *trans*‐stilbene is more favorable (ΔΔG=−12.7 kcal/mol) with respect to **5 b**, but the release of the corresponding carbene is again thermodynamically uphill by ΔG=+40.7 kcal/mol with respect to the starting materials **1** and PhCHCl_2_. With Ph_2_CCl_2_ as reactant the overall olefin formation is significantly more favorable with respect to the corresponding carbene complex **5 c** (ΔΔG=−34.1 kcal/mol) and the carbene release from **5 c** is less uphill in Gibbs energy with respect to **1** and Ph_2_CCl_2_ (ΔG=+20.6 kcal/mol). Overall, with an increasing number of phenyl rings bound to the central carbon atom of the geminal dichloride the stability of the corresponding carbene complexes of type **5** is reduced and olefin formation as well as carbene release become more favorable.

As the formation of **5** or **3** from **1** suggests that the central CDP‐carbon atom in **1** is actively involved in at least one of the bond activation steps, intermediates, as well as connecting transition states, were located on the same level of theory. The initial oxidative addition of geminal dichlorides by rhodium pincer‐type complexes has previously been reported.[^72^, ^73^] In line with these findings a transition state (**TS_1/15 a_**, ΔG=+11.6 kcal/mol) could be located, leading to the octahedral rhodium(III) intermediate **15 a** (ΔG=−16.7 kcal/mol) that contains a new CH_2_Cl‐ligand adjacent to the CDP‐carbon atom as well as an additional chlorido ligand with respect to **1**. This first C−Cl‐bond activation step is best described as an S_N_2‐type oxidative addition step with the rhodium center as nucleophile. The intramolecular, nucleophilic attack of the coordinated CDP‐carbon atom to the carbon atom of the CH_2_Cl ligand via **TS_15 a/5 a_** (ΔG=−6.8 kcal/mol with respect to **1** + CH_2_Cl_2_) results in nucleophilic substitution of the chloride substituent by the CDP‐entity and formation of **5 a** (ΔG=−34.4 kcal/mol). The second C−Cl‐bond activation by the CDP‐ligand has an even smaller effective barrier with respect to **15 a** of +9.9 kcal/mol than the initial metal‐based bond activation. The observed combination of a classical metal‐ and (nucleophilic) ligand‐based reactivity is unprecedented and allows for the consecutive cleavage of two different bonds in a substrate, which bears high potential for entirely new directions in homogeneous catalysis. In this regard, we took advantage of the identified reactivity and investigated a catalytic approach for the dehalogenation of geminal dichlorides.

**Catalytic Conversion of Geminal Halides** As complexes such as **6** seem to reductively eliminate H_2_ upon exchange of two chlorido by two hydrido ligands (Scheme 2, **6** → **7**), we reasoned that hydride transfer reagents may close a catalytic cycle and allow for a regeneration of an active species after the dual C–Cl‐bond activation and the release of the resulting fragments. The conversion of geminal dichlorides was observed with a number of reagents, such as different silanes and hydrido borates, where LiBH_4_ lead to the highest conversions (Table 1). In case of Ph_2_CCl_2_ full conversion is observed with two equivalents of LiBH_4_ in the presence of 2.5 mol % **1** at 50 °C in THF after 24 h (Entry 1), resulting in the selective formation of tetraphenyl ethylene with 94 % yield (product type **I**). In the absence of catalyst **1** 58 % conversion of Ph_2_CCl_2_ was detected under otherwise identical conditions, but the mono‐hydrodehalogenation product of type **III** is selectively formed in 57 % yield (Entry 2). In case of benzal chloride (PhCHCl_2_) as substrate no reaction is observed without catalyst, whereas the reaction proceeds with full conversion in the presence of **1**, but no olefin formation is observed in this case (Entry 3). Instead, a C–C‐coupling product (**II**) is detected with 35 % yield, indicating a radical pathway, as well as 36 % mono‐ (**III**) and 24 % di‐hydrodehalogenation product (**IV**). The formation of **II**, **III** and **IV** as products instead of **I**, is in line with an unfavorable carbene dissociation after the dual C–Cl‐bond activation of PhCHCl_2_. In the absence of **1** no conversion is observed (Entry 4). The alkyl‐substituted substrate 2,2‐dichlorobutane is fully converted under the reaction conditions (Entry 5), yielding 2 % of the C–C‐coupling product **II** and 95 % of the mono‐hydro‐dehalogenation product **III**, but no olefin **I** and no **IV**, whereas no conversion of the starting material is detected in the absence of **1** (Entry 6). The reaction with 2,2‐dichloropropane yielded 2‐chloropropane (61 %, Entry 7). Resonances associated with propane were not observed in the NMR spectra, however its formation cannot be ruled out. CH_2_Cl_2_ as substrate showed full conversion and a significant amount of product formation could be observed, but we were unable to precisely quantify the volatile products (type **III** chloromethane and **IV** methane). We performed an isotopic labeling experiment using LiBD_4_ as reducing agent in a flame‐sealed NMR tube to confirm the origin of the hydrogen atoms in the formed products and detected CH_2_DCl as well as CH_2_D_2_ by means of ^1^H and ^2^H NMR spectroscopy (Entry 8). Again, no conversion is observed in the absence of catalyst **1** (Entry 9). As LiBD_4_ as well as some of the geminal dichlorides are rather inexpensive chemicals, the developed protocol may serve as an attractive methodology for the partial deuterium labeling of chemicals.

**Table 1:** Catalytic dehalogenation with complex **1** as catalyst.^[a]^

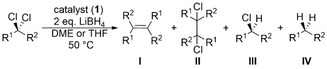								
Entry	t	Cat.	R^1^ / R^2^	Conv^.[b]^	Product(s) (Yield)^[b]^			
	[h]	[mol %]		[ %]	**I**	**II**	**III**	**IV**
1	24	2.4	Ph / Ph	>99	94	0	0	0
2	24	0	Ph / Ph	58	0	0	57	0
3	24	2.5	Ph / H	>99	0	35	36	24
4	24	0	Ph / H	0	0	0	0	0
5	92	2.5	Me / Et	>99	0	2	95	0
6	24	0	Me / Et	0	0	0	0	0
7	24	2.5	Me / Me	81	0	0	61	0^[d]^
8	24	3.0	H / H	>99	CH_2_DCl+CH_2_D_2_^[c]^			
9	24	0	H / H	0	0			

^[a]^Reaction conditions: 2.5 mol% **1**, 1 equiv. substrate and 2 equiv. LiBH_4_ or LiBD_4_ in pyridine/THF‐*d*_8_. ^[b]^Determined by ^1^H and ^13^C{^1^H} NMR spectroscopy. ^[c]^Additionally determined by ^2^H NMR spectroscopy. ^[d]^ Propane was not observed, yet its formation cannot be ruled out

**Scheme 5:**
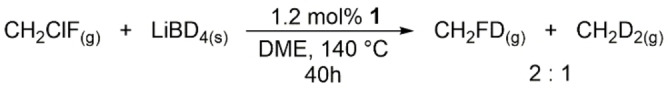
Catalytic conversion of the CFC CH_2_ClF.

Remarkably, even CH_2_ClF reacts in a catalytic reaction at 140 °C (Scheme 5), leading to 85 % conversion of LiBD_4_ after 40 hours, which was used (instead of LiBH_4_) as reductant in a sealed NMR tube to identify and quantify the possible reaction products. By ^2^H and ^19^F NMR spectroscopy CH_2_D_2_ and CH_2_DF were identified as reaction products in the liquid phase. An ^19^F NMR spectrum in water of the precipitate formed during the reaction revealed the formation of LiF. By approximation of the tube volume, the pressure after the reaction and the Henry coefficient for methane we estimated that CH_2_D_2_ is formed with a turnover number of approx. 70 and CH_2_DF with a turnover number of 158 after 40 h. The observation of CH_2_D_2_ and CH_2_DF as reaction products suggest that the initial C–Cl‐bond cleavage is metal‐centered, followed by a potential ligand‐centered C–F‐cleavage, which would underline the high nucleophilicity of coordinated CDPs.

**Figure 6:**
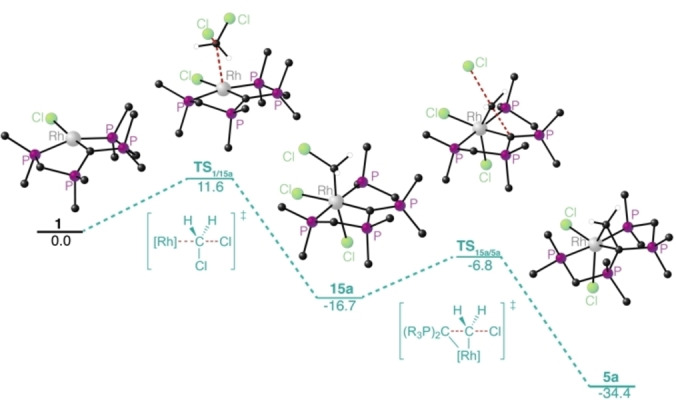
Calculated pathway for the activation of geminal dichlorides by complex **1** (B97D3/def2‐TZVPP/SMD{THF}, phenyl‐rings and non‐relevant hydrogen atoms are omitted for clarity). Δ*G* values are given in kcal/mol.

**Scheme 6:**
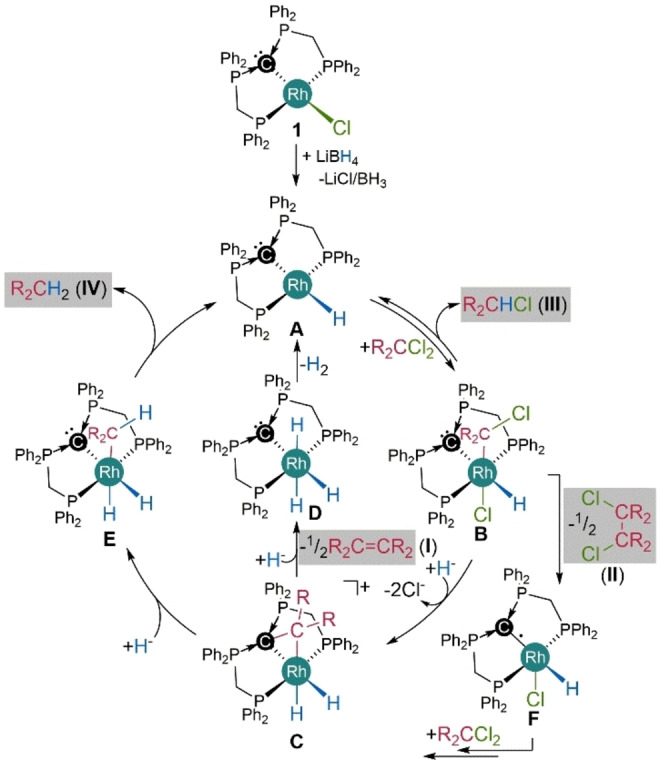
Plausible pathways for the catalytic conversion of geminal dichlorides by pre‐catalyst **1**. H^−^ refers to hydrido moieties delivered by LiBH_4_.

Based on the pathway depicted in Figure 6 we assume that in the presence of hydride‐transfer‐reagents, the chlorido ligands of the active species and intermediates experience partial or full exchange by hydrido ligands (full exchange is exemplary depicted in Figure 6). A plausible mechanism for the observed catalytic reactions could therefore involve the active species **A** that reacts in a S_N_2‐type metal‐centered oxidative addition step to **B** (Scheme 6). A second C–Cl‐bond is cleaved by attack of the CDP‐ligand in **B**, leading to complex **C** involving a stabilized carbene (CH_2_). Release of the carbene and its subsequent dimerization to the corresponding olefin (**I**) leads to **D**, which could reductively eliminate H_2_ and regenerate the active species **A**. An alternative pathway involves cleavage of the C–C‐bond in **C** and the generation of an alkyl ligand in **E**, which can reductively eliminate **IV** and regenerate the active species **A**. The formation of the mono‐dehalogenation product I**II** can be explained by reductive elimination from **B** to give **A** and I**II**. The product containing a newly formed C–C‐bond (**II)**, is likely formed in a radical pathway that involves homolytic cleavage of a Rh–C‐bond in **B** to a chloromethyl radical and **F**. According to the proposed mechanism, complex **3** should be an active pre‐catalyst, as well. Similar conversions are observed with **3** as pre‐catalyst for 2,2‐dichlorbutane and Ph_2_CCl_2_ supporting the proposed mechanism.. However, the product distribution was different in case of Ph_2_CCl_2_.

## Conclusion

In conclusion, we report a range of unique ligand‐centered reactivity patterns for rhodium complexes with a pincer‐type ligand encompassing a single carbon as ligating atom in a carbodiphosphorane (CDP) giving rise to two important features:

Firstly, our investigations show that pincer‐ligand platforms based on CDPs are weak internal bases when coordinated to a metal, but exhibit strong nucleophilicity and, secondly, they are easily oxidized at low potentials in the reported rhodium complexes.

Strong nucleophilicity and redox‐noninnocence render the CDP‐pincer a remarkable actor ligand that can facilitate the cleavage of dihydrogen involving a carbon‐centered radical in concert with an ancillary ligand. This is an unprecedented example of ligand‐ligand‐cooperation (LLC) and bears important relevance for a metal‐free activation of non‐polar bonds, such as H–H‐, C–H‐, and C–C. Consequently, the pronounced nucleophilicity of the coordinated CDP‐moiety was utilized for a novel type of dual bond activation that involves the consecutive cleavage of two C–Cl‐bonds This finding bears special importance as the reactivity significantly differs from classical MLC schemes. Eventually, we could take advantage of this unique dual reactivity pattern for catalytic olefination ‐ and hydrodehalogenation reactions.

## Supporting Information

The authors have cited additional references within the Supporting Information (SI ^[74–106]^)

## Conflict of Interests

The authors declare no conflict of interest.

## Supporting information

As a service to our authors and readers, this journal provides supporting information supplied by the authors. Such materials are peer reviewed and may be re‐organized for online delivery, but are not copy‐edited or typeset. Technical support issues arising from supporting information (other than missing files) should be addressed to the authors.

Supporting Information
