# Supplementary material for: Carbon‐Centered Reactivity in Carbodiphosphorane‐Based Ligands Allowing for Redox‐Non‐Innocent Ligand/Ligand Dual Bond‐Activation
Source: Angew Chem Int Ed Engl. 2025 Jan 21;64(7):e202419786. doi: 10.1002/anie.202419786 (PMC11811687; doi:10.1002/anie.202419786)
Supplement: Supplementary file 1 — Supporting Information [file ANIE-64-e202419786-s001.pdf]

Angewandte Chemie
Eine Zeitschrift der Gesellschaft Deutscher Chemiker

Supporting Information

Carbon-Centered Reactivity in Carbodiphosphorane-Based Ligands Allowing for Redox-Non-Innocent Ligand/Ligand Dual Bond-Activation

P. Schatz, W. Xu, S. Rynek, L. Maser, N. Heise, O. Fuhr, D. Fenske, H. H. Haeri, D. Hinderberger, M. Vogt, R. Langer**

Supporting Information for:

Carbon-Centered Reactivity in Carbodiphosphorane-Based Ligands Allowing for Redox-Non-Innocent Ligand/Ligand Dual Bond-Activation

Index

1. **Materials and Methods** .... - 3 -
2. **NMR spectroscopy** .... - 3 -
3. **IR Spectroscopy** .... - 4 -
4. **CHN/S Analysis** .... - 4 -
5. **High Resolution Mass Spectrometry** .... - 4 -
6. **Cyclic Voltammetry** .... - 5 -
7. **Determination of the magnetic moment using Evans' method** .... - 5 -
8. **Synthesis and Characterization of 1-7** .... - 6 -
9. **Crystallographic Details** .... - 17 -
10. **EPR Spectroscopy** .... - 31 -
11. **Computational Details** .... - 37 -
12. **Spectra and Voltammograms for 1-7** .... - 39 -
13. **Reactivity Study of Complex 4** .... - 66 -
14. **Experiments addressing H/D exchange reactions:** .... - 79 -
15. **Experiments addressing catalytic activity towards geminal dichlorides:** .... - 82 -
16. **References** .... - 97 -

1. Materials and Methods

All experiments were carried out under an atmosphere of purified argon 5.0 using standard Schlenk techniques or in a *MBraun LABmaster* / *GS MEGA* glove box.

Diethylether, tetrahydrofuran (THF) and toluene were dried over Na. *n*-Hexane was dried over $LiAlH_4$. Dichloromethane (DCM) and acetonitrile were dried over CaH_2 prior distillation under argon protective atmosphere. The solvents were stored under argon and over appropriate molecular sieves. Deuterated solvents and pyridine were degassed with freeze-pump-thaw cycles and stored over appropriate molecular sieves under argon atmosphere.

Ferrocen, thiophenol, tributhyltinhydride, AgOTf, [Rh(acac)(ethylene)$_2$] and [Rh(cod)Cl_2], KO^tBu, hexachloroethane, HCl x Et_2O were purchased from commercial sources (*ABCR* and *Sigma-Aldrich*). The ligand precursor bis(diphenylphosphino)methane (dppm) was synthesized following the procedure published by K. Sommer.[1] [HC(dppm)$_2$]Cl was synthesized according to the literature by Peringer and co-workers.[2] Sodium tetrakis[3,5-trifluoromethyl)phenyl]borate (NaBArF)],[3] ferroceniumhexafluorophosphate,[4] and acetylferrocene[5] / acetylferroceniumtriflate[6] was synthesized according to the literature procedures, using AgOTf instead of $AgBF_4$.

2. NMR spectroscopy

^{1}H, ^{13}C, ^{11}B, ^{19}F and ^{31}P NMR spectra were recorded using Agilent Technologies 400 MHz VNMRS and 500 MHz DD2 NMR spectrometers at 300 K. ^{1}H and ^{13}C {^{1}H}, ^{13}C-APT (attached proton test) NMR chemical shifts are reported in ppm with respect to tetramethylsilane. The resonance of the residual protons of the deuterated solvent was used as internal reference for ^{1}H NMR spectra. The solvent peak of the deuterated solvent was used as internal reference for ^{13}C NMR spectra. ^{11}B NMR chemical shifts are reported in ppm referenced to $BF_3(OEt_2)$ as external standard. ^{19}F NMR chemical shifts are reported in ppm with respect to $CFCl_3$ as external standard. ^{31}P NMR chemical shifts are reported in ppm with respect to H_3PO_4 and referenced to an external 85 % solution of phosphoric acid in D_2O. ^{119}Sn NMR shifts are reported in ppm with respect to Me_4Sn. The following abbreviations are used for the description of NMR data: br (broad), s (singlet), d (doublet), t (triplet), q (quartet), quin (quintet), m (multiplet).

3. IR Spectroscopy

FT-IR spectra were recorded by attenuated total reflection (ATR) of the solid samples on a Bruker Tensor 27 spectrometer at ambient temperature. The intensity of the absorption band is indicated as w (weak), m (medium), s (strong), vs (very strong) and br (broad).

4. CHN/S Analysis

Elemental microanalyses were carried out by combustion analysis in an UNICUBE CHN(S) analyzer (ELEMENTAR) equipped with an optional kit for fluorine-containing samples using argon as carrier/shielding gas and tin-foil crucibles for sample preparation (or silver-foil crucibles if fluorine was present in the sample), as well as in an ELEMENTAR-VARIO EL using helium as carrier/shielding gas and tin crucibles for sample preparation.

5. High Resolution Mass Spectrometry

High-resolution mass spectra were recorded on a *Thermo Scientific Q Exactive Plus* spectrometer equipped with an *Orbitrap Mass Analyzer* and two different ion sources: (a) LIFDI (liquid injection field desorption ionization) by *Linden CMS*, Germany and (b) *Thermo Scientific HESI-II* (heated electro spray ionization). External mass calibration was performed in HESI mode by measuring *calmix solution* provided from *Thermo Scientific*. *LTQ ESI Positiv* Ion Calibration Solution and *ESI Negativ* Ion Calibration Solution, respectively.

LIFDI specifications: The emitter heating current (EHC) was ramped during the spectral acquisition up to 80 mA maximum at a constant heating rate of 20 $mAmin^{-1}$. Moderate glowing furnished bake-off of residual sample amounts prior sample reloading. Emitter high voltage and EHC were controlled using the Linden LIFDI-700 electronics and LIFDI-700 control software. Standard emitters were obtained from Linden CMS (activated 13 µm tungsten wire). Ion acquisition was accomplished in positive LIFDI mode with counter electrode potential at -10 kV and in negative LIFDI mode at + 6 kV. The sample solutions were stored in a 1.5 mL glass vial equipped with a PTFE coated rubber septum (*Macherey-Nagel*, screw cap N9) prior their delivery to the emitter via a fused silica capillary (0.75 µm diameter). Sample deposition on the emitter was optically monitored via camera image. Air sensitive samples were prepared in a glove box under argon atmosphere and transferred in the glass vial under a protective argon atmosphere for LIFDI measurement.

6. Cyclic Voltammetry

Cyclic voltammograms were recorded on a µSTAT 400 (*Deutsche METROHM GmbH*) potentiostat equipped with a *TSC 1600-closed* electro-chemical cell (*RHG Instruments*) with platinum working- and counter electrode using a micro pseudo reference Ag electrode. The cell was charged with the analyte solution (2 mM, 0.7 mL) in a glovebox under argon atmosphere. Electrolyte: tetrabutylammonium hexafluorophosphate (0.1 M). Internal reference: ferrocene/ferrocenium redox couple by addition of crystalline ferrocene.

7. Determination of the magnetic moment using Evans' method

The ^{1}H NMR-spectra were recorded using an Agilent Technologies 500 MHz DD2 NMR spectrometer at 300 K. For each experiment, 4 mg of the paramagnetic complexes were dissolved in 1 mL DCM-d_2 or THF-d_8 and ca. 300 µL were placed in a coaxial NMR tube. Another 5 mm NMR tube was filled with 600 µL of the accordingly deuterated solvent. The coaxial tube was carefully inserted in the 5 mm NMR tube. The Evans experimental NMR-spectra were acquired as a standard ^{1}H-NMR-spectrum. A solvent correction is due to the diluted system not necessary.

The calculation of the experimental magnetic moment can be obtained by the following equation:[7,8]

$$\mu_{exp} = 2.828\sqrt{\frac{3 \cdot T}{4\pi \cdot F} \cdot \frac{\Delta_f}{c}}$$

Where T is the temperature, F the spectrometer frequency in Hz, Δ_f the shift in Hz and c the concentration in mol·mL^{-1}.

8. Synthesis and Characterization of **1-7**

Synthesis of [({dppm}$_2$C)RhCl] (1)

95.6 mg (100.0 µmol) [({dppm}$_2$CH)RhCl]Cl (**7**) and 11.2 mg (100.0 µmol) KO^{t}Bu were suspended in 6 mL toluene and stirred for 16 hours at ambient temperature. Afterwards, the solvent was removed *in vacuo*. The brown residue was resolved in 3 mL THF and was filtered through a syringe filter (PTFE 0.5 µm porosity). The reddish-brown solution was then added dropwise in 15 mL *n*-hexane to precipitate a brown solid, which was decanted from the mother liquor and subsequently washed three times with 4 mL *n*-hexane. The product was further dried *in vacuo* for 24 hours to receive [({dppm}$_2$C)RhCl] (**1**) Yield: 85.0 mg (92.5 µmol, 92.5%). Single crystals, suitable for a scXRD analysis, were obtained layering a THF solution with *n*-hexane.

^{1}H NMR (499.71 MHz, dimethylsulfoxide-d_6) δ 7.74 (br q, J = 5.6 Hz, 8H), 7.41 (br dt, J = 14.5, 7.1 Hz, 12H), 7.32 br (q, J = 9.0, 7.2 Hz, 12H), 7.11 (br t, J = 7.4 Hz, 8H), 4.00 (br s, 4H) ppm.

^{13}C{^{1}H} NMR (125.67 MHz, dimethylsulfoxide-d_6) δ 134.14-133.98 (m, *partially obscured* , 4C, $C_{ar-ipso}$), 133.88 (t, J = 7.0 Hz, 8C, CH_{ar}), 132.27 (t, J = 4.8 Hz, 8C, CH_{ar}), 131.13 (br s, 4C, CH_{ar}), 130.03 (br s, 4C, CH_{ar}), 129.32-129.97(m, *partially superimposed*, 4C, $C_{ar-ipso}$, 128.15 (t, J = 4.3 Hz, 8C, CH_{ar}), 127.97 (t, J = 5.2 Hz, 8C, CH_{ar}), 42.19 (m, 2C, PCH$_2$P), 21.33 (weak m in 1D spectrum, identified via 2D ^{1}H^{13}C HMBC correlation cross peak, 1C, (Ph$_2$P)$_2$$C$) ppm.

^{31}P{^{1}H} NMR (202.30 MHz, dimethylsulfoxide-d_6) δ 29.87 (dt, $^1J_{PRh}$ = 148.4, J_{PP} = 50.1 Hz), 27.48 (t, J_{PP} = 49.5 Hz) ppm.

IR ATR (cm^{-1}): 3647 m, 3334 m, 3050 m, 2923 m, 2853 m, 2325 m, 1951 m, 1622 m, 1586 m, 1571 m, 1482 m, 1434 s, 1360 m, 1308 m, 1278 m, 1135 m, 1100 s, 1068 m, 1041 m, 1026 m, 998 m, 959 m, 846 m, 806 m, 781 m, 769 m, 736 s, 689 s, 617 m, 553 m, 524 s, 502 s, 471 s, 435 m, 415 s, 372 m, 342 m, 300 m, 262 m, 231 m, 218 m

Element.Anal.calcd for $C_{51}H_{44}ClP_4Rh$: C 66.64%, H 4.83%; found: C 65.96%, H 5.05 %

HRMS calcd. m/z for M$^+$ [$C_{51}H_{44}ClP_4Rh$]$^+$: 918.11316; found: 918.1120; Δ= 1.21 ppm.

Synthesis of [({dppm}$_2$C)RhCl]OTf (2-OTf)

20.0 mg (21.8 µmol) of [({dppm}$_2$C)RhCl] (**1**) were dissolved in 1.5 mL THF. A slowly dropwise addition of 8 mg (21.2 µmol, 0.97 equiv.) acetylferroceniumtriflate in 1.0 mL THF leads to a reddish pink solution. The solution was then dropped in 15 mL *n*-hexane to precipitate a red solid, which was decanted from the mother liquor and subsequently washed four times with 4 mL *n*-hexane. Continuous drying for 24 hours at high vacuum led to 11.2 mg (10.5 µmol, 48.2%) of a reddish pink powder of [({dppm}$_2$C)RhCl]OTf (**2-OTf**). Single crystals, suitable for a scXRD analysis, were obtained layering a THF solution with a few drops of *n*-hexane.

Effective magnetic moment (Evan´s method): 1.54 μ_b

IR ATR (cm^{-1}): 3055 m, 2959 m, 2916 m, 2357 m, 2325 m, 1616 m, 1587 m, 1573 m, 1482 m, 1435 m, 1364 m, 1257 s, 1222 m, 1183 m, 1158 m, 1099 s, 1068 m, 1027 s, 998 m, 923 m, 845 m, 801 m, 770 s, 752 m, 736 s, 686 s, 635 s, 616 m, 571 m, 518 s, 498 s, 475 s, 428 m, 418 m, 397 m, 367 s, 329 m, 302 s, 266 m, 257 m, 227 m, 210 s, 204 s

Element.Anal. calcd for [$C_{51}H_{44}ClP_4Rh$][(O_3SCF_3)]: C 58.47 %, H 4.15 %, S 3.00 %; found: C 57.78% H 4.30%, S 3.01%.

HRMS calcd. m/z for M$^+$-Otf [$C_{51}H_{44}ClP_4Rh$]$^+$: 918.11316; found: 918.11456 ; Δ= 1.53 ppm

Synthesis of [({dppm}$_2$C)RhCl]PF$_6$ (2-PF$_6$)

20.0 mg (21.8 µmol) of [({dppm}$_2$C)RhCl] (**1**) were dissolved in 2 mL THF. A slowly dropwise addition of 7 mg (21.2 µmol, 0.97 equiv.) ferrocenium hexafluorophosphate in 1 mL THF leads to a red solution. The solution was then added dropwise in 15 mL *n*-hexane to precipitate a reddish-pink solid, which was decanted from the mother liquor and subsequently washed four times with *n*-hexane. After continuous drying for 24 hours at high vacuum the solid was dissolved in 2 mL THF and layered with a few drops of *n*-hexane. The mixture was stored at -40°C and red crystals formed overnight. The crystals were decanted from the mother liquor and washed two times with 2 mL of *n*-hexane. The product was further dried *in vacuo* for 24 hours to give 9.8 mg (9.2 µmol, 42.2%) of [({dppm}$_2$C)RhCl]PF$_6$ (**2-PF$_6$**).

Synthesis of [({dppm}$_2$C)RhCl$_3$] (3)

Method A: 150 mg (163.0 µmol) of [({dppm}$_2$C)RhCl] (**1**) was suspended in 6 mL THF under vigorous stirring. 46 mg (194 µmol, 1.2 equiv.) of hexachloroethane (C_2Cl_6) was added and the mixture was allowed to react at ambient temperature for 16 h. Subsequently, all volatiles were evaporated *in vacuo* and the residual solids were dissolved in 2 mL of DCM. The solution was filtered through a syringe filter (PTFE 0.5 µm porosity). A few drops of diethylether were added to the filtrate and the clear solution was allowed to crystallize at ambient temperature. After one week, a few crystals (1-2 mg) of [({dppm}$_2$C)RhCl$_3$] had formed along with an amorphous powder. The suspension was decanted from the crystals and the crystals were washed briefly with dichloromethane. The mother liquor and dichloromethane washing solution were combined to give a clear solution and the previously obtained crystals were used as seeds. The mixture was stored at -40°C and large dark brown crystals formed overnight. The crystals were decanted washed with diethylether. Note the product was obtained as a mixture of crystals with quite different colors: Larger crystals appear as dark brown, smaller as off-white. The product was grinded and dried at high vacuum to give 52 mg (0.053 mmol, 32 % yield) [({dppm}$_2$C)RhCl$_3$] (**3**) as a brownish powder.

^{1}H NMR (499.71 MHz, dichlormethane-*d*$_2$) δ 7.74 (m, 8H, CH_{ar}), 7.52 (br q, J = 7 Hz, 8H, CH_{ar}), 7.26 (br q, J = 7 Hz, 8H, CH_{ar}), 7.15 (br q, J = 7 Hz, 16H, CH_{ar}), 4.15 (br p, J = 4.5 Hz, 4H, PCH_2P) ppm.

^{13}C{^{1}H} NMR (125.67 MHz, dichlormethane-*d*$_2$) δ 134.04 (t, J = 5.2 Hz, 8C, CH$_{ar}$), 133.68 (t, J = 5.6 Hz, 8C, CH$_{ar}$), 131.89 (t, J = 1.4 Hz, , 4C, CH$_{ar}$), 131.79 (*pseudo* tt, $^1J_{CP}$ = 23.2, $^3J_{CP}$ = 1.9 Hz, , 4C, *partially superimposed,* $C_{ar\text{-}ipso}$), 129.82 (t, J = 1.1 Hz, , 4C, CH$_{ar}$), 128.83 (dtt, $^1J_{CP}$ = 77.3 Hz $^2J_{CRh}$ = 38.4 Hz, $^3J_{CP}$ = 2.0 Hz, 4C, *partially superimposed,* $C_{ar\text{-}ipso}$) 128.24 (t, J = 5.9 Hz, 8C, CH$_{ar}$), 127.65 (t, J = 5.1 Hz, , 8C, CH$_{ar}$), 38.39 – 36.40 (m, J_{CP}, J_{CRh}, 2C, PCH$_2$P), -21.91 (td, J = 81.3, 35.8 Hz, (dppm)$_2$$C$) ppm.

^{31}P{^{1}H} NMR (202.30 MHz, dichlormethane-*d*$_2$) δ 25.70 (t, J_{PP} = 36.3 Hz, 2P, (Ph$_2$$P$)$_2$C), 16.84 (dt, J_{PR} = 94.9, J_{PP} = 36.3 Hz, 2P, Rh-P) ppm.

IR ATR (cm^{-1}): 3629 m, 3457 m, 3051 m, 2987 m, 2917 m, 2854 m, 2683 m, 2325 m, 1981 m, 1901 m, 1821 m, 1616 m, 1587 m, 1573 m, 1506 m, 1484 m, 1434 s, 1356 m, 1315 m, 1265 m, 1190 m, 1159 m, 1141 m, 1096 s, 1067 m, 1052 m, 1027 m, 998 m, 933 m, 895 m, 840 m, 773 m, 737 s, 725 s, 690 s, 617 m, 523 s, 502 s, 486 s, 470 s, 438 s, 412 m, 372 m, 352 m, 321 s, 277 s, 267 s, 236 m, 213 s, 207 s

Element.Anal.calcd. for $C_{51}H_{44}Cl_3P_4Rh$: C 61.87%, H 4.48%; found: C 61.17% H 4.11%

HRMS calcd. m/z for M$^+$ [$C_{51}H_{44}Cl_3P_4Rh$]$^+$: 988.05086; found: 988.05121; Δ= 0.35 ppm.

Alternative method B: 25.0 mg (97 µmol) of [Rh(acac)(ethylene)$_2$] and 79.3 mg (97 µmol) of [(dppm)$_2$CH]Cl were dissolved in 2 mL 1,2-difluorobenzene and stirred for 5 minutes at ambient temperature. The solution was stored at -40°C for 24 hours to obtain orange microcrystals. These crystals were dried *in vacuo* for 12 hours. Afterwards 40.0 mg (42 µmol, 1.00 equiv.) of the orange crystalline powder and 20.0 mg (84 µmol, 2.0 equiv.) C_2Cl_6 were dissolved in 8 mL 1,2-difluorobenzene and allowed to stir for 24 hours at ambient temperature. All volatiles of the resulting dark brown solution were removed *in vacuo*. The brownish residue was washed three times with 2 mL diethylether and dried again *in vacuo*. The beige solid was dissolved in 5 mL dichloromethane, layered with 4 mL diethylether and stored at -30°C. After 1-2 days brown crystals were formed. The final product [({dppm}$_2$C)RhCl$_3$] (**3**) was obtained after continuous drying of the crystalline precipitate under high vacuum (25.6 mg, 25.9 µmol, 61.2%).

Synthesis of [({dppm}$_2$C)RhCl$_3$]OTf (4-OTf)

25.0 mg (25.3 µmol) of [({dppm}$_2$C)RhCl$_3$] (**3**) were dissolved in 1.5 mL dichloromethane. A slowly dropwise addition of 9 mg (23.9 µmol, 0.94 equiv.) acetylferroceniumtriflate in 1 mL dichloromethane leads to a dark brownish green solution. The solution was then dropped in 15 mL *n*-hexane to precipitate a dark green solid, which was decanted from the mother liquor and subsequently washed four times with 4 mL *n*-hexane. Continuous drying for 24 hours at high vacuum lead to a brownish green powder of [({dppm}$_2$C)RhCl$_3$]OTf. Single crystals, suitable for a scXRD study, were obtained via gas-phase diffusion of *n*-hexane into a saturated dichloromethane solution.

Effective magnetic moment (Evan´s method): 1.67 μ_b.

IR ATR (cm^{-1}): 3061 m, 2953 m, 2902 m, 2325 m, 1586 m, 1574 m, 1485 m, 1436 m, 1366 m, 1278 m, 1258 s, 1225 m, 1194 m, 1154 m, 1146 m, 1100 m, 1030 s, 999 m, 955 m, 852 m, 795 m, 778 m, 734 s, 689 s, 636 s, 573 m, 520 s, 499 s, 479 s, 443 m, 430 m, 414 m, 392 m, 380 m, 333 s, 302 m, 276 m, 237 m, 210 s

Element.Anal. calcd for [$C_{51}H_{44}Cl_3P_4Rh$][$B(C_8H_3F_3)_4$]: C 53.79%, H 3.05%; found: C 53.87% H 3.46%

HRMS calcd. m/z for M$^+$-OTf [$C_{51}H_{44}Cl_3P_4Rh$]$^+$: 988.05086; found: 988.05234 ; Δ= 1.50 ppm.

Synthesis of [({dppm}$_2$C)RhCl$_3$]BArF (4-BArF)

25.0 mg (25.3 µmol) of [({dppm}$_2$C)RhCl$_3$] (**3**) were dissolved in 1.5 mL dichloromethane. A slowly dropwise addition of 27 mg (24.5 µmol, 0.98 equiv.) acetylferrocenium BArF in 1 mL dichloromethane leads to a dark brownish green solution. The solution was then dropped in 15 mL *n*-hexane to precipitate a dark green solid, which was decanted from the mother liquor and subsequently washed four times with 4 mL *n*-hexane. Continuous drying for 24 hours at high vacuum lead to 37 mg (16.2, 79.0%) of a green microcrystalline powder of [({dppm}$_2$C)RhCl$_3$]BArF.

Synthesis of [({dppm}$_2$C-CH$_2$)RhCl$_2$]Cl (5a)

92.0 mg (100.1 μmol) [({dppm}$_2$C)RhCl] (**1**) were dissolved in 6 mL dichloromethane, resulting in a red solution. Single crystals could be obtained by layering the solution with *n*-hexane after two weeks. Yield: 30.2 mg (30.0 μmol, 30%).

^{1}H NMR (502.29 MHz, dimethylsufoxide-d_6, 300 K) δ 7.89 (q, J = 5.3 Hz, 4H, CH_{ar}), 7.83 – 7.76 (m, 8H, CH_{ar}), 7.71 (t, J = 7.5 Hz, 2H, CH_{ar}), 7.59 – 7.54 (m, 4H, CH_{ar}), 7.48 – 7.39 (m, 10H, CH_{ar}), 7.36 (t, J = 7.4 Hz, 4H, CH_{ar}), 7.28 (t, J = 7.6 Hz, 4H, CH_{ar}), 7.13 (t, J = 7.1 Hz, 4H, CH_{ar}), 4.67 (m, 4H, PCH_2P), 3.04 (t, J = 18.3 Hz, 2H, CCH_2Rh) ppm.

^{13}C{^{1}H} NMR (126.31 MHz, DMSO-d_6, 300 K) δ 135.40 – 135.01 (m, 4H, CH$_{ar}$), 134.60 (d, J = 15.9 Hz, 4H, CH$_{ar}$), 133.63 (t, J = 5.8 Hz, 4C, CH$_{ar}$), 133.38 – 133.07 (m, 10C, CH$_{ar}$), 131.52 (t, J = 23.3 Hz, 2C, CH$_{ar}$), 130.50 (d, J = 36.7 Hz, 4C, CH$_{ar}$), 129.41 (t, J = 5.8 Hz, 4C, CH$_{ar}$), 128.61 (t, J = 6.2 Hz, 4C, CH$_{ar}$), 128.14 (t, J = 4.7 Hz, 4C, $C_{ar\text{-}ipso}$), 127.33 (t, J = 4.9 Hz, 4C, CH$_{ar}$), 119.40 – 118.31 (m, 2C, RhP$C_{ar\text{-}ipso}$), 117.56 – 116.46 (m, 2C, Rh-P$C_{ar\text{-}ipso}$), 27.95 – 27.03 (m, 2C, PCH$_2$P), 22.73 – 22.26 (m, 1C, CCH$_2$Rh), 17.16 (d, J = 20.2 Hz, 1C, PCP) ppm.

^{31}P{^{1}H} NMR (203.34 MHz, dimethylsulfoxide-d_6, 300 K) δ 39.97 (td, J_{PP} = 21.8 Hz, $^3J_{PH}$ = 3.8 Hz), 19.73 (dt, $^1J_{PRh}$ = 102.1 Hz, J_{PP} = 21.8 Hz) ppm.

IR ATR (cm^{-1}): 3358 m, 3052 m, 2860 m, 1620 m, 1586 m, 1574 m, 1484 m, 1435 s, 1365 m, 1323 m, 1156 m, 1100 s, 1027 m, 997 m, 843 m, 776 m, 742 s, 729 s, 690 s, 613 m, 536 m, 520 s, 505 s, 473 s, 445 s, 433 s, 372 s, 347 s, 324 s, 306 m, 278 s, 236 s, 229 s, 204 s

Element.Anal. calcd for [$C_{52}H_{46}Cl_3P_4Rh$] ·3 DCM: C 55.25%, H 4.29%, found: C 55.47%, H 4.66%

HRMS calcd. m/z for M$^+$-HCl [$C_{52}H_{45}ClP_4Rh$]$^+$: 931.12098; found: 931.12194; Δ= 1.03 ppm.Note, complex **1** is also detected due cleavage of the {CH$_2$} fragment.

Synthesis of [({dppm}$_2$C-CHPh)RhCl$_2$]OTf (5b)

40mg (43.5 µmol) of [({dppm}$_2$C)RhCl] were suspended in 2 mL THF and 14 µL of benzalchloride (217.6 µmol, 5 equiv.) were added via a EPPENDORF pipette. After stirring for 1 hour a pink precipitate were formed. The reaction mixture was filtered, washed with small portions of THF until the filtrate is nearly colourless. Subsequently washing with *n*-hexane (2 times, 0.5 mL) and diethylether (2 times, 0.5 mL) and drying in high vacuum leads to 24 mg (22.2 µmol, 51% yield) of a pink powder. For suitable single crystals the powder were suspended in THF and 4 mg sodium trifluormethansulfonate (23.2 µmol) were added. The reaction mixture is allowed to stir for 2 hours to form a clear reddish orange solution. Single crystals were obtained via layering the mixture with few drops of *n*-hexane.

Note, the reaction always produce byproducts which could be identify as **3** and **6**.

^{1}H NMR (502.29 MHz, dimethylsulfoxide-d$_6$, 300K) δ 8.24 – 8.18 (m, 2H, CH_{ar}), 8.11 – 7.99 (m, 6H, CH_{ar}), 7.98 – 7.91 (m, 4H, CH_{ar}), 7.88 – 7.80 (m, 3H, CH_{ar}), 7.67 – 7.53 (m, 6H, CH_{ar}), 7.48 – 7.36 (m, 7H, CH_{ar}), 7.32 – 7.23 (m, 4H, CH_{ar}), 7.21 – 7.14 (m, 4H, CH_{ar}), 7.08 – 6.98 (m, 4H, CH_{ar}), 6.89 (t, J = 7.4 Hz, 1H, CH_{ar}), 6.80 (td, J = 7.8, 3.8 Hz, 2H, CH_{ar}), 6.52 (t, J = 7.7 Hz, 2H, CH_{ar}), 5.41 (m, 1H, PCH_2P), 5.13 (m, 1H, PCH_2P), 5.08 (m, obscured, 1H, Rh(PhC*H*)), 5.03 (br, m, obscured, 1H, PCH_2P, assignment via 2D ^{1}H^{13}C HSQC NMR), 3.0 (td, J = 16.8 Hz, 9.8 Hz, 1H, PCH_2P, assignment via 2D ^{1}H^{13}C HSQC NMR) ppm.

^{13}C{^{1}H} NMR (125.67 MHz, dimethylsulfoxide-*d$_6$*, 300 K) δ 139.27 (br s, 1C, HC$C_{ar\text{-}ipso}$), 135.32 (d, *J* = 3.0 Hz, 2C, CH$_{ar}$), 135.03 – 135.14 (m, 2C, CH$_{ar}$), 134.33 - 134.63 (m, 4C, CH$_{ar}$), 134.18 – 133.87 (m, 3C, CH$_{ar}$), 133.32 - 133.55 (m, 3C, CH$_{ar}$), 133.14 (d, *J* = 9.8 Hz, 3C, CH$_{ar}$), 139.92 (d, *J* = 10.9 Hz, 2C, CH$_{ar}$), 132.15 (d, *J* = 9.3 Hz, 2C, CH$_{ar}$), 131.11 Hz (br s, 1C, CH$_{ar}$), 130.86 (d, *J* = 8.4 Hz, 2C, CH$_{ar}$), 130.39 (dd, *J* = 26.0 Hz, 13.2 Hz, 4C, CH$_{ar}$), 129.47 – 129.81 (m, 3C, CH$_{ar}$), 129.17 (d, J = 12.9 Hz, 2C, CH$_{ar}$), 128.65 (m, 4C, CH$_{ar}$), 128.15 (d, *J* = 9.5 Hz, 2C, CH$_{ar}$), 127.73 - 127.94 (m, 4C, CH$_{ar}$), 127.53 (m, 6C, CH$_{ar}$), 126.87 (br s, 1C, CH$_{ar}$), 122.17 (dd, $^1J_{CP}$ = 75.0 Hz, *J* = 8.2 Hz, 1C, RhP$C_{ar\text{-}ipso}$), 121.05 (d, $^1J_{CP}$ = 50.2 Hz, 1C, CP$C_{ar\text{-}ipso}$), 120.39 (d, $^1J_{CP}$ = 38.0 Hz, 1C, CP$C_{ar\text{-}ipso}$), 117.93 (dd, $^1J_{CP}$ = 75.0 Hz, *J* = 8.2 Hz, 1C, RhP$C_{ar\text{-}ipso}$), 46.96 (br, m, 1C, Rh*C*H, very weak resonance, assignment via 2D ^{1}H^{13}C HSQC NMR), 39.86 (obscured with dmso-d$_6$, br, m, 1C, P*C*H$_2$P, very weak resonance, assignment via 2D ^{1}H^{13}C HSQC NMR), 26.47 (br, m, 1C, P*C*H$_2$P, very weak resonance, assignment via 2D ^{1}H^{13}C HSQC NMR) 19.61 (m, 1C, obscured, P*C*P very weak resonance, assignment via 2D ^{1}H^{13}C HMBC NMR) ppm.

^{31}P{^{1}H} NMR (202.30 MHz, dimethylsulfoxid-*d$_6$*) δ 38.61 (dddd, J = 39.6, 20.2, 14.1, 5.7 Hz, 1P, PC*P*), 36.83 (tdd, J = 38.5, 11.0, 3.9 Hz, 1P, PC*P*), 17.85 (ddd, $^1J_{RhP}$ = 98.9 Hz, J = 20.7,

11.2 Hz, 1P, Rh*P*), 15.40 (ddd, $^{1}J_{RhP}$ = 98.8 Hz, J = 20.8, 11.2 Hz, 1P, Rh*P*), -0.34 (ddd, $^{1}J_{RhP}$ = 109.8 Hz, J = 38.6, 14.2 Hz, 1P, Rh*P*), -2.79 (ddd, $^{1}J_{RhP}$ = 109.7 Hz, J = 38.4, 14.3 Hz, 1P, Rh*P*) ppm.

IR ATR (cm^{-1}): 3050 m, 2825 m, 1586 m, 1573 m, 1487 m, 1435 m, 1367 m, 1336 m, 1194 m, 1160 m, 1098 m, 1070 m, 1029 m, 998 m, 893 m, 837 m, 780 m, 741 s, 731 s, 691 s, 632 m, 603 m, 582 m, 536 m, 522 s, 503 s, 474 s, 449 m, 433 m, 412 m, 389 m, 378 m, 336 m, 302 m, 282 m, 258 m, 215 m, 208 s

Element.Anal. calcd. for $[C_{58}H_{50}Cl_2P_4Rh][SO_3CF_3]$·DCM: C 56.36%, H 4.10%; found: C 55.99% H 4.35 %.

HRMS calcd. m/z for M^+ $[C_{58}H_{50}Cl_2P_4Rh]^+$: 1043.12896; found: 1043.1282; Δ= -0.75 ppm calcd. m/z for M^+- {HCl,CHPh} $[C_{51}H_{43}ClP_4Rh]^+$: 917.10533; found: 917.1050; Δ= -0.38 ppm

Note, according to the synthesis complex **3** is also found in HRMS along with complex **1** after cleavage of the {CHPh} fragment and one formal HCl.

Alternative method B: 40 mg (43.5 µmol) of [({dppm}$_2$C)RhCl] (**1**) are dissolved in 3 mL benzalchloride. The red solution was layered with *n*-hexane and allowed to rest in the fridge to form a red precipitate over night. The red solid were decantated from the mother liquor and subsequently washed two times with 0.5 mL diethylether. The resulting pink solid were dissolved in dichlormethane and layered with few drops of diethylether. After 8 hours a red microcrystalline precipitate was formed. Finally the crystals were filtered of and washed three times with *n*-hexane and dried *in vacuo* overnight to get 18 mg (16.6 µmol, 38%) of a red microcrystalline powder of [({dppm}$_2$C-CHPh)RhCl$_2$]Cl (**5b-Cl**). Addition of equivalent sodium trifluormethansulfonate (16.6 µmol, 2.9 mg) in 2 mL THF leads to a clear reddish orange solution. The solvent was removed *in vacuo* to obtain a reddish pink solid, which was subsequently washed with *n*-hexane and dried *in vacuo* for 16 hours to receive [({dppm}$_2$C-CHPh)RhCl$_2$]OTf (**5b**).

Synthesis of [({dppm}$_2$CH)RhCl$_3$]Cl (6-Cl)

50.0 mg (50.5 µmol) [({dppm}$_2$C)RhCl$_3$] (**3**) were dissolved in 2 mL dichloromethane and 0.1 mL (200 µmol, 4.0 equiv.) of a 2M HCl solution in diethylether were slowly added under vigorous stirring. Immediately a discolouration of the solution and precipitate of an orange solid were observed. The reaction mixtures were allowed to rest for 15 minutes before the orange precipitate was decantated from the mother liquor and subsequently washed three times with 4 mL diethylether. Continuous drying for 24 hours at high vacuum lead to an orangish yellow powder of [({dppm}$_2$CH)RhCl$_3$]Cl (**6**). Yield: 45.1 mg (43.9 µmol, 87 %). Note, due to low solubility of **6** in various organic solvents, anion exchange was necessary using sodium tetrakis[3,5-bis(trifluoromethyl)phenyl]borate NaBArF (1:1 ratio) to get sufficient solubility in CD_3CN for NMR spectroscopy.

^{1}H NMR (499.71 MHz, acetonitrile-d$_3$, 300 K) δ 7.96 – 7.89 (m, 4H, CH_{ar}), 7.72-7.65 (m, 18H, CH_{ar}), 7.67 (s, 4H, CH_{ar}), 7.56 – 7.48 (m, 8H, CH_{ar}), 7.48 – 7.42 (m, 6H, CH_{ar}), 7.41 – 7.36 (m, 4H, CH_{ar}), 7.26 (dt, J = 15.5, 7.7 Hz, 8H, CH_{ar}), 7.21 – 7.16 (m, 4H, CH_{ar}), 5.51 (td, J = 20.3, 2.2 Hz, 1H, (dppm)$_2$CH), 4.94 – 4.83 (m, 2H, P-CH_2-P), 4.45 (ddt, J = 14.4, 9.8, 4.1 Hz, 2H, P-CH_2-P) ppm.

^{13}C{^{1}H} NMR (125.67 MHz, acetonitrile-d$_3$, 300 K) δ 162.60 (q, $^1J_{CB}$ = 49.8 Hz, 4C, B-C_{BArf}), 136.09 (dd, J = 5.2, 3.4 Hz, 4C, CH$_{ar}$), 135.83 (d, J = 11.6 Hz, 4C, CH$_{ar}$), 135.65 (s, 8C, CH$_{BArf}$), 134.66 (t, J = 5.6 Hz, 4C, CH$_{ar}$), 134.17 (t, J = 5.4 Hz, 4C, CH$_{ar}$),133.96 (d, J = 10.5 Hz, 4C, CH$_{ar}$),131.87 (d, J = 15.6 Hz, 4C, CH$_{ar}$), 131.06 (d, J = 12.5 Hz, 4C, CH$_{ar}$), 130.10 (d, J = 13.3 Hz, 4C, CH$_{ar}$), 129.81 (q, $^2J_{CF}$, 8C, CCF$_{3BArf}$ obscured), 128.84 (m , 8C, CH$_{ar}$), 128.68 (dd, $^1J_{CP}$ = 31.6 Hz, $^2J_{CP}$ = 2.8 Hz , 2C, C(H)P$C_{ar\text{-}ipso}$, partially obscured, couplings extracted from ^{13}C APT NMR spectrum), 128.68 (dd, $^1J_{CP}$ = 25.9 Hz, $^2J_{CP}$ = 2.6 Hz , 2C, C(H)P$C_{ar\text{-}ipso}$, partially obscured, couplings extracted from ^{13}C APT NMR spectrum), 125.46 (q, $^1J_{CF}$ = 271 Hz, 8C, CF$_{3BArf}$ partially obscured),118 (s, 4C, CH$_{BArf}$,superimposed), 117.08 (d, $^1J_{CP}$= 81.4 Hz, 2C, RhP$C_{ar\text{-}ipso}$), 121.59 (d, $^1J_{CP}$= 77.9 Hz, 2C, RhP$C_{ar\text{-}ipso}$), 33.80 (d, $^1J_{CP}$ = 63.7 Hz, 2C, PCH$_2$P) 8.37 (d, $^1J_{CRh}$= 29 Hz, 1C, PC(H)P, very weak resonance, assignment via 2D ^{1}H^{13}C HSQC / HMBC NMR) ppm.

^{31}P{^{1}H} NMR (202.30 MHz, acetonitrile-d$_3$, 300 K) δ 43.58 (t, J_{PP} = 30.3 Hz), 13.65 (dt, $^1J_{PRh}$= 89.8 Hz, J_{PP} = 30.3 Hz) ppm.

IR ATR (cm^{-1}): 3325 m, 3056 m, 2990 m, 2963 m, 2917 m, 2855 m, 2325 m, 1811 m, 1614 m, 1586 m, 1574 m, 1486 m, 1435 s, 1361 m, 1341 m, 1261 m, 1194 m, 1158 m, 1097 s, 1028 m, 997 m, 930 m, 882 m, 773 m, 737 s, 687 s, 640 s, 616 m, 547 m, 522 s, 503 s, 494 s, 472 s, 446 s, 409 s, 393 s, 378 s, 339 s, 298 s, 279 s, 237 s, 214 s, 206 s

Element.Anal. calcd. for $[C_{51}H_{45}Cl_4P_4Rh]{\cdot}0.5$ DCM: C 57.86%, H 4.34%; found: C 57.34% H 4.65%

HRMS calcd. m/z for M^+-HCl $[C_{51}H_{44}Cl_3P_4Rh]^+$: 988.05086; found: 988.05055; Δ= 0.31 ppm.

Synthesis of [({dppm}$_2$CH)RhCl]Cl (7-Cl)

37.0 mg (75.0 µmol) [Rh(cod)Cl_2] and 123.0 mg (150.1 µmol, 2.0 equiv.) [(dppm)$_2$CH]Cl were dissolved in 6 mL of dichloromethane to form an immediately colour change to reddish orange and stirred for 10 minutes at ambient temperature. The solvent was removed *in vacuo* to obtain a reddish orange solid, which was subsequently washed with *n*-hexane and dried *in vacuo* for 16 hours to receive [({dppm}$_2$CH)RhCl]Cl (**7**). Single crystals were obtained by layering a solution of **7** in dichloromethane with *n*-hexane. Yield: 136.2 mg (142.5 µmol, 95%).

^{1}H NMR (499.71 MHz, methanol-*d$_4$*, 300 K) δ 8.04 – 7.90 (m, 8H, CH_{ar}), 7.80 (dd, J = 12.8, 7.6 Hz, 4H, CH_{ar}), 7.63 (dd, J = 12.2, 7.6 Hz, 4H, CH_{ar}), 7.52 – 7.41 (m, 6H, CH_{ar}), 7.36 (m, 6H, CH_{ar}), 7.31 (t, J = 7.3 Hz, 4H, CH_{ar}), 7.24 (td, J = 8.0, 3.0 Hz, 4H, CH_{ar}),7.16 (td, J = 8.0, 2.9 Hz, 4H, CH_{ar}), 4.12 – 4.00 (m, 2H, PCH_2P), 3.83 – 3.74 (m, 2H, PCH_2P), PC*H*P obscured due to H/D exchange with CD_3OD.

^{13}C{^{1}H} NMR (125.67 MHz, methanol-*d$_4$*, 300 K) δ 135.33 (d, J = 2.6 Hz, 2C, CH$_{ar}$), 135.18 – 135.79 (m, 4C, partially obscured C(H)P$C_{ar\text{-}ipso}$), 134.75-134.51 (m, 12C, CH$_{ar}$), 134.72 (d, J = 2.6 Hz, 2C, CH$_{ar}$), 133.61 (br s, 2C, CH$_{ar}$), 133.53 (br s, 2C, CH$_{ar}$),131.36 (br s, 2C, CH$_{ar}$), 131.19 (br s, 2C, CH$_{ar}$),130.37 (d, J = 2.8 Hz, 4C, CH$_{ar}$), 130.26 (d, J = 2.8 Hz, 4C, CH$_{ar}$),129.55 – 129.39 (m, 8C, CH$_{ar}$), 123.77 (dt, $^1J_{CP}$= 76.6 Hz, J= ca. 3 Hz 2C, RhP$C_{ar\text{-}ipso}$), 122.72 (dt, $^1J_{CP}$ = 80.8, J= ca 3 Hz 3.0 Hz, 2C, RhP$C_{ar\text{-}ipso}$), 33.2 (br, m, 2C, PCH$_2$P, very weak resonance, assignment via 2D ^{1}H^{13}C HSQC NMR),

^{31}P{^{1}H} NMR (202.30 MHz, methanol-*d$_4$*) δ 7.19 (dt, $^1J_{PRh}$ = 143.8, J_{PP} = 37.8 Hz), 29.24 (t, J_{PP} = 37.8 Hz).

IR ATR (cm^{-1}): 3647 m, 3352 m, 3050 m, 2835 m, 2325 m, 1981 m, 1953 m, 1903 m, 1810 m, 1621 m, 1586 m, 1573 m, 1505 m, 1483 m, 1434 s, 1365 m, 1337 m, 1312 m, 1277 , 1189 m, 1139 m, 1096 s, 1026 m, 998 m, 923 m, 845 m, 776 m, 736 s, 727 s, 685 s, 629 m, 616 m, 563 m, 508 s, 475 s, 450 s, 417 s, 374 s, 286 s, 230 m, 204 s

Element.Anal. calcd for [$C_{51}H_{45}Cl_2P_4Rh$]: C 64.10%, H 4.75%; found: C 64.22% H 5.08 %.

HRMS calcd. m/z for M$^+$-HCl [$C_{51}H_{44}ClP_4Rh$]$^+$: 918.11316; found: 918.11192; Δ= 1.35 ppm.

9. Crystallographic Details

The single crystal X-ray diffraction data for the structural analysis of **1-3** and **5-7** were collected using graphite-monochromated Mo-Kα-radiation ($\lambda_{MoK\alpha}$ = 0.71073) on an imaging plate system STOE IPDS2T and IPDS2 at 170 K. Single crystal X-ray diffraction data of **4** was collected on a STADI VARI diffractometer with monochromated Ga-Kα-radiation ($\lambda_{GaK\alpha}$ = 1.34143 Å) at 150 K. The structures were solved with SHELXT in the Olex2 software version 1.5 by intrinsic phasing method and refined against F^2 by full-matrix-least- square techniques using SHELXL[9–12] Crystallographic data for **1-7** was deposited at Cambridge Crystallographic Data Centre (CCDC 2336555 (for **1**), 2336556 (for **2-OTf**), 2336557 (for **2-PF₆**), 2336558 (for **3**), 2336559 (for **4-OTf**), 2336560 (**7-Cl**), 2336561 (for **6-Cl**), 2371569 (for **5b-Cl**), and 2371571 (for **5a**),) and can be obtained free of charge via www.ccdc.cam.ac.uk/. Selected Crystallographic data is summarized in Table S1-S7 the molecular structures are depicted in Figure S1-S7.

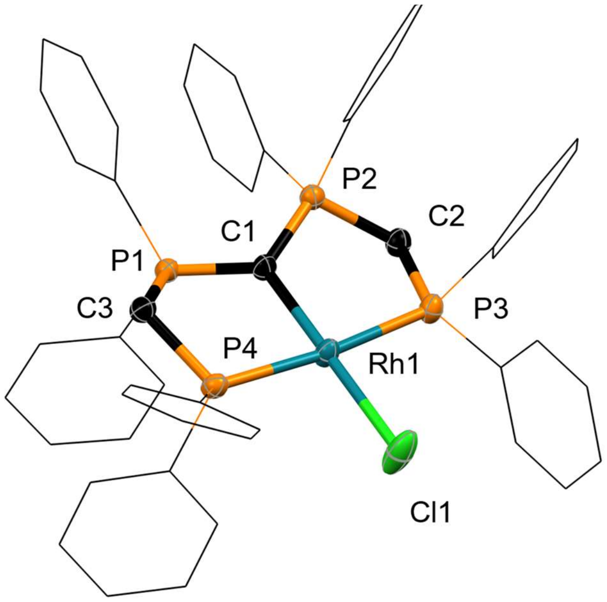

Figure S1 Molecular structure of [({dppm}$_2$C)RhCl] (**1**) derived from scXRD study (thermal ellipsoids are drawn with 50% probability, hydrogen atoms are omitted for clarity and phenyl-rings are display as wire frame).

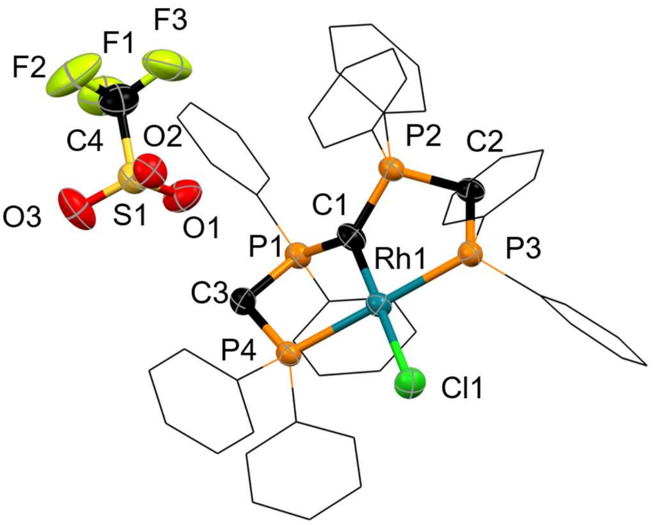

Figure S2 Molecular structure of [({dppm}$_2$C)RhCl]OTf·2THF (**2-OTf**) derived from scXRD study (thermal ellipsoids are drawn at 50% probability, hydrogen atoms and solvent molecule are omitted for clarity. Phenyl rings are display as wire frame.

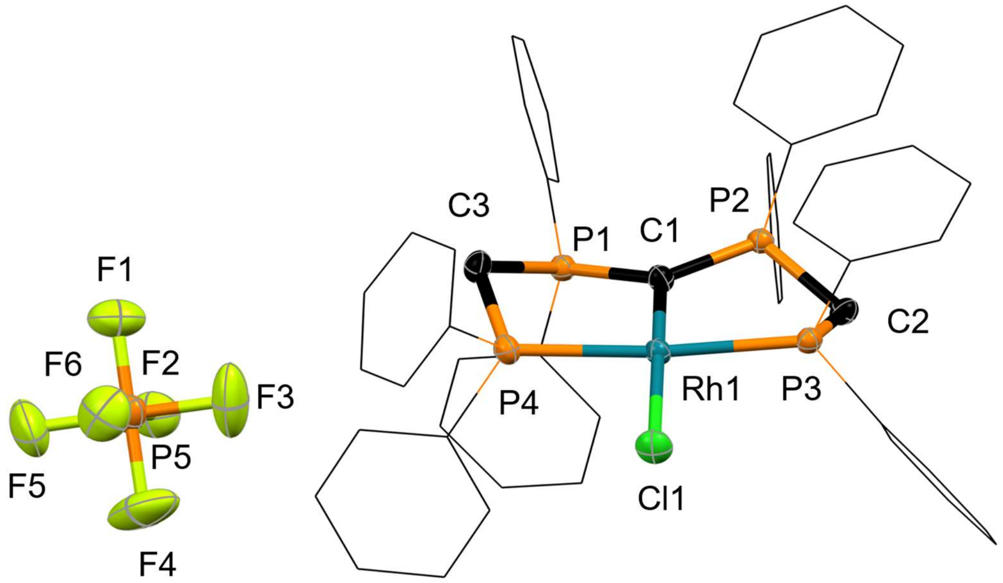

Figure S3 Molecular structure of [({dppm}$_2$C)RhCl]PF$_6$·(o-C$_6$F$_2$H$_4$) (**2-PF$_6$**) derived from scXRD study (thermal ellipsoids are drawn at 50% probability, hydrogen atoms and solvent molecule are omitted for clarity. Phenyl rings are display as wire frame.

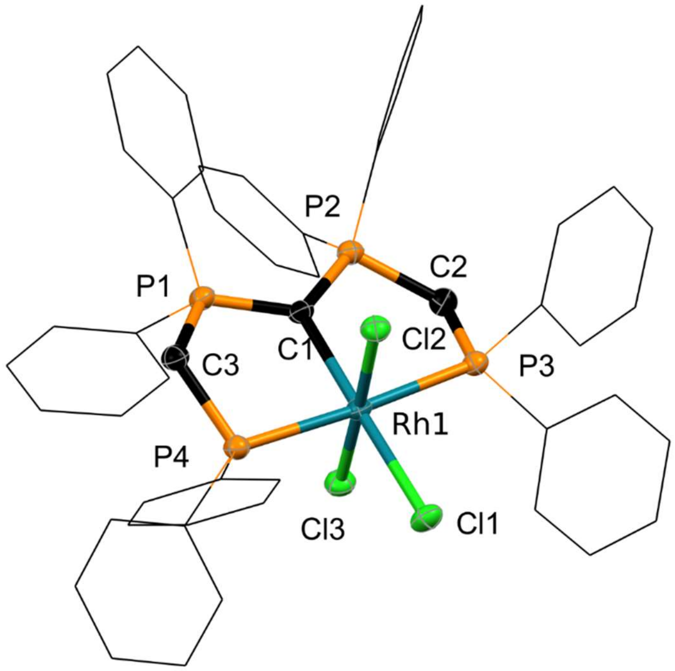

Figure S4 Molecular structure of [({dppm}$_2$C)RhCl$_3$]·Et$_2$O (**3**) derived from scXRD study (thermal ellipsoids are drawn with 50% probability, hydrogen atoms and solvent molecule are omitted for clarity. Phenyl rings are display as wire frame.

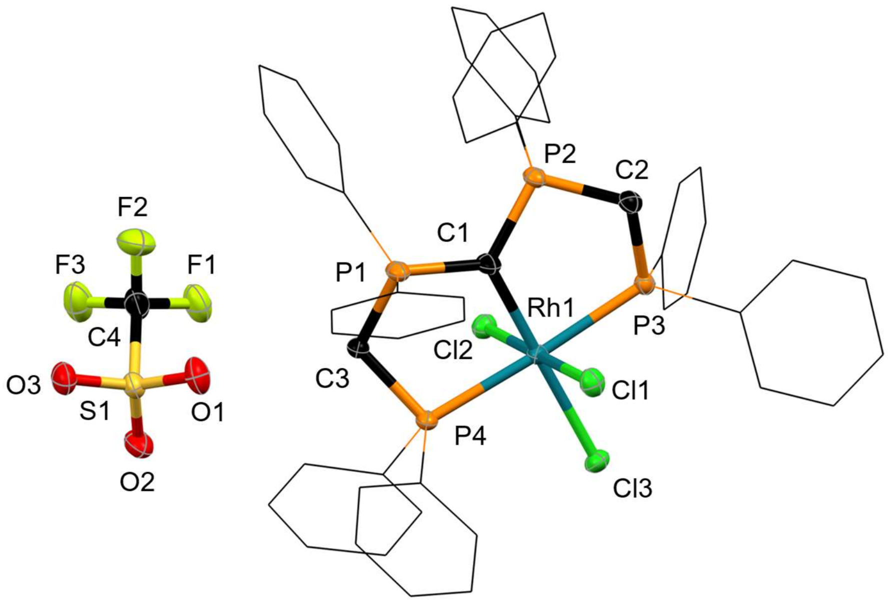

Figure S5 Molecular structure of [({dppm}$_2$C)RhCl$_3$]OTf·2THF (**4-OTf**) derived from scXRD study (thermal ellipsoids are drawn with 50% probability, hydrogen atoms and solvent molecules are omitted for clarity. Phenyl rings are display as wire frame.

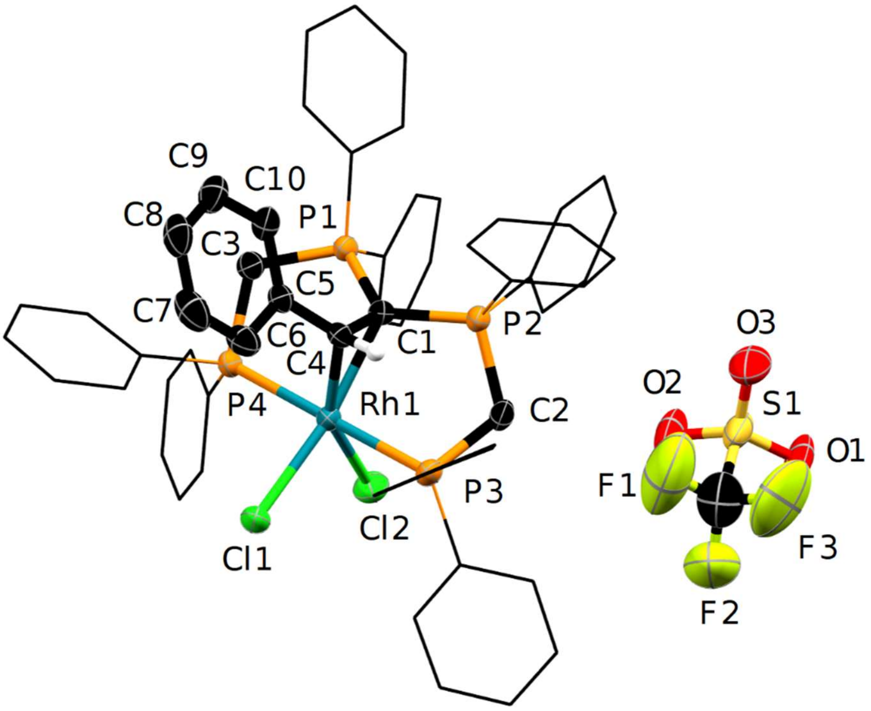

Figure S6 Molecular structure of [({dppm}$_2$C-CHPh)RhCl$_2$]OTf·2C$_7$H$_6$Cl$_2$ (**5b**).derived from scXRD study (thermal ellipsoids are drawn at 50% probability, hydrogen atoms and solvent molecules are omitted for clarity (except C4-H) and phenyl-rings are display as wire frame, except toluene moiety).

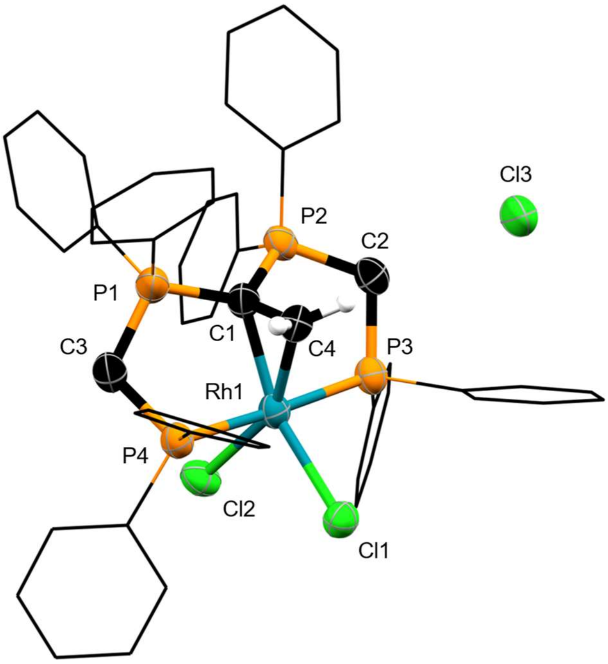

Figure S7 Molecular structure of [({dppm}$_2$C-CH$_2$)RhCl$_2$]Cl·2C$_7$H$_6$Cl$_2$ (**5a**).derived from scXRD study (thermal ellipsoids are drawn at 50% probability, hydrogen atoms and solvent molecules are omitted for clarity (except C4-H$_2$) and phenyl-rings are display as wire frame.

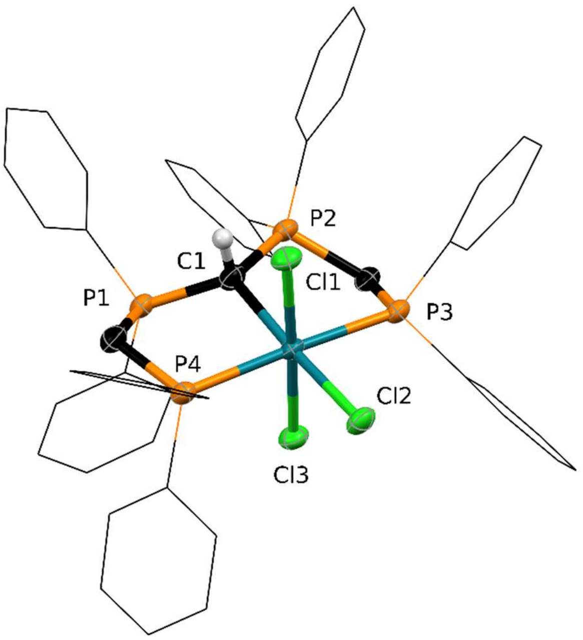

Figure S8 Molecular structure of [({dppm}$_2$CH)RhCl$_3$]Cl x 1 CH_3CN (**6**) derived from scXRD study (thermal ellipsoids are drawn at 50% probability, hydrogen atoms and solvent molecules are omitted for clarity (except C1-H) and phenyl-rings are display as wire frame).

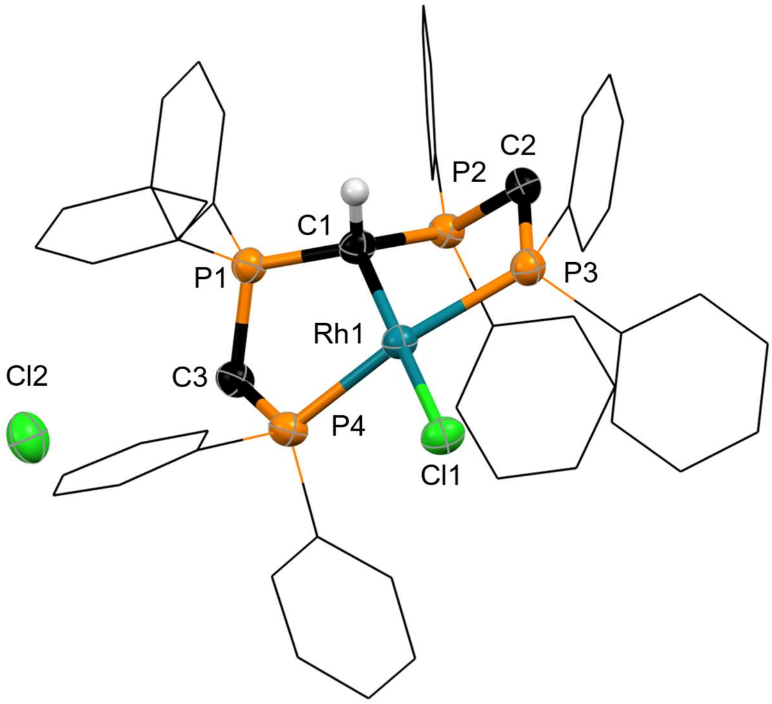

Figure S9 Molecular structure of [({dppm}$_2$CH)RhCl]Cl·1.5CH_2Cl_2 (**7-Cl**) derived from scXRD study (thermal ellipsoids are drawn at 50% probability, hydrogen atoms and solvent molecules are omitted for clarity (except C1-H) and phenyl-rings are display as wire frame).

Table S1 Crystal data and structure refinement for [({dppm}$_2$C)RhCl] (**1**).

Identification code	CCDC 2336555
Empirical formula	$C_{51}H_{44}ClP_4Rh$
Formula weight	919.10
Temperature/K	170
Crystal system	monoclinic
Space group	C2/c
a/Å	21.729(4)
b/Å	12.910(3)
c/Å	14.886(3)
α/°	90
β/°	91.50(3)
γ/°	90
Volume/Å^3	4174.3(14)
Z	4
ρ_{calc}g/cm^3	1.462
μ/mm^{-1}	0.663
F(000)	1888.0
Crystal size/mm^3	0.32 × 0.16 × 0.15
Radiation	MoKα (λ = 0.71073)
2Θ range for data collection/°	3.67 to 58.546
Index ranges	-29 ≤ h ≤ 29, -17 ≤ k ≤ 17, -20 ≤ l ≤ 17
Reflections collected	42578
Independent reflections	5645 [R_{int} = 0.0617, R_{sigma} = 0.0277]
Data/restraints/parameters	5645/0/267
Goodness-of-fit on F^2	1.034
Final R indexes [I>=2σ (I)]	R_1 = 0.0283, wR_2 = 0.0697
Final R indexes [all data]	R_1 = 0.0388, wR_2 = 0.0736
Largest diff. peak/hole / e Å^{-3}	0.56/-0.62

Table S2 Crystal data and structure refinement for [({dppm}$_2$C)RhCl]OTf·2THF (**2-OTf**).

Identification code	CCDC 2336556
Empirical formula	$C_{60}H_{60}ClF_3O_5P_4RhS$
Formula weight	1212.38
Temperature/K	170
Crystal system	monoclinic
Space group	$P2_1/n$
a/Å	14.036(3)
b/Å	25.123(5)
c/Å	17.957(4)
α/°	90
β/°	104.93(3)
γ/°	90
Volume/Å^3	6118(2)
Z	4
ρ_{calc}g/cm^3	1.316
μ/mm^{-1}	0.515
F(000)	2500.0
Crystal size/mm^3	0.19 × 0.13 × 0.08
Radiation	MoKα (λ = 0.71073)
2Θ range for data collection/°	3.242 to 51.998
Index ranges	-17 ≤ h ≤ 16, -30 ≤ k ≤ 27, -22 ≤ l ≤ 22
Reflections collected	31837
Independent reflections	12008 [R_{int} = 0.1564, R_{sigma} = 0.1494]
Data/restraints/parameters	12008/0/628
Goodness-of-fit on F^2	0.969
Final R indexes [I>=2σ (I)]	R_1 = 0.0710, wR_2 = 0.1604
Final R indexes [all data]	R_1 = 0.1528, wR_2 = 0.2031
Largest diff. peak/hole / e Å^{-3}	0.80/-1.15

Table S3 Crystal data and structure refinement for $[(\{dppm\}_2C)RhCl]PF_6 \cdot C_6H_4F_2$ (**2-PF$_6$**).

Identification code	CCDC 2336557
Empirical formula	$C_{54}H_{46}ClF_7P_5Rh$
Formula weight	1121.12
Temperature/K	170
Crystal system	monoclinic
Space group	$P2_1/n$
a/Å	18.4657(4)
b/Å	11.6960(2)
c/Å	24.1370(5)
α/°	90
β/°	92.303(2)
γ/°	90
Volume/Å^3	5208.77(18)
Z	4
ρ_{calc}g/cm^3	1.430
μ/mm^{-1}	0.593
F(000)	2280.0
Crystal size/mm^3	0.6 × 0.367 × 0.2
Radiation	MoKα (λ = 0.71073)
2Θ range for data collection/°	4.852 to 58.5
Index ranges	-25 ≤ h ≤ 23, -15 ≤ k ≤ 16, -33 ≤ l ≤ 33
Reflections collected	40212
Independent reflections	13968 [R_{int} = 0.0486, R_{sigma} = 0.0376]
Data/restraints/parameters	13968/0/601
Goodness-of-fit on F^2	1.075
Final R indexes [I>=2σ (I)]	R_1 = 0.0485, wR_2 = 0.1274
Final R indexes [all data]	R_1 = 0.0687, wR_2 = 0.1405
Largest diff. peak/hole / e Å^{-3}	1.36/-0.56

Table S4 Crystal data and structure refinement for [({dppm}$_2$C)RhCl$_3$]·Et$_2$O (**3**).

Identification code	CCDC 2336558
Empirical formula	$C_{55}H_{54}Cl_3OP_4Rh$
Formula weight	1064.12
Temperature/K	170
Crystal system	monoclinic
Space group	$P2_1/n$
a/Å	13.2305(6)
b/Å	24.3223(13)
c/Å	16.4471(7)
α/°	90
β/°	96.415(3)
γ/°	90
Volume/Å^3	5259.5(4)
Z	4
ρ_{calc}g/cm^3	1.344
μ/mm^{-1}	0.636
F(000)	2192.0
Crystal size/mm^3	0.4 × 0.233 × 0.1
Radiation	MoKα (λ = 0.71073)
2Θ range for data collection/°	4.984 to 58.408
Index ranges	-18 ≤ h ≤ 18, -33 ≤ k ≤ 28, -22 ≤ l ≤ 22
Reflections collected	39243
Independent reflections	14079 [R_{int} = 0.0540, R_{sigma} = 0.0523]
Data/restraints/parameters	14079/0/573
Goodness-of-fit on F^2	0.983
Final R indexes [I>=2σ (I)]	R_1 = 0.0345, wR_2 = 0.0739
Final R indexes [all data]	R_1 = 0.0601, wR_2 = 0.0800
Largest diff. peak/hole / e Å^{-3}	0.70/-0.56

Table S5 Crystal data and structure refinement for [({dppm}$_2$C)RhCl$_3$]OTf (**4-OTf**).

Identification code	CCDC 2336559
Empirical formula	$C_{52}H_{44}Cl_3F_3O_3P_4RhS$
Formula weight	1139.07
Temperature/K	150
Crystal system	triclinic
Space group	P-1
a/Å	11.9084(4)
b/Å	12.8508(4)
c/Å	19.2819(6)
α/°	73.137(3)
β/°	78.541(3)
γ/°	69.103(2)
Volume/Å^3	2623.14(16)
Z	2
ρ_{calc}g/cm^3	1.442
μ/mm^{-1}	3.950
F(000)	1158.0
Crystal size/mm^3	0.15 × 0.10 × 0.02
Radiation	Ga Kα (λ = 1.34143)
2Θ range for data collection/°	6.918 to 125.014
Index ranges	-7 ≤ h ≤ 15, -16 ≤ k ≤ 16, -23 ≤ l ≤ 25
Reflections collected	29647
Independent reflections	12254 [R_{int} = 0.0186, R_{sigma} = 0.0166]
Data/restraints/parameters	12254/15/611
Goodness-of-fit on F^2	1.056
Final R indexes [I>=2σ (I)]	R_1 = 0.0386, wR_2 = 0.1073
Final R indexes [all data]	R_1 = 0.0408, wR_2 = 0.1087
Largest diff. peak/hole / e Å^{-3}	1.12/-0.69

Table S6 Crystal data and structure refinement for [({dppm}$_2$C-CH$_2$)RhCl$_2$]Cl·2C$_7$H$_6$Cl$_2$ (**5a**).

Identification code	CCDC 2371570
Empirical formula	$C_{55}H_{52}Cl_9P_4Rh$
Formula weight	1258.80
Temperature/K	170
Crystal system	monoclinic
Space group	C2/c
a/Å	18.975(4)
b/Å	16.198(3)
c/Å	37.033(7)
α/°	90
β/°	103.92(3)
γ/°	90
Volume/Å^3	11048(4)
Z	8
ρ_{calc}g/cm^3	1.514
μ/mm^{-1}	0.898
F(000)	5120.0
Crystal size/mm^3	0.31 × 0.2 × 0.13
Radiation	MoKα (λ = 0.71073)
2Θ range for data collection/°	2.266 to 51.46
Index ranges	-23 ≤ h ≤ 23, -19 ≤ k ≤ 19, -44 ≤ l ≤ 45
Reflections collected	48069
Independent reflections	10418 [R_{int} = 0.1143, R_{sigma} = 0.0601]
Data/restraints/parameters	10418/0/511
Goodness-of-fit on F^2	1.046
Final R indexes [I>=2σ (I)]	R_1 = 0.0641, wR_2 = 0.1833
Final R indexes [all data]	R_1 = 0.0782, wR_2 = 0.1960
Largest diff. peak/hole / e Å^{-3}	1.47/-1.5

Table S7 Crystal data and structure refinement for [({dppm}$_2$C-CHPh)RhCl$_2$]Cl·2C$_7$H$_6$Cl$_2$ (**5b-Cl**).

Identification code	CCDC 2371569
Empirical formula	$C_{73}H_{62}Cl_6F_3O_3P_4RhS$
Formula weight	1193.74
Temperature/K	170
Crystal system	orthorhombic
Space group	$P2_12_12_1$
a/Å	11.0535(2)
b/Å	25.1092(6)
c/Å	26.4595(6)
α/°	90
β/°	90
γ/°	90
Volume/Å^3	7343.7(3)
Z	4
ρ_{calc}g/cm^3	1.080
μ/mm^{-1}	0.462
F(000)	2440.0
Crystal size/mm^3	0.4 × 0.35 × 0.3
Radiation	MoKα (λ = 0.71073)
2Θ range for data collection/°	3.244 to 55.888
Index ranges	-14 ≤ h ≤ 12, -33 ≤ k ≤ 33, -34 ≤ l ≤ 31
Reflections collected	50330
Independent reflections	17166 [R_{int} = 0.0488, R_{sigma} = 0.0408]
Data/restraints/parameters	17166/0/635
Goodness-of-fit on F^2	0.986
Final R indexes [I>=2σ (I)]	R_1 = 0.0348, wR_2 = 0.0847
Final R indexes [all data]	R_1 = 0.0439, wR_2 = 0.0887
Largest diff. peak/hole / e Å^{-3}	0.57/-0.27
Flack parameter	0.51(2)

Table S8 Crystal data and structure refinement for [({dppm}$_2$CH)RhCl$_3$]Cl x CH_3CN (**6-Cl**).

Identification code	CCDC 2336561
Empirical formula	$C_{51}H_{45}Cl_4P_4Rh$ x CH_3CN
Formula weight	1067.51
Temperature/K	170
Crystal system	triclinic
Space group	P-1
a/Å	11.8080(9)
b/Å	11.9659(8)
c/Å	20.0343(15)
α/°	72.891(5)
β/°	78.105(6)
γ/°	68.309(5)
Volume/Å^3	2498.7(3)
Z	2
ρ_{calc}g/cm^3	1.419
μ/mm^{-1}	0.720
F(000)	1092.0
Crystal size/mm^3	0.4 × 0.3 × 0.2
Radiation	Mo Kα (λ = 0.71073)
2Θ range for data collection/°	4.504 to 52.044
Index ranges	-14 ≤ h ≤ 14, -14 ≤ k ≤ 14, -23 ≤ l ≤ 24
Reflections collected	20969
Independent reflections	9774 [R_{int} = 0.1294, R_{sigma} = 0.0931]
Data/restraints/parameters	9774/0/578
Goodness-of-fit on F^2	1.059
Final R indexes [I>=2σ (I)]	R_1 = 0.0624, wR_2 = 0.1818
Final R indexes [all data]	R_1 = 0.0825, wR_2 = 0.1994
Largest diff. peak/hole / e Å^{-3}	1.89/-1.84

Table S9 Crystal data and structure refinement for [({dppm}$_2$CH)RhCl]Cl·1.5CH$_2$Cl$_2$ (**7-Cl**).[1]

Identification code	CCDC 2336560
Empirical formula	$C_{105}H_{95}Cl_{10}P_8Rh_2$
Formula weight	2164.88
Temperature/K	170
Crystal system	trigonal
Space group	R-3
a/Å	46.2473(13)
b/Å	46.2473(13)
c/Å	25.3093(8)
α/°	90
β/°	90
γ/°	120
Volume/Å^3	46880(3)
Z	18
ρ_{calc}g/cm^3	1.380
μ/mm^{-1}	0.741
F(000)	19890.0
Crystal size/mm^3	0.4 × 0.22 × 0.15
Radiation	Mo Kα (λ = 0.71073)
2Θ range for data collection/°	2.594 to 53.542
Index ranges	-58 ≤ h ≤ 58, -58 ≤ k ≤ 52, -31 ≤ l ≤ 32
Reflections collected	84574
Independent reflections	21971 [R_{int} = 0.0736, R_{sigma} = 0.0508]
Data/restraints/parameters	21971/0/1129
Goodness-of-fit on F^2	1.063
Final R indexes [I>=2σ (I)]	R_1 = 0.0528, wR_2 = 0.1427
Final R indexes [all data]	R_1 = 0.0741, wR_2 = 0.1564
Largest diff. peak/hole / e Å^{-3}	1.43/-1.74

[1] two independent molecules in asymmetric unit

10. EPR Spectroscopy

CW-EPR measurements were performed on two frequencies X- (9.4 GHz) and Q- (34GHz) to resolve both A-tensor and g-tensor elements. Room temperature X-Band (9.43 GHz) CW-EPR measurements were performed on a Magnettech MiniScope MS400 benchtop spectrometer (now Bruker BioSpin, Ettlingen). Spectra were recorded with a microwave power of 1 mW, 100 KHz modulation frequency, modulation amplitude of 0.05 mT and 4096 points. Q-band CW-EPR (33.9 GHz) measurements were conducted on a Bruker EMX-plusQ spectrometer, using an ER5106QT resonator. A microwave power of 0.1mW was applied for all samples and for all background measurements. Unless noted otherwise, a modulation amplitude of 0.5mT was used during measurements. The modulation frequency was set to 100 kHz and measuring temperature was varied from 150 to 20 K. A Sumitomo cryo compressor-F70 was used for cooling together with a Mercury iTC (Oxford Instruments) to control the temperature.
All pulse EPR measurement were conducted at X-band on an Elexsys E580 EPR spectrometer equipped with an MS3-114 resonator (Bruker Biospin, Ettingen) equipped with 300 W solid state amplifier and AWG unit. A closed cycle cryostat (ARS- 4WH, www.arscryo.com) was used for cooling during measurements down to 20 K. For all $\frac{\pi}{2}$ microwave pulses a 16 ns pulse length and for π microwave pulses a 32 ns pulse length were applied. The Hahn echo sequence[13] ($\frac{\pi}{2} - \tau - \pi - \tau - echo$) -used to collect field swept spectra with an initial inter-pulse delay of τ =176 ns. The three-pulse ESEEM (hereafter 3PE, $\frac{\pi}{2} - \tau - \frac{\pi}{2} - T - \frac{\pi}{2} - \tau - echo$) were used with four step phase cycling to eliminate the unwanted echoes.[14,15] The chosen τ value was 176 ns and the second inter-pulsed delay (T) was set to 300 ns, incremented in 16 ns steps. The obtained 3PE time traces were processed using a home-written MATLAB (MATLAB R2022b, the MathWorks & Inc., Natick, MA, USA) code. We first corrected the baseline (using a stretched exponential function), then apodized it with a Hamming window and finally zero-filled to 1024 points. At last, Fourier transformation provided the magnitude spectra. We used a conventional four pulse HYSCORE[16] ($\frac{\pi}{2} - \tau - \frac{\pi}{2} - t1 - \pi - \mathrm{t2} - \frac{\pi}{2} - \tau - echo$) where t_1 and t_2 are incremented mixing times. The typical value of 176 ns was chosen for the inter-pulse time τ and the delay times $t_1 = t_2$ were set to 300 ns initially. A time increment of 16 ns together with an eight step phase cycling were used. All HYSCORE data were processed as mentioned above.

The EasySpin software package (version 6.0.0-dev.51) was used for spectral simulations of EPR spectra. We considered natural abundancy of nuclei through simulations.[17]

EPR sample preparation:

Samples solutions of complex **2-OTf** and complex **4-BArF** were prepared in a glove box under argon atmosphere (2 mM solutions in THF), filled into 3 mm (outer diameter) EPR quartz tubes (Qsil, Germany) and subsequently flame-sealed. The sample solutions in the quartz tubes were vitrified in pre-cooled 2-methyl butane and frozen in liquid nitrogen prior their insertion into the resonator, immediately before measurement.

EPR spectroscopic data concerning [({dppm}$_2$C)RhCl]OTf (2-OTf)

Table S10 Spin Hamiltonian parameters of complex **2** obtained by spectral simulations.*

	X-band (9.4 GHz, 20 K)	Q-band (34 GHz, 50 K)
Complex **2**	[g_{xx}, g_{yy}, g_{zz}], ^{103}Rh [A_{xx}, A_{yy}, A_{zz}] /MHz	[g_{xx}, g_{yy}, g_{zz}], ^{103}Rh [A_{xx}, A_{yy}, A_{zz}] /MHz
	isomer1 (0.8)<br><br>g = [2.600 2.087 2.002], A=[40 70 120]<br>g-Strain = [0.11 0.01 0.002]<br>g-Frame = [0 30 0]<br><br>*isomer2* (0.2)<br>g = [2.10 2.015 2.001], A=[10 30 60]	*isomer1 (1.0)*<br>g =[2.580 2.095 2.000], A=[50 70 120]

* in parenthesis is the contribution percent of each isomer is given.

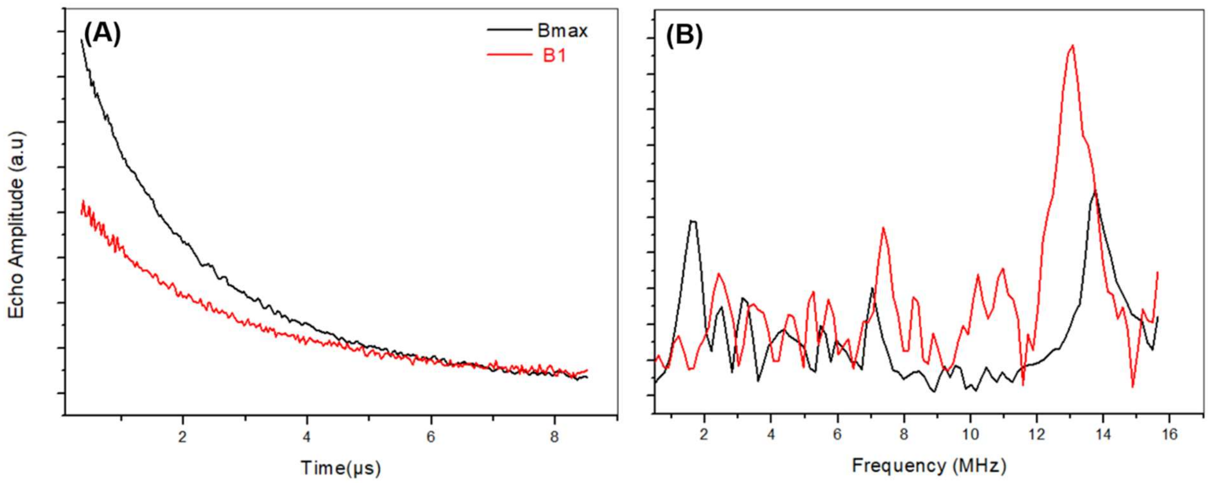

Figure S10 X-band three pulse ESEEM spectra of complex **2** at 20 K. The measured time traces (**A**) and Fourier transformed magnitude spectra (**B**) are shown for field positions B_{max} (in black) and B_1 (in red). Corresponding field position are shown in Fig. 3A.

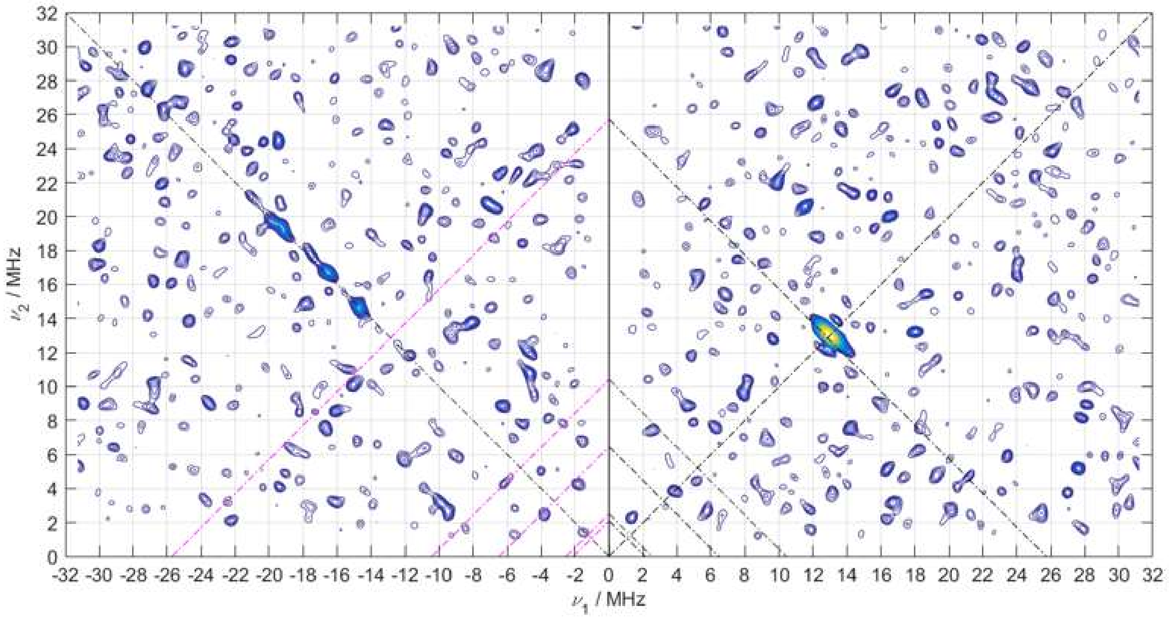

Figure S11 HYSCORE spectrum of complex **2** measured at B_1 position at 20 K, which reveals the matrix protons (Larmor frequency ~14MHz).

EPR spectroscopic data concerning [({dppm}$_2$C)RhCl$_3$]BAr^F (4-BArF)

Table S11 Spin Hamiltonian parameters of complex **4** obtained by spectral simulations

	X-band (9.4 GHz, 298 K)	X-band (9.4 GHz, 50 K)
Complex **4**	[g_{xx}, g_{yy}, g_{zz}], [A_{xx}, A_{yy}, A_{zz}] /MHz	[g_{xx}, g_{yy}, g_{zz}], [A_{xx}, A_{yy}, A_{zz}] /MHz
	g = [2.080 2.025 2.015]<br>A (^{103}Rh) = [27 27 27]<br>A(^{31}P, ^{31}P) = [74 74 74; 75 75 75]	*isomer1* (0.8)<br>g = [2.085 2.060 2.039], A (^{103}Rh) = [10 65 10]<br>*isomer2* (0.2)<br>g = [2.105 2.065 2.008], A (^{103}Rh) = [10 30 10]

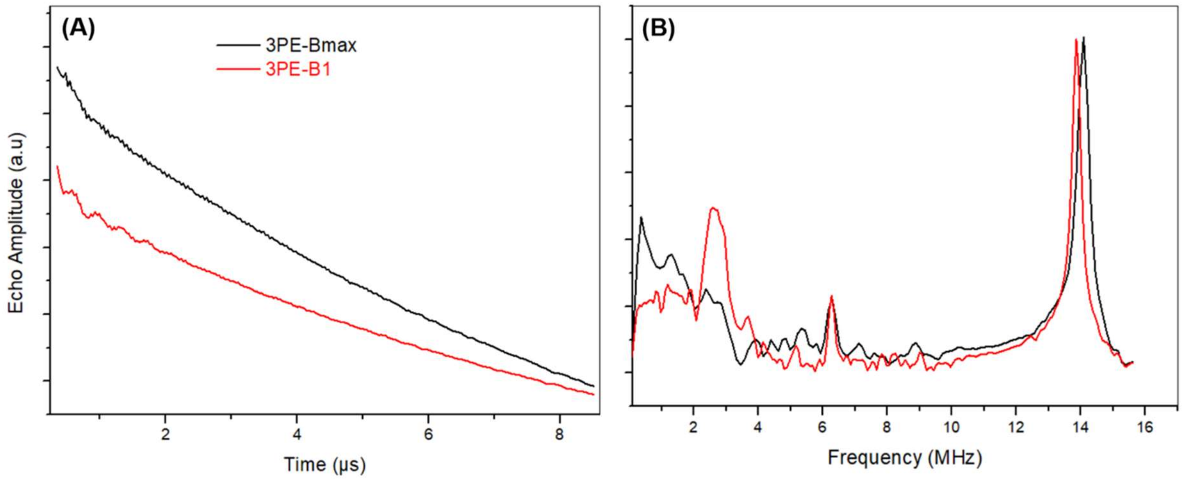

Figure S12 X-band three pulse ESEEM spectra of complex **4** at 30 K. (**A**) ESEEM time traces at two field position. (**B**) Corresponding Fourier transformed, magnitude spectra at different field positions. Corresponding field position are shown in Fig. 3B.

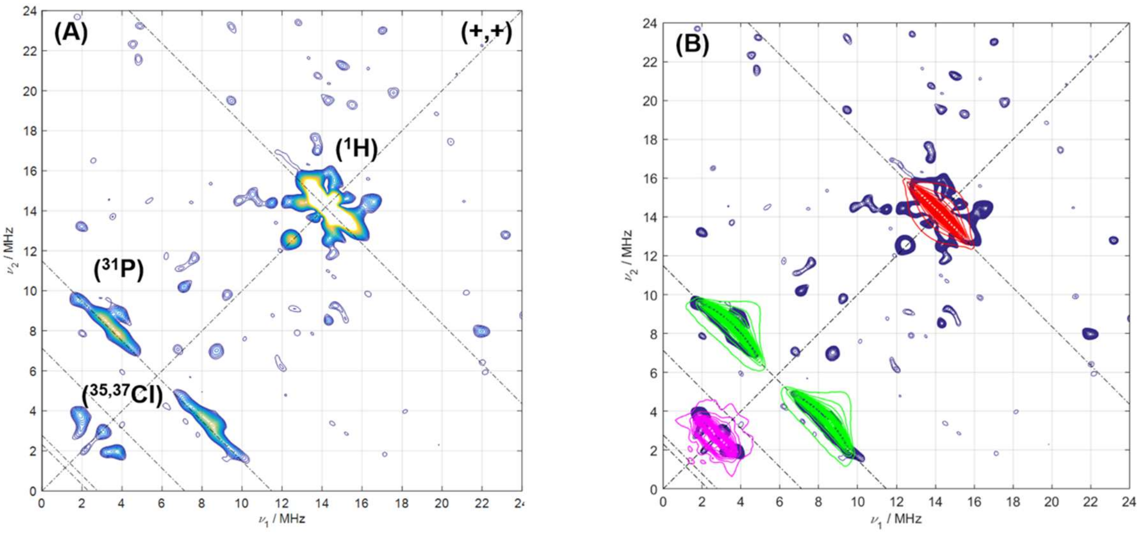

Figure S13 HYSCORE spectrum of complex **4** measured at 30 K at B_{max} **.** (**A**) Experimental and (**B**) corresponding simulation at weakly coupled quadrant show delocalization of spin density over chlorine and phosphorous nuclei, as well as pmatrix protons.

Table S12 Calculated spin Hamiltonian parameters for complex **2** and **4**.*

**Complex 2**		**Complex 4**	
nuclei	A_{iso}(MHz)	nuclei	A_{iso}(MHz)
^{14}C	27.19	^{28}C	29.32
^{6}C	4.48	^{29}C	14.87
^{9}C	8.00	^{30}C	28.55
^{26}C	23.38	^{31}C	17.42
^{36}C	23.43	^{7}C	91.15
^{4}Cl**	5.6<br>e^2qQ=-23.02, η=0.97	^{2}Cl	1.48<br>e^2qQ=-32.88, η=0.46
^{2}P	-10.05	^{3}Cl	4.36<br>e^2qQ=-32.88, η=0.46
^{3}P	-11.47	^{4}Cl	1.18<br>e^2qQ=-44.64, η=0.03
^{12}P	-27.12	^{14}P	-47.15
^{19}P	-25.40	^{15}P	-46.86

*Hyperfine couplings are calculated for all atoms at BP86 def2-SVP def2-SVP/J level of theory. For Rh we have used def2-TZVPP. Atom numbering for **2** and **4** are according to Figure S16 below.** For all chlorine nuclei the calculated quadrupolar couplings (e^2qQ) and asymmetry parameter (η) are also given.

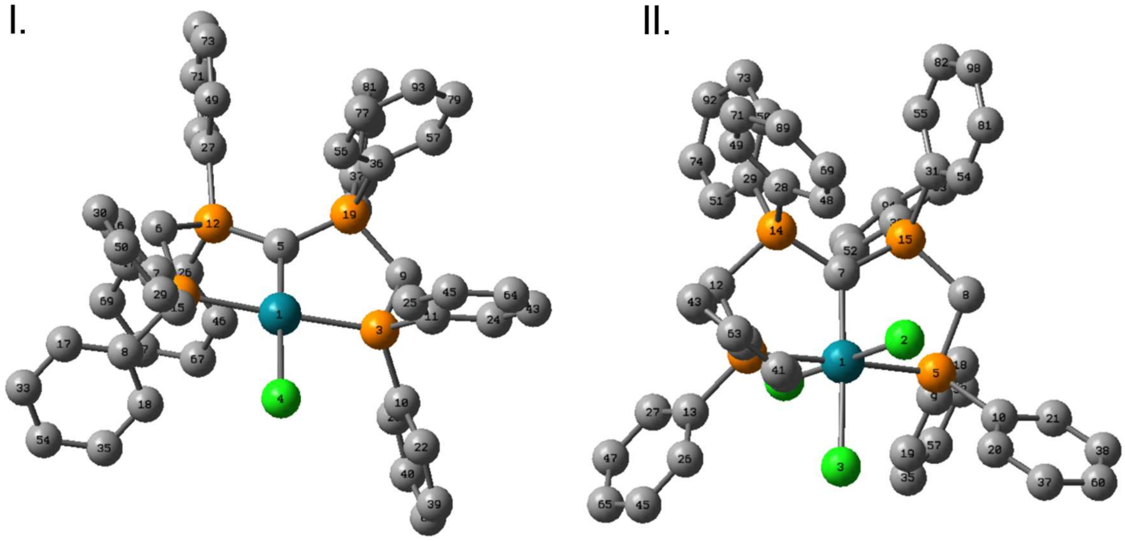

Figure S14 Numbering scheme for calculated spin Hamiltonian parameters for complex **2** and **4.**

11. Computational Details

Gaussian16[18] Revision B.01 was used to perform DFT calculations with different functionals: BP86,[19,20] B97D3[21,22] and ωB97X-D functional.[23] Geometry optimizations were performed with the def2-TZVP and the def2-TZVPP basis set.[24,25] Frequency calculations of optimized structures were performed at the same level of theory to characterize the structures to be minima. xyz-coordinates of all minima states are available as separate xyz-files. All natural bond orbital (NBO) analysis were performed using the NBO 3.0 package.[26] The spin densities for complexes **2** and **4** are summarized in Table S13 at different levels of theory. Laplacian contour line plots (Figure 5) were created with the program Multiwfn.[27]

Table S13 Calculated spin densities at different levels of theory.

	C^{CDP}	Rh	*trans*-Cl	P^{CDP}	P^{CDP}	C^{ipso}	C^{ipso}	C^{ipso}	C^{ipso}
$[(cdp)RhCl_3]^+$ (**4**)									
wB97xd / def2-TZVPP	61.78%	17.77%	0.80%	2.79%	2.78%	1.63%	2.61%	2.65%	1.67%
wB97xd / def2-TZVP	64.33%	17.04%	0.67%	1.90%	1.89%	1.44%	2.56%	2.59%	1.46%
BP86 / def2-TZVPP	52.47%	31.24%	9.17%	-0.01	-0.01	2.64%	0.75%	2.54%	1.03%
BP86 / def2-TZVP	52.34%	39.87%	9.54%	-0.09	-0.32	2.75%	0.70%	2.59%	1.02%
B97D3 / def2-TZVP	56.35%	29.08%	8.50%	0.37%	0.19%	2.35%	0.77%	2.18%	0.95%
$[(cdp)RhCl]^+$ (**2**)									
wB97xd / def2-TZVPP	9.12%	80.87%	5.75%	2.56%	2.56%	0.30%	0.03%	0.81%	0.02%
BP86 / def2-TZVPP	19.80%	65.43%	9.48%	3.22%	3.38%	0.85%	-0.49%	0.91%	-0.68%
BP86 / def2-TZVP	18.75%	65.81	10.26%	3.27%	4.15%	0.64%	-0.52%	0.68%	0.82%
B97D3 / def2-TZVP	19.52%	66.31%	9.95%	4.79%	5.10%	0.20%	-0.53%	0.29%	-0.82%

Calculation of Hyperfine couplings and spin densities were conducted by ORCA 3.0.3 software package developed by Neese,[28] at uB3LYP/def2-SVP (Cl, H, P, and C atoms) and uB3LYP/def2-TZVP ZORA (for Rh).[29] Since the THF and toluene solvents are among low dielectric media and made no big influence on the optimized structures, therefore only the gas phase optimzed structures are reported. The optimized structure was then checked for having imaginary frequencies indicating if the structure is in a true minimum on the potential energy surface or not. It turned out that the structures is a real minimum energy structure.

Educts, intermediates, transition states and products in the pathway of the activation of geminal dichlorides, were optimized by the density functional theory (DFT) with Grimme's B97D3 functional and the def2svp basis set in the gas phase using Gaussian16. Frequency analysis calculations of optimized structures were performed at the same level of theory (B97D3/def2-SVP) to characterize the structures to be minima (no imaginary frequency) or transition states (one imaginary frequency). Based on the B97D3/def2-SVP optimized geometries, the energy

results were further refined by calculating the single point energy at the B97D3/def2tzvp level of theory. The bulky solvation effect of THF was simulated by SMD continuum solvent mode at the B97D3/def2tzvp level of theory.[30] Intrinsic reaction coordinate (IRC) calculations were performed to confirm the connection between two correct minima for a transition state.

12. Spectra and Voltammograms for **1-7**

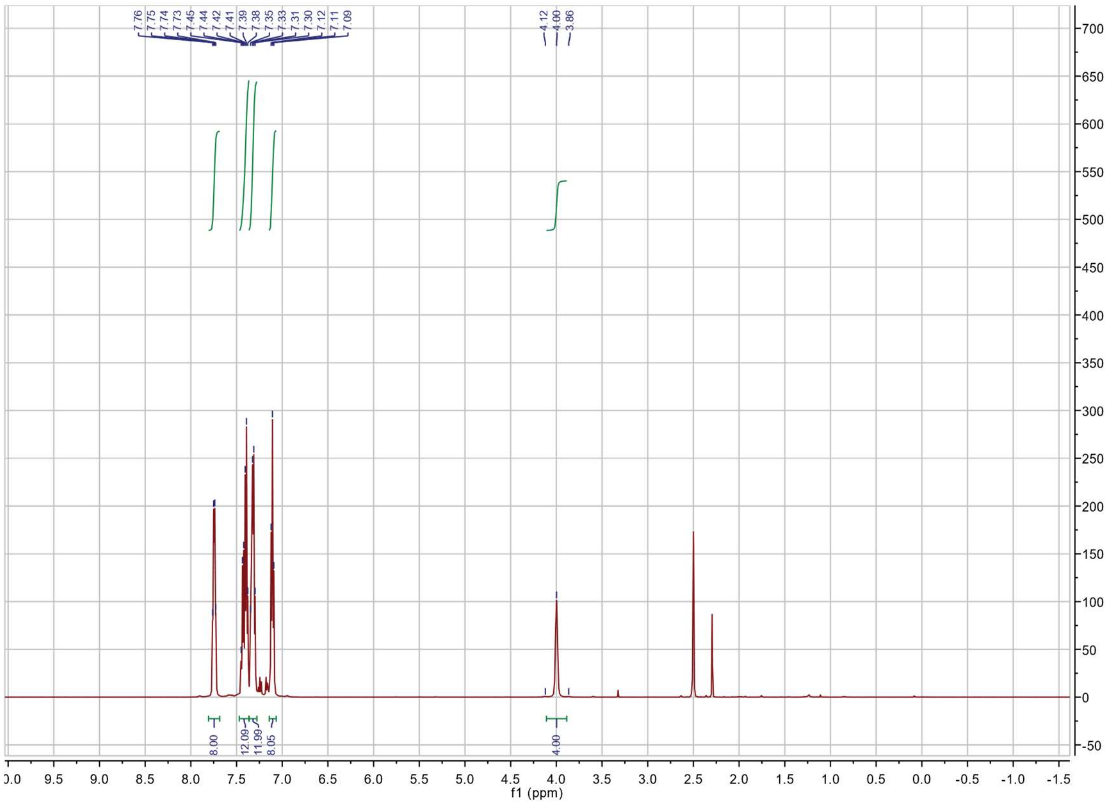

Figure S15 ^{1}H NMR spectrum (499.71 MHz, dimethylsulfoxide-d_6, 300 K) of [({dppm}$_2$C)RhCl] (**1**).

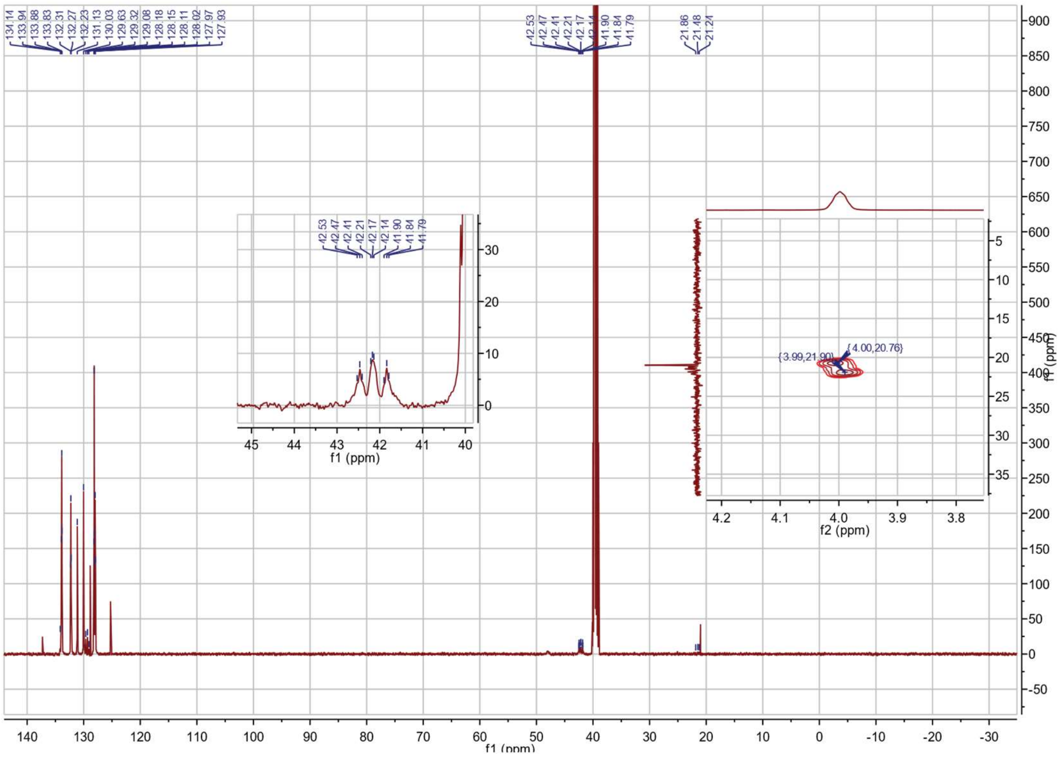

Figure S16 ^{13}C{^{1}H} NMR spectrum (125.67 MHz, dimethylsulfoxide-d$_6$, 300 K) and insert of a section of the 2D ^{1}H^{13}C HMBC NMR spectrum of [({dppm}$_2$C)RhCl] (**1**).

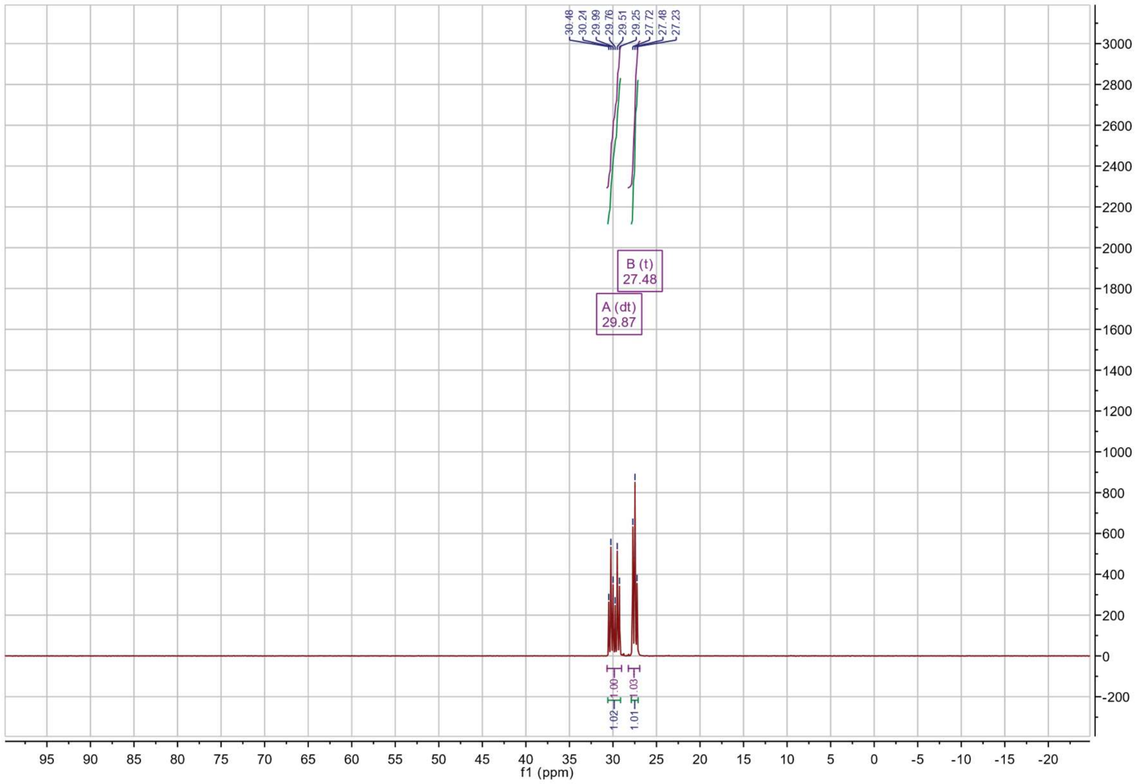

Figure S17 $^{31}P\{^{1}H\}$ NMR spectrum (202.30 MHz, dimethylsulfoxide-d$_6$, 300 K) of [({dppm}$_2$C)RhCl] (**1**).

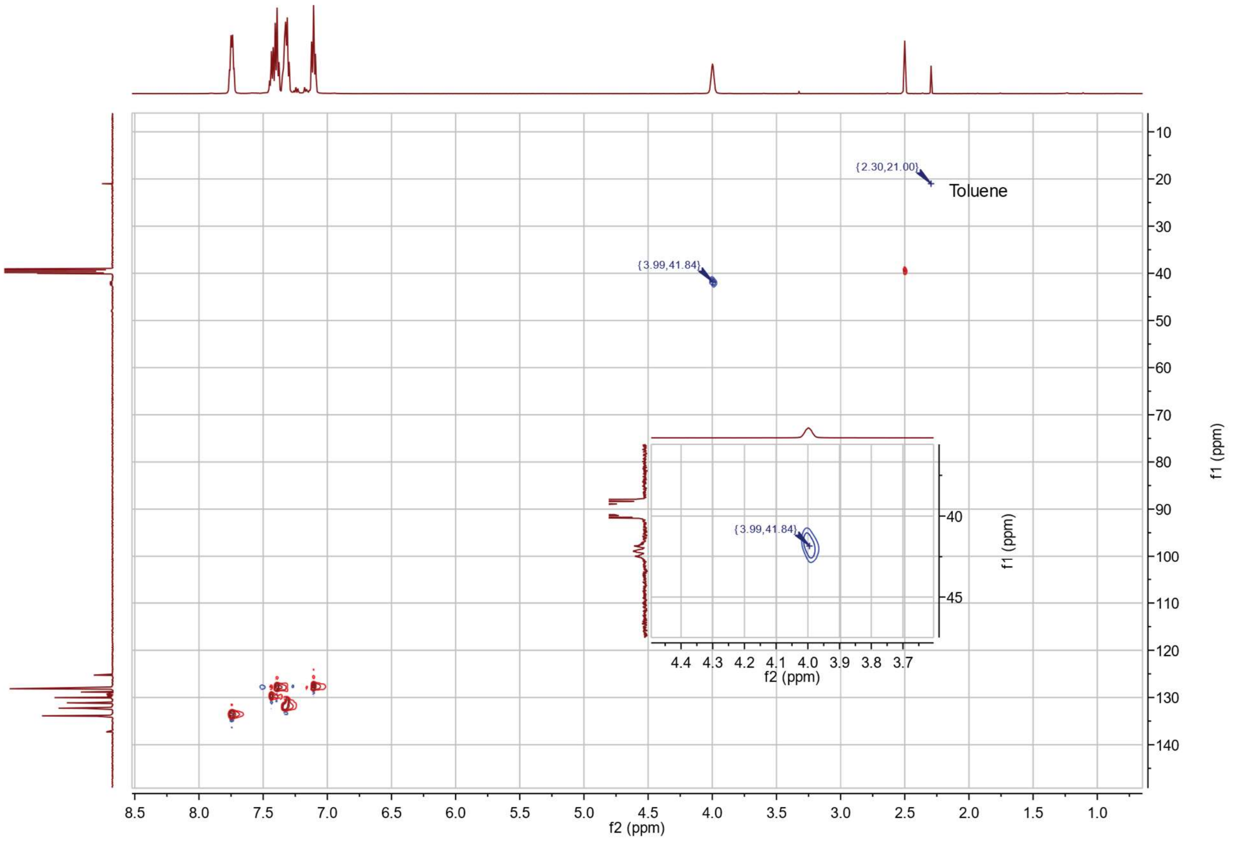

Figure S18 $^{1}H^{13}C$ HSQC NMR spectrum (499.72, 125.66 MHz, dimethylsulfoxide-d$_6$, 300 K) of [({dppm}$_2$C)RhCl] (**1**).

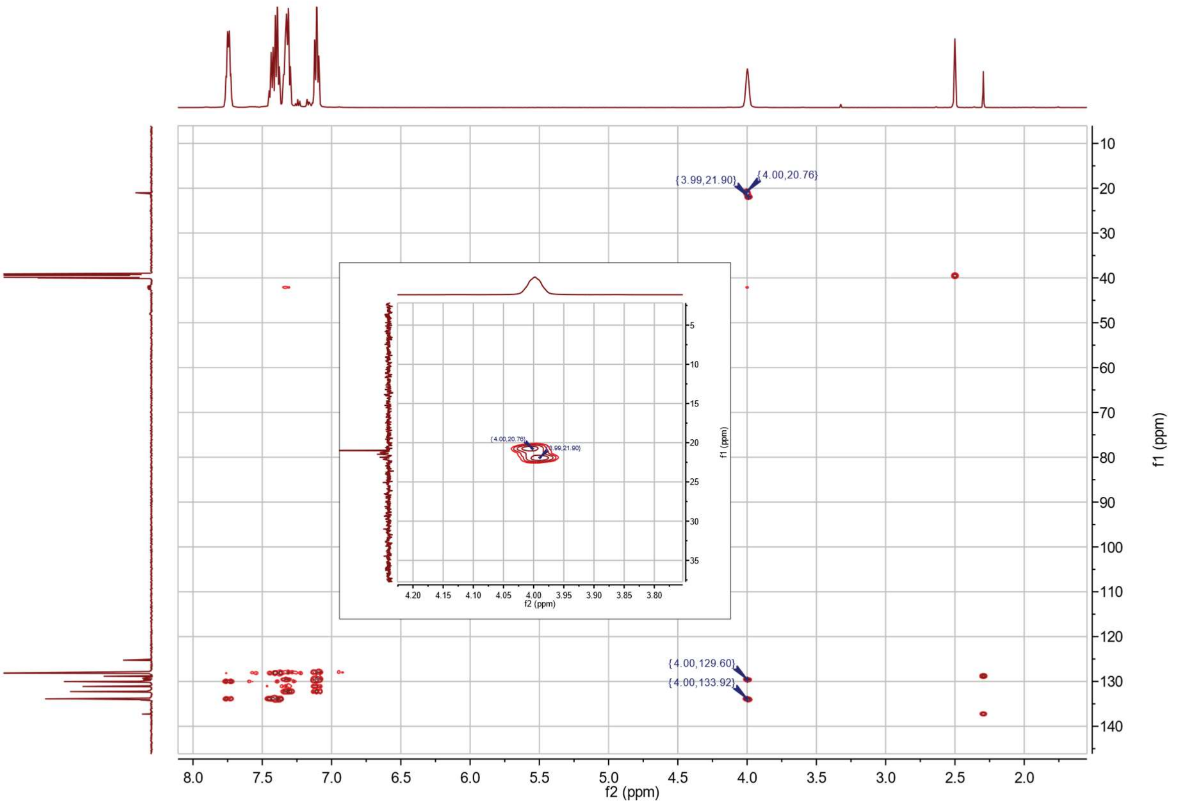

Figure S19 $^{1}H^{13}C$ HMBC NMR spectrum (499.72, 125.66 MHz, dimethylsulfoxide-d_6, 300 K) of [({dppm}$_2$C)RhCl] (**1**).

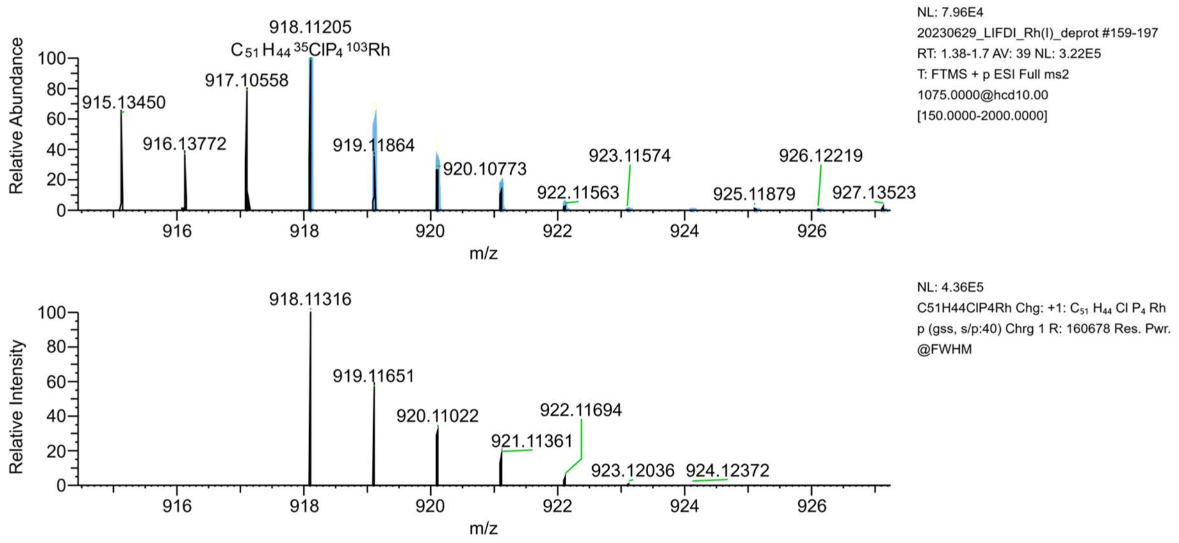

Peak Mass	Display F...	S Fit	RDB	Delta [pp...	Theo. ma...	Rank	Combine...	# Match...	# Missed...	MS Cov....	Pattern C...	MSMS M.
918.1120 5	$C_{51}H_{44}{}^{35}C$ $lP_4{}^{103}Rh$	13.47981 5046108 4	32.50	-1.21	918.1131 6	1	58.77	6	0	61.29	54.77	(Collecti on)

Figure S20 Top: Section of the LIFDI HRMS spectrum (positive mode) of M^{+} [({dppm}$_2$C)RhCl] (**1**); Bottom: simulated isotope pattern M^{+} = [**1**]$^{+}$.

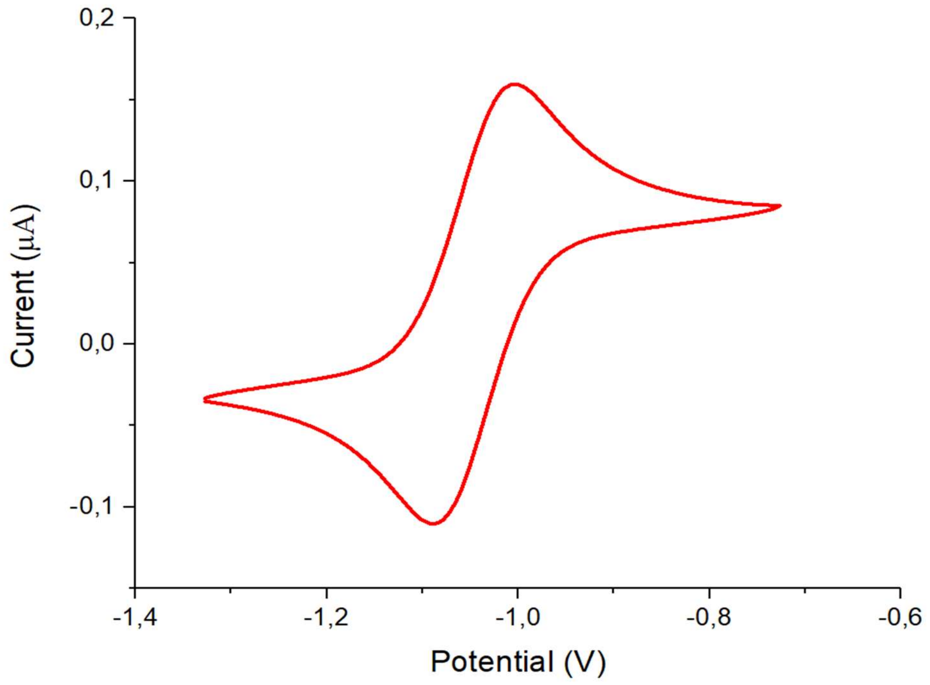

Figure S21 Cyclic voltammogram of [({dppm}$_2$C)RhCl] (**1**) (2mM sample concentration in 0.1 M TBAPF$_6$ THF solution; 100 mV/s scan rate, $E_{1/2}$ = -1.07 V vs Fc/Fc$^+$).

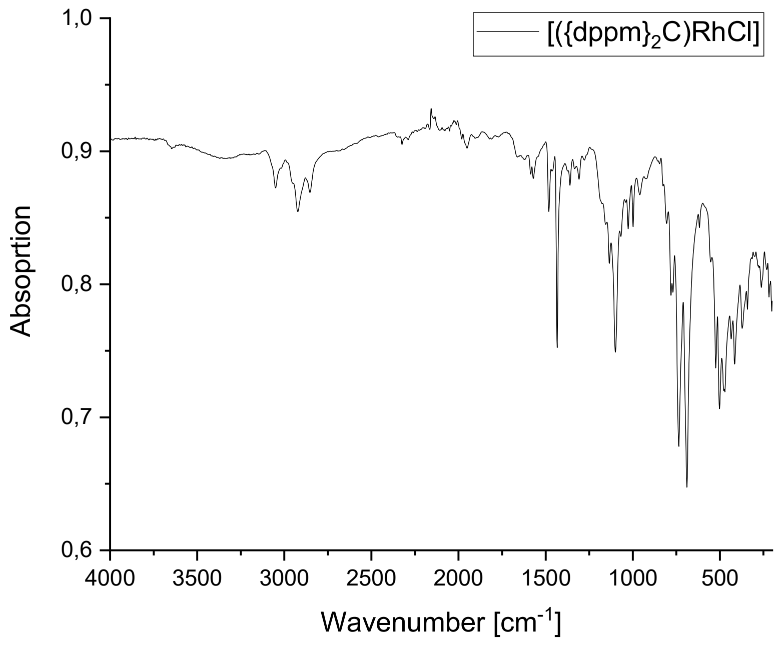

Figure S22 IR (ATR) spectrum of [({dppm}$_2$C)RhCl] (**1**).

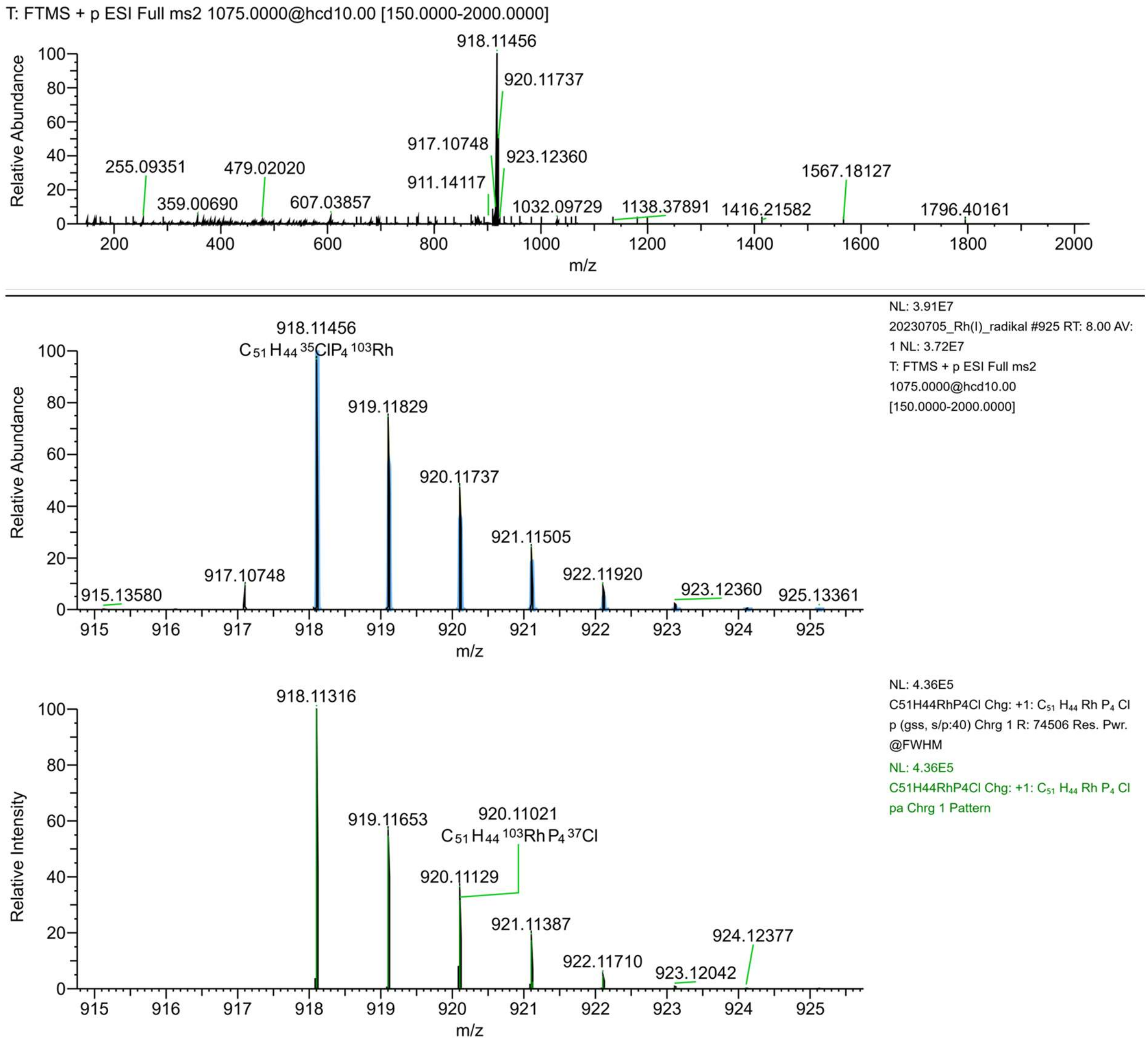

Peak Mass	Display F...	S Fit	RDB	Delta [pp...	Theo. ma...	Rank	Combine...	# Match...	# Missed...	MS Cov....	Pattern C...	MSMS M..
918.11456	$C_{51}H_{44}{}^{35}ClP_4{}^{103}Rh$	11.5184525568128	32.50	1.53	918.11316	1	45.5	5	2	47.39	55.02	(Collection)

Figure S23 Top: Full LIFDI HRMS spectrum (positive mode) of [({dppm}$_2$C)RhCl]OTf (**2**); Middle: relevant section of molecule ion peak; Bottom: simulated isotope pattern of molecule ion peak M^+ = [**2**-OTf]$^+$.

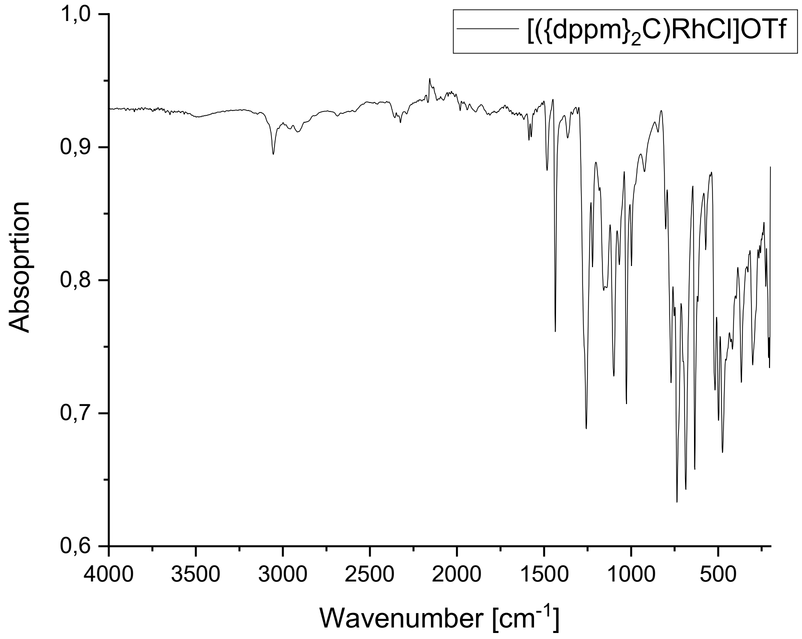

Figure S24 IR (ATR) spectrum of [({dppm}$_2$C)RhCl]OTf (**2**).

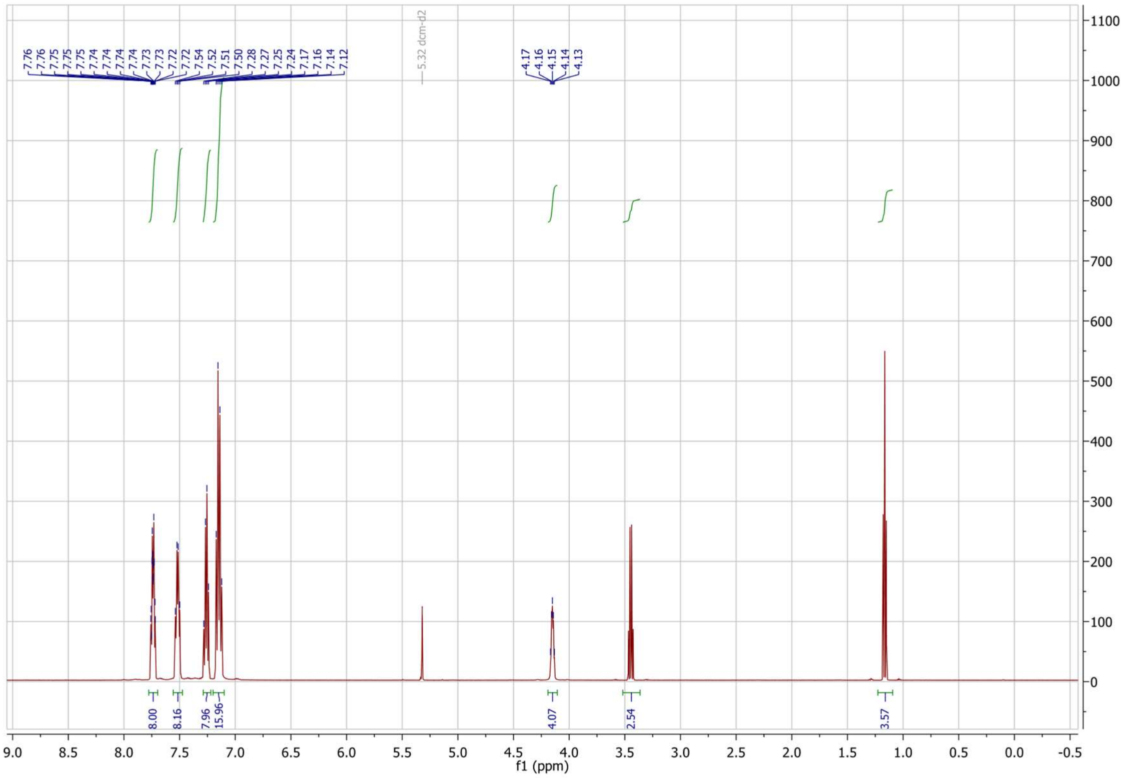

Figure S25 ^{1}H NMR spectrum (499.72 MHz, dichlormethane-d_2, 300 K) of [({dppm}$_2$C)RhCl$_3$] (**3**) x 1.5 Et_2O crystallized with 1.5 equiv. diethylether in the crystal lattice).

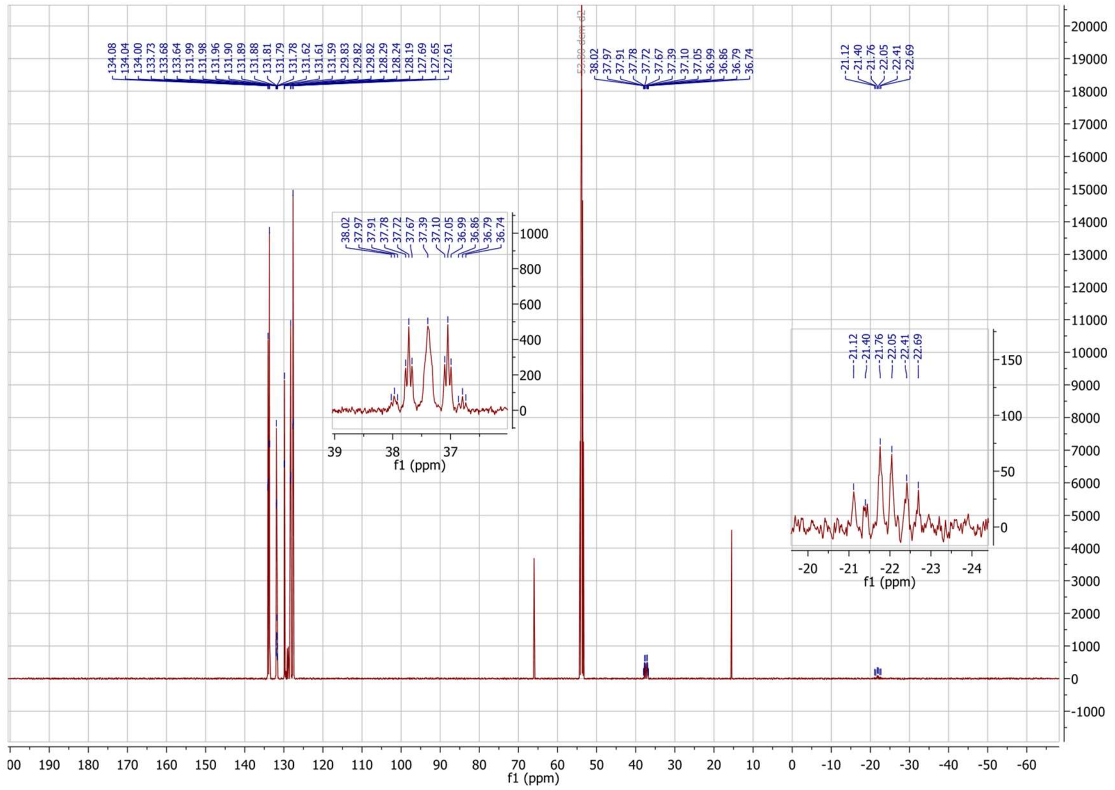

Figure S26 $^{13}C\{^{1}H\}$ NMR spectrum (125.66 MHz, dichlormethane-d_2, 300 K) of [({dppm}$_2$C)RhCl$_3$] (**3**) x 1.5 Et_2O crystallized with 1.5 equiv. diethylether in the crystal lattice).

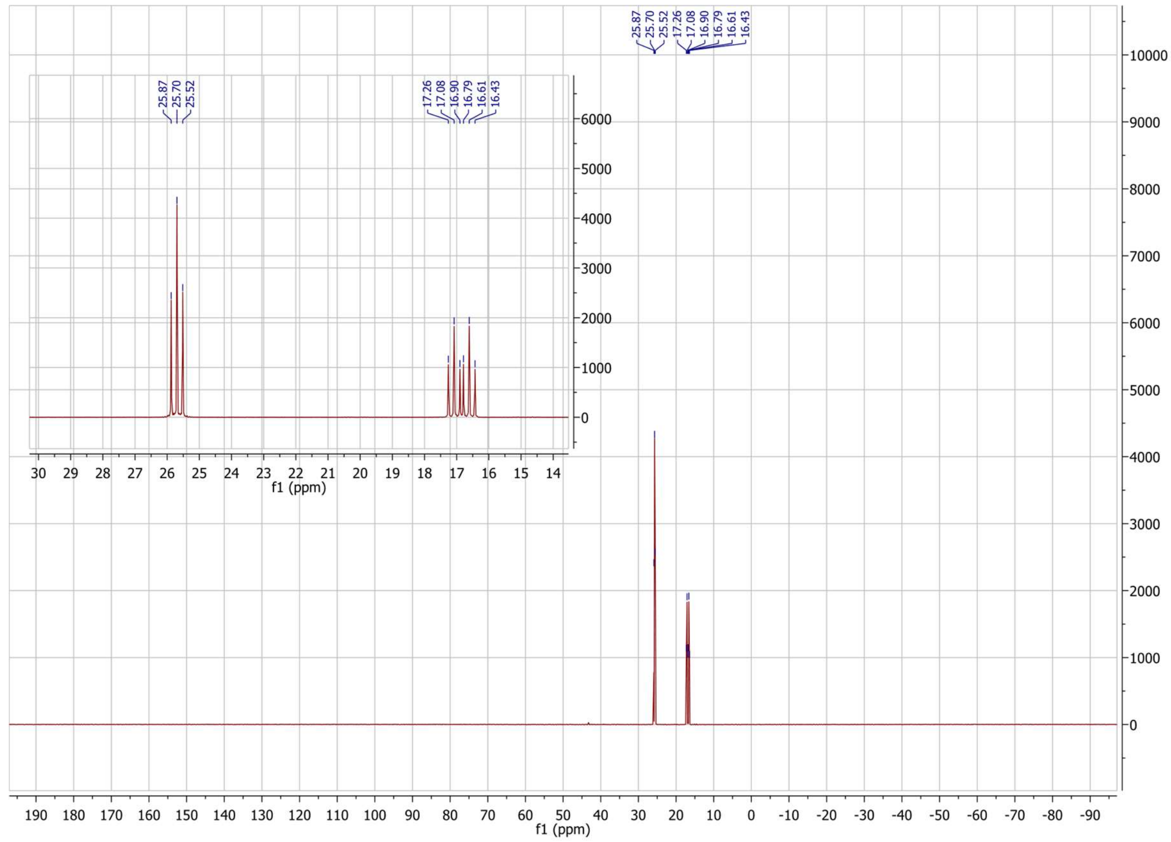

Figure S27 $^{31}P\{^{1}H\}$ NMR spectrum (202.30 MHz, dichlormethane-d_2, 300 K) of [({dppm}$_2$C)RhCl$_3$] (**3**).

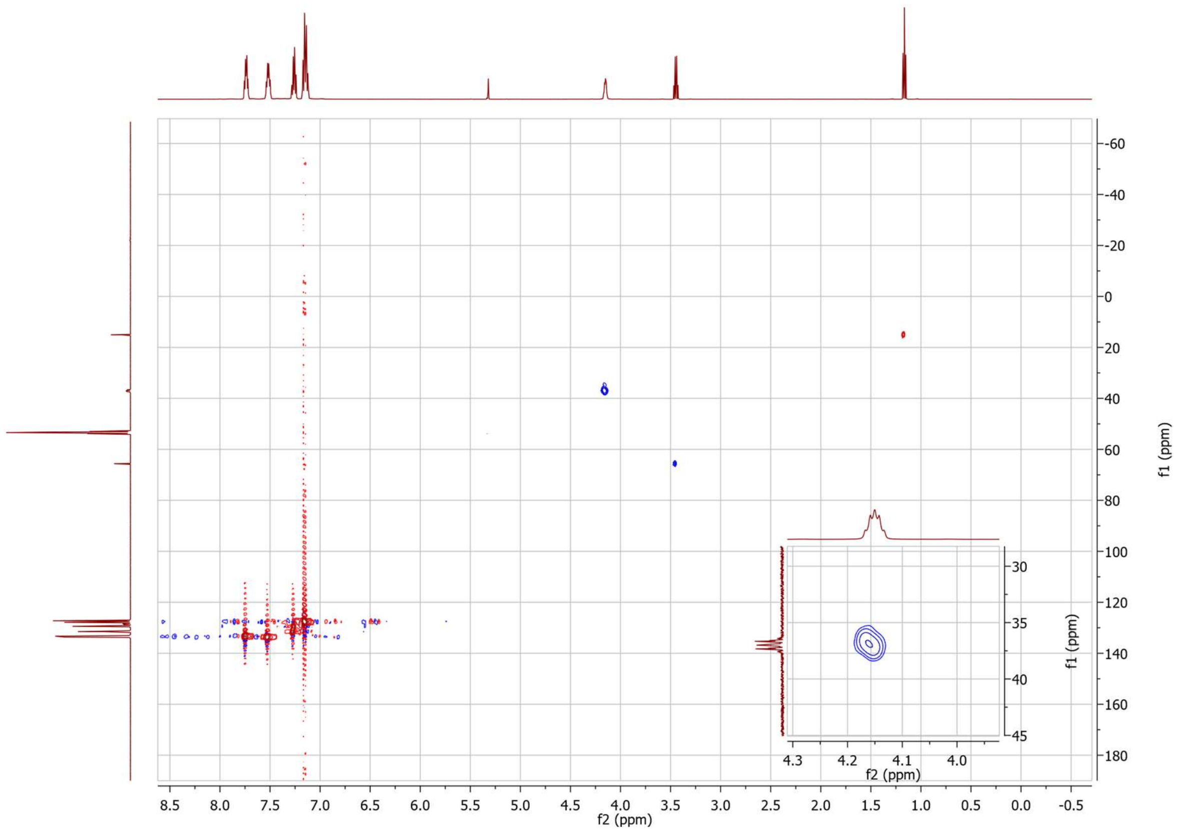

Figure S28 $^{1}H^{13}C$ HSQC NMR spectrum (499.72, 125.66 MHz, dichlormethane-d_2, 300 K) of [({dppm}$_2$C)RhCl$_3$] (**3**).

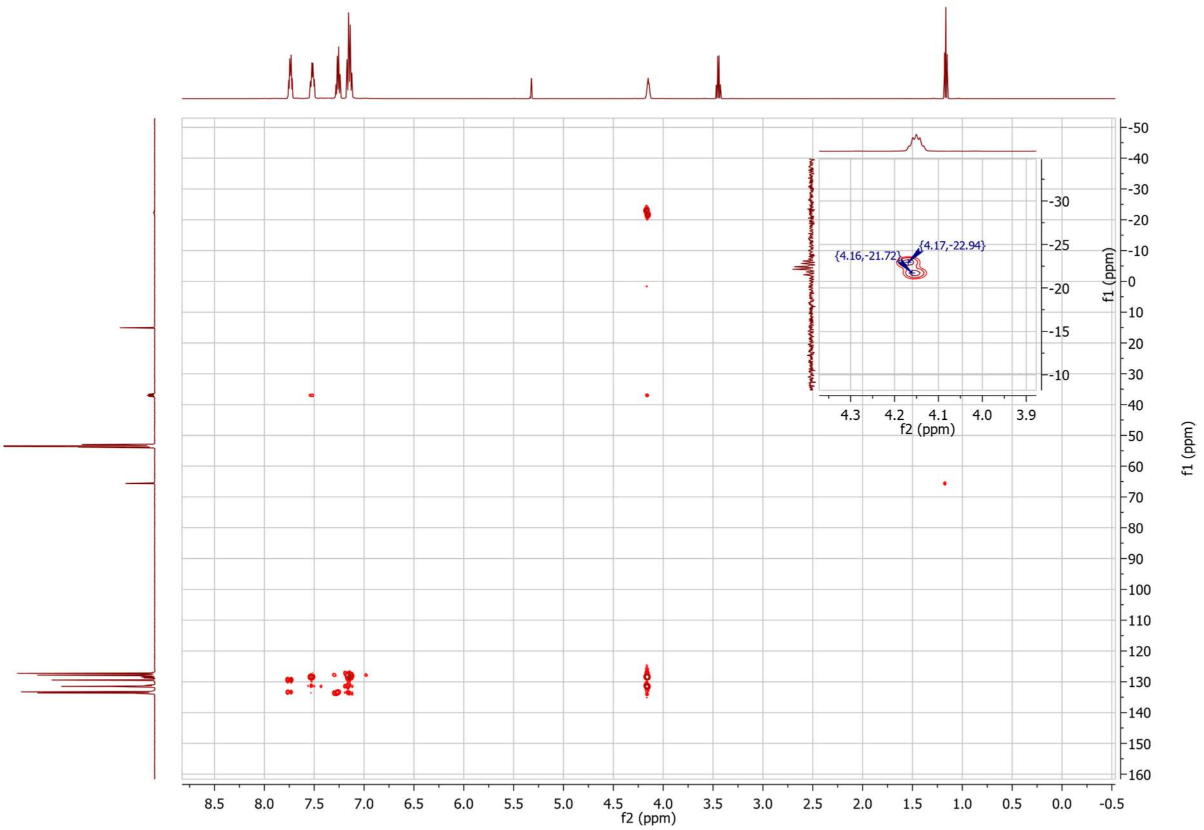

Figure S29 $^{1}H^{13}C$ HMBC NMR spectrum (499.72, 125.66 MHz, dichlormethane-d_2, 300 K) of [({dppm}$_2$C)RhCl$_3$] (**3**).

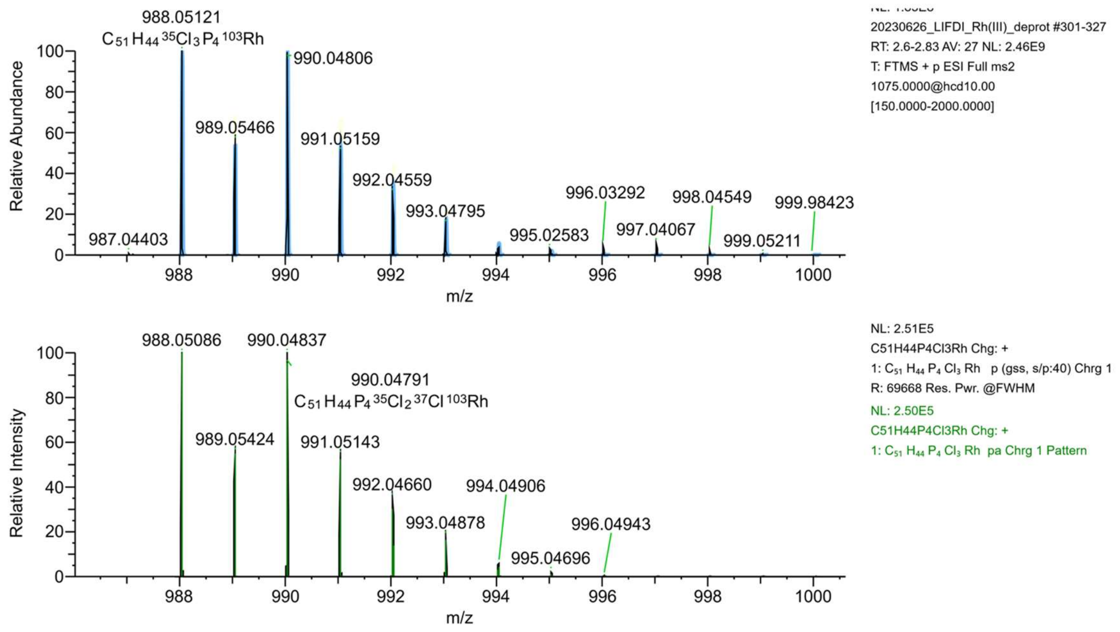

Peak Mass	Display F...	S Fit	RDB	Delta [pp...	Theo. ma...	Rank	Combine...	# Match...	# Missed...	MS Cov....	Pattern C...	MSMS M.
988.05121	$C_{51}H_{44}{}^{35}Cl_3P_4{}^{103}Rh$	39.136359105431 6	31.50	0.35	988.05086	1	92.82	8	1	95.81	97.66	(Collection)

Figure S30 Top: Section of the LIFDI HRMS spectrum (positive mode) of M$^+$ [({dppm}$_2$C)RhCl$_3$] (**3**); Bottom: simulated isotope pattern of M$^+$ = [**3**]$^+$.

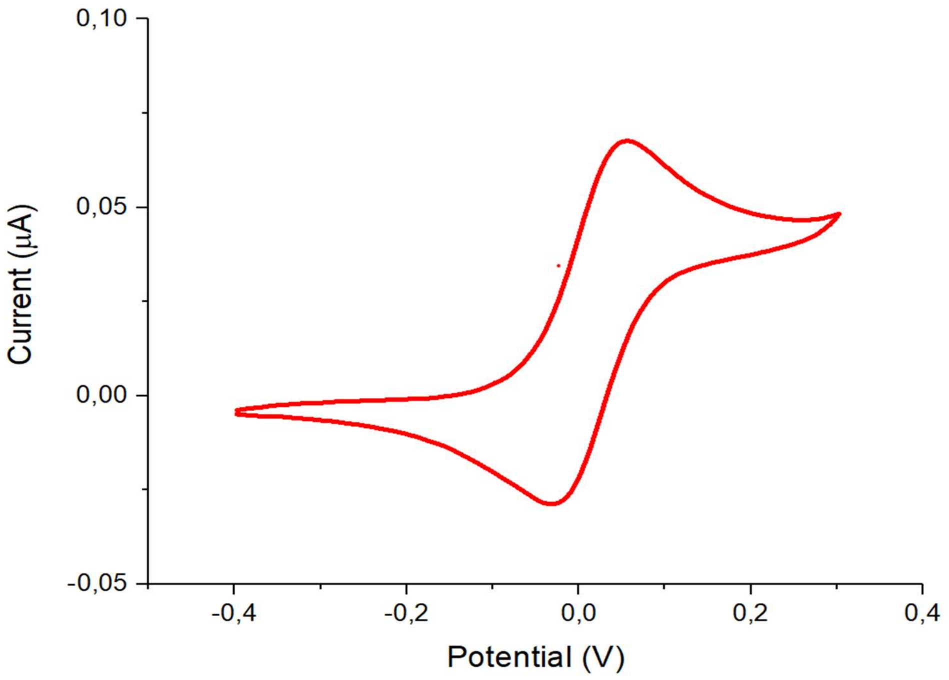

Figure S31 Cyclic voltammogram of [({dppm}$_2$C)RhCl$_3$] (**3**) (2mM sample concentration in 0.1 M TBAPF$_6$ dichlormethane solution; 100 mV/s scan rate, $E_{1/2}$ = +0.05 V vs Fc/Fc$^+$).

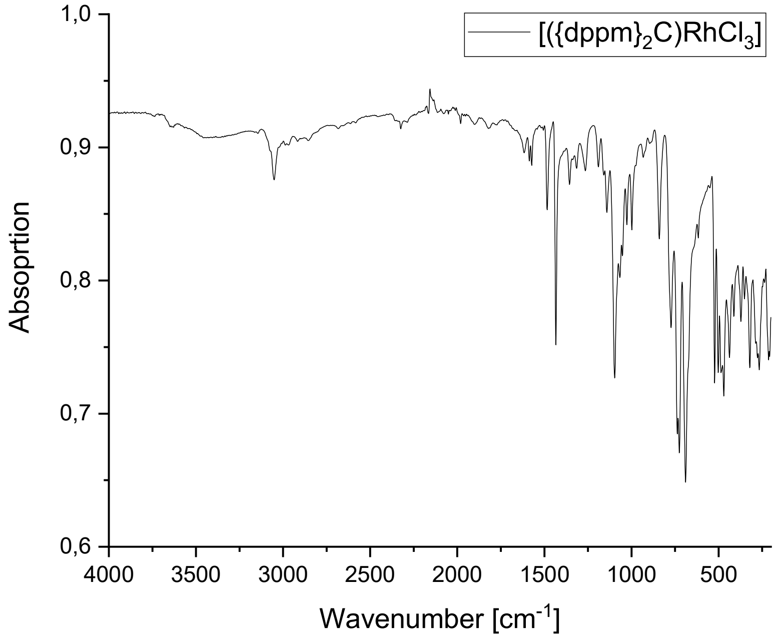

Figure S32 IR (ATR) spectrum of [({dppm}$_2$C)RhCl$_3$] (**3**).

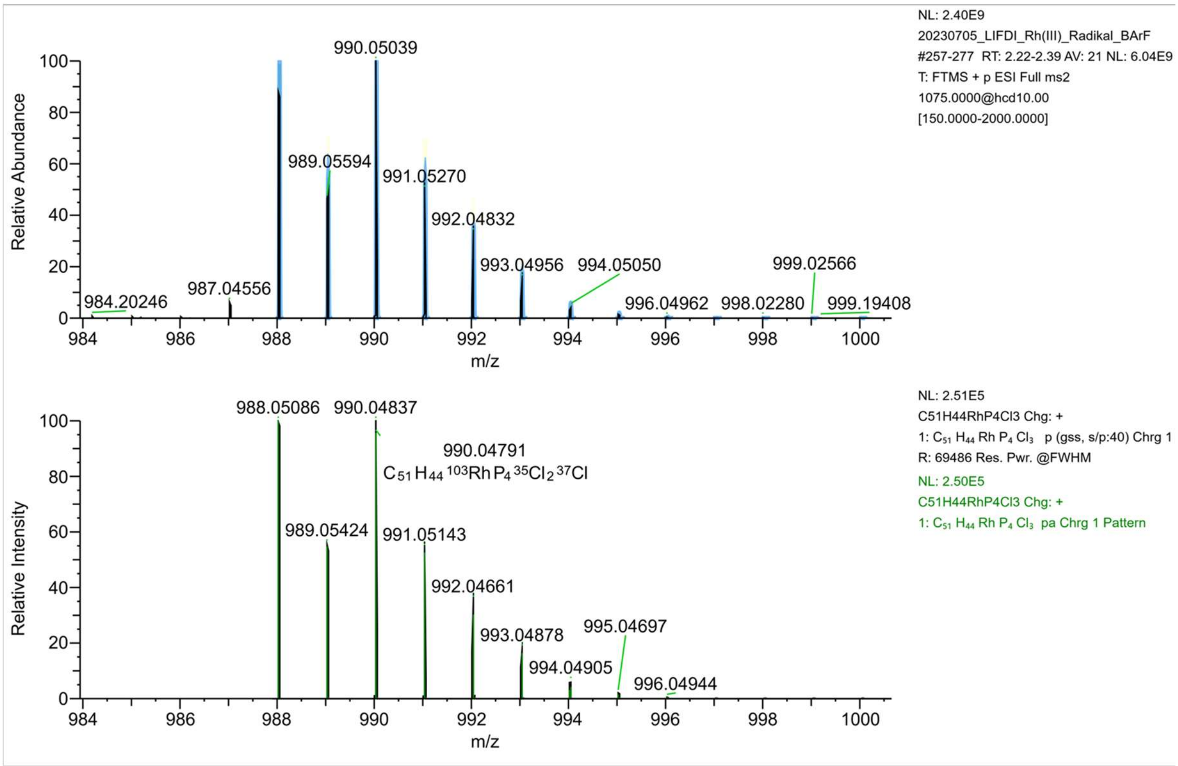

Peak Mass	Display F...	S Fit	RDB	Delta [m...	Theo. ma...	Rank	Combine...	# Match...	# Missed...	MS Cov....	Pattern C...	MSMS M.
988.0523 4	$C_{51}H_{44}{}^{35}Cl_3P_4{}^{103}Rh$	49.13085 5438866 5	31.50	1.48	988.0508 6	1	96.12	9	0	98.73	98.37	(Collection)

Figure S33 Top: Section of the LIFDI HRMS spectrum (positive mode) of [({dppm}$_2$C)RhCl$_3$]OTf (**4**); Bottom: simulated isotope pattern of M$^+$ = [**4**-OTf]$^+$.

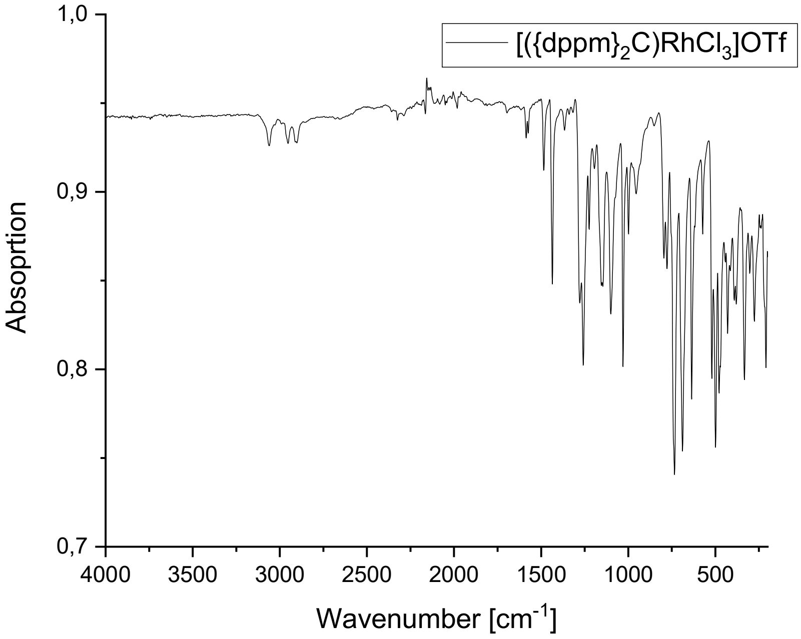

Figure S34 IR (ATR) spectrum of [({dppm}$_2$C)RhCl$_3$]OTf (**4**).

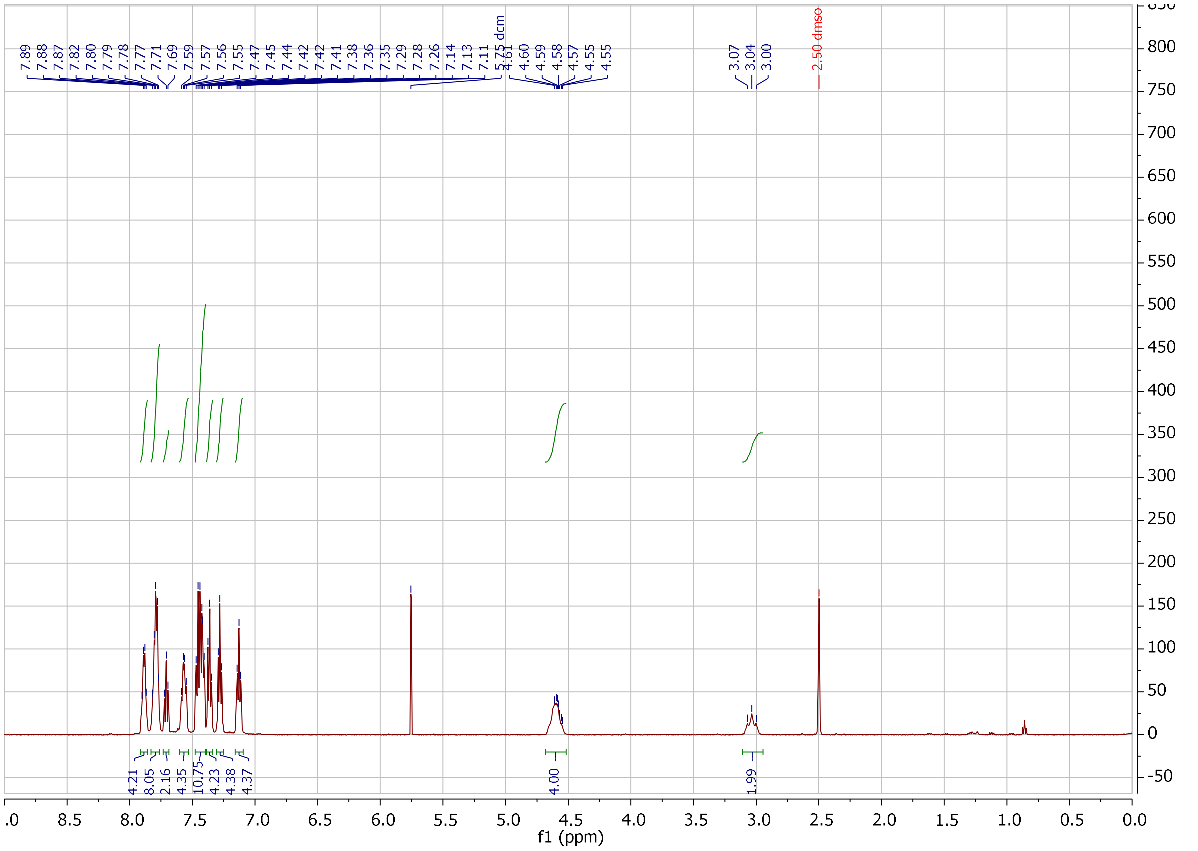

Figure S35 ^{1}H NMR spectrum (502.23 MHz, dimethylsulfoxide-d$_6$, 300 K) of [({dppm}$_2$C-CH$_2$)RhCl$_2$]Cl (**5a**).

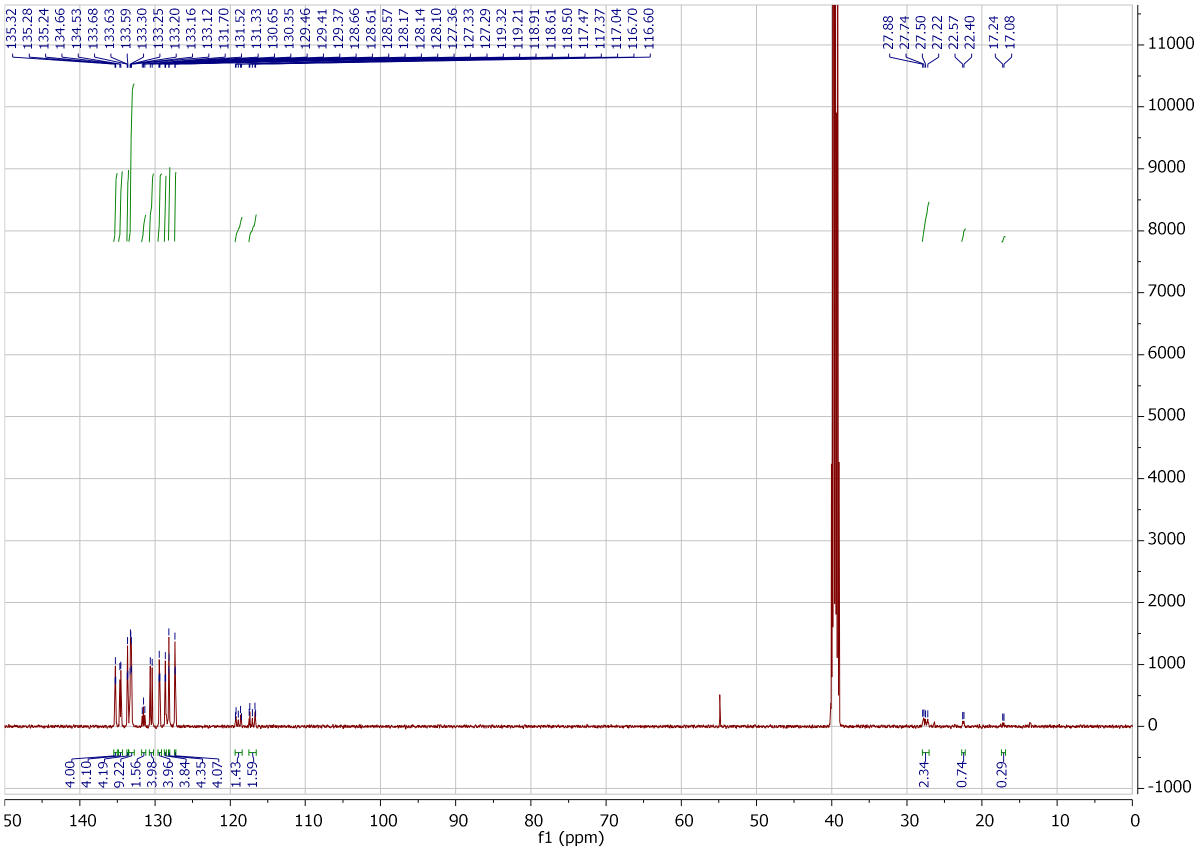

Figure S36 $^{13}C\{^{1}H\}$ NMR spectrum (125.66 MHz, dimethylsulfoxide-d$_6$, 300 K) of [({dppm}$_2$C-CH$_2$)RhCl$_2$]Cl (**5a**).

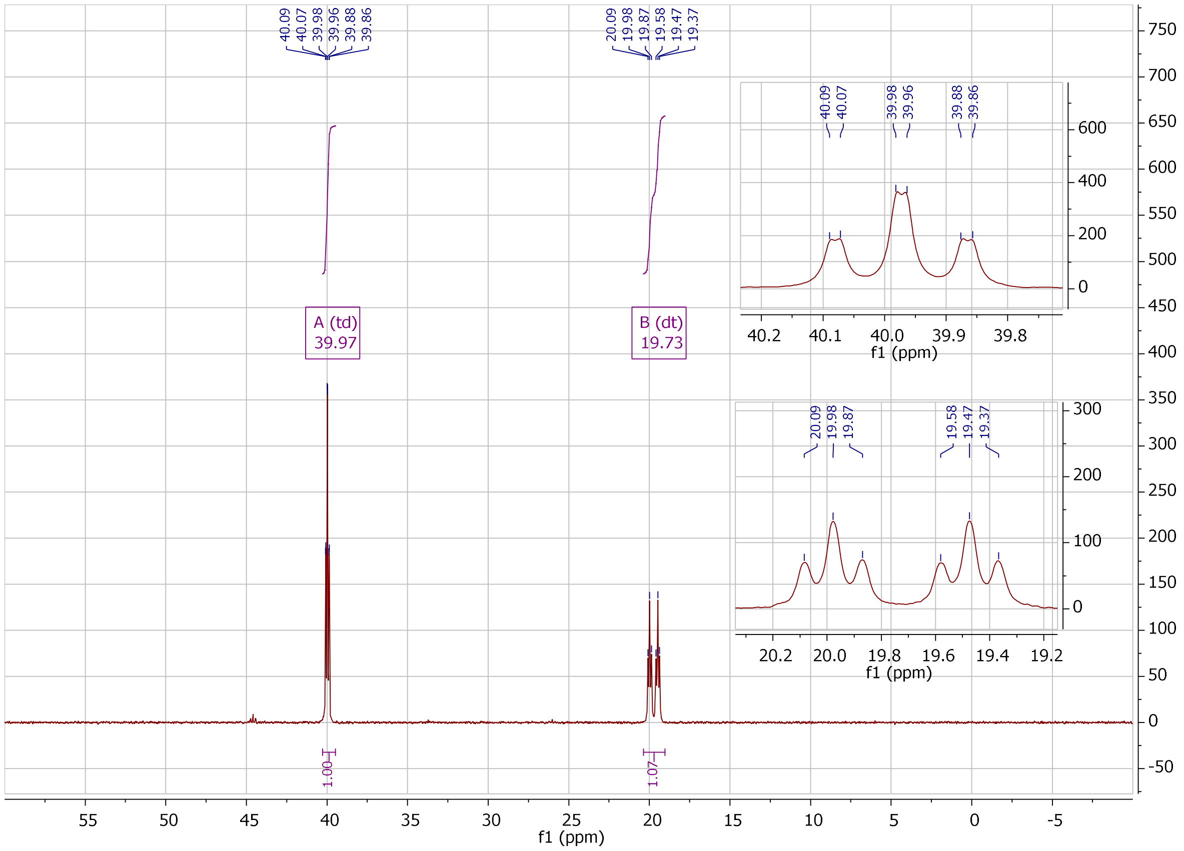

Figure S37 $^{31}P\{^{1}H\}$ NMR spectrum (220.30 MHz, dimethylsulfoxide-d_6, 300 K) of [({dppm}$_2$C-CH_2)$RhCl_2$]Cl (**5a**).

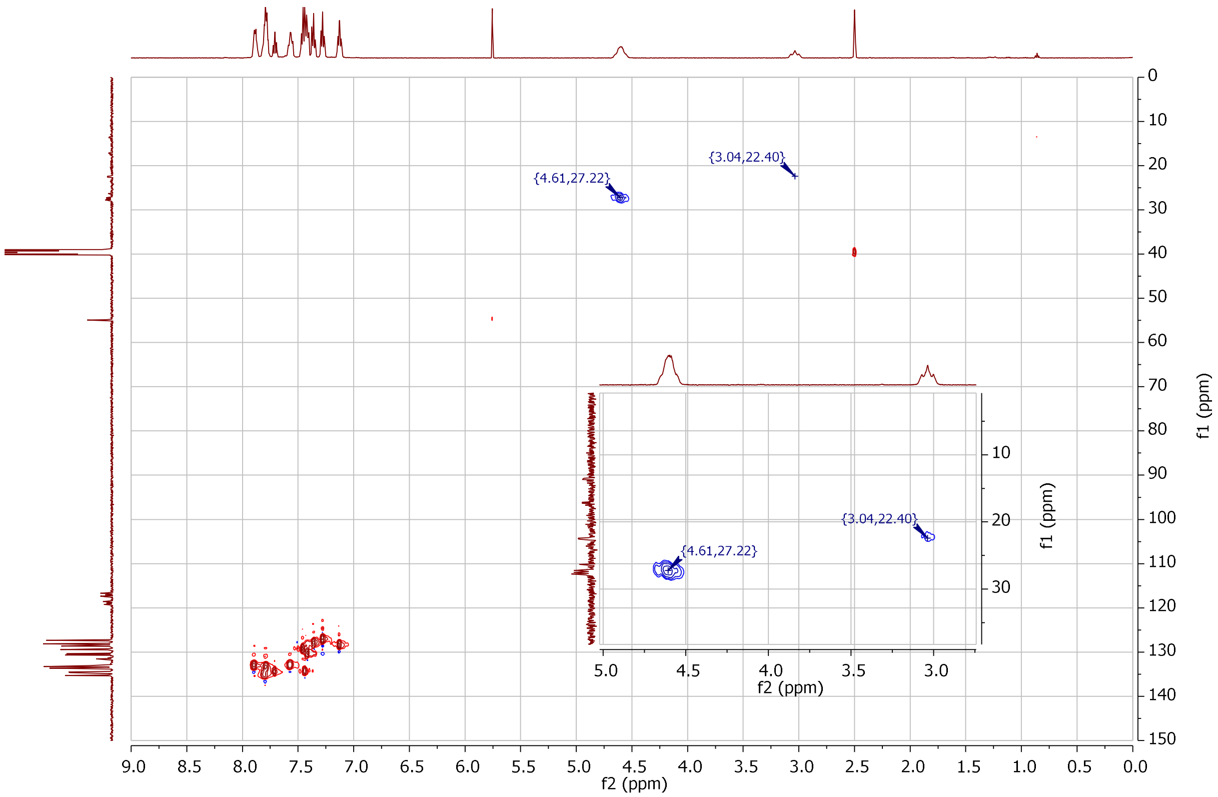

Figure S38 $^{1}H^{13}C$ HSQC NMR spectrum (502.23, 125.66 MHz, dimethylsulfoxide-d_6, 300 K) of [({dppm}$_2$C-CH_2)$RhCl_2$]Cl (**5a**).

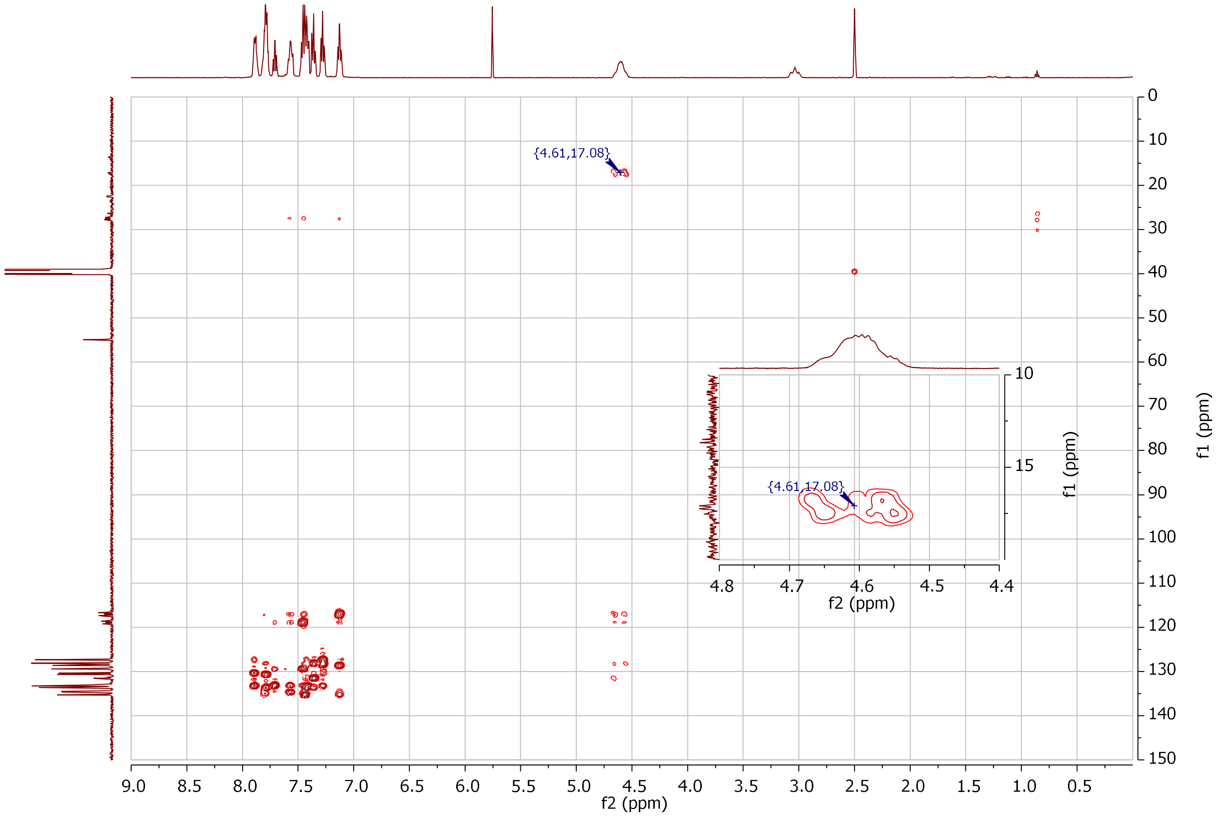

Figure S39 $^{1}H^{13}C$ HMBC NMR spectrum (502.23, 125.66 MHz, dimethylsulfoxide-d_6, 300 K) of [({dppm}$_2$C-CH$_2$)RhCl$_2$]Cl (**5a**).

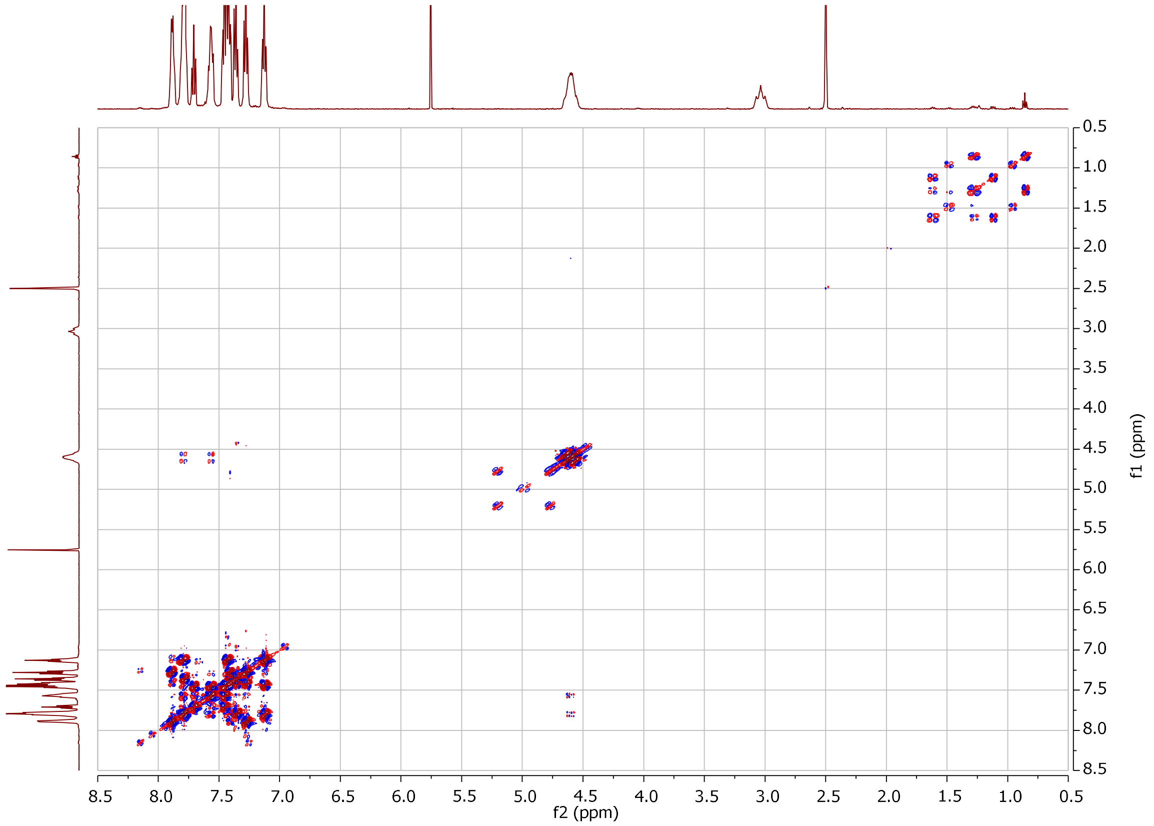

Figure S40 $^{1}H^{1}H$ COSY NMR spectrum (502.23 MHz, dimethylsulfoxide-d_6, 300 K) of [({dppm}$_2$C-CH$_2$)RhCl$_2$]Cl (**5a**).

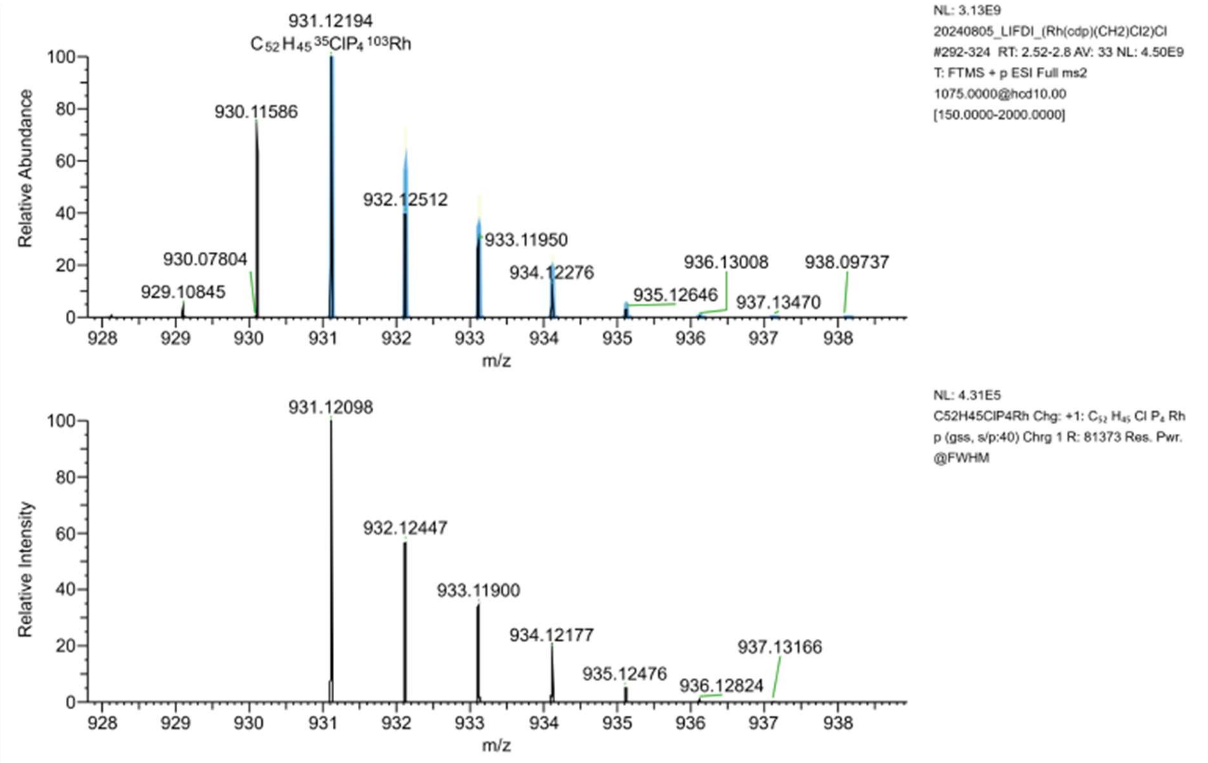

Peak Mass	Display F...	S Fit	RDB	Delta [pp...	Theo. ma...	Rank	Combine...	# Match...	# Missed...	MS Cov....	Pattern C...	MSMS M...
931.12194	$C_{52}H_{45}{}^{35}ClP_4{}^{103}Rh$	18.289342374985 1	33.00	1.03	931.12098	1	87.51	7	0	91.36	88.54	(Collection)

Figure S41 Top: Section of the LIFDI HRMS spectrum (positive mode) of [({dppm}$_2$C-CH$_2$)RhCl$_2$]Cl (**5a**) – Cl; Bottom: simulated isotope pattern of M+ -HCl = [**5a**–HCl]+.

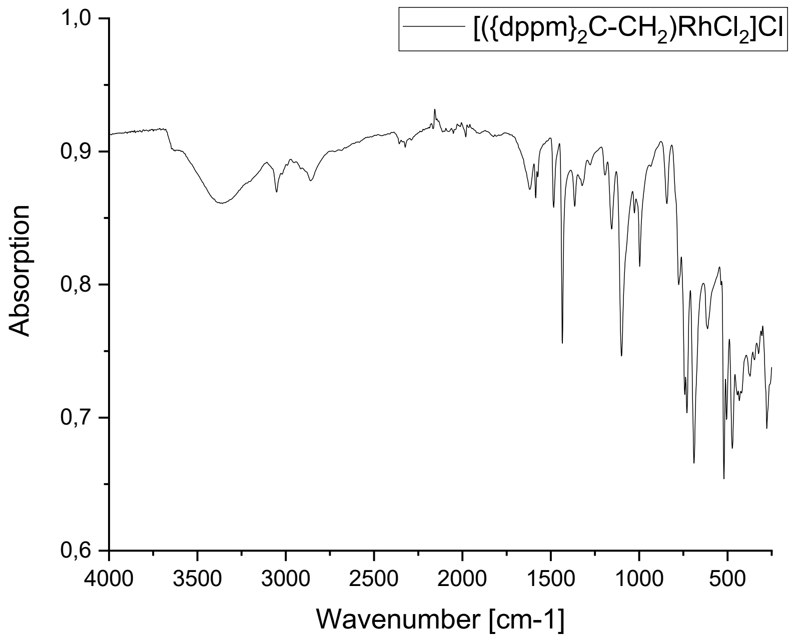

Figure S42 IR (ATR) spectrum of [({dppm}$_2$C-CH$_2$)RhCl$_2$]Cl (**5a**).

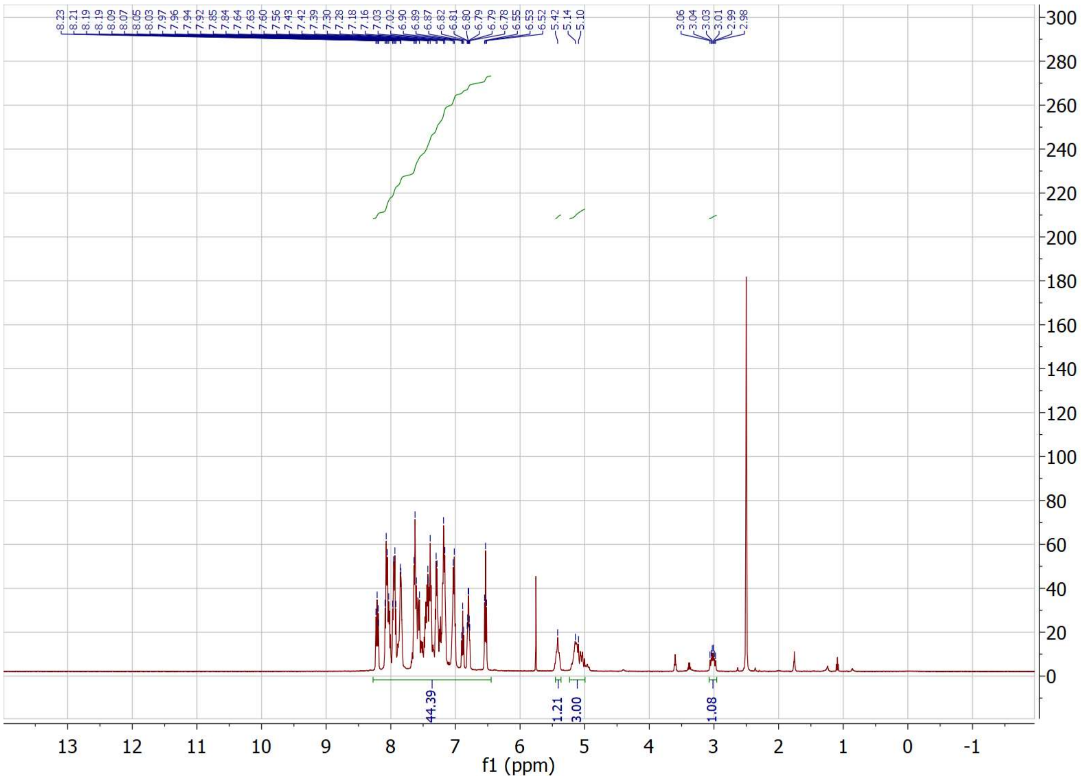

Figure

Figure S43 ^{1}H NMR spectrum (502.23 MHz, dimethylsulfoxide-d_6, 300 K) of [({dppm}$_2$C-CHPh)RhCl$_2$]Cl (**5b-Cl**).

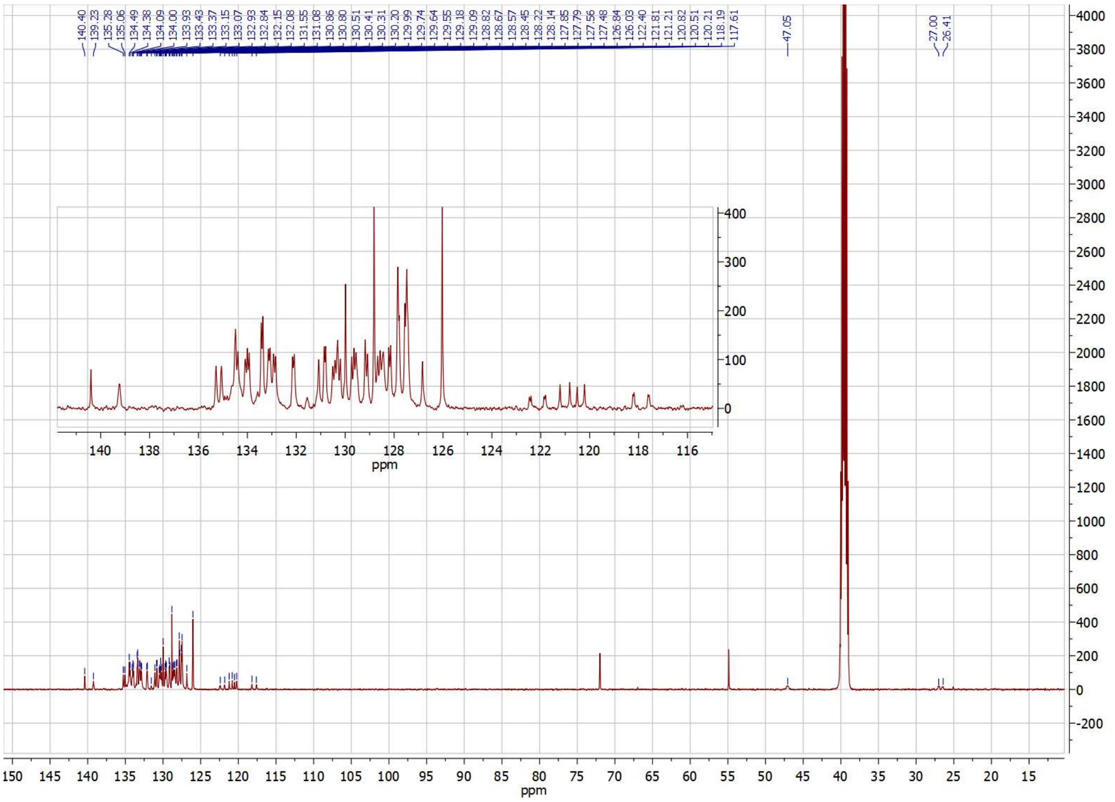

Figure S44 $^{13}C\{^{1}H\}$ NMR spectrum (125.66 MHz, dimethylsulfoxide-d_6, 300 K) of [({dppm}$_2$C-CHPh)RhCl$_2$]Cl (**5b-Cl**).

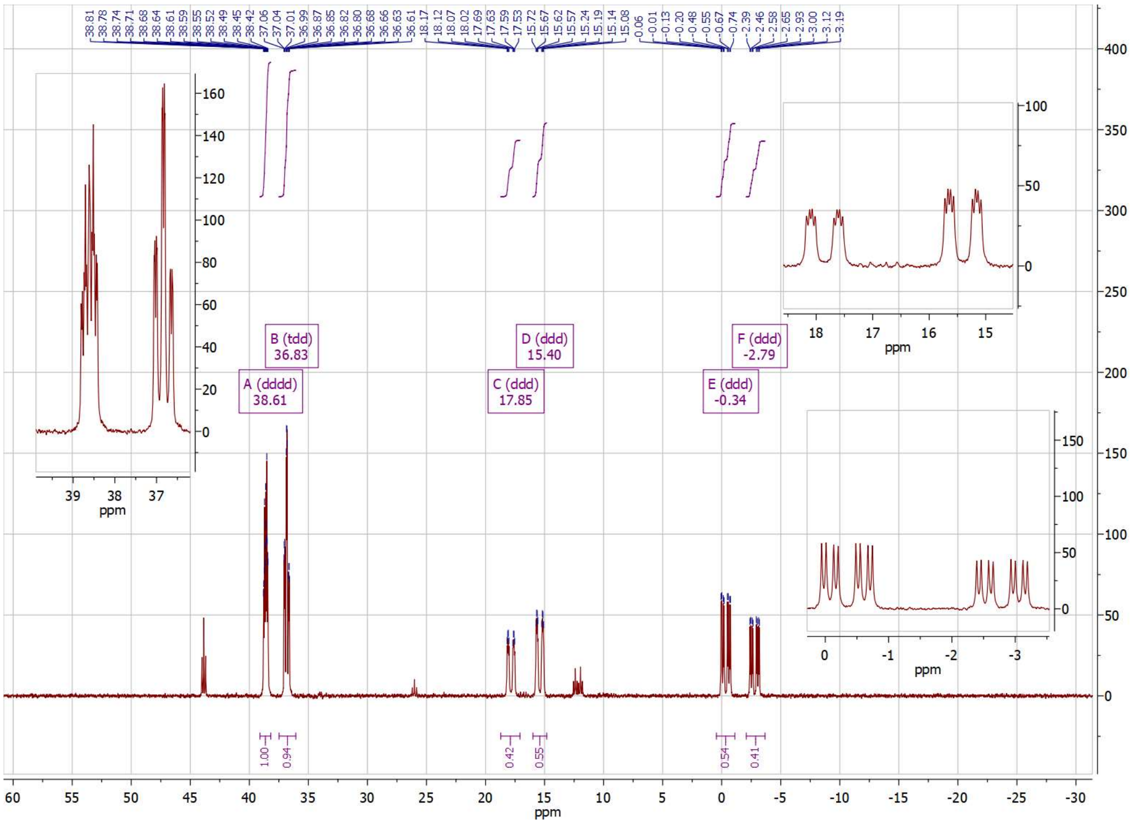

Figure S45 $^{31}P\{^1H\}$ NMR spectrum (202.30 MHz, dimethylsulfoxide-d$_6$, 300 K) of [({dppm}$_2$C-CHPh)RhCl$_2$]Cl (**5b-Cl**).

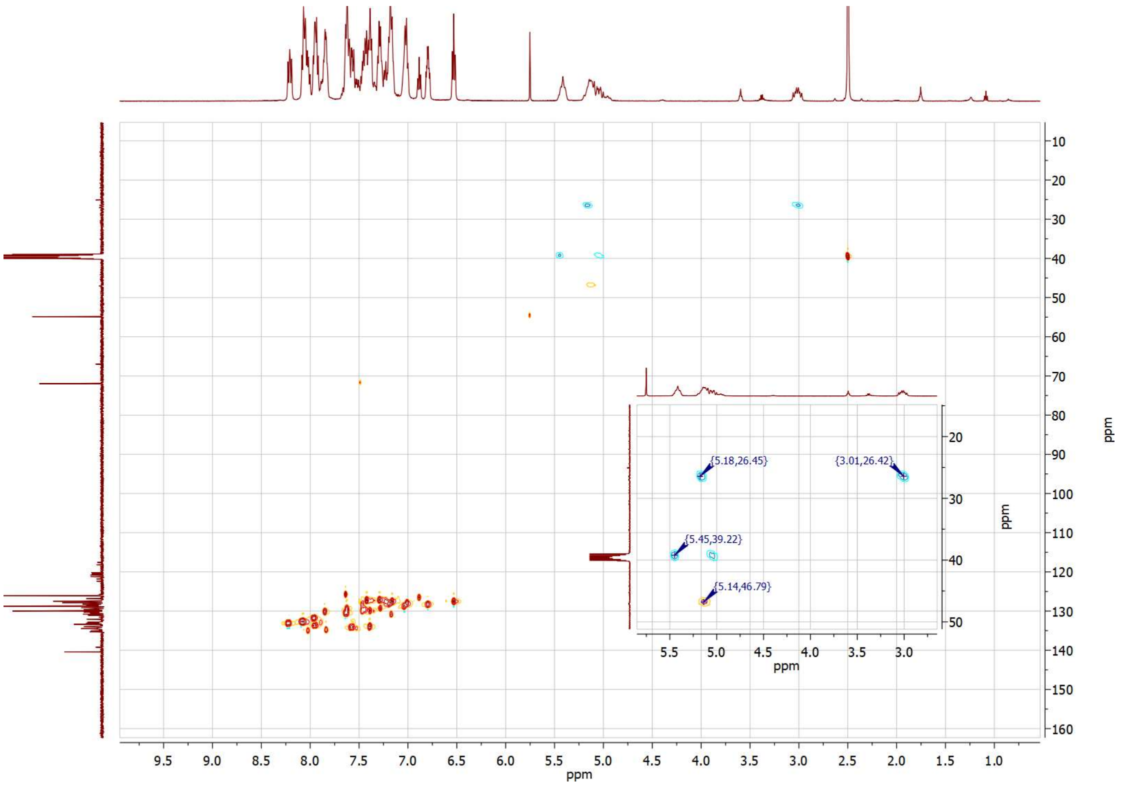

Figure S46 $^1H^{13}C$ HSQC NMR spectrum (502.29, 125.66 MHz, dimethylsulfoxide-d$_6$, 300 K) of [({dppm}$_2$C-CHPh)RhCl$_2$]Cl (**5b-Cl**).

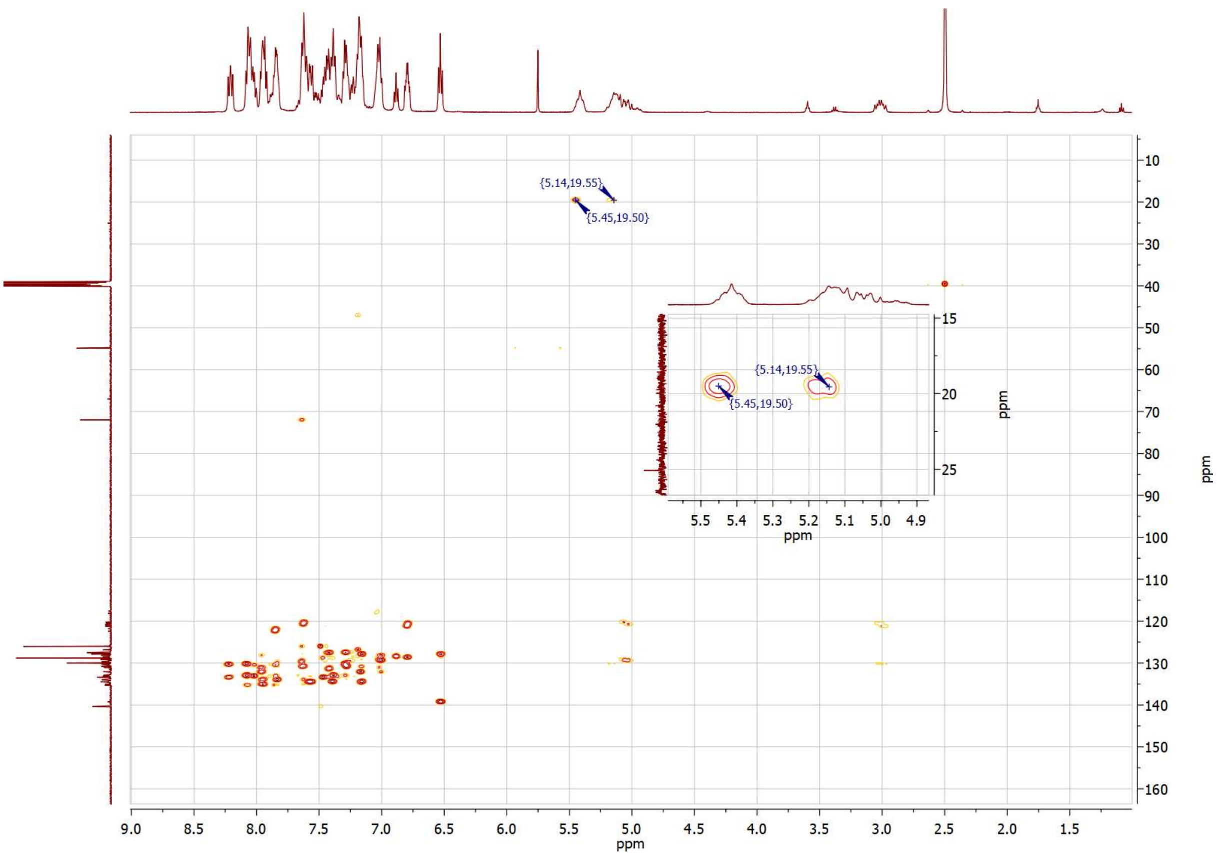

Figure S47 $^1H^{13}C$ HMBC NMR spectrum (502.29, 125.66 MHz, dimethylsulfoxide-d_6, 300 K) of [({dppm}$_2$C-CHPh)RhCl$_2$]Cl (**5b-Cl**).

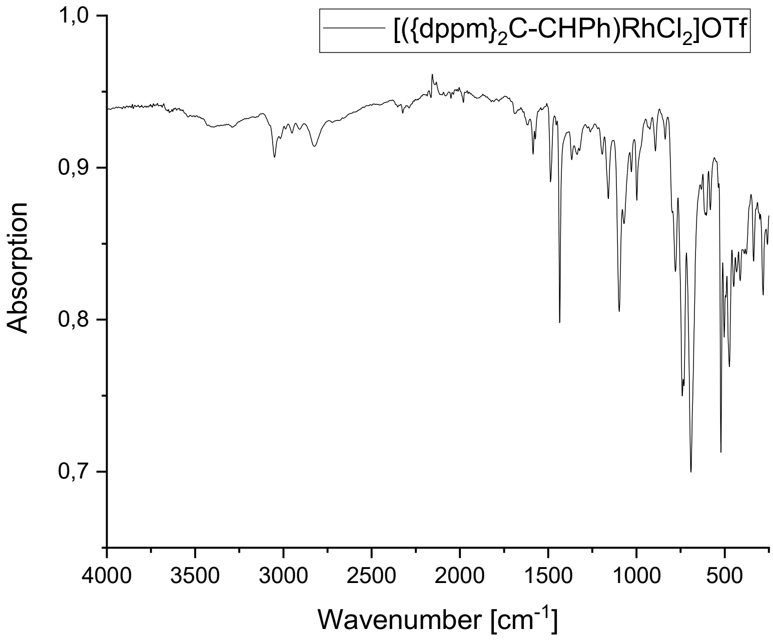

Figure S48 IR (ATR) spectrum of [({dppm}$_2$C-CHPh)RhCl$_2$]Cl (**5b-OTf**).

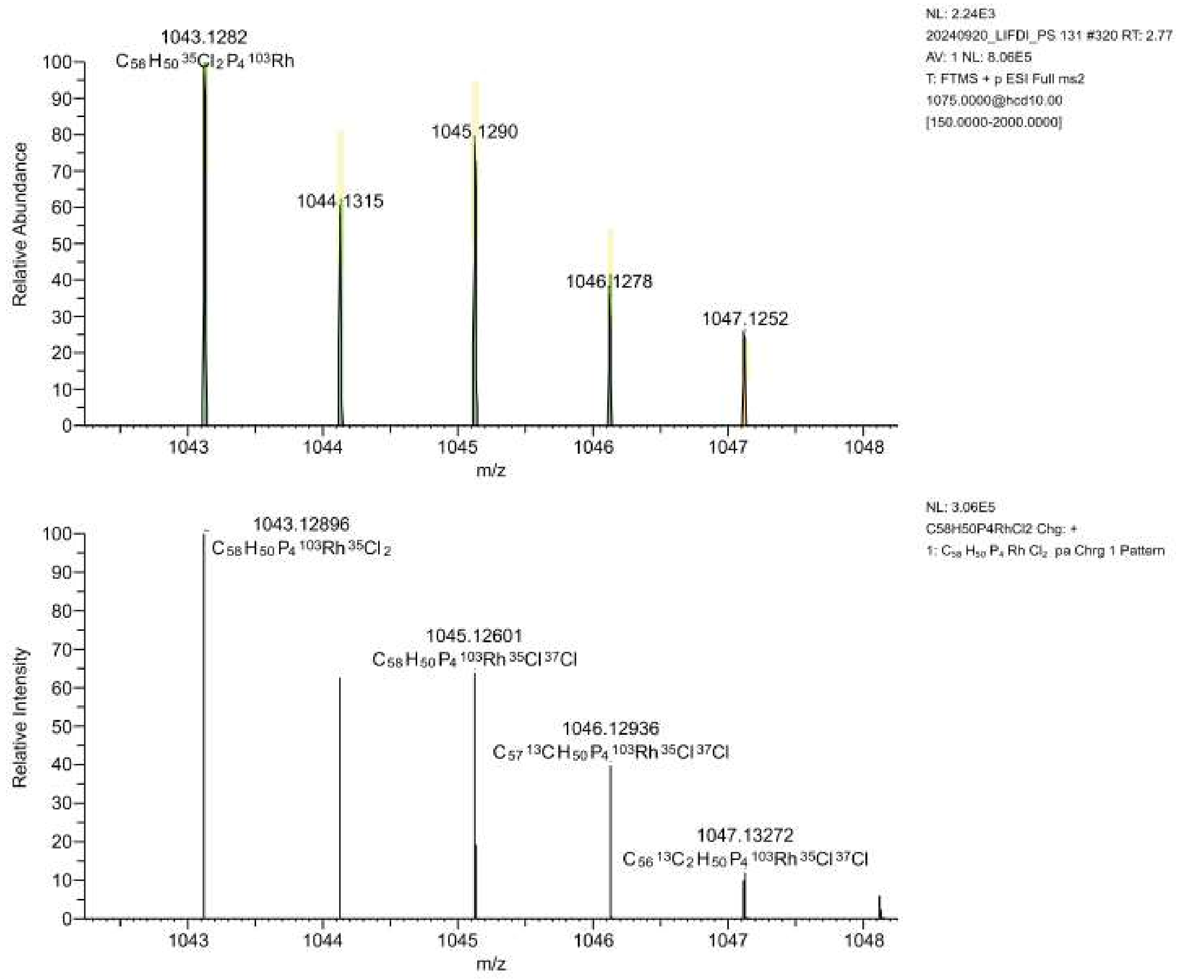

Peak Mass	Display...	S Fit	RDB	Delta [p...	Theo. m...	Rank	Combin...	# Match...	# Misse...	MS Cov....	Pattern...	MSMS...
1043.1282	$C_{58}H_{50}{}^{35}Cl_2P_4{}^{103}Rh$	49.315418697035 4	36.00	-0.75	1043.12896	1	89.18	5	0	91.4	93.82	(Collection)

Figure S49 Top: Section of the LIFDI HRMS spectrum (positive mode) of [({dppm}$_2$C-CHPh)RhCl$_2$]OTf (**5b**); Bottom: simulated isotope pattern of M+ = [**5b**]+.

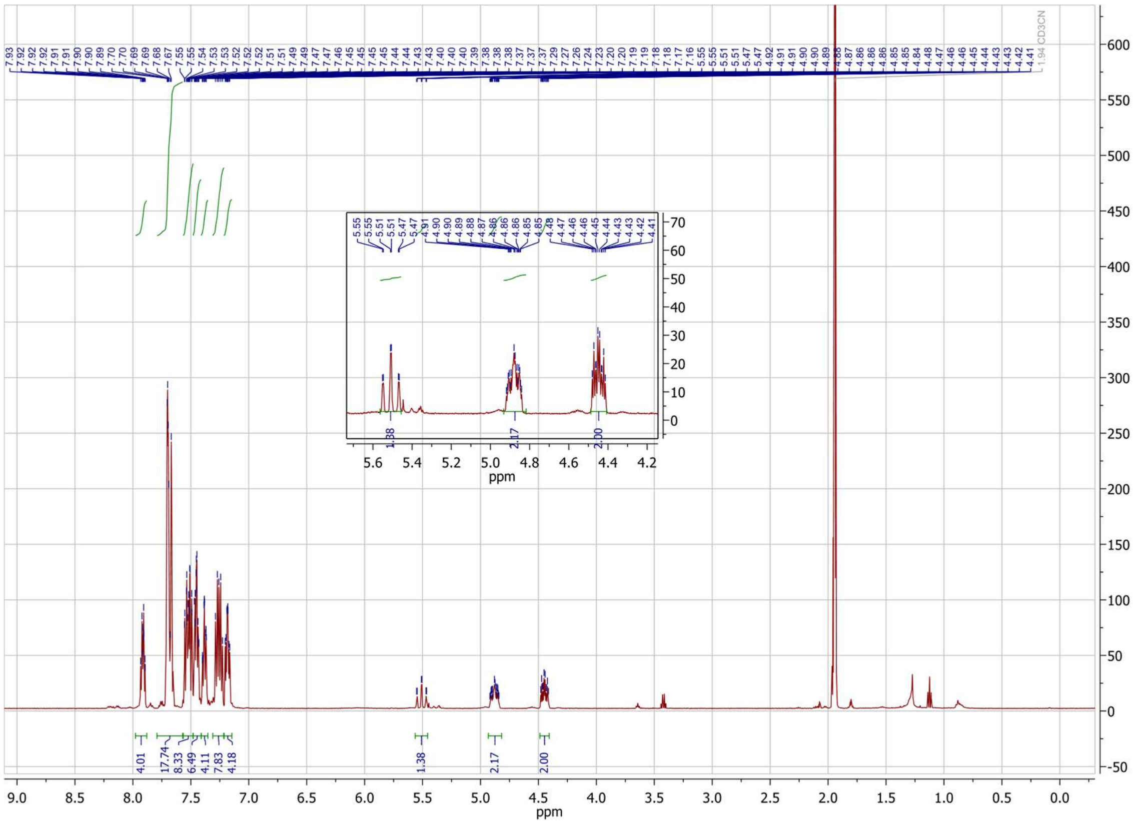

Figure S50 ^{1}H NMR spectrum (499.72 MHz, acetonitrile-d_3, 300 K) of [({dppm}$_2$CH)RhCl$_3$]BArF (**6-BarF**).

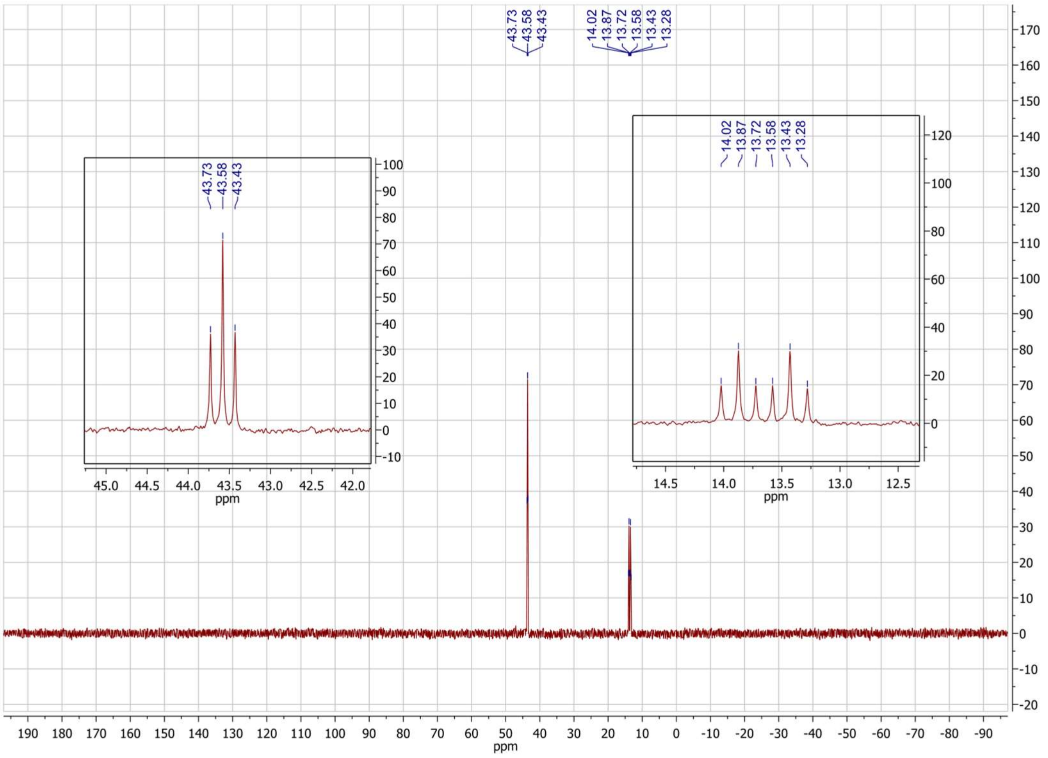

Figure S51 $^{31}P\{^{1}H\}$ NMR spectrum (202.30 MHz, acetonitrile-d_3, 300 K) of [({dppm}$_2$CH)RhCl$_3$]BArF (**6-BArF**).

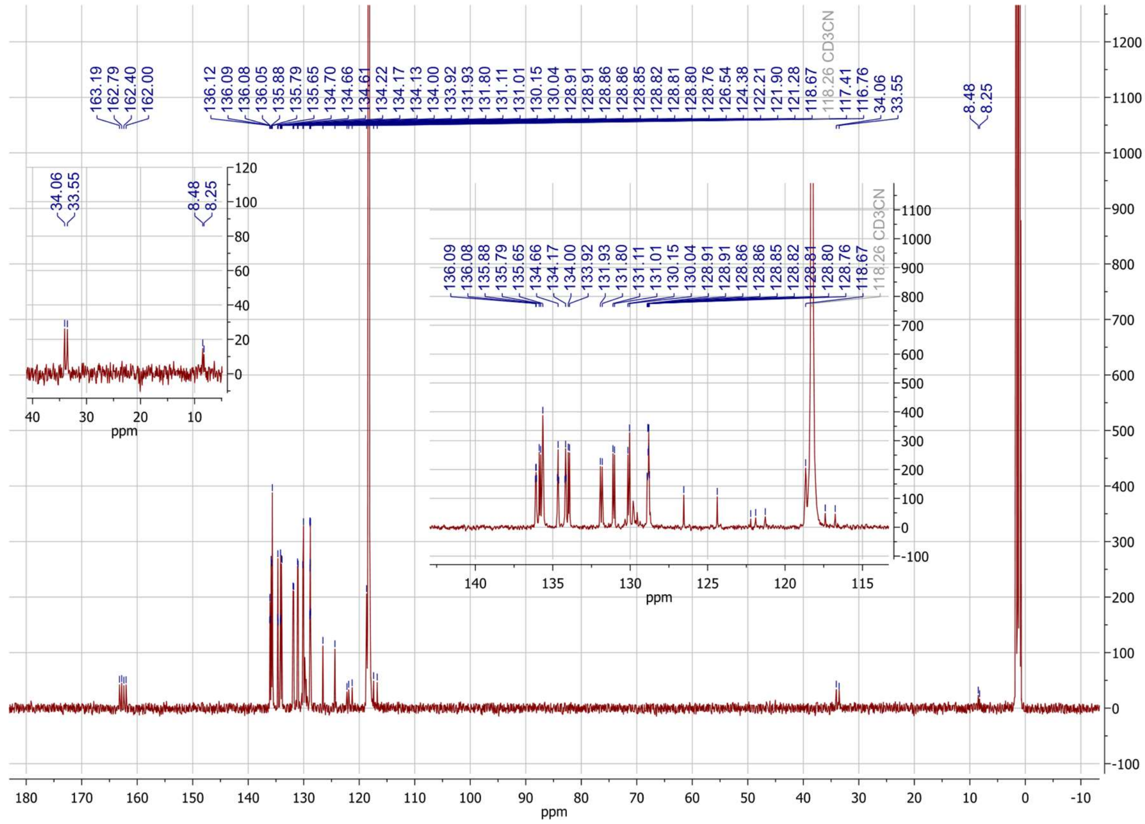

Figure S52 $^{13}C\{^1H\}$ NMR spectrum (125.66 MHz, acetonitrile-d$_3$, 300 K) of [({dppm}$_2$CH)RhCl$_3$]BArF (**6-BArF**).

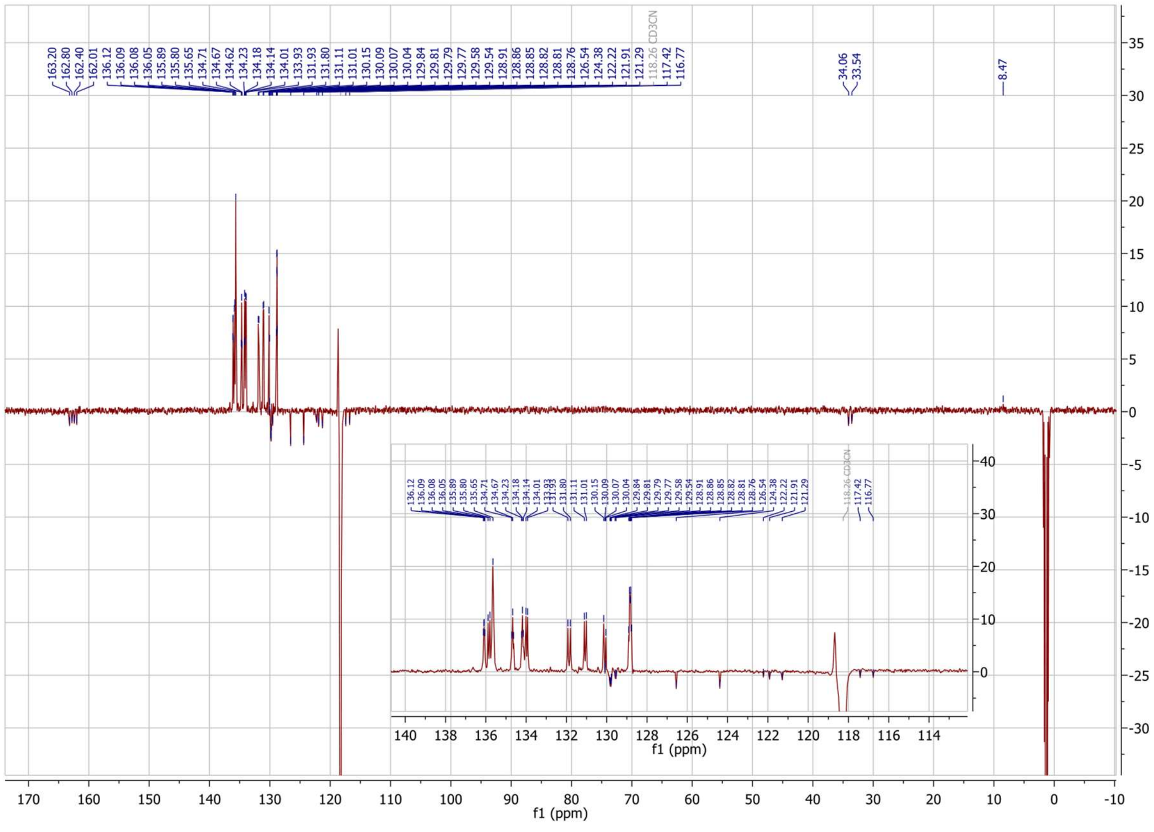

Figure S53 ^{13}C APT NMR spectrum (125.66 MHz, acetonitrile-d$_3$, 300 K) of [({dppm}$_2$CH)RhCl$_3$]BarF (**6-BArF**).

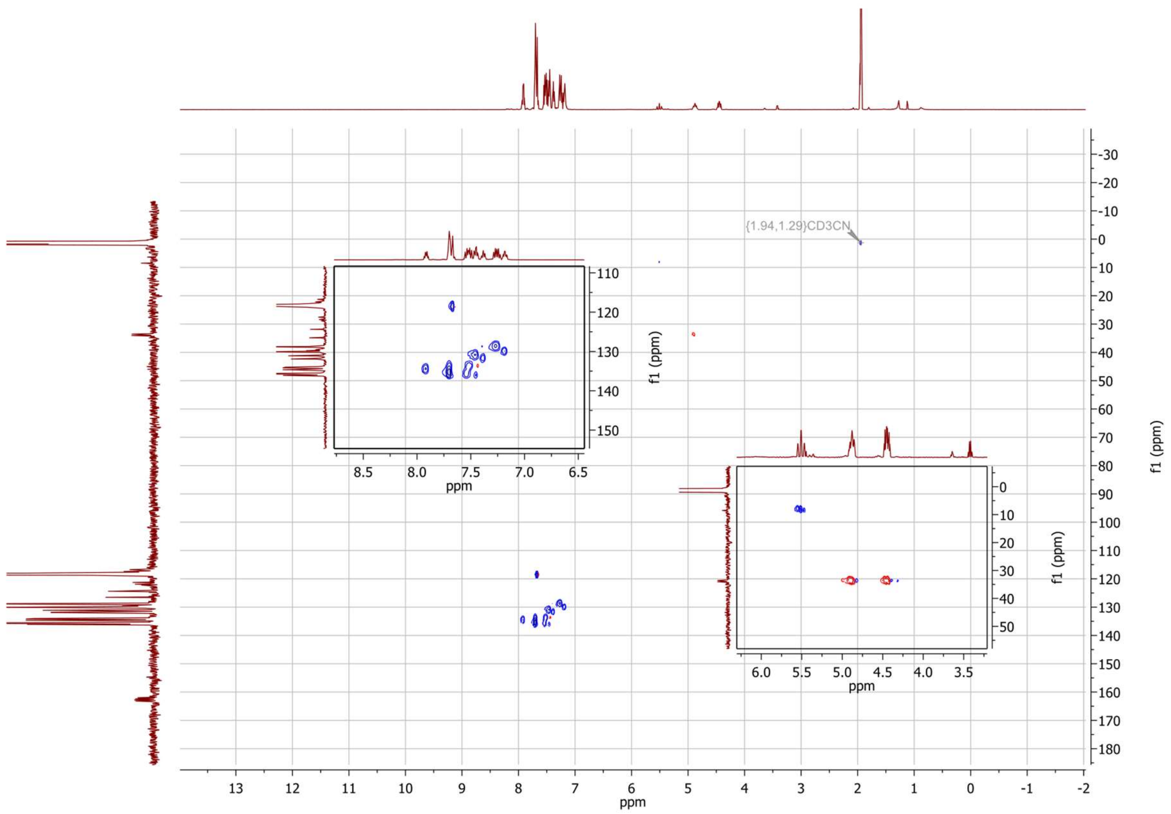

Figure S54 $^{1}H^{13}C$ HSQC NMR spectrum (499.72, 125.66 MHz, acetonitrile-d_3, 300 K) of [({dppm}$_2$CH)RhCl_3]BArF (**6-BarF**).

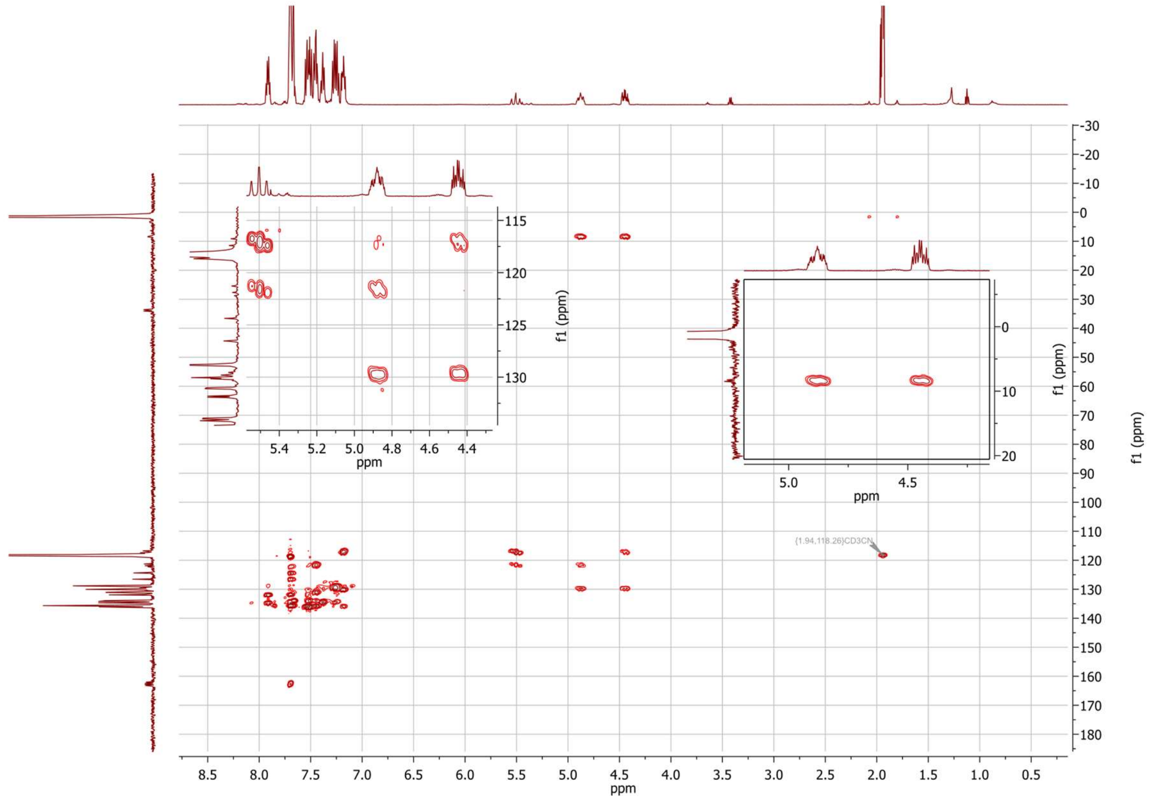

Figure S55 $^{1}H^{13}C$ HMBC NMR spectrum (499.72, 125.66 MHz, acetonitrile-d_3, 300 K) of [(({dppm}$_2$CH)RhCl_3]BArF (**6-BarF**).

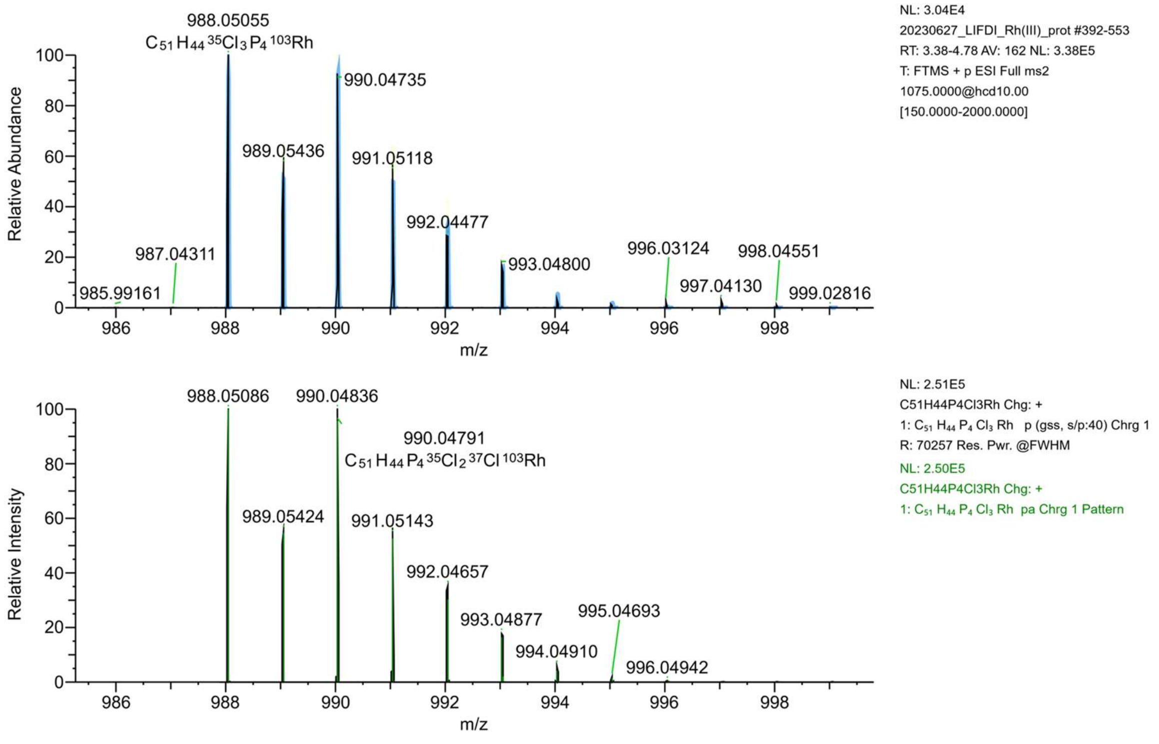

Peak Mass	Display F...	S Fit	RDB	Delta [pp...	Theo. ma...	Rank	Combine...	# Match...	# Missed...	MS Cov....	Pattern C...	MSMS M.
988.05055	$C_{51}H_{44}{}^{35}Cl_3P_4{}^{103}Rh$	38.723922166025 9	31.50	-0.32	988.05086	1	94.31	9	0	97.4	97.69	(Collection)

Figure S56 Top: Section of the LIFDI HRMS spectrum (positive mode) of [({dppm}$_2$CH)RhCl$_3$]Cl (**6-Cl**); Bottom: simulated isotope pattern. M$^+$ = [**6**-HCl]$^+$.

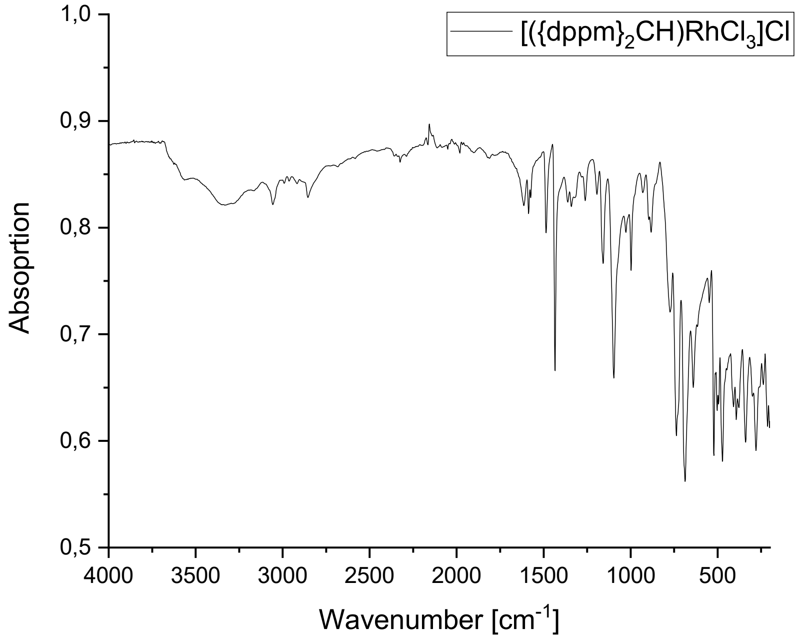

Figure S57 IR (ATR) spectrum of [({dppm}$_2$CH)RhCl$_3$]Cl (**6**).

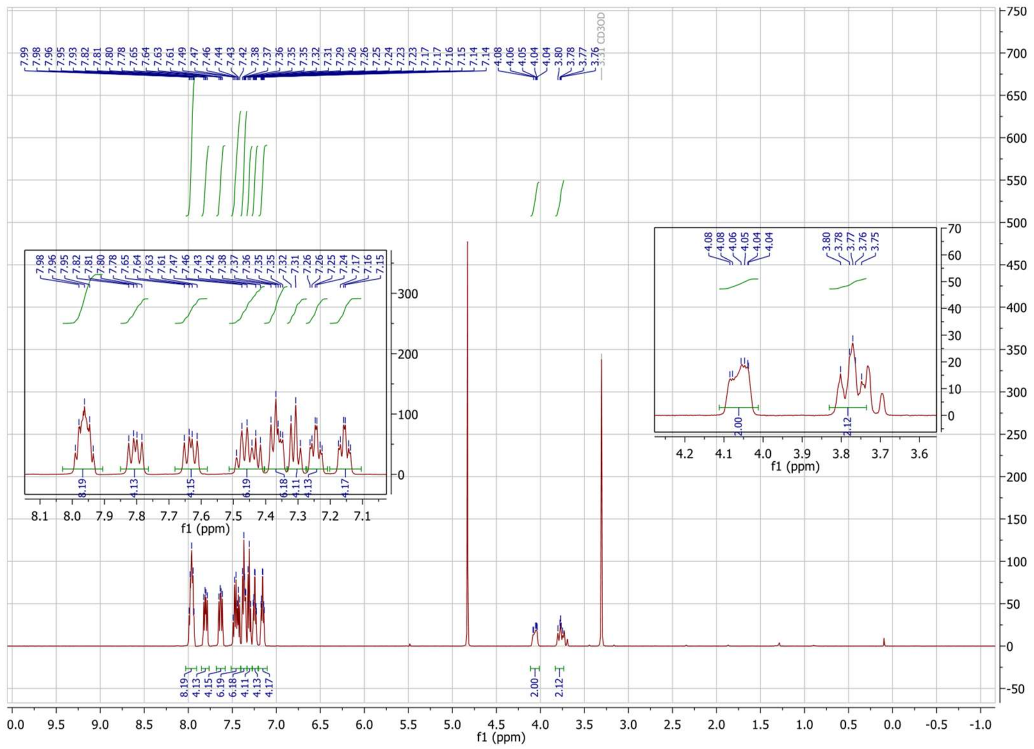

Figure S58 ^{1}H NMR spectrum (499.71 MHz, methanol-d$_4$, 300 K) of [({dppm}$_2$CH)RhCl]Cl (**7-Cl**). Note: PC(H)P obscured due to H/D exchange with CD_3OD.

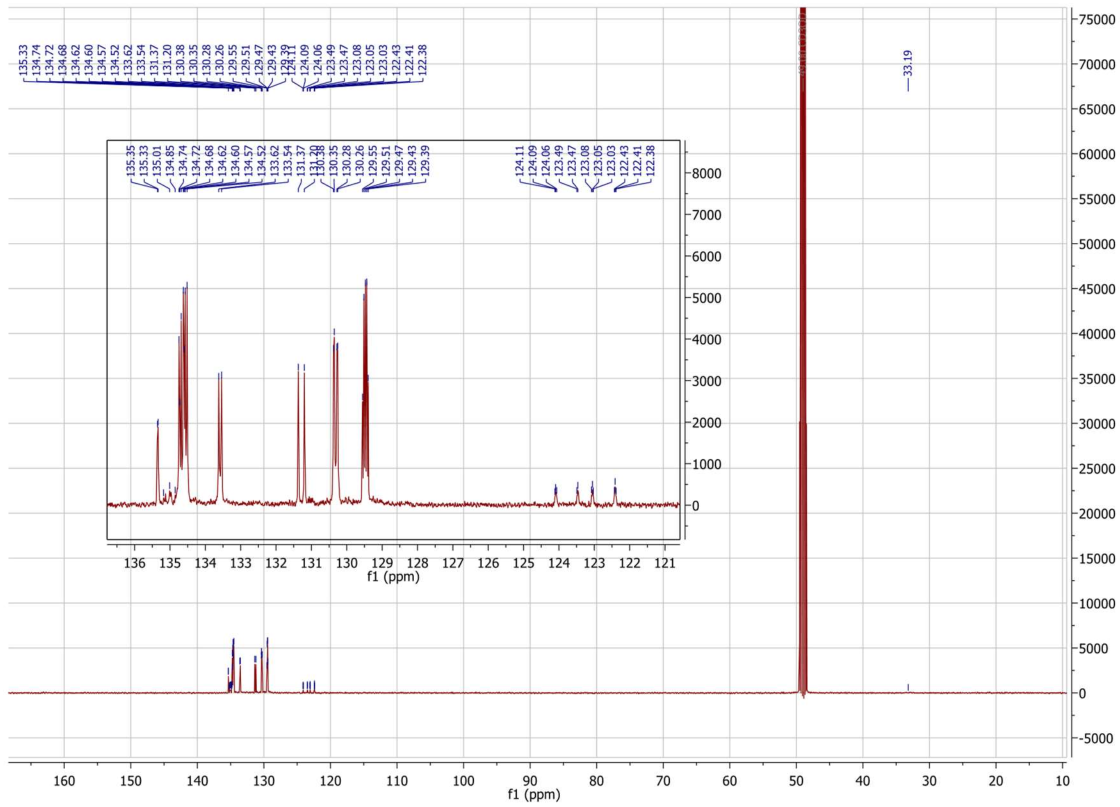

Figure S59 ^{13}C{^{1}H} NMR spectrum (125.67 MHz, methanol-d$_4$, 300 K) [({dppm}$_2$CH)RhCl]Cl (**7-Cl**) with insert of the aromatic section.

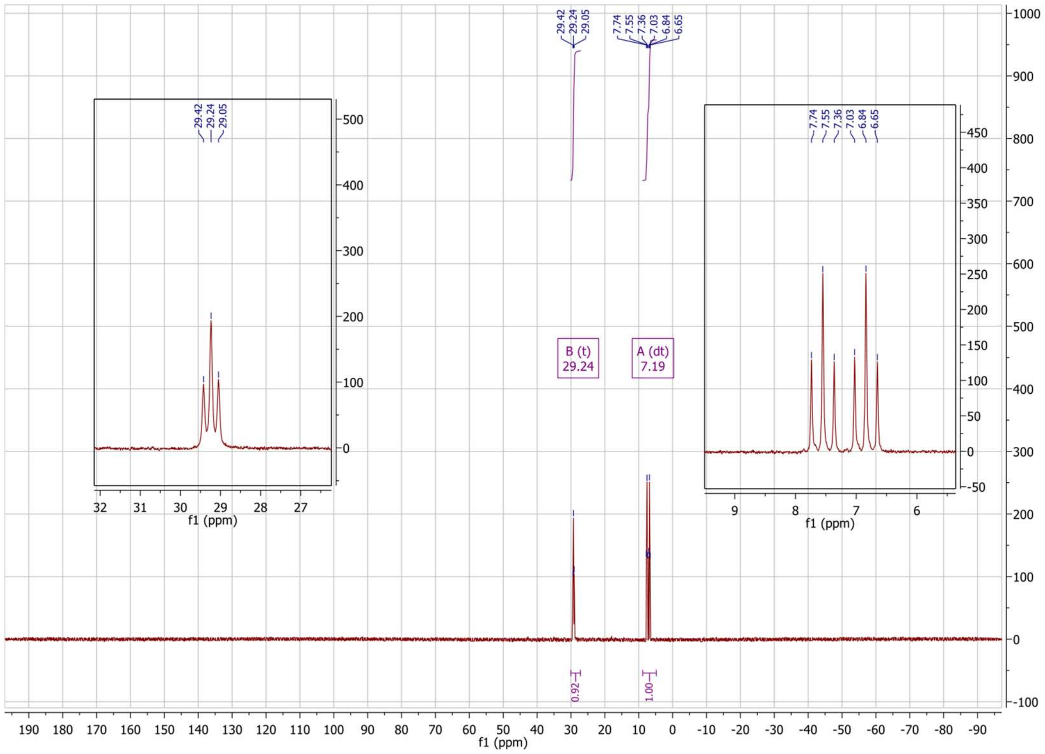

Figure S60 $^{31}P\{^{1}H\}$ NMR spectrum (202.30 MHz, methanol-d_4, 300 K) of [({dppm}$_2$CH)RhCl]Cl (**7-Cl**).

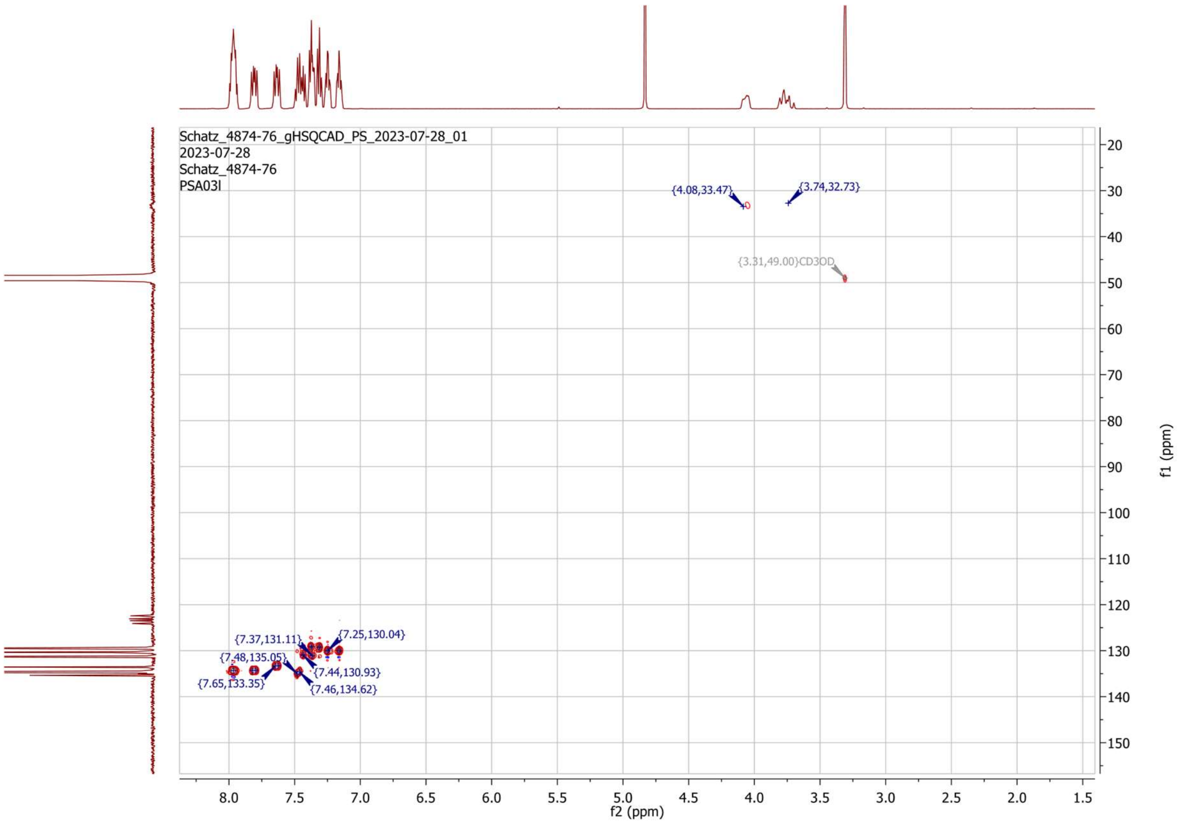

Figure S61 $^{1}H^{13}C$ HSQC NMR spectrum (499.71, 125.66 MHz, methanol-d_4, 300 K) of [({dppm}$_2$CH)RhCl]Cl (**7-Cl**).

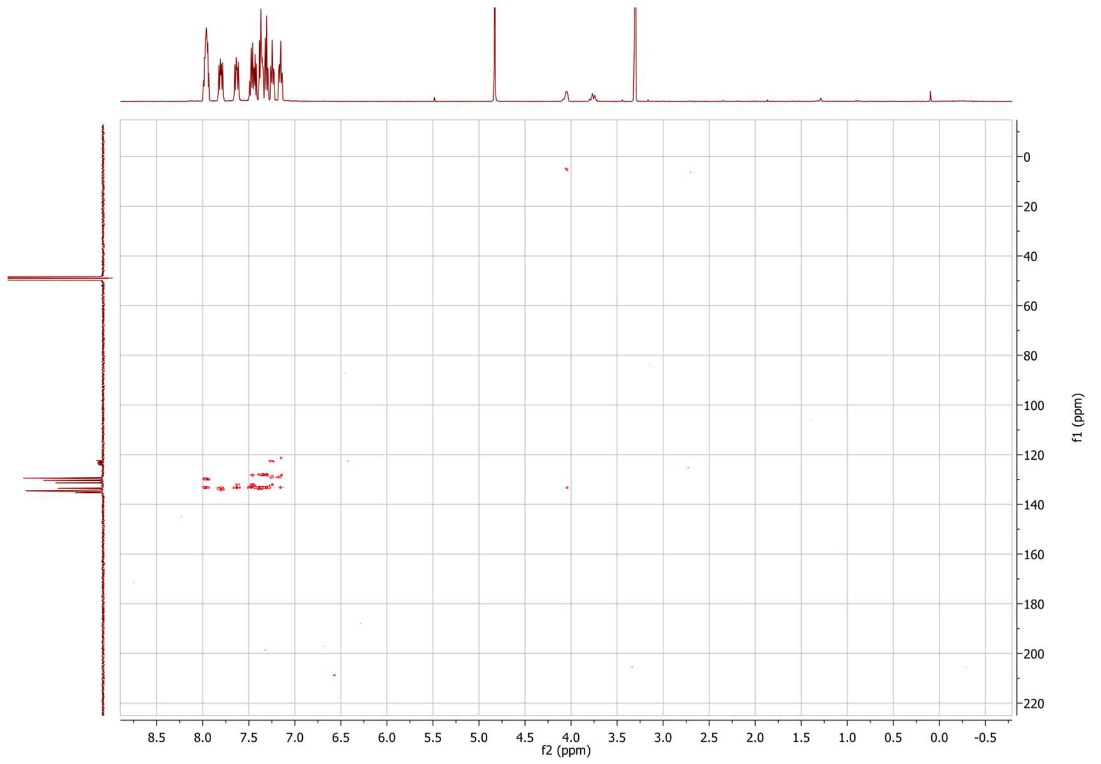

Figure S62 $^{1}H^{13}C$ HMBC NMR spectrum (499.71, 125.66 MHz, methanol-d$_4$, 300 K) of [({dppm}$_2$CH)RhCl]Cl (**7-Cl**).

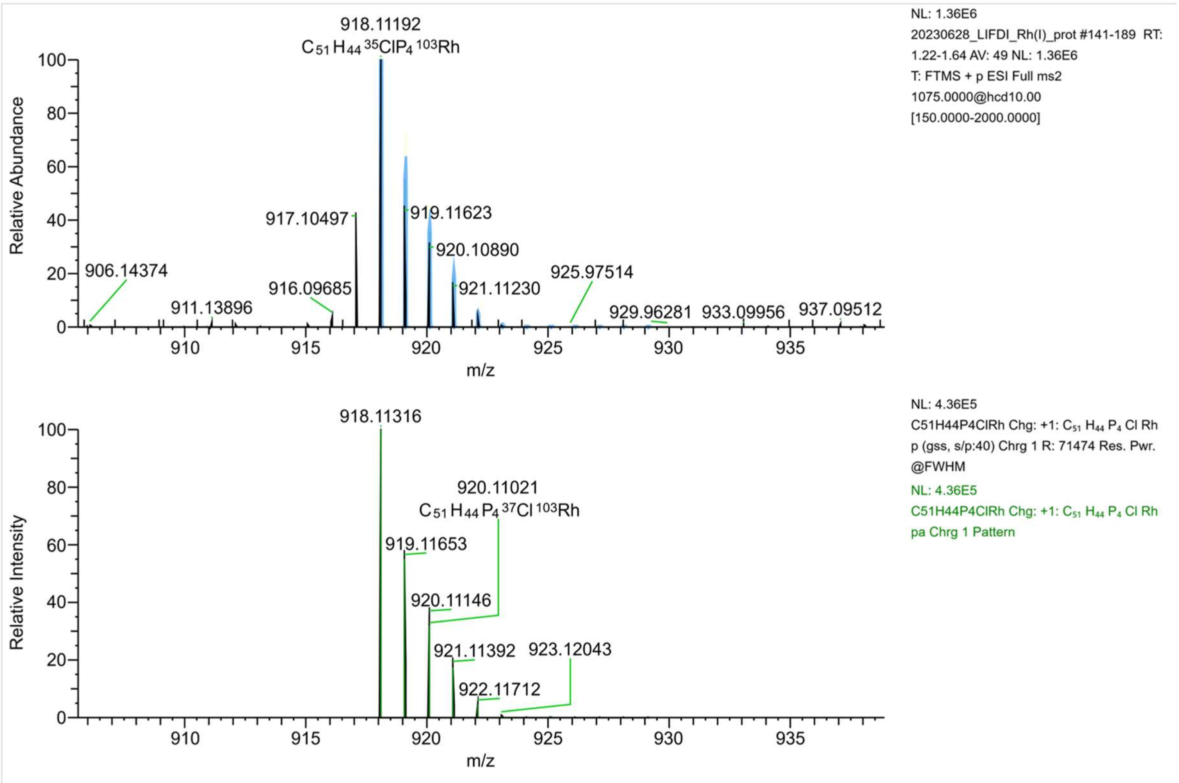

Peak Mass	Display F...	S Fit	RDB	Delta [pp...	Theo. ma...	Rank	Combine...	# Match...	# Missed...	MS Cov....	Pattern C...	MSMS M...
918.1119 2	$C_{51}H_{44}{}^{35}ClP_4{}^{103}Rh$	30.44225 5091048 9	32.50	-1.35	918.1131 6	1	95.91	7	0	99.54	99.55	(Collecti on)

Figure S63 Top: Section of the HRMS spectrum (positive mode) of [({dppm}$_2$CH)RhCl]Cl (**7-Cl**); Bottom: simulated isotope pattern. M^+ = [**7**-HCl]$^+$.

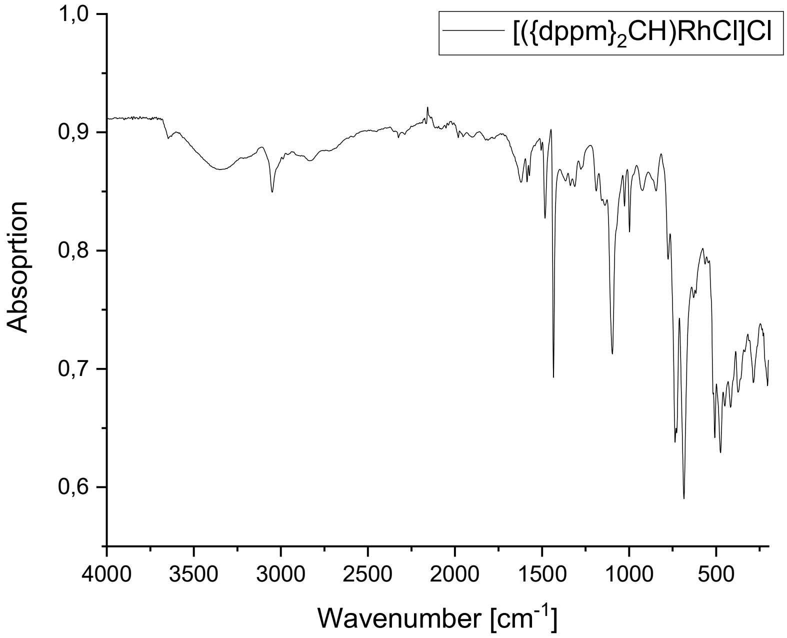

Figure S64 IR (ATR) spectrum of [({dppm}$_2$CH)RhCl]Cl (**7-Cl**).

13. Reactivity Study of Complex **4**

*Reactivity of [({dppm}$_2$C)RhCl$_3$]OTf (**4**) towards thiophenol Ph-SH*

The reaction study was performed in a J. YOUNG-NMR-Tube under inert conditions. The reaction was closely followed stepwise by means of NMR spectroscopy and HR-mass spectrometry (See spectral data below). The starting material Ph-SH was evaluated with respect to Ph-S-S-Ph impurities in a control experiment diluting 0.5 µl (5.06 µmol) in 0.8 mL DCM prior subjection to the HR-MS measurement. Only trace amounts of Ph-SH were detected under the experimental conditions (see spectrometric data below).

6.0 mg (5.27 µmol) of [({dppm}$_2$C)RhCl$_3$]OTf (**4**) were dissolved in 0.8 mL DCM-d$_2$ to form a dark green suspension. To this suspension 0.5 µl (4.90 µmol, 0.93 equiv.) of Ph-SH were added with an EPPENDORF-pipette. The reaction mixture was intermittently shaken at ambient temperature and allowed to react for 15 minutes, which led to a colour change to a brownish yellow. Subsequently, another 0.5 µl portion of Ph-SH was added into the J. YOUNG -NMR-Tube and the mixture was intermittently shaken at ambient temperature for additional 15 minutes. The reaction was followed by means of *^{31}P{^{1}H} NMR* spectroscopy and HR-mass spectrometry (See spectral data below)

*Reactivity of [({dppm}$_2$C)RhCl$_3$]OTf (**4**) towards tributyltinhydride (H-SnBu$_3$)*

The reaction study was performed in a J. YOUNG-NMR-Tube under inert conditions. The reaction was closely followed by means of NMR spectroscopy. Spectra are displayed in Figure S70-S71 below.

*Reaction of **4** with HSnBu$_3$*

12.5 mg (10.97 µmol) of [({dppm}$_2$C)RhCl$_3$]OTf (**4**) were dissolved in 0.8 mL DCM to form a dark green suspension. Subsequently, H-SnBu$_3$ (2.9 µl, 10.97 µmol, 1.0 equiv.) was added to the suspension via an EPPENDORF-pipette. The colour of the reaction mixture immediately changes to orange-yellow. The ^{31}P{^{1}H} NMR spectrum is displayed in Figure S70 C exhibiting resonances associated with a mixture of complex **6** and **7**.

Upon further treatment of the solution with excess H-SnBu$_3$ (5.9 µl, 21.94 µmol, 2.0 equiv.) the colour of the reaction mixture changes to orange. Inspection of the ^{31}P{^{1}H} NMR reveals the formation of complex **7** as the final product Figure S70 D.

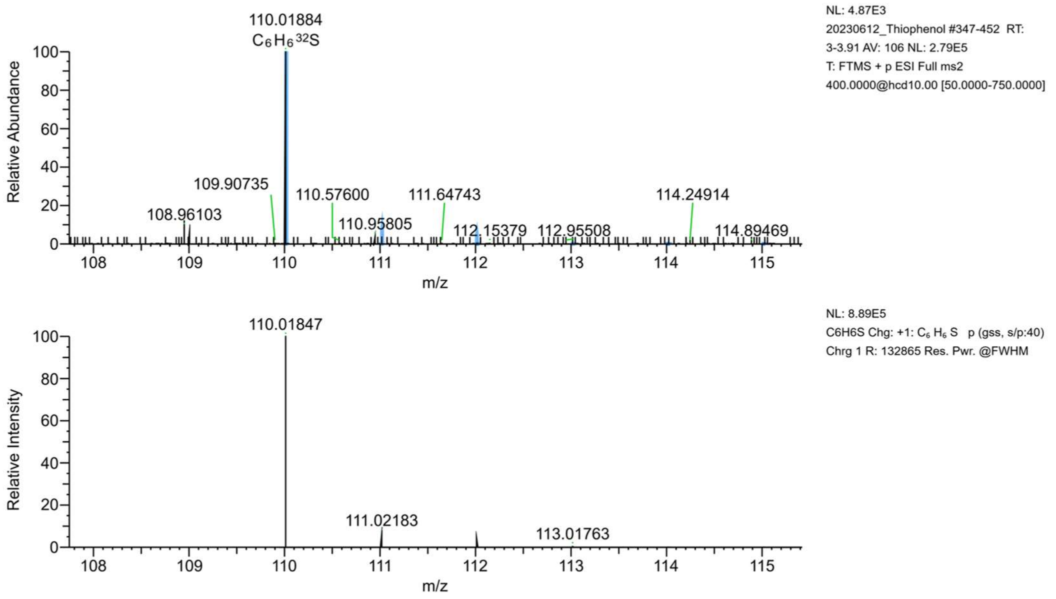

Peak Mass	Display F...	S Fit	RDB	Delta [pp...	Theo. ma...	Rank	Combine...	# Match...	# Missed...	MS Cov....	Pattern C...	MSMS M..
110.0188 4	$C_6H_6{}^{32}S$	13.80223 3846306 7	4.00	3.38	110.0184 7	1	82.1	3	0	85.89	90.08	(Collecti on)

Figure S65 Control experiment; assessment of the purity of Ph-SH. Section of the HRMS spectrum (positive mode) of 6.13 mM thiophenol in DCM Top: $(PhSH)^+$ experimental vs. simulated isotope pattern.

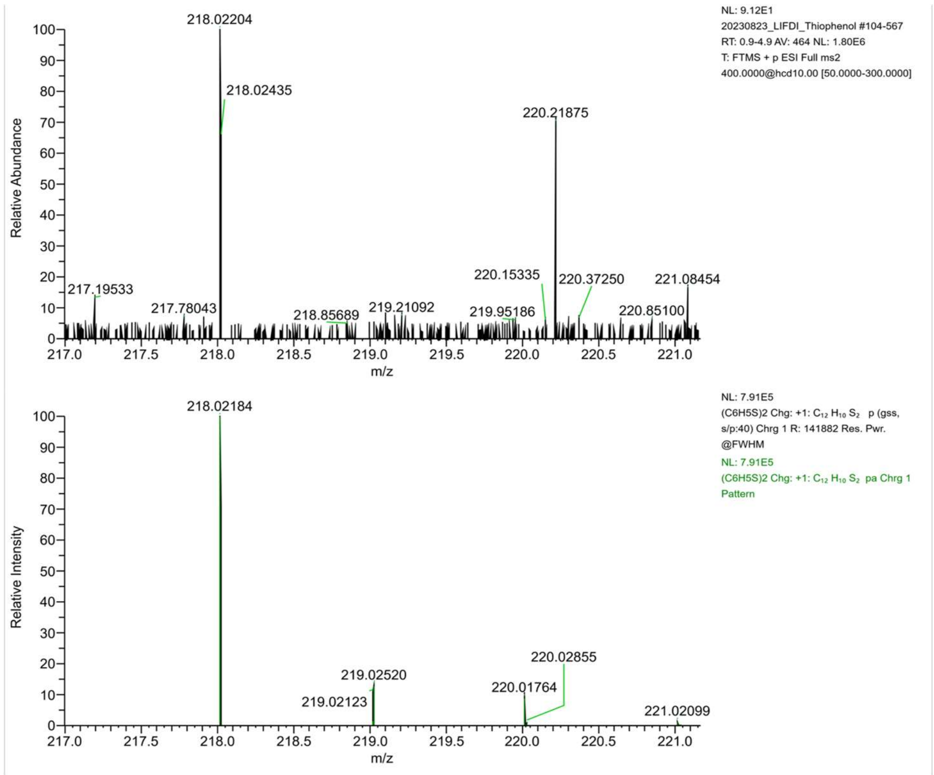

Figure S66 Control experiment; assessment of the purity of Ph-SH. Section of the LIFDI HRMS spectrum (positive mode) of 6.13 mM thiophenol in DCM. $[PhS\text{-}SPh]^+$ experimental (top) vs. simulated (bottom) isotope pattern. Note, this control experiment indicates only trace amounts of PhS-SPh being present in the starting material solution at the same PhSH concentration with respect to the reactivity study below.

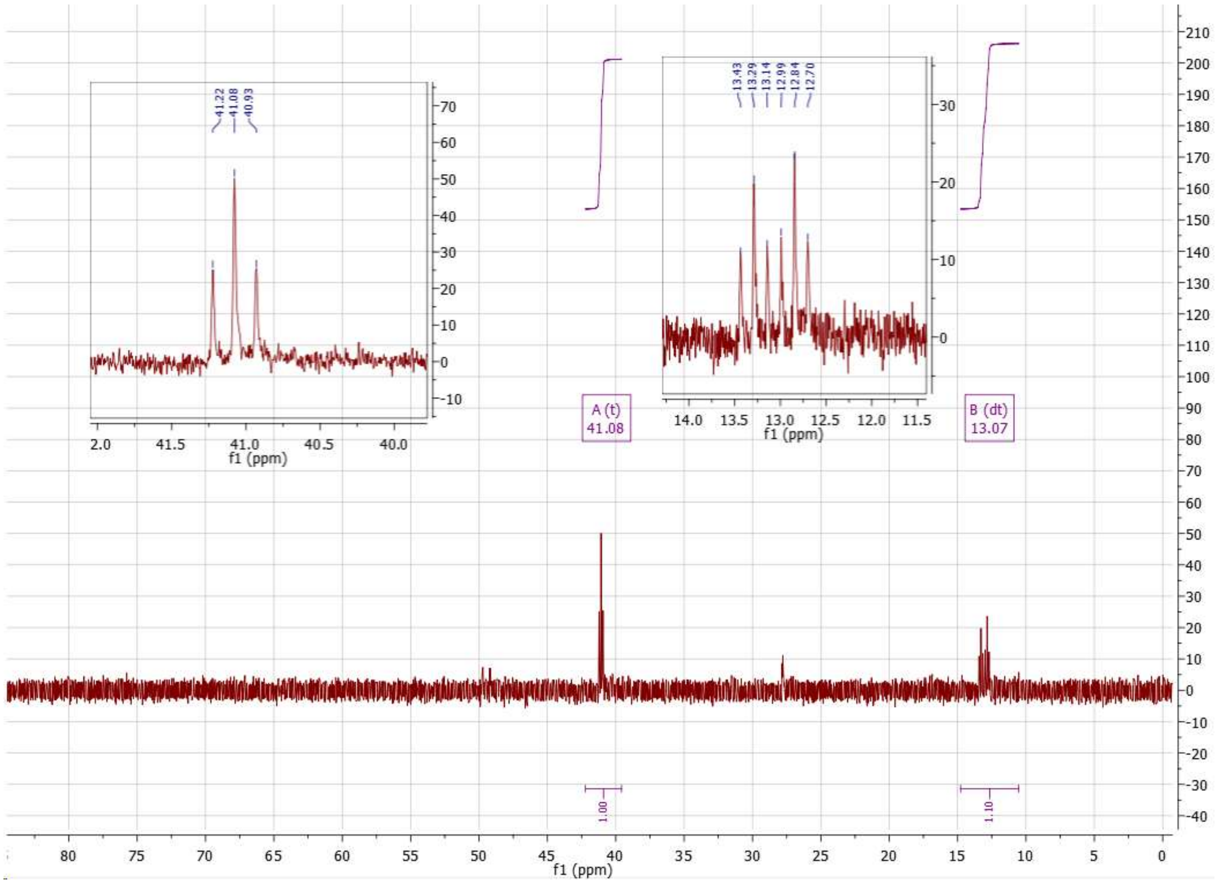

Figure S67 Reactivity study; $^{31}P\{^{1}H\}$ NMR (202.3 MHz, dichlormethane, 300 K) spectrum of the reaction mixture in DCM of **4** and Ph-SH (two times 0.5µL portions of Ph-SH, after 30 min at ambient temperature).

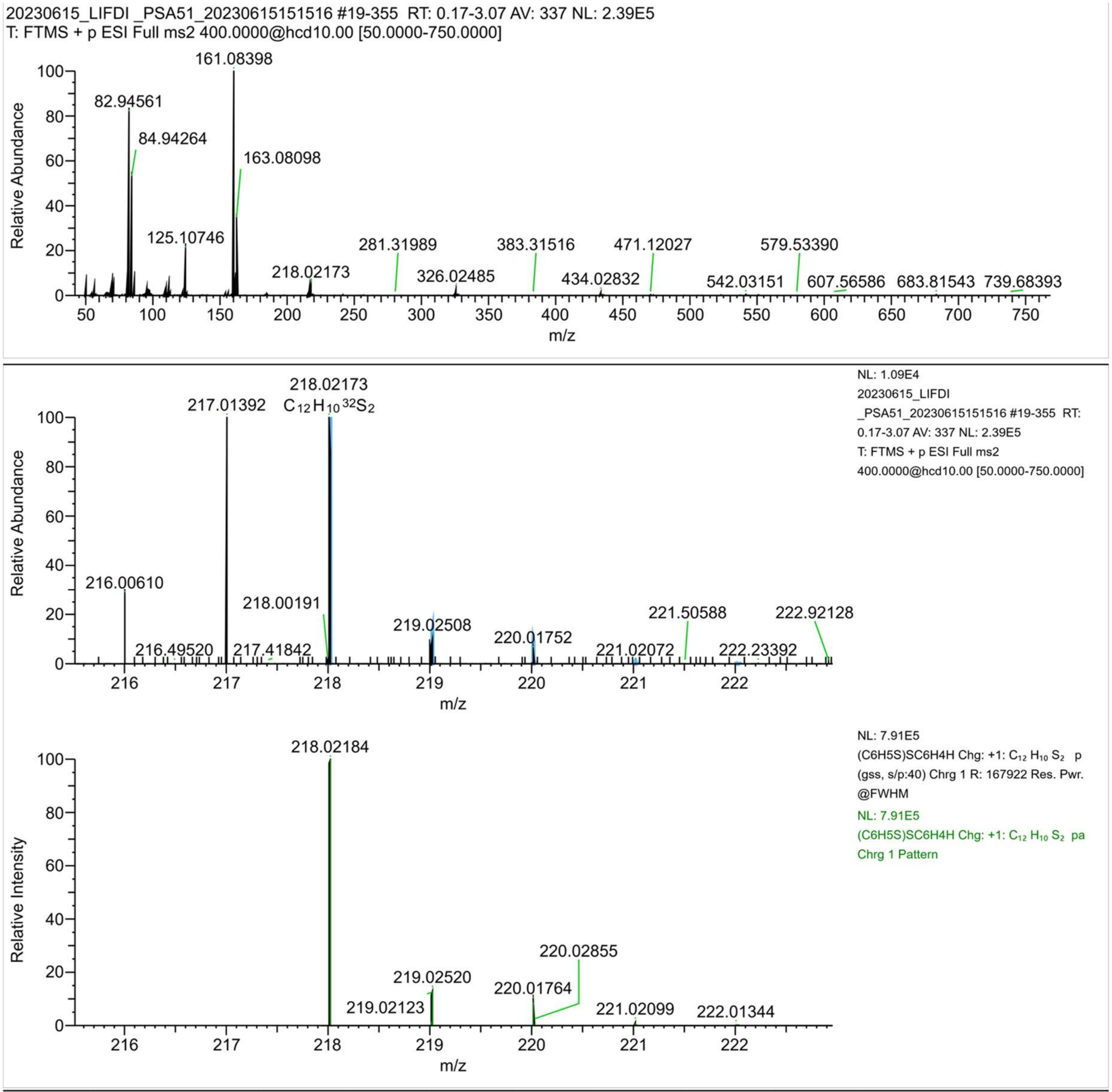

Peak Mass	Display F...	S Fit	RDB	Delta [pp...	Theo. ma...	Rank	Combine...	# Match...	# Missed...	MS Cov....	Pattern C...	MSMS M...
218.02173	$C_{12}H_{10}{}^{32}S_2$	15.992379793887 9	8.00	-0.54	218.02184	1	82.19	6	0	85.87	89.98	(Collection)

Figure S68 Reactivity study; Top: Full HRMS spectrum (LIFDI, positive mode) of the in situ-sampled reaction mixture of **4** and PhSH in DCM (6.13 mM thiophenol in DCM) Middle: Section of the HRMS spectrum showing experimental isotope pattern of $[PhS\text{-}SPh]^+$ (218.02173 m/z) at significant intensity along with $[PhS\text{-}SPh]\text{-}H^+$ (217.01392 m/z). Bottom: simulated isotope pattern of $[PhS\text{-}SPh]^+$.

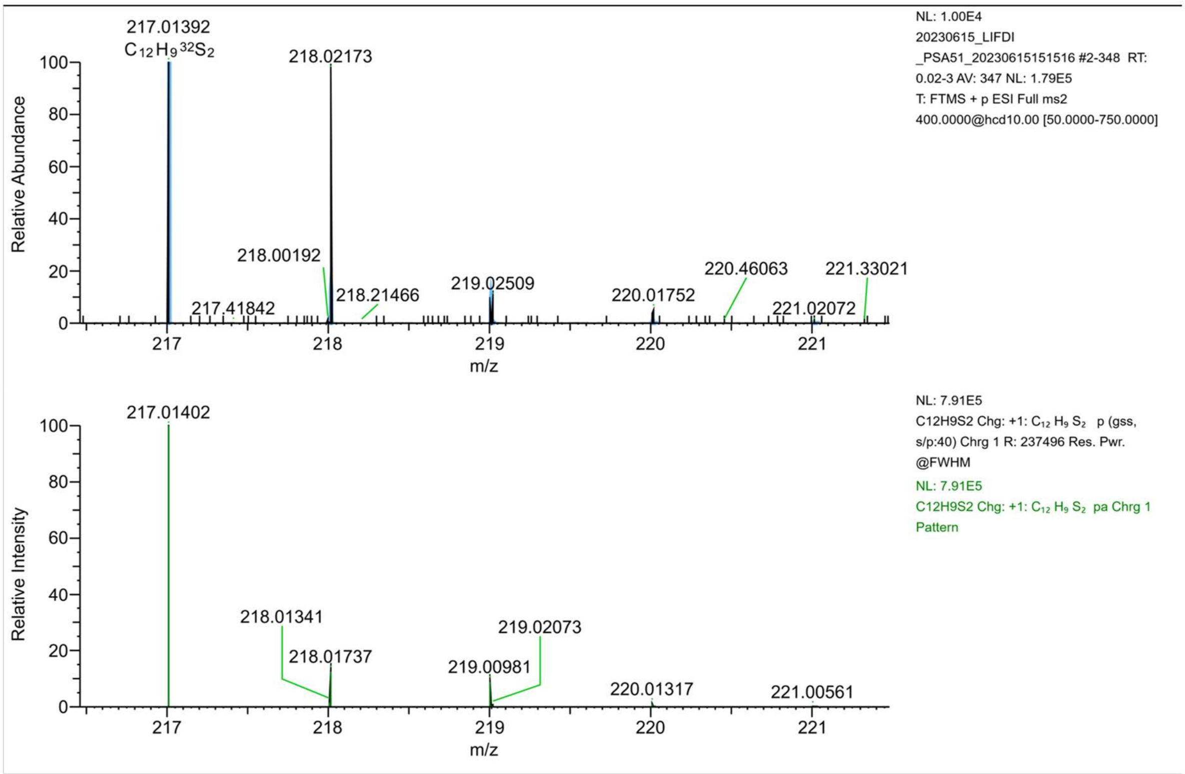

Peak Mass	Display F...	S Fit	RDB	Delta [pp...	Theo. ma...	Rank	Combine...	# Match...	# Missed...	MS Cov....	Pattern C...	MSMS M...
217.01392	$C_{12}H_9{}^{32}S_2$	25.2020311984047	8.50	-0.47	217.01402	1	49.65	6	0	51.01	97.18	(Collection)

Figure S69 Reactivity study;Top: Section of the HRMS spectrum (LIFDI, positive mode) of the in situ-sampled reaction mixture of **4** and PhSH in DCM (6.13 mM thiophenol in DCM) showing the isotope pattern of [PhS-SPh]-H^+ (217.01392 m/z) at significant intensity along with $[PhS\text{-}SPh]^+$ (218.02173 m/z). Bottom: simulated isotope pattern of [PhS-SPh]-H^+.

Reactivity of [({dppm}$_2$C)RhCl$_3$]Otf with Bu$_3$SnH

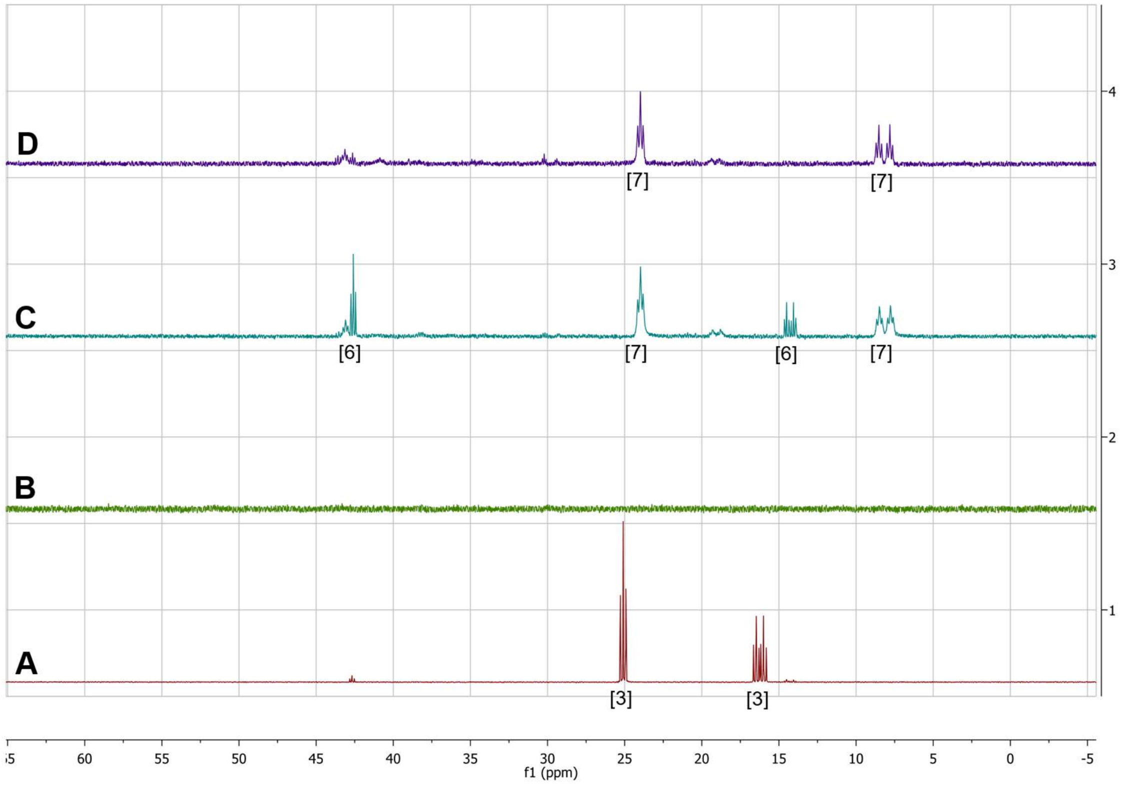

Figure S70 Stacked $^{31}P\{^1H\}$ NMR spectra (202.30 MHz, dichlormethane, 300 K) of (**A**) [({dppm}$_2$C)RhCl$_3$] (**3**); (**B**) [({dppm}$_2$C)RhCl$_3$]OTf (**4**) (NMR silent); (**C**) addition of Bu$_3$SnH to (**4**) – formation of [({dppm}$_2$CH)RhCl$_3$]OTf (**6**) and [({dppm}$_2$CH)RhCl]OTf (**7-OTf**); (**D**) final product [(({dppm}$_2$CH)RhCl]OTf (**7**).

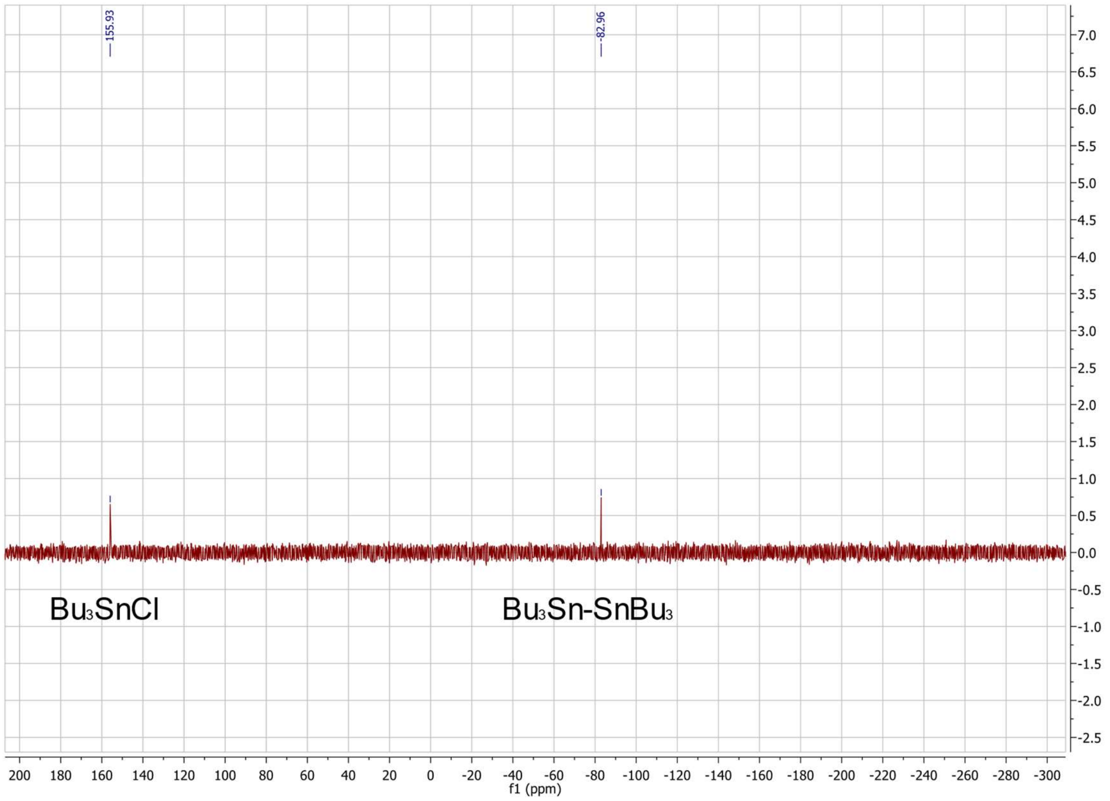

Figure S71 ^{119}Sn NMR spectra (186.33 MHz, DCM, 300 K) recoded after the reaction between complex **4** and Bu_3SnH.

Reactivity study of [({dppm}$_2$C)RhCl$_3$]BArF (4-BArF) with H_2 and D_2 gas

(I, H_2) A *J. Young* NMR tube fitted with a *Kontes* valve was charged with [({dppm}C)RhCl$_3$]BArF (**4-BArF**, 10 mg, 0.005 mmol). The solid was dissolved in 0.7 mL CD_2Cl_2 to give a dark green solution, which was subjected to ^{1}H and ^{31}P{^{1}H} NMR spectroscopic measurements. The spectra remained broad (^{1}H) and featureless (^{31}P) as expected for the paramagnetic species (blank spectra).

Subsequently, the solution was subjected to a slight overpressure (0.1 bar) of H_2 gas and the solution was vigorously shaken to saturate the CD_2Cl_2 with H_2 (note the ^{1}H NMR resonance of dissolved H_2 in the spectra). The reaction was monitored: NMR spectra were recorded after ca 4 h and overnight (ca 12 h), after which a bright yellow solution was formed. The spectra indicated the formation of the protonated complex [({dppm}$_2$CH)RhCl$_3$]BArF (**6-BArF**).

(II, D_2) A *J. Young* NMR tube fitted with a *Kontes* valve was charged with [({dppm}C)RhCl$_3$]BArF (**4-BArF**, 33 mg, 0.018 mmol and dissolved in 0.6 mL CH_2Cl_2.to give a dark green solution A ^{31}P{^{1}H} NMR spectrum was recorded to obtain the starting (blank) spectrum. The spectrum remained featureless as expected for the paramagnetic species in

solution. Subsequently, a D_2 overpressure of 0.9 bar was applied. The tube was purged three times with D_2/Vac ant the Solution was vigorously shaken to ensure saturation. The ^{2}H NMR spectrum included the resonance associated with D_2 gas indicating sufficient D_2 gas dissolved in the reaction medium. ^{2}H NMR spectra were immediately measured and were consecutively recorded overnight. The spectra indicated the incorporation of deuterium into the ligand scaffold (methylene bridges and *C*DP moiety) to give [({dppm}$_2$CH)^{D}RhCl$_3$]BArF (**6^D-BArF**).

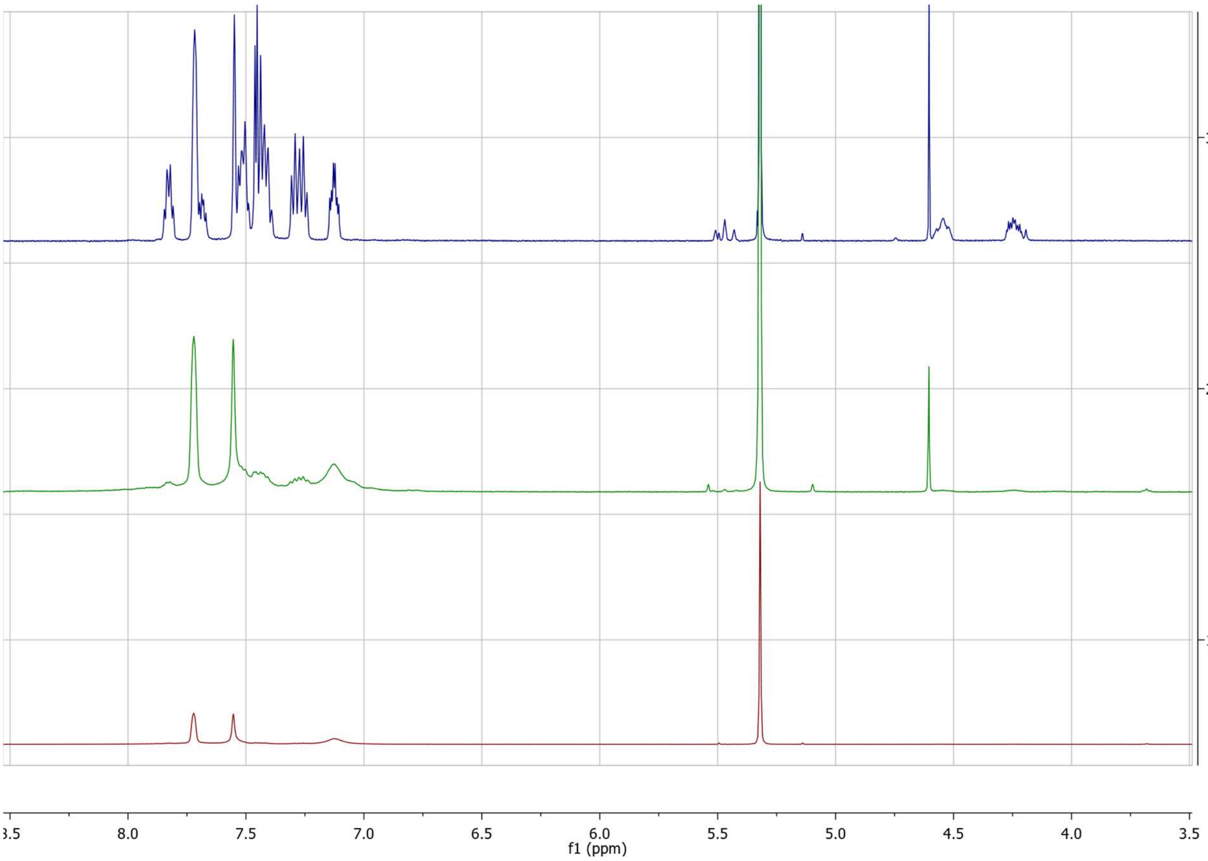

Figure S72 ^{1}H NMR spectrum ((502.28 MHz, CD_2Cl_2, 300 K) of [({dppm}C)RhCl$_3$]BArF (**4-BArF**) (bottom), under H_2 gas after 4h (middle), under H_2 gas after 12h (top).

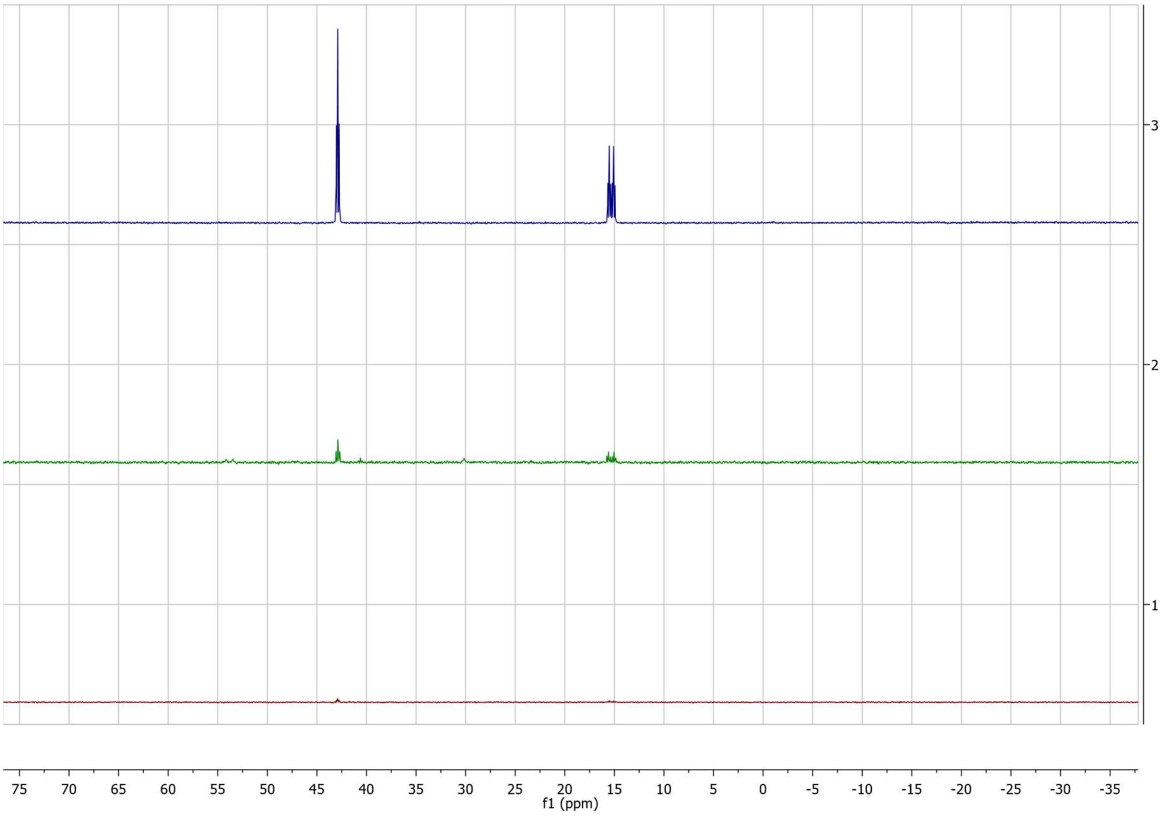

Figure S73 $^{31}P\{^1H\}$ NMR spectrum (203.3 MHz, CD_2Cl_2, 300 K) of [({dppm}C)RhCl$_3$]BArF (**4-BArF**) (bottom), under H_2 gas after 4h (middle), under H_2 gas after 20h (top).

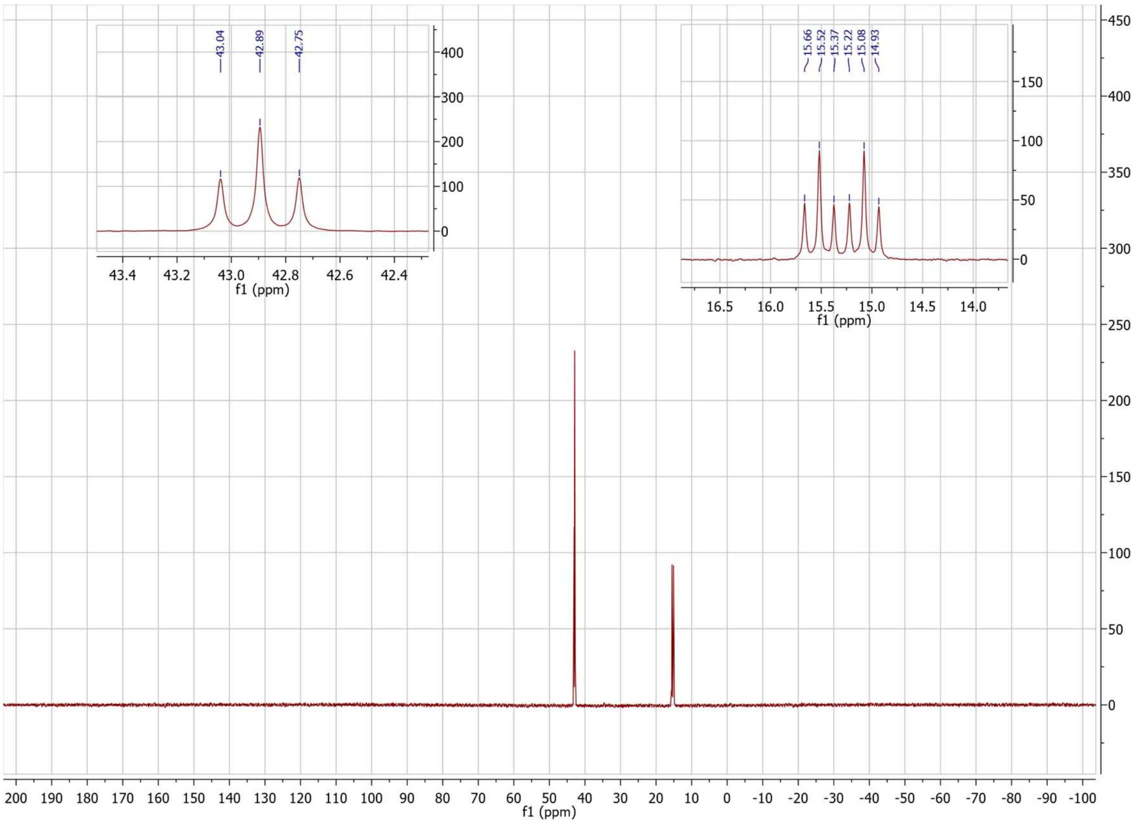

Figure S74 $^{31}P\{^1H\}$ NMR spectrum (203.3 MHz, CD_2Cl_2, 300 K) of [({dppm}C)RhCl$_3$]BArF (**4-BArF**) + H_2 gas (20h).

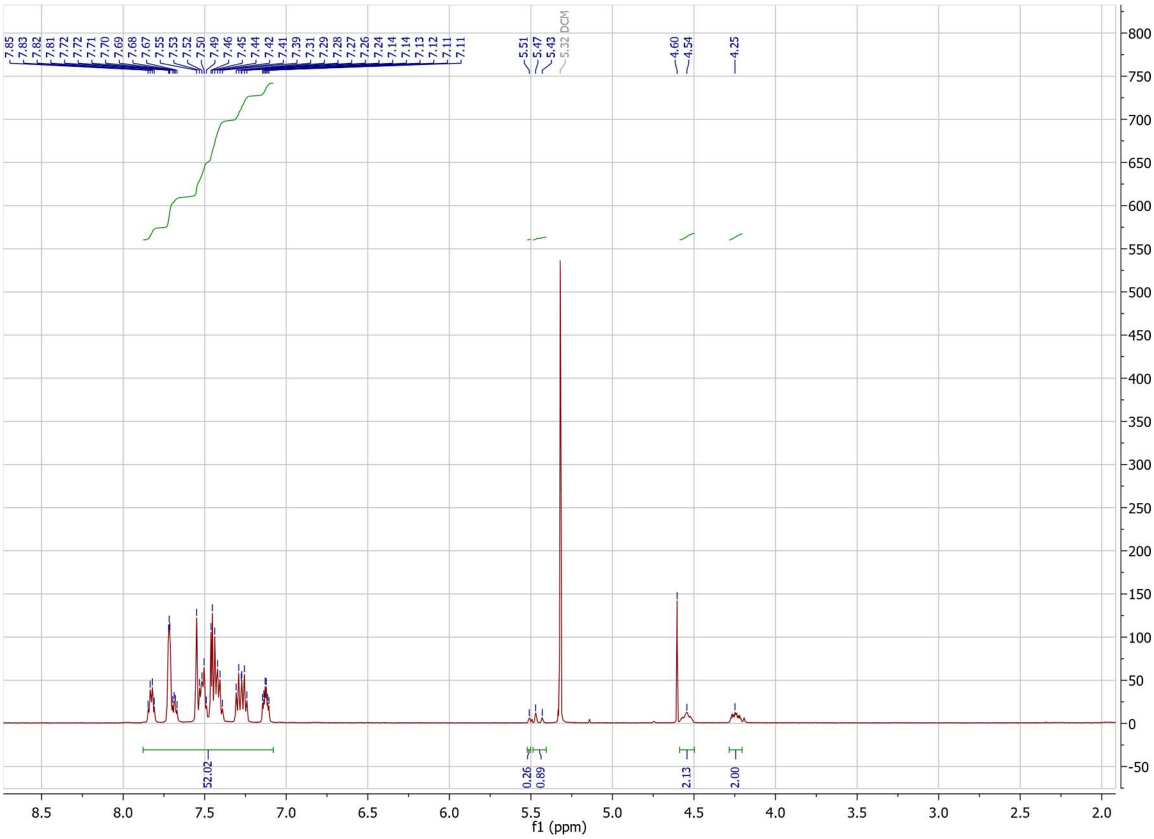

Figure S75 ^{1}H NMR spectrum (502.28 MHz, CD_2Cl_2, 300 K) of [({dppm}C)RhCl$_3$]BArF (**4-BArF**) + H_2 gas after 20h.

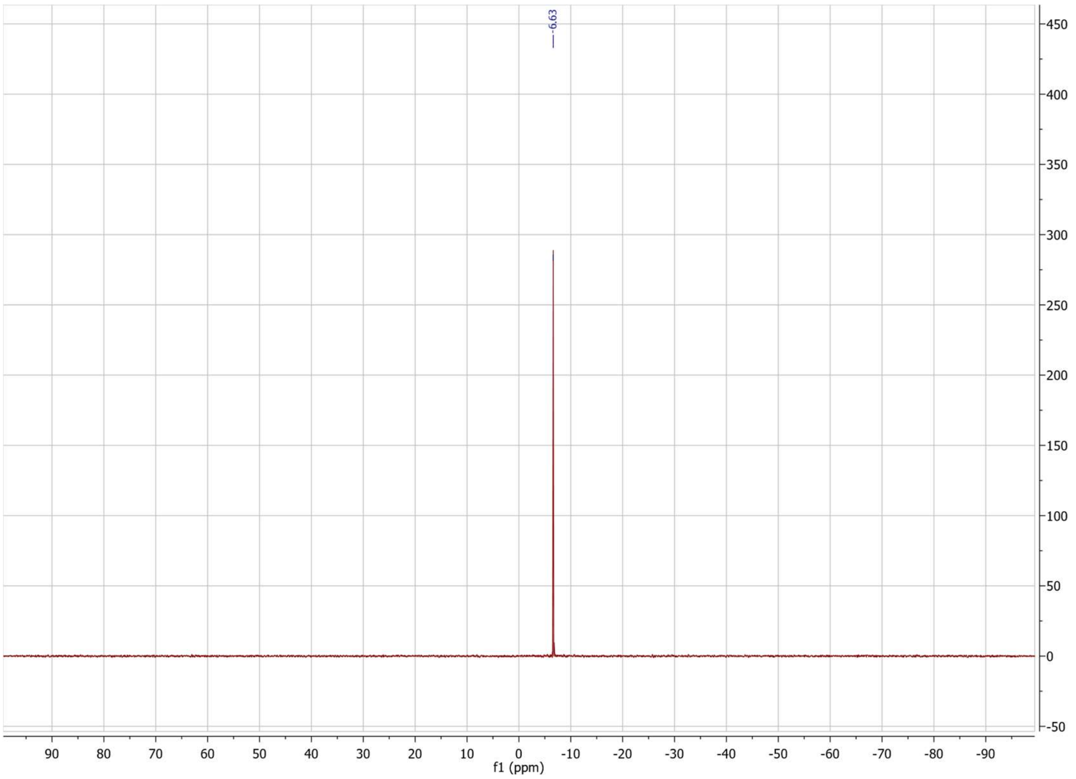

Figure S76 $^{11}B\{^{1}H\}$ NMR spectrum (161.2 MHz, CD_2Cl_2, 300 K) of [({dppm}C)RhCl$_3$]BArF (**4-BArF**) + H_2 gas after 48h.

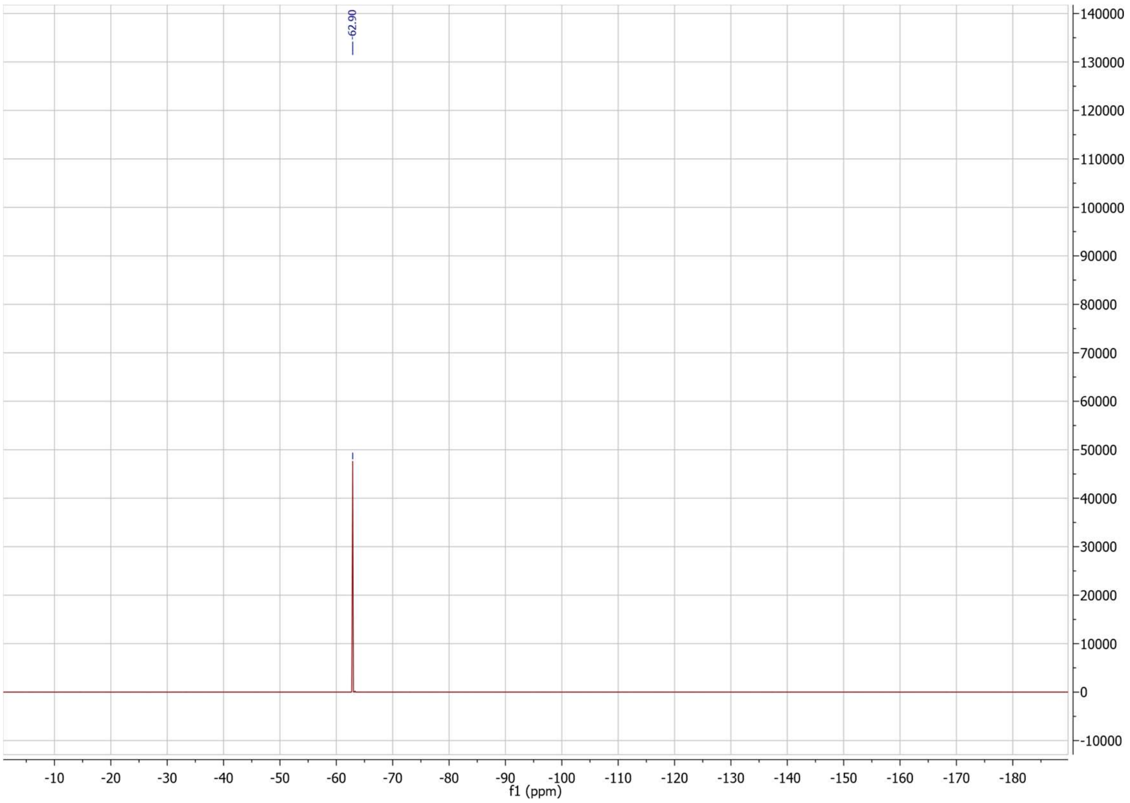

Figure S77 $^{19}F\{^1H\}$ NMR spectrum (472.6 MHz, CD_2Cl_2, 300 K) of [({dppm}C)$RhCl_3$]BArF (**4-BArF**) + H_2 gas after 48h.

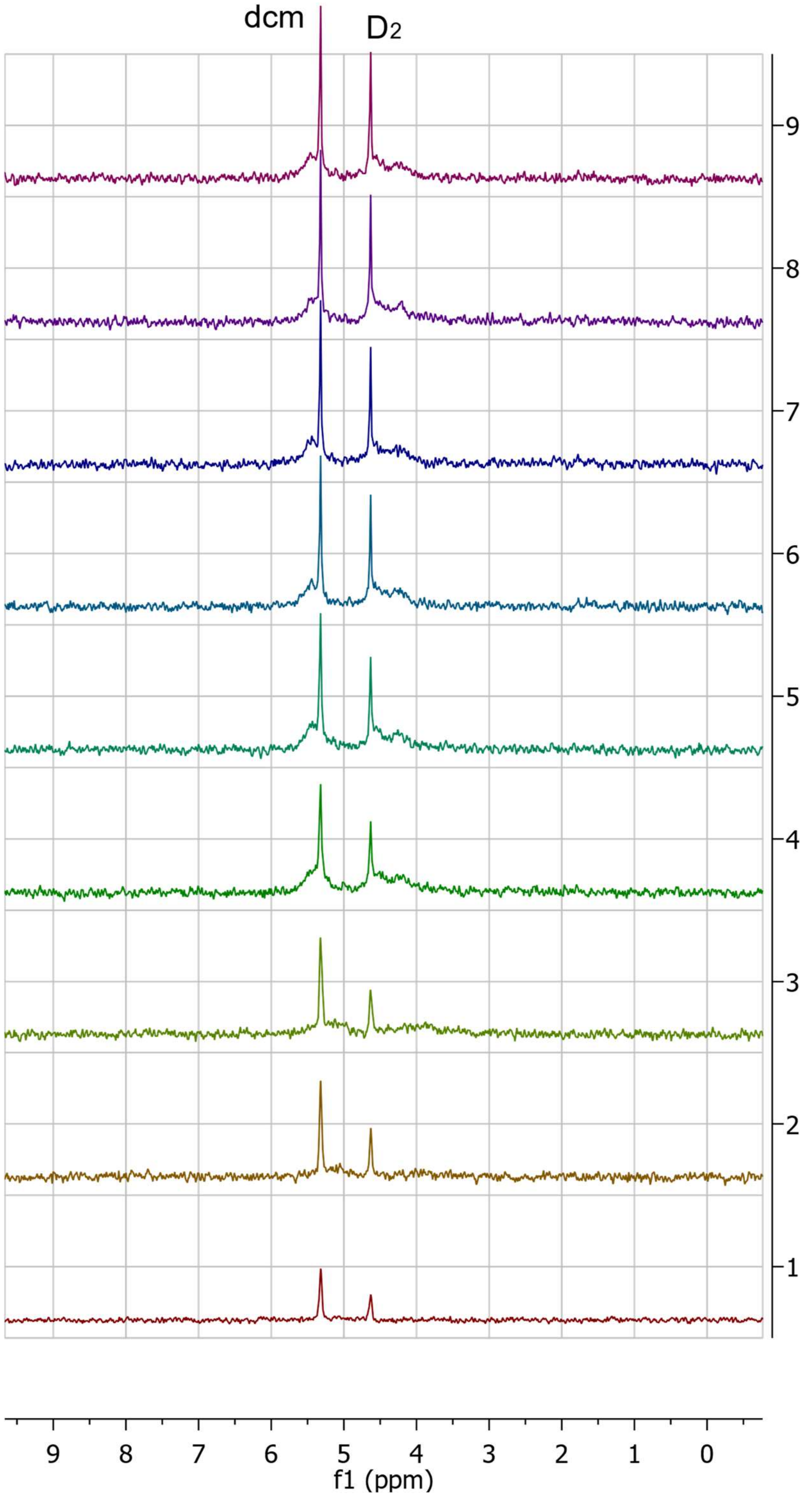

Figure S78 ^{2}H NMR spectrum (77.10 MHz, CH_2Cl_2, 300 K) of [({dppm}C)$RhCl_3$]BArF (**4-BArF**) + D_2 gas. Stacked spectra (0-20h).

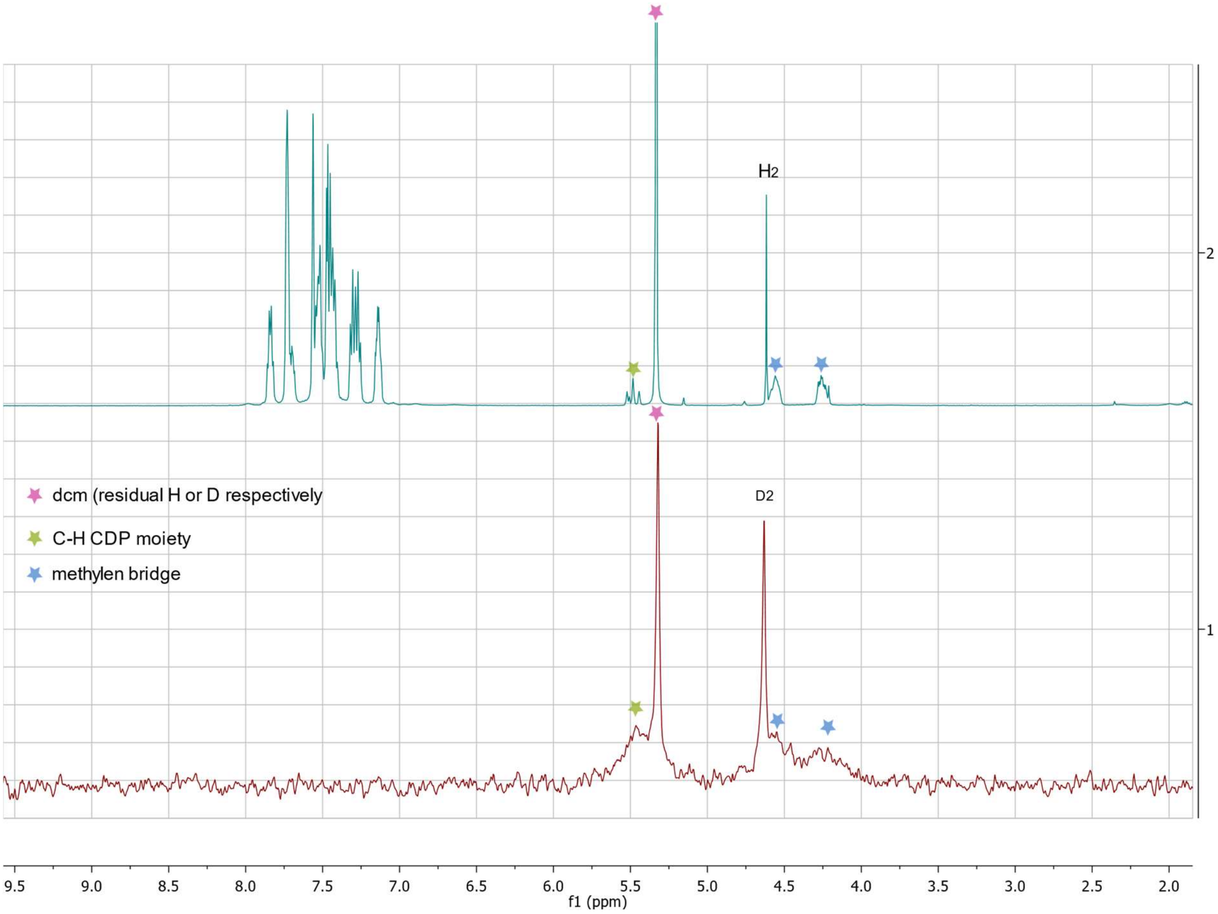

Figure S79 Stacked ^{1}H- (top) and ^{2}H NMR spectrum (bottom) (203.3 / 77.10 MHz, CD_2Cl_2/CH_2Cl_2, 300 K) of the reaction of [({dppm}C)$RhCl_3$]BArF (**4-BArF**) + H_2 (top) and D_2 gas (bottom).

14. Experiments addressing H/D exchange reactions:

(I. D_2/H_2) The reaction solution in the J. Young NMR tube pressurized with D_2 (see experimental description above) was degassed and the atmosphere was saturated with H_2 gas. The respective ^2H NMR spectra were recorded before and after the exchange of the atmosphere.

(II. methanol-d4) A *J. Young* NMR tube was charged with [({dppm}$_2$CH)RhCl$_3$]BAr^F (**6-BAr^F**, 30 mg, 0.016 mmol). The solid was subsequently dissolved in 0.7 mL CD_2Cl_2 to give a light orange solution. Three drops of CD_3OD from a *Pasteur* pipette were added to the solution and ^1H NMR spectrum was recorded. No H/D exchange was concluded by means of an observed change of the multiplicity or integral value of the ^1H resonances. Subsequently, 5µL of CD_3COOD was added to the mixture. A ^1H NMR spectrum was recorded after 12 h at ambient temperature. No H/D exchange was concluded by means of an observed change of the multiplicity or integral value of the ^1H resonances.

(III. CF_3COOD) A *J. Young* NMR tube was charged with [({dppm}C-H)RhCl$_3$]BArF ((**6-BAr^F** ,15 mg, 0.008 mmol). The solid was subsequently dissolved in 0.7 mL CD_2Cl_2 to give a light orange solution. 5µL of CF_3COOD was added to the solution and a ^1H NMR was recorded, which exhibits a significant reduction the integral value of the resonance associated with the protonated carbodiphosphoran moiety (-30% decrease). Therefore, all volatiles in the NMR tube were removed in *vacuo* and the solids were re-dissolved in CH_2Cl_2. Subsequently, a ^2H NMR spectrum was recorded indicating the presence of deuterium in the methylen bridges as well as in the C-*H* CDP moiety.

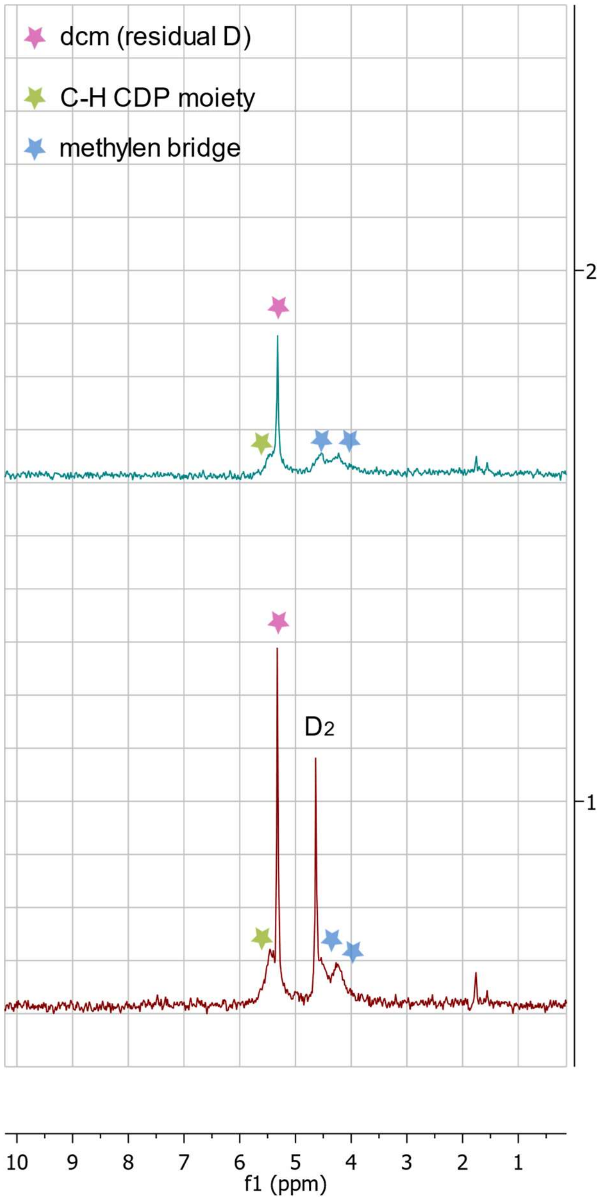

Figure S80 Stacked ^{2}H NMR spectra (77.10 MHz, CH_2Cl_2, 300 K) of the reaction of [({dppm}C)RhCl$_3$]BArF (**4-BArF**) + D_2 gas (bottom) and the same reaction mixture under H_2 atmosphere for 24 h (top).

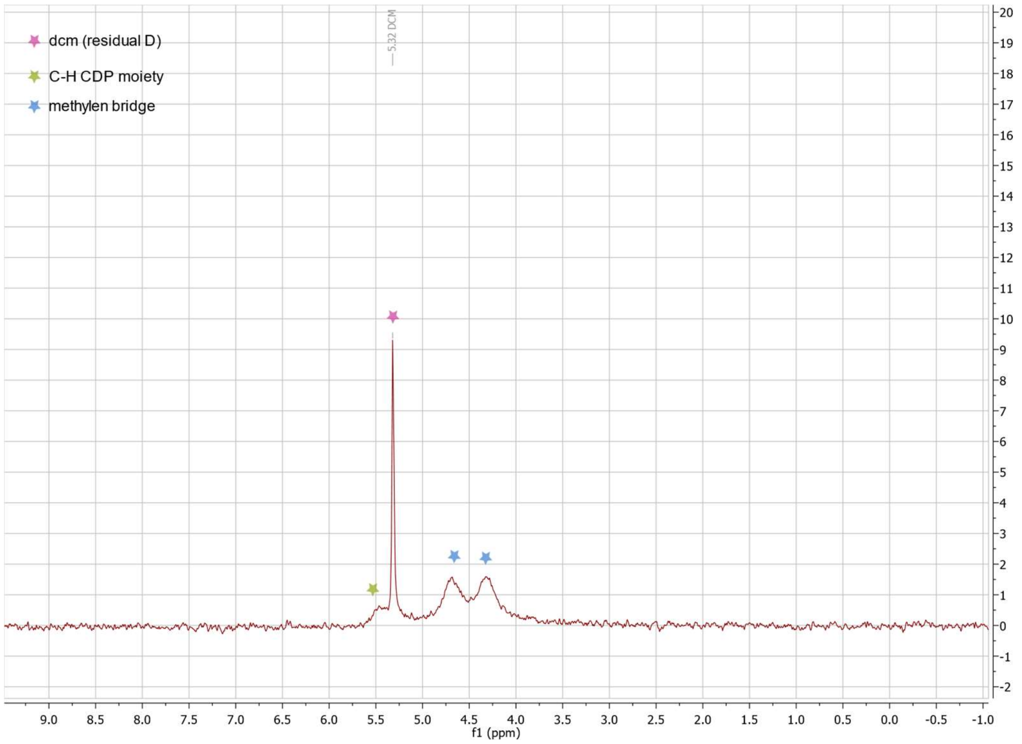

Figure S81 ^{2}H NMR spectrum (77.10 MHz, CH_2Cl_2, 300 K) of [({dppm}CH)RhCl$_3$]BArF (**6-BArF**) + CF_3COOD: H/D exchange giving rise to ^{2}H NMR resonances associated with the methylene bridge and C-H CDP moiety.

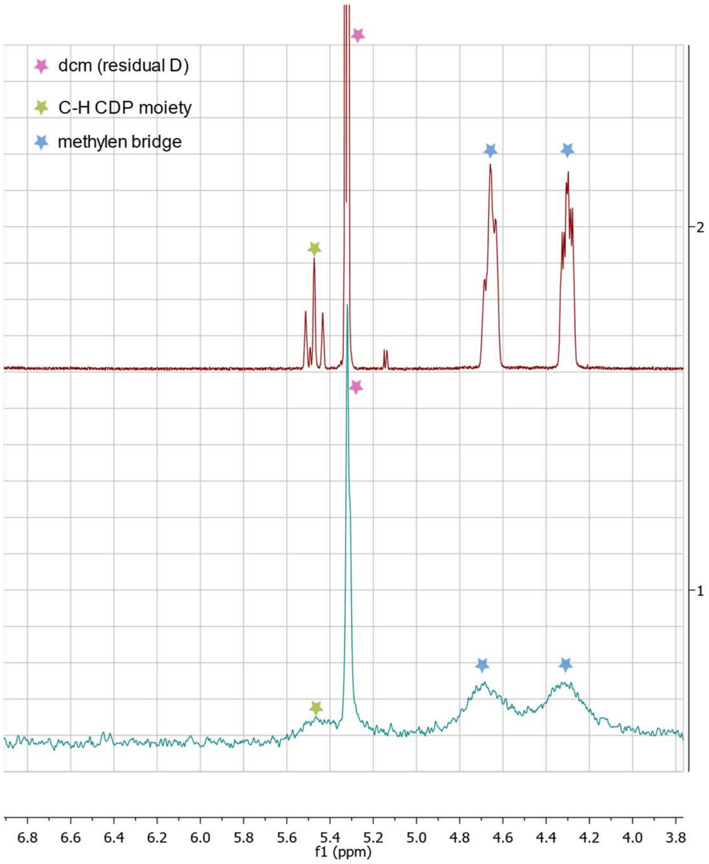

Figure S82 Stacked ^{1}H- (top) and ^{2}H NMR spectrum (bottom) (203.3 / 77.10 MHz, CH_2Cl_2, 300 K) of [({dppm}CH)RhCl$_3$]BArF (**6-BArF**) (top) + CF_3COOD (bottom).

15. Experiments addressing catalytic activity towards geminal dichlorides:

All substrates except dichloromethane were degassed applying three freeze-pump-thaw cycles and subsequently stored under argon atmosphere. Dichloromethane was prepared as stated above.

Standard procedure catalytic reactions.

All experiments were carried out under an protective argon atmosphere. The precatalyst (≈ 2.5 mol%) and lithium borohydride (2.2 eq.) were weighed into a Schlenk tube equipped with a magnetic stir bar inside of a Glovebox. The substrates (≈ 300 μmol) and THF-d_8 (1.85 ml) were added to a second Schlenk tube before being added entirely to the catalyst mixture. After adding the solvent mixture, the Schlenk tube was put into a preheated oil bath (50 °C) and left there stirring for the given duration. For NMR measurements the stated volumes of the reaction solution were sampled and transferred into a NMR tube and *m*-xylene was added as internal standard after the reaction. For quantification of the product the resonance of the quaternary carbon atoms of *m*-xylene (δ = 138.3 ppm) were utilized; the utilized resonances of the products are shown in the table below. Relative Yields are given as combined yields of all products in the sample.

Products were identified by comparison with literature spectra (reported in $CDCl_3$) as well as predicted spectra by MestReNova (see table below).

Deviations from the standard procedure

In case of the experiments where dichloromethane was used as substrate the precatalyst (approx. 2.5 mol%) and lithium borodeuteride (approx. 3 eq.; instead of lithium borohydride) were weighed in directly into a NMR tube and pyridine was used as solvent. Substrate and solvent were premixed in a Schlenk tube and 0.7 ml were sampled into the NMR tube. The tube was subsequently flame sealed and left to warm up to ambient temperature. Subsequently, the reaction solution was put into a preheated oil bath (50 °C) and left there to react for the given duration.

Table S14 Utilized product resonances for the quantification via quantitative $^{13}C\{^1H\}$ NMR spectroscopy in thf-d_8.

Molecule	carbon atom	chemical shift δ [ppm] (*literature ref. in $CDCl_3$*)[a]
tetraphenylethylene	(Ph_2-***C***=***C***-Ph_2)	144.8 (*144*)
chlorodiphenylmethane	C_{quart}	142.8 (*141*)
1,2-dichlorotetraphenylethane	C_{quart}	144.0 (simulated spectra)
1,1,2,2-tetraphenylethane	C_{quart}	142.6 (144)
1,2-dichloro-1,2-diphenylethane	C_{quart}	139.3 (*138*)
benzyl chloride	C_{quart}	140.1 (*138*)
toluene	C_{quart}	138.5 (*138*)
dibenzyl	C_{quart}	142.7 (*142)*
2-chlorobutane	(CH_3-***C***ClH-C_2H_5)	61.2 (*60, in 1,4-dioxane)*
3,4-dichloro-3,4-dimethylhexane	C_{quart}	72.7 (simulated spectra)
2-chloropropane	(CH_3-***C***ClH-CH_3)	54.8 (*53*)

a) M. I. Watkins, G. A. Olah, *J. Am. Chem. Soc.* **1981**, *103*, 6566; S. Rej, S. Pramanik, H. Tsurugi, K. Mashima, *Chem. Commun.* **2017**, *53*, 13157; W. Liu, F. Hou, *Tetrahedron* **2017**, *73*, 931; Y. Kamada, Y. Kitamura, T. Tanaka, T. Yoshimitsu, *Org. Biomol. Chem.* **2013**, *11*, 1598; G. Hua, Y. Li, A. M. Z. Slawin, J. D. Woollins, *Dalton transactions (Cambridge, England : 2003)* **2007**, 1477; J. Elguero, R. M. Claramunt, R. Garcerán, S. Julià, L. Avila, J. M. Del Mazo, *Magnetic Reson in Chemistry* **1987**, *25*, 260; A. Ejchart, *Org. Magn. Reson.* **1981**, *15*, 22.

A) Experiments with [({dppm}$_2$C)RhCl] (1) as catalyst

(I. dichlorodiphenyl methane (DCDPM)) 103.5 mg (436.5 µmol) DCDPM were dissolved in 1.85 mL THF-d_8 and subsequently added to a mixture of 9.6 mg (10.4 µmol, 2.39 mol%) [({dppm}$_2$C)RhCl] (**1**) and 20.6 mg (945.6 µmol, 2.2 equiv.) lithium borohydride, resulting in a dark brown solution and excessive gas evolution. The reaction solution was immediately heated to 50°C. After 24 hours 0.65 mL of the mixture were sampled for quantitative $^{13}C\{^1H\}$ NMR measurement and 9.3 mg (87.6 µmol) *m*-xylene were added for quantification of tetraphenyl ethylene (TPE). Conversion (DCDPM): 100%, yield (TPE): 71.8 µmol (93.7 %).

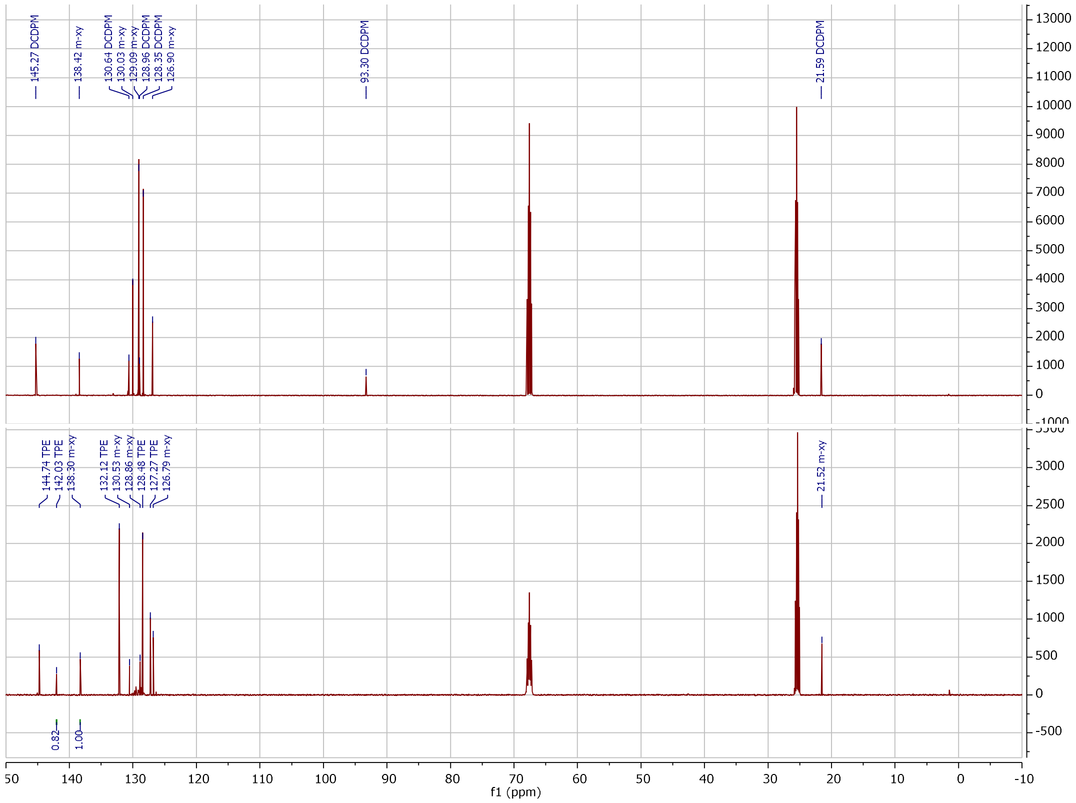

Figure S83 Stacked quantitative $^{13}C\{^{1}H\}$ NMR spectra (126.3 MHz, THF-d_8, 300 K) before the reaction (top) and after 24 h at 50°C (bottom).

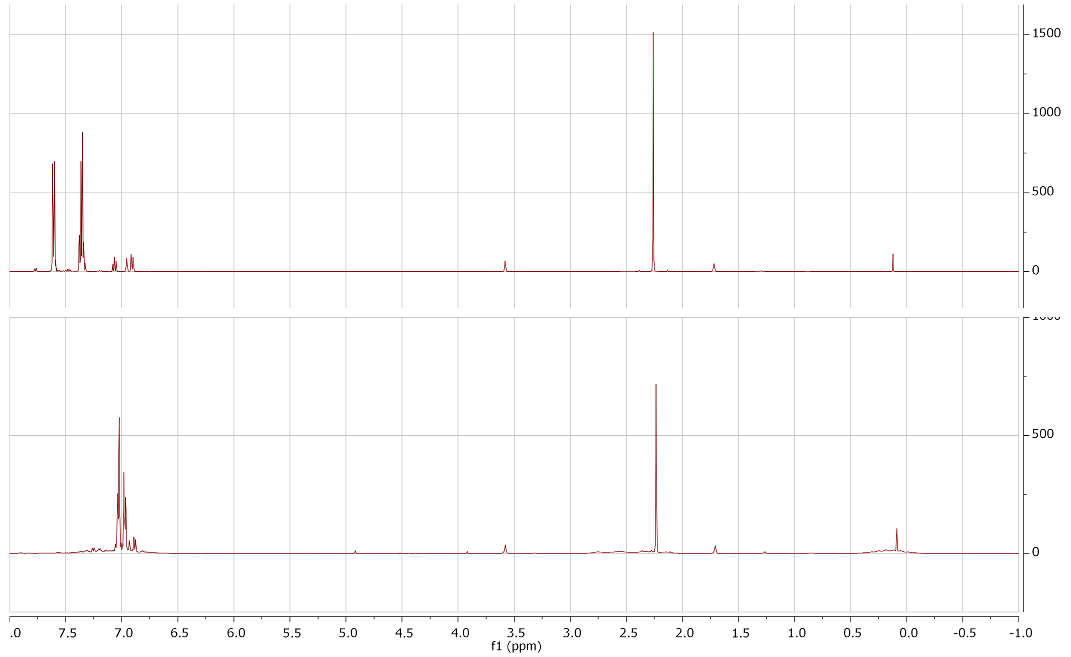

Figure S84 Stacked ^{1}H NMR spectrum (502.29 MHz, THF-d_8, 300 K) before the reaction (top) and after 24 h at 50°C (bottom).

(II. benzal chloride (BC)) 66.3 mg (411.7 µmol) BC were dissolved in 1.85 mL THF-d_8 and subsequently added to a mixture of 9.5 mg (10.3 µmol, 2.51 mol%) [({dppm}$_2$C)RhCl] (**1**) and 19.8 mg (908.9 µmol, 2.2 equiv.) lithium borohydride, resulting in a dark brown solution and excessive gas evolution. The reaction solution was immediately heated to 50°C. After 24 hours 0.65 mL were withdrawn from the solution for quantitative $^{13}C\{^1H\}$ NMR measurement and 14.6 mg (137.5 µmol) *m*-xylene were added for the quantification of the products. Conversion (BC): 100%, yield: 103.4 µmol of a mixture of three products (95.0%). The mixture of products was distributed as follows:

1,2-dicholoro-1,2-diphenyl ethane	37.125 µmol
benzyl chloride	38.500 µmol
toluene	24.750 µmol

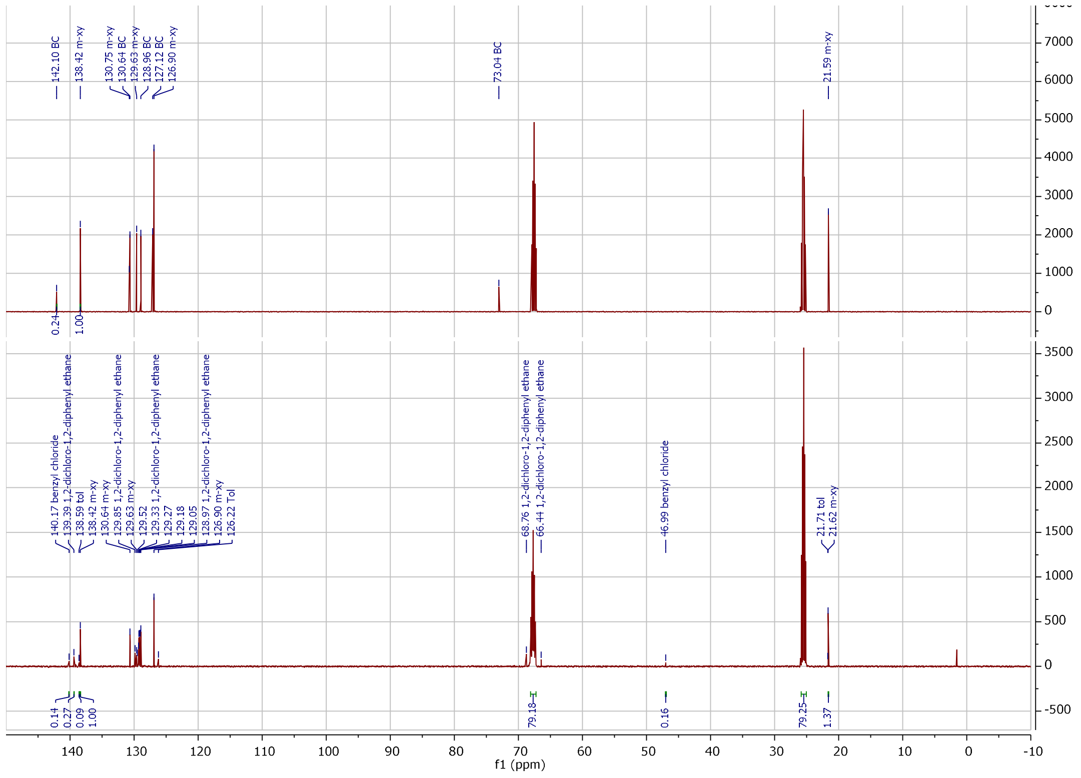

Figure S85 Stacked quantitative $^{13}C\{^1H\}$ NMR spectra (126.3 MHz, THF-d_8, 300 K) before the reaction (top) and after 24 h at 50°C (bottom).

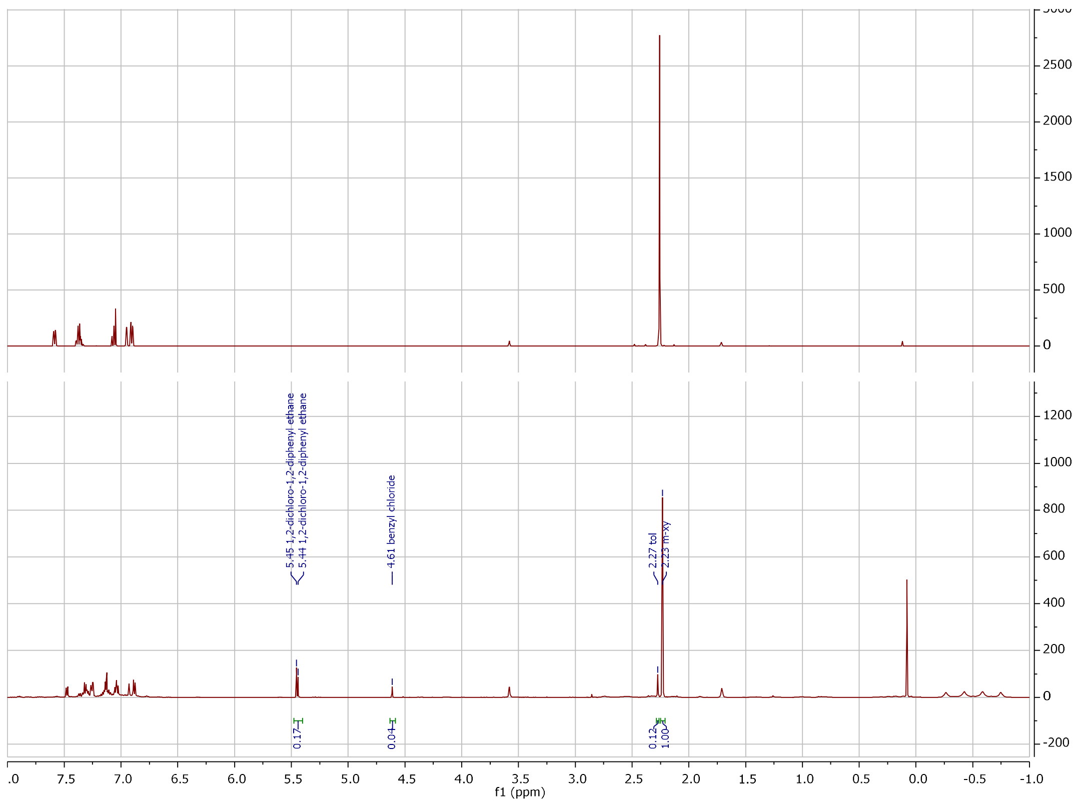

Figure S86 Stacked ^{1}H NMR spectrum (502.29 MHz, THF-d_8, 300 K) before the reaction (top) and after 24 h at 50°C (bottom).

(III. 2,2-dichlorobutane (DCB)) 54.1 mg (425.9 µmol) DCB were dissolved in 1.85 mL THF-d_8 and subsequently added to a mixture of 10.0 mg (10.9 µmol, 2.50 mol%) [({dppm}$_2$C)RhCl] (**1**) and 20.9 mg (959.4 µmol, 2.25 equiv.) lithium borohydride, resulting in a dark brown solution and observable gas evolution. The reaction solution was immediately heated to 50°C. After 20 hours and 90 hours, respectively, 0.65 mL were withdrawn from the solution for quantitative $^{13}C\{^1H\}$ NMR measurement and 18.6 mg (124.6 µmol) and 1.2 mg (11.3 µmol) *m*-xylene were added for the quantification of the products. **20h:** conversion (DCB): 51.8 %, yield (2-chlorobutane): 74.80 µmol (50.0%). **92h:** conversion (DCB): 100%, yield: 142.61 µmol of a mixture of two products (97.6%). The mixture of products was distributed as follows:

92h

3,4-dichloro-3,4-dimethyl hexane	3.390 µmol
2-chlorobutane	139.216 µmol

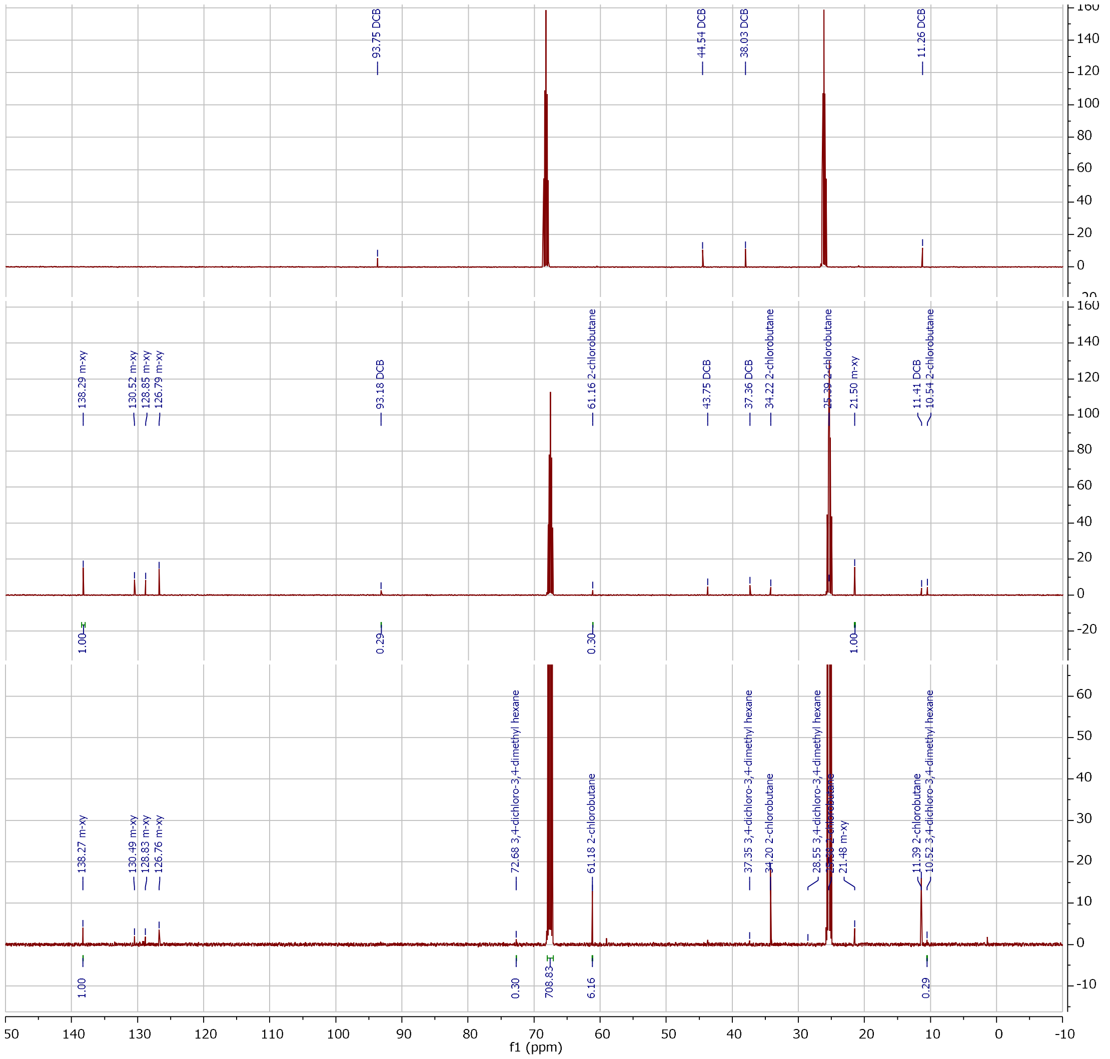

Figure S87 Stacked quantitative $^{13}C\{^1H\}$ NMR spectra (126.3 MHz, THF-d_8, 300 K) before the reaction (top, without *m*-xylene as internal standard) after 20 h (mid) and 92 h (bottom) at 50°C.

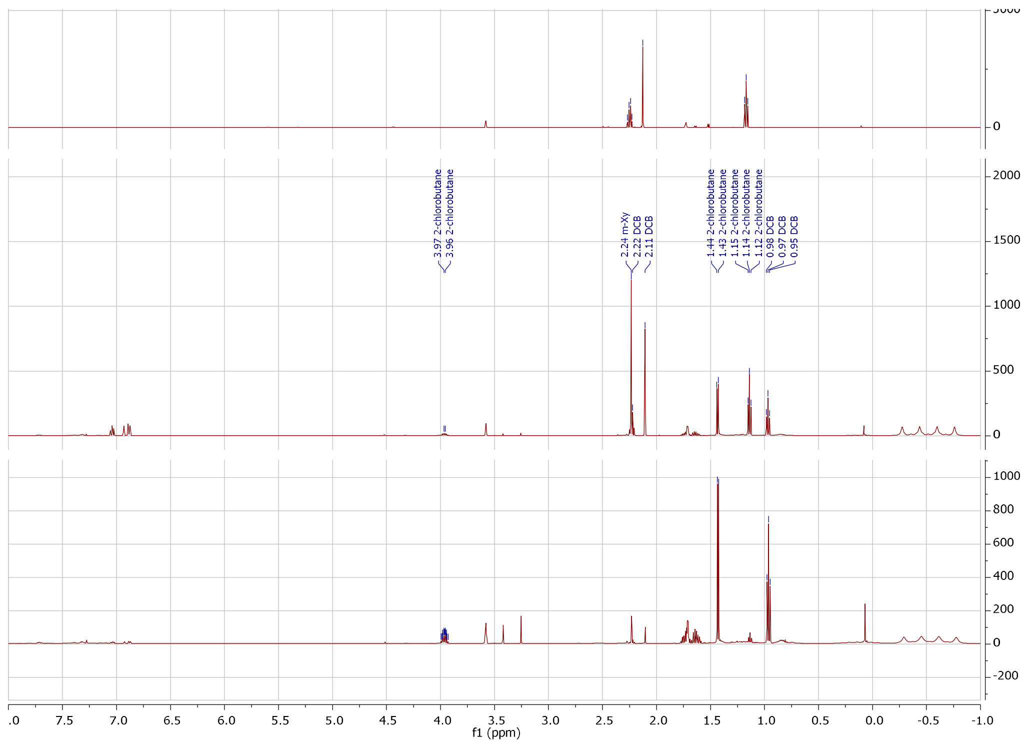

Figure S88 Stacked ^{1}H NMR spectrum (502.29 MHz, THF-d_8, 300 K) before the reaction (top, without *m*-xylene as internal standard), after 20 h (mid) and 92 h (bottom) at 50°C.

(IV.a dichloromethane (DCM) in THF) 20.7 mg (243.8 µmol) DCM were dissolved in 1.4 mL THF and 0.7 mL of the solution were subsequently added to a mixture of 3.4 mg (3.7 µmol, 4.10 mol%) [({dppm}$_2$C)RhCl] (**1**) and 8.4 mg (325.5 µmol, 2.7 equiv.) lithium borodeuteride in a NMR tube, leading to a dark brown solution and observable gas evolution. The solution was frozen and the tube was flame sealed under reduced pressure. Subsequently, the NMR tube was slowly warmed to ambient temperature and subsequently heated to 50°C. After 24 hours ^{2}H and ^{13}C{^{1}H} NMR spectra were measured. Conversion was incomplete and yield was not determined.

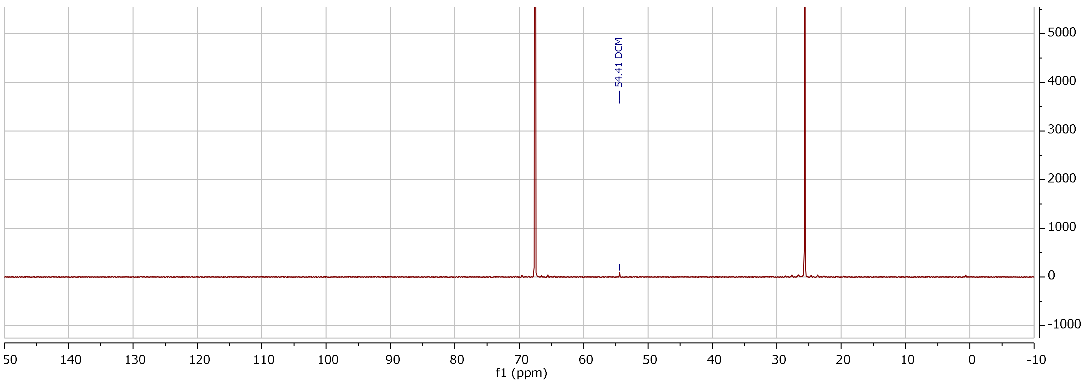

Figure S89 ^{13}C{^{1}H} NMR spectrum (126.3 MHz, THF-H_8, 300 K) after 24 h at 50°C.

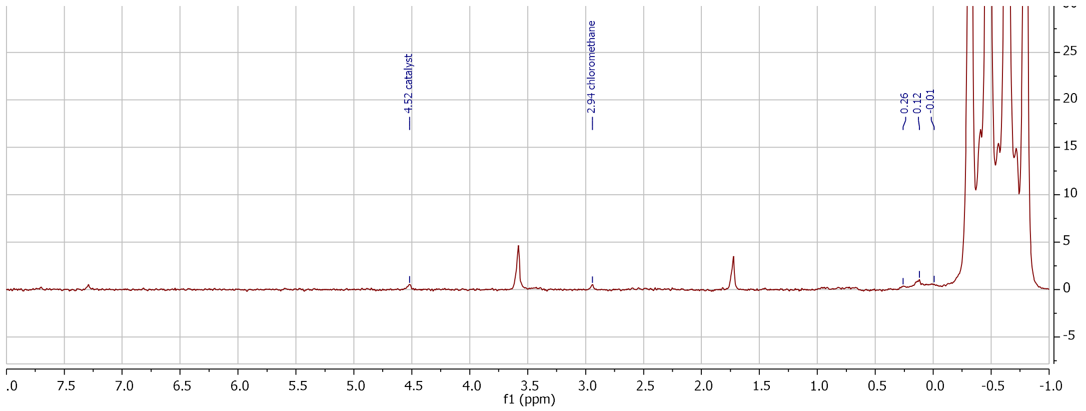

Figure S90 ^{2}H NMR spectrum (77.10 MHz, THF-H_8, 300 K) before after 24 h at 50°C.

(IV.b dichloromethane (DCM) in pyridine) 40.7 mg (479.2 µmol) DCM were dissolved in 2.8 mL pyridine and 0.7 mL of the solution were subsequently added to a mixture of 3.4 mg (3.7 µmol, 4.17 mol%) [({dppm}$_2$C)RhCl] (**1**) and 8.4 mg (325.5 µmol, 2.7 equiv.) lithium borodeuteride in a NMR tube, leading to a dark red solution and observable gas evolution. The solution was frozen and the tube flame sealed under reduced pressure. Subsequently, the NMR tube was slowly warmed to ambient temperature and subsequently heated to 50°C. After 24 hours ^{2}H and ^{13}C{^{1}H} NMR spectra were measured. Conversion (DCM): 100%, yield was not determined.

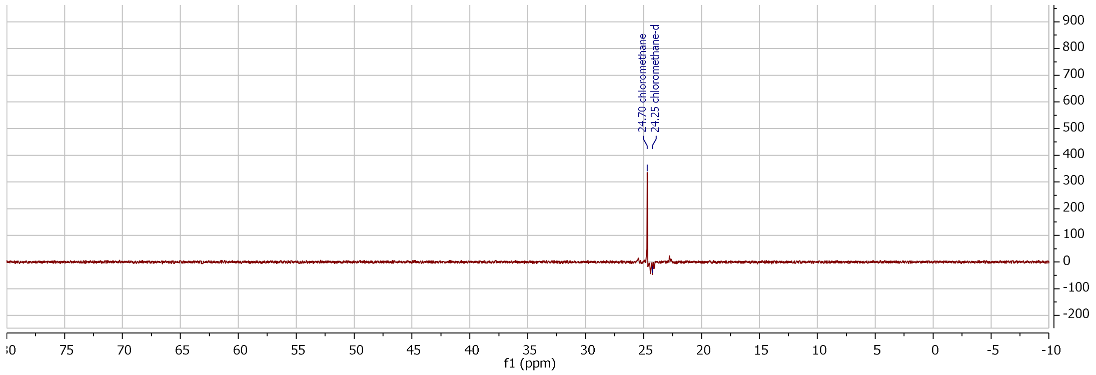

Figure S91 ^{13}C APT NMR spectrum (126.3 MHz, pyridine-H_5, 300 K) after 24 h at 50°C.

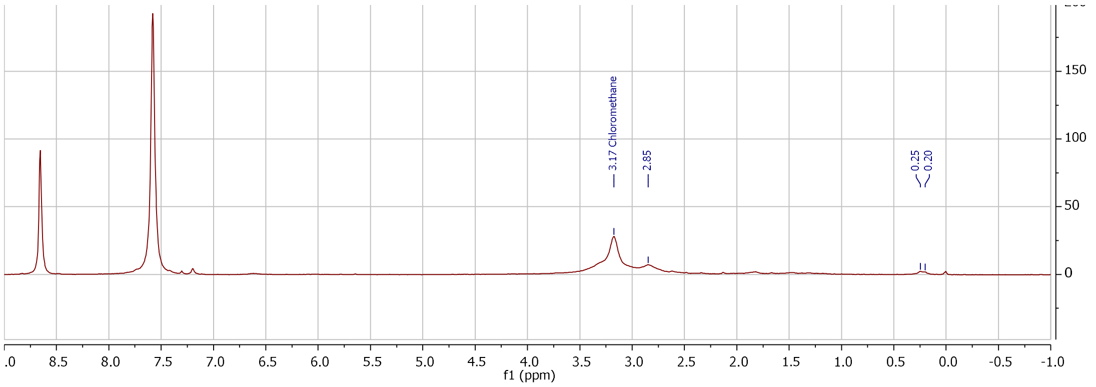

Figure S92 ^{2}H NMR spectrum (77.10 MHz, pyridine-H_5, 300 K) after 24 h at 50°C.

(V. 2,2-dichloropropane (DCP)) 37.7 mg (333.4 µmol) 2,2-dichloropropane was dissolved in 1.85 mL THF-d_8. A mixture of 7.5 mg (8.2 µmol; 2.45 mol%) [({dppm}$_2$C)RhCl] **1** and 15.8 mg (725.3 µmol; 2.2 equiv.) lithium borohydride was added into a second Schlenk tube. The solution was entirely transferred to the solid mixture, leading to visible gas evolution and a dark brown solution. The reaction mixture was immediately heated to 50°C. After 24 hours 0.65 mL were withdrawn from the solution for quantitative $^{13}C\{^1H\}$-NMR measurements. 6.5 mg (102.7 µmol) *m*-xylene was added after 24 hours for the quantification of the product(s). **24h**: conversion (DCP): 81.2 %, yield (2-chloropropane): 71.0 µmol (60.6 %).

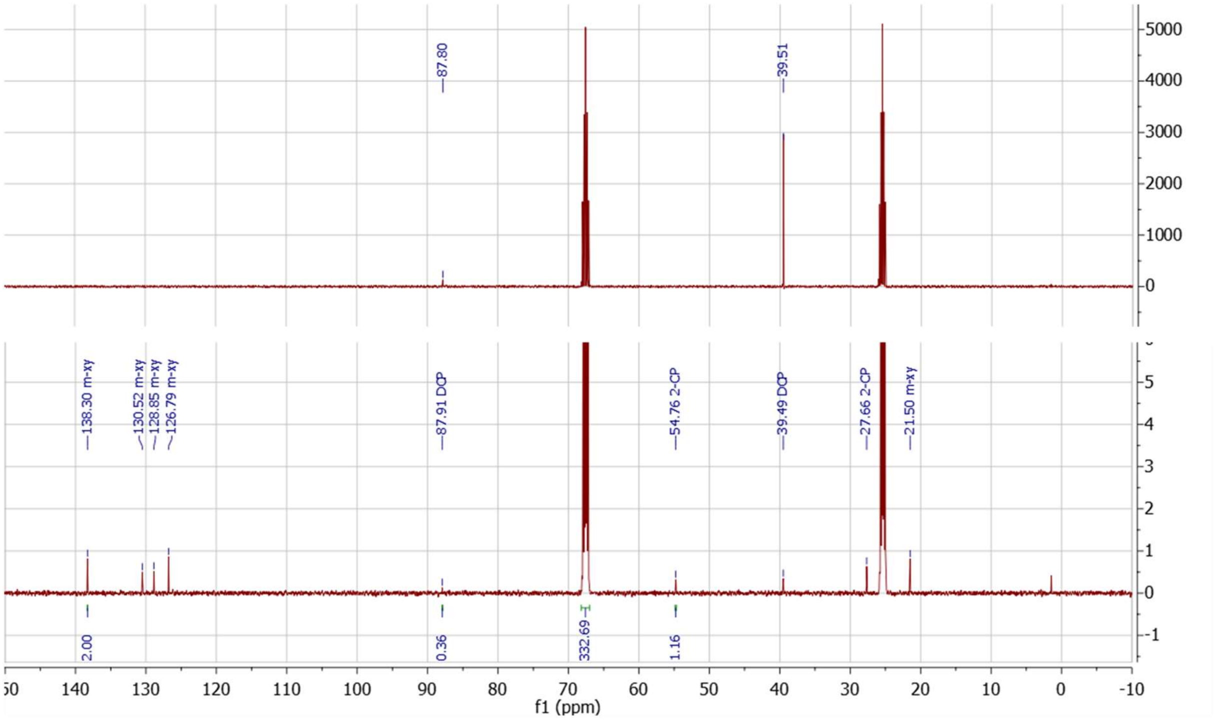

Figure S93 Stacked quantitative ^{13}C NMR spectra (126.3 MHz, THF-d_8, 300 K) before the reaction (top, without *m*-xylene as internal standard) after 24 h (bottom) at 50°C.

B) Experiments under similar conditions as A) without [({dppm}$_2$C)RhCl] (1)

(I. dichlorodiphenyl methane (DCDPM)) 101.7 mg (428.8 µmol) DCDPM were dissolved in 1.85 mL THF-d_8 and subsequently added to 20.6 mg (945.6 µmol, 2.2 equiv.) lithium borohydride, resulting in a colourless solution with residual lithium borohydride. The reaction solution was heated to 50°C immediately. After 24 hours 0.65 mL were withdrawn from the solution for quantitative $^{13}C\{^1H\}$ NMR measurement and 6.7 mg (63.1 µmol) *m*-xylene were added for quantification of DCDPM and chlorodiphenyl methane (CDPM). Conversion (DCDPM): 57.8%, yield (CDPM): 86.5 µmol (57.3 %).

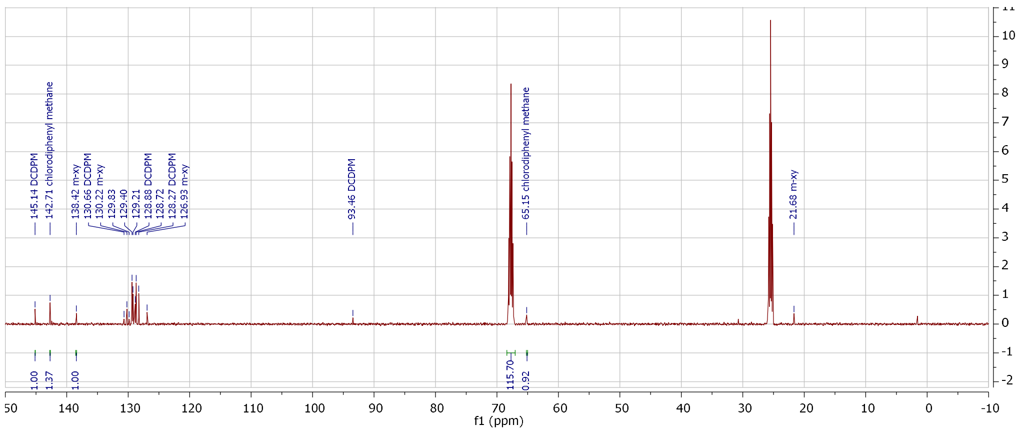

Figure S94 Quantitative ^{13}C NMR spectrum (126.3 MHz, THF-d_8, 300 K) after 24 h at 50°C.

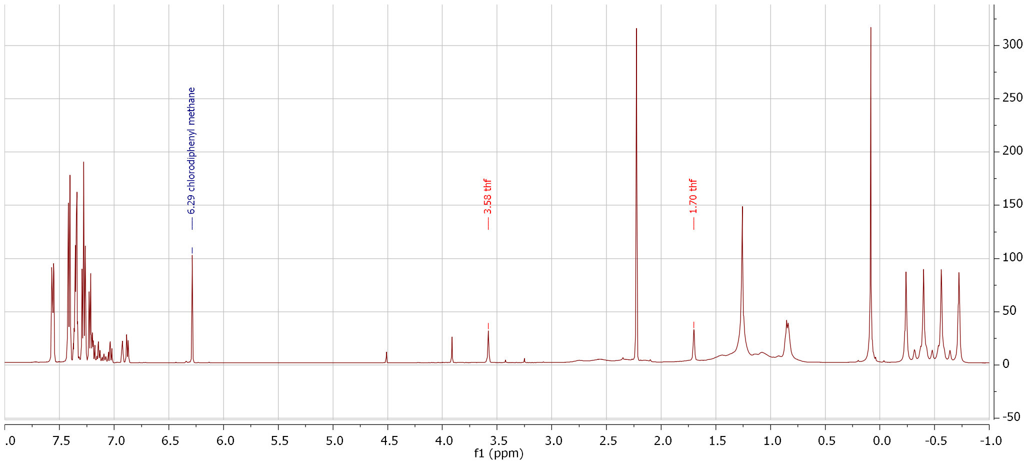

Figure S95 ^1H NMR spectrum (502.29 MHz, THF-d_8, 300 K) after 24 h at 50°C.

(II. benzal chloride (BC)) 54.1 mg (335.9 µmol) BC were dissolved in 1.85 mL THF-d_8 and subsequently added to 19.8 mg (908.9 µmol, 2.7 equiv.) lithium borohydride, resulting in a colourless solution with residual lithium borohydride. The reaction solution was heated to 50°C immediately. After 24 hours 0.65 mL were withdrawn from the solution for quantitative $^{13}C\{^1H\}$ NMR measurement and 11.4 mg (107.4 µmol) *m*-xylene were added for the quantification of the product(s). Conversion (BC): 0%, yield: 0%.

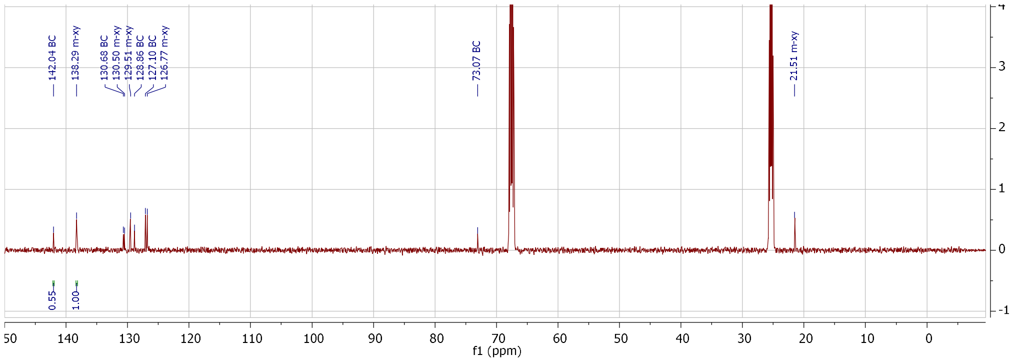

Figure S96 Quantitative ^{13}C NMR spectrum (126.3 MHz, THF-d_8, 300 K) after 24 h at 50°C.

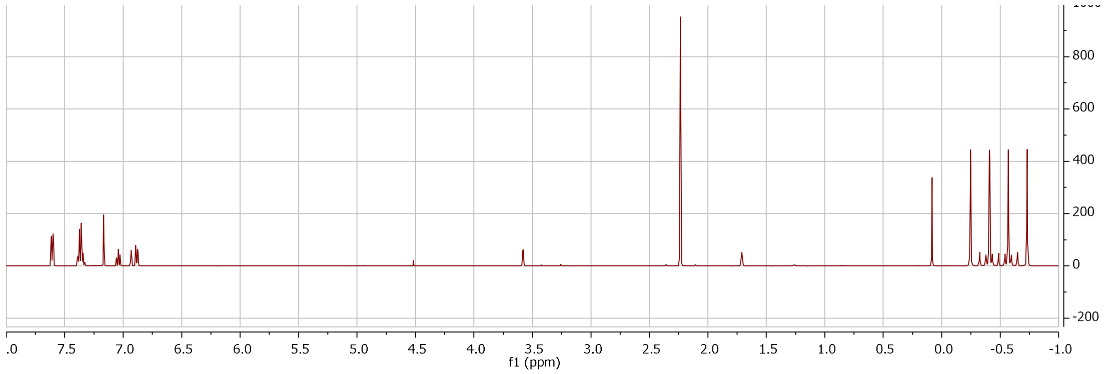

Figure S97 ^{1}H NMR spectrum (502.29 MHz, THF-d_8, 300 K) after 24 h at 50°C.

(III. 2,2-dichlorobutane (DCB)) 55.1 mg (433.5 µmol) DCB were dissolved in 1.85 mL THF-d_8 and subsequently added to 20.9 mg (959.4 µmol, 2.2 equiv.) lithium borohydride, resulting in a colourless solution with residual lithium borohydride. The reaction solution was heated to 50°C immediately. After 24 hours 0.65 mL were withdrawn from the solution for quantitative ^{13}C NMR measurement and 17.2 mg (117.7 µmol) *m*-xylene were added for the quantification of the products. Conversion (DCB): 0 %, yield: 0%.

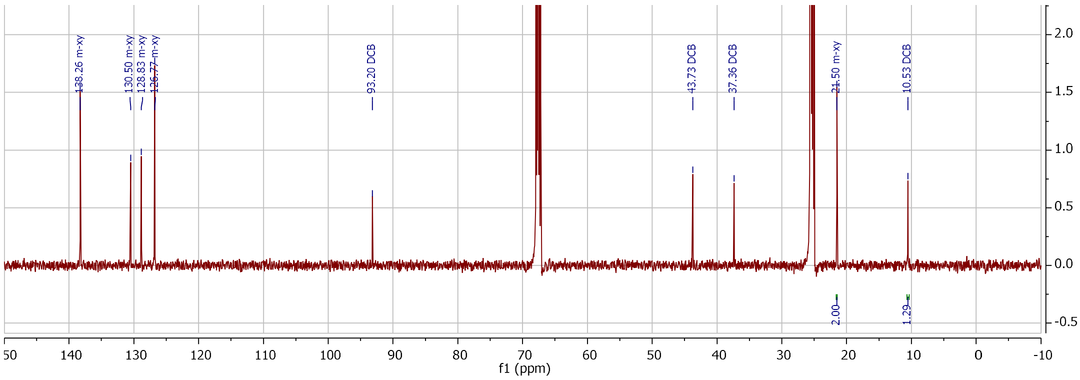

Figure S98 Quantitative ^{13}C NMR spectrum (126.3 MHz, THF-d_8, 300 K) after 24 h at 50°C.

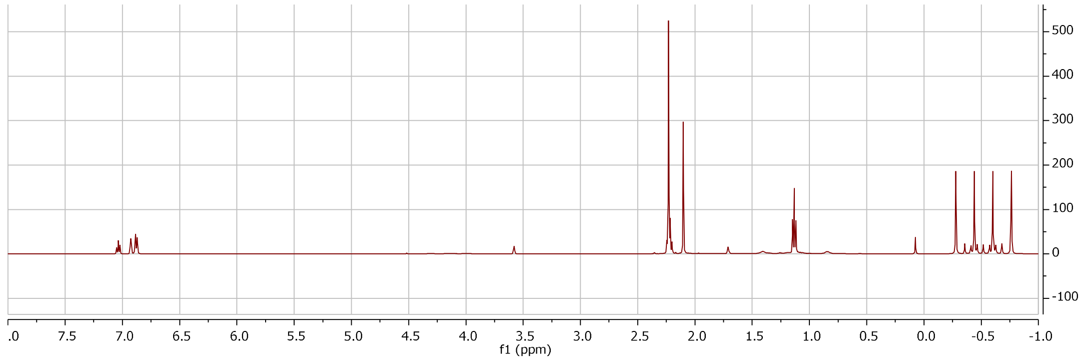

Figure S99 ^{1}H NMR spectrum (502.29 MHz, THF-d_8, 300 K) after 24 h at 50°C.

(IV. dichloromethane (DCM) in pyridine) 26.0 mg (306.2 µmol) DCM were dissolved in 1.4 mL pyridine and 0.7 mL of the solution were subsequently added to 8.8 mg (341.0 µmol, 2.2 equiv.) lithium borodeuteride in a NMR tube, leading to a colourless solution with residual lithium borodeuteride. The solution was frozen and the tube flame sealed under reduced pressure. Subsequently, the NMR tube was slowly warmed to ambient temperature and heated to 50°C. After 24 hours ^{2}H and $^{13}C\{^{1}H\}$ NMR spectra were measured. No reaction of DCM was observed.

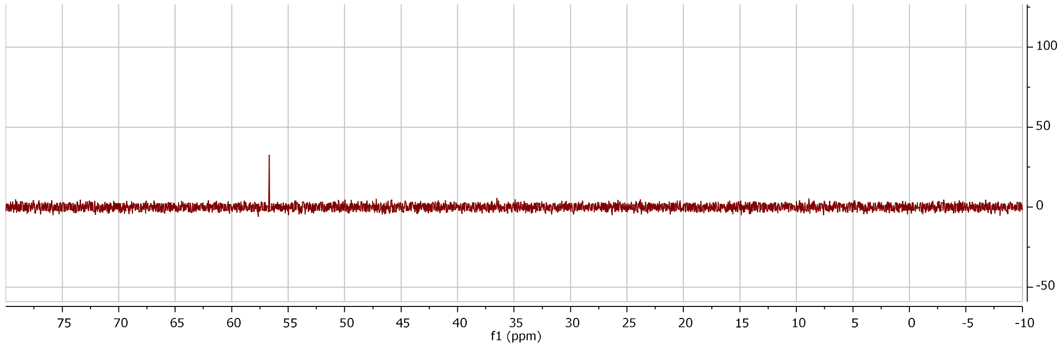

Figure S100 ^{13}C APT NMR spectrum (126.3 MHz, pyridine-H_5, 300 K) after 24 h at 50°C.

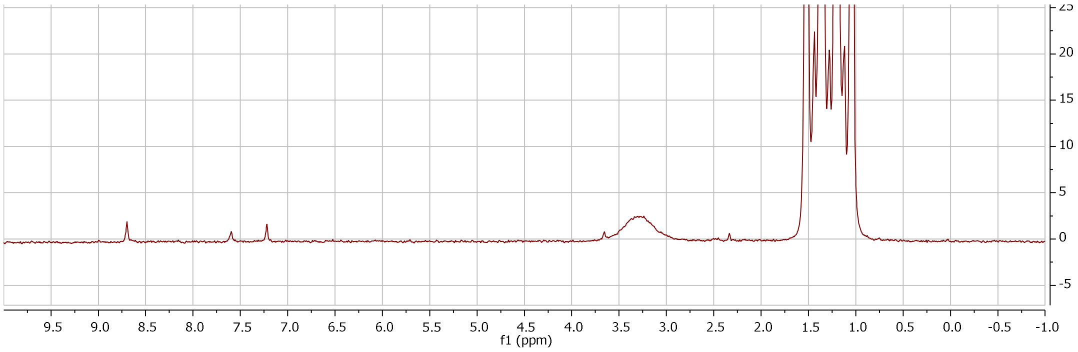

Figure S101 ^{2}H NMR spectrum (77.10 MHz, pyridine-H_5, 300 K) after 24 h at 50°C.

C) Experiments with [({dppm}$_2$C)RhCl] (1) as potential catalyst for C-F bond activation

(I. chlorofluoromethane) 1.8 mg (1.9 µmol) [({dppm}$_2$C)RhCl] (**1**) and 4.0 mg (153.2 µmol) lithium borodeuteride were dissolved in 1.0 mL 1,2-dimethoxyethane and stirred at ambient temperature for two minutes. Subsequently 12.0 mg (82.4 µmol) (trifluoromethyl)benzene and 15.5 mg (184.2 µmol) benzene-d_6 were added. The solution was transferred immediately into a flame sealable NMR tube, frozen with liquid nitrogen and reduced pressure was applied. After warming up to ambient temperature under reduced pressure, the reaction vessel was flushed with chlorofluoromethane, the solution was frozen using liquid nitrogen and the tube was subsequently flame sealed. The sealed NMR tube was slowly warmed to ambient temperature, before being heated at 140°C for 40 hours. The conversion of $LiBD_4$ (85%) was determined by integration of the resonance for $LiBD_4$ in the ^{2}H NMR spectrum relative to the integral of C_6D_6 before and after the reaction. The yields of deuterated products were estimated by the following method: Based on the integration of resonances for CH_2D_2 and CH_2DF relative to the internal standard C_6D_6 in the ^{2}H NMR spectra were used to estimate the amount of products in solution. As an approximation we used Henry's constant for CH_4 in EtOH (H^{px} = 686 bar)[31–33] and calculated the approx. amount of CH_2D_2 and CH_2DF in the gas phase, assuming an ideal gas.

Table 15 Approximated amounts of products in solution and in the gas phase for the reaction of CH_2ClF.

Product	$n_{solution}$ / µmol	n_{gas_phase} / µmol	n_{sum} / µmol	TON
CH_2DF	31.3	268.3	299.6	158
CH_2D_2	13.8	118.3	132.1	70

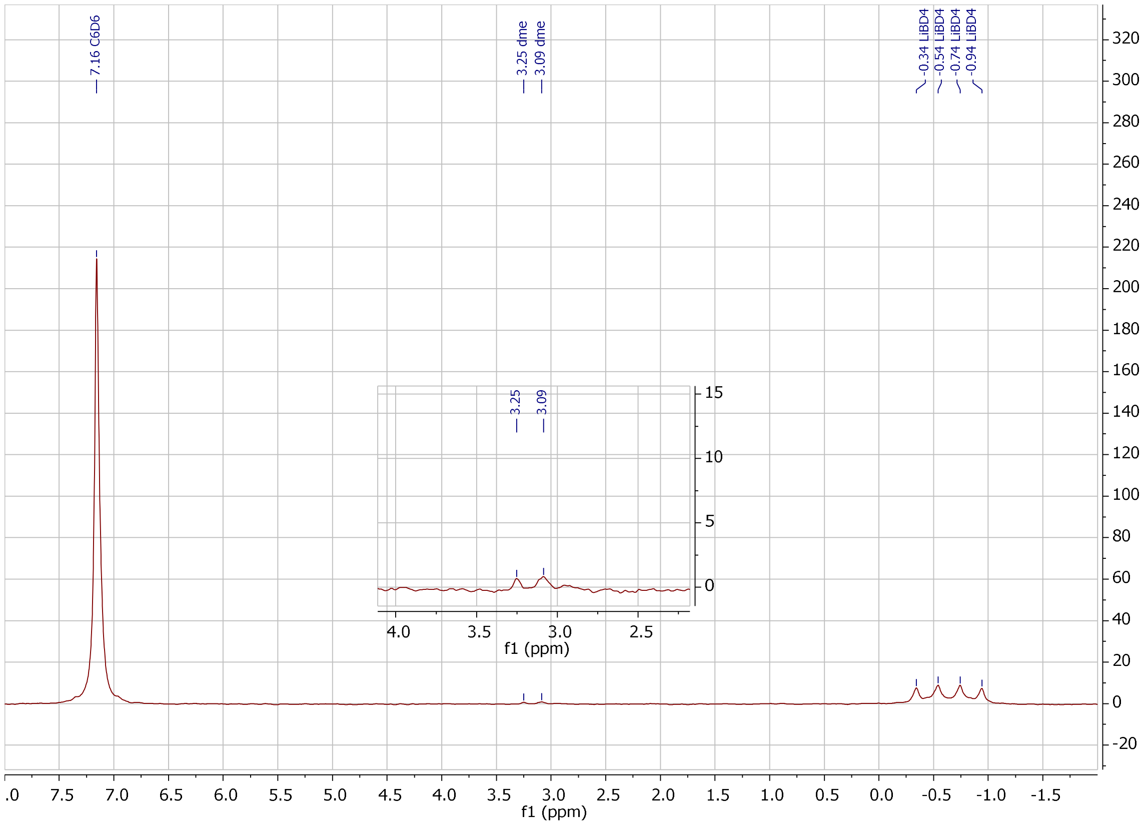

Figure S102 ^{2}H NMR spectrum (67.75 MHz, 1,2-dimethoxyethane, 300 K) of the reaction solution before the reaction.

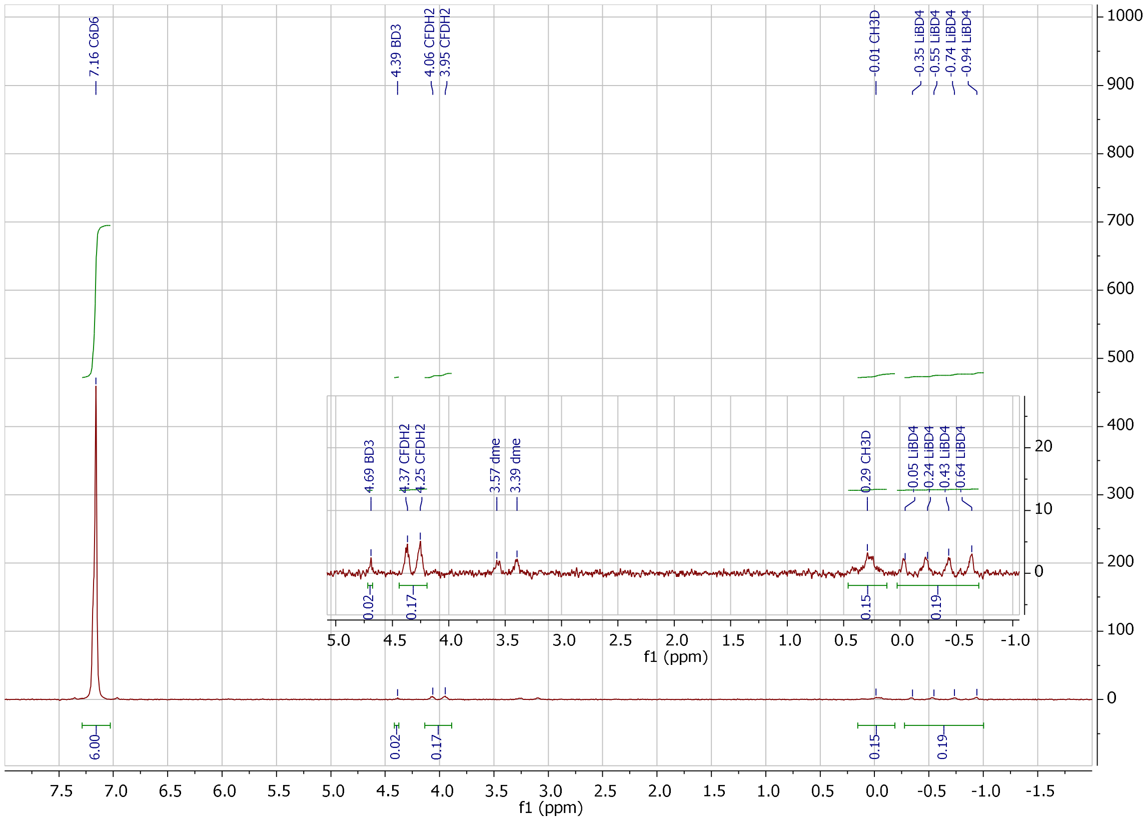

Figure S103 Quantitative ^{2}H NMR spectrum (67.75 MHz, 1,2-dimethoxyethane, 300 K) of the reaction solution after 40h at 140°C.

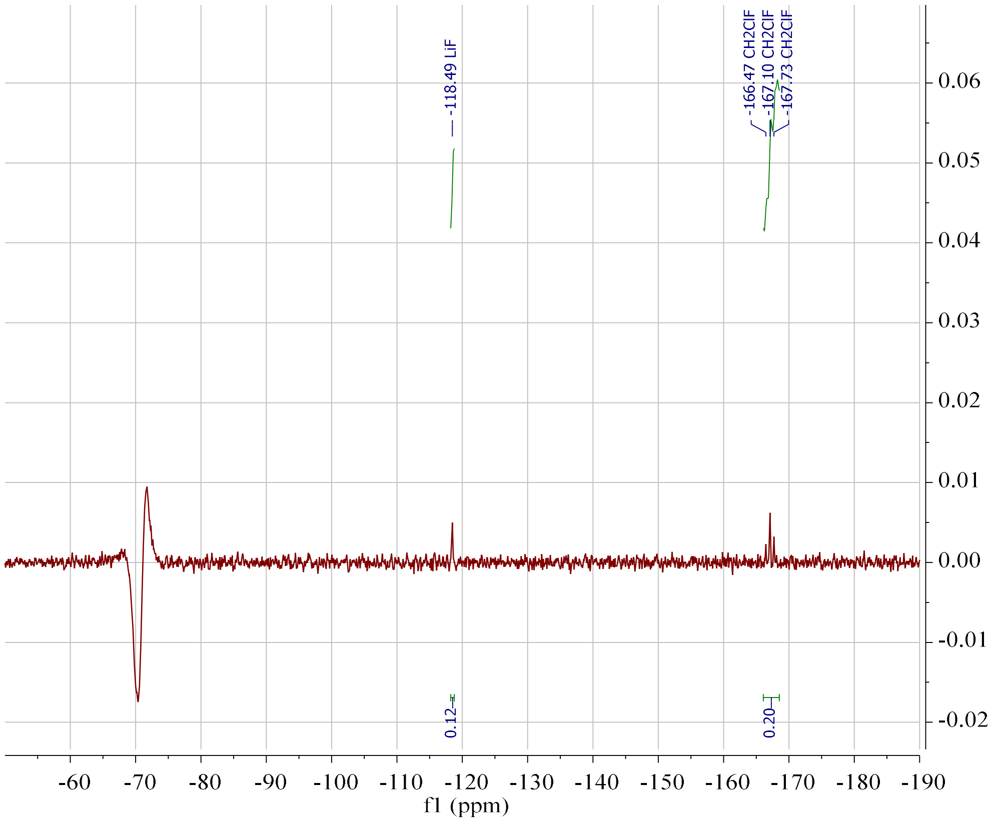

Figure S104 ^{19}F NMR spectrum (75 MHz, H_2O, 300 K) of the aqueous extract solution of the formed solids after 40h at 140°C LiF formation.

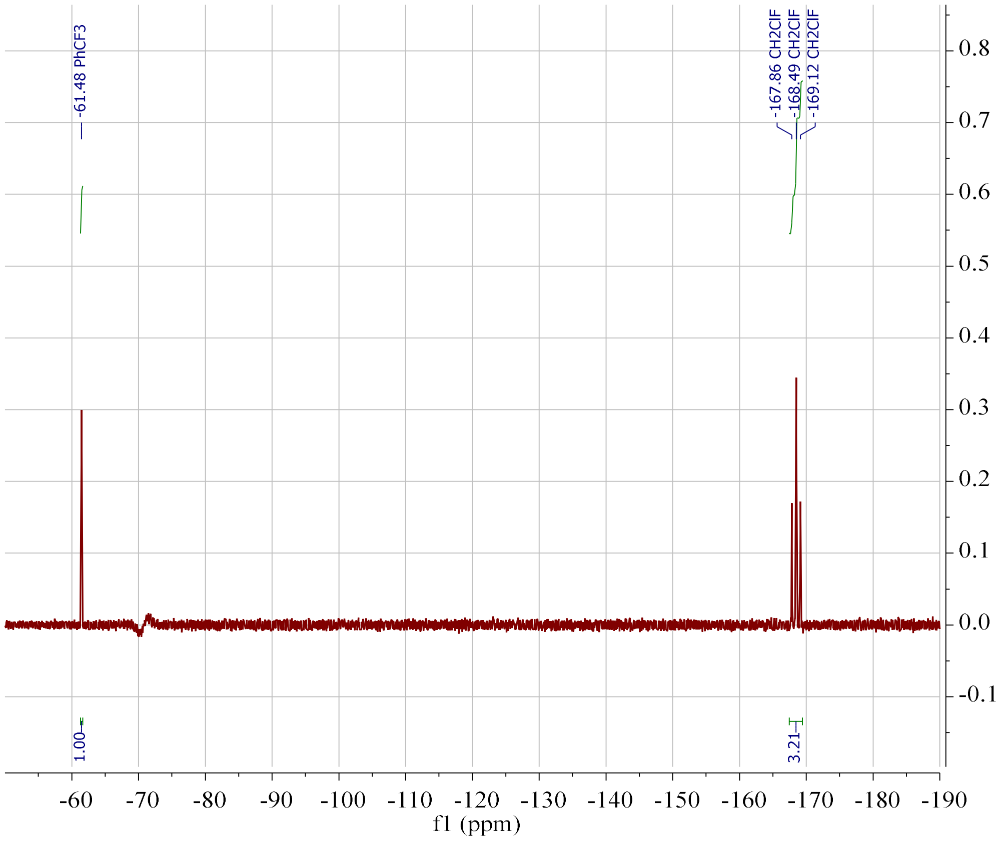

Figure S105 ^{19}F NMR spectrum (75 MHz, 1,2-dimethoxyethane, 300 K) of the reaction solution after 40h at 140°C between -50 ppm and -190 ppm.

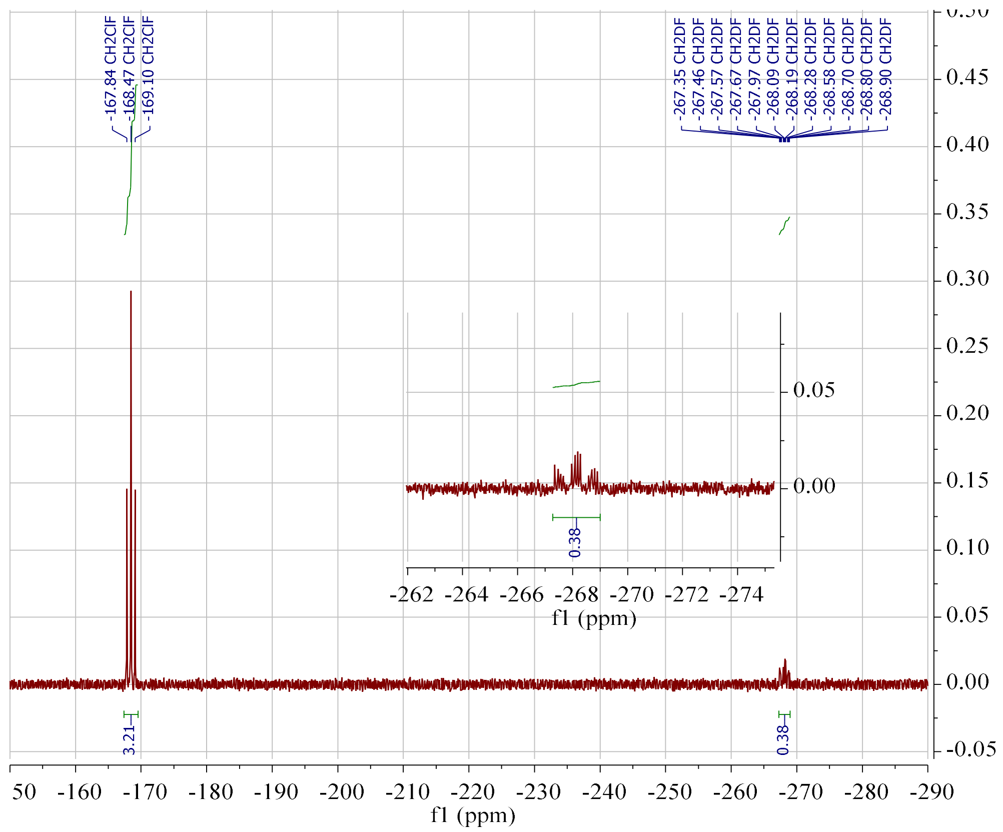

Figure S106 ^{19}F NMR spectrum (75 MHz, 1,2-dimethoxyethane, 300 K) of the reaction solution after 40h at 140°C between -150 ppm and -290 ppm.

16. References

[1] K. Sommer, *Zeitschrift für Anorg. und Allg. Chemie* **1970**, *376*, 37–43.

[2] C. Reitsamer, S. Stallinger, W. Schuh, H. Kopacka, K. Wurst, D. Obendorf, P. Peringer, *Dalt. Trans.* **2012**, *41*, 3503–3514.

[3] N. A. Yakelis, R. G. Bergman, *Organometallic* **2005**, *24*, 3579–3581.

[4] U. Jahn, P. Hartmann, I. Dix, P. G. Jones, *European J. Org. Chem.* **2001**, 3333–3355.

[5] R. Bozak, *J. Chem. Educ.* **1966**, *43*, 73.

[6] N. G. Connelly, W. E. Geiger, *Chem. Rev.* **1996**, *96*, 877–910.

[7] D. F. Evans, *J. Chem. Soc.* **1959**, 2003–2005.

[8] C. G. Lin, M. Hutin, C. Busche, N. L. Bell, D. L. Long, L. Cronin, *Dalt. Trans.* **2021**, *50*, 2350–2353.

[9] O. V. Dolomanov, L. J. Bourhis, R. J. Gildea, J. A. K. Howard, H. Puschmann, *J. Appl. Crystallogr.* **2009**, *42*, 339–341.

[10] G. M. Sheldrick, *Acta Crystallogr. Sect. A Found. Crystallogr.* **2008**, *64*, 112–122.

[11] L. J. Bourhis, O. V. Dolomanov, R. J. Gildea, J. A. K. Howard, H. Puschmann, *Acta Crystallogr. Sect. A Found. Crystallogr.* **2015**, *71*, 59–75.

[12] G. M. Sheldrick, *Acta Crystallogr. Sect. A Found. Crystallogr.* **2015**, *71*, 3–8.

[13] E. L. Hahn, *Phys. Rev.* **1950**, *80*, 580–594.

[14] J. M. Fauth, A. Schweiger, L. Braunschweiler, J. Forrer, R. R. Ernst, *J. Magn. Reson.* **1986**, *66*, 74–85.

[15] L. G. Rowan, E. L. Hahn, W. B. Mims, *Phys. Rev.* **1965**, *137*, A61–A71.

[16] P. Höfer, A. Grupp, H. Nebenführ, M. Mehring, *Chem. Phys. Lett.* **1986**, *132*, 279–282.

[17] S. Stoll, A. Schweiger, *J. Magn. Reson.* **2006**, *178*, 42–55.

[18] M. J. Frisch, G. W. Trucks, H. B. Schlegel, G. E. Scuseria, M. A. Robb, J. R. Cheeseman, G. Scalmani, V. Barone, G. A. Petersson, H. Nakatsuji, et al., **2016**.

[19] A. D. Becke, *Phys. Rev. A* **1988**, *38*, 3098–3100.

[20] J. P. Perdew, *Phys. Rev. B* **1986**, *33*, 8822–8824.

[21] S. Grimme, S. Ehrlich, L. Goerigk, *J. Comput. Chem.* **2011**, *32*, 1456–1465.

[22] S. Grimme, *J. Comput. Chem.* **2006**, *27*, 1787–1799.

[23] J. Da Chai, M. Head-Gordon, *Phys. Chem. Chem. Phys.* **2008**, *10*, 6615–6620.

[24] F. Weigend, C. Hättig, H. Patzelt, R. Ahlrichs, S. Spencer, A. Willets, *Phys. Chem. Chem. Phys.* **2006**, *8*, 1057.

[25] F. Weigend, R. Ahlrichs, K. A. Peterson, T. H. Dunning, R. M. Pitzer, A. Bergner, *Phys. Chem. Chem. Phys.* **2005**, *7*, 3297.

[26] A. E. Reed, J. E. Carpenter, F. Weinhold, *NBO, Version 3.0*, **n.d.**

[27] T. Lu, F. Chen, *J. Comput. Chem.* **2012**, *33*, 580–592.

[28] F. Neese, *WIREs Comput. Mol. Sci.* **2012**, *2*, 73–78.

[29] E. Van Lenthe, J. G. Snijders, E. J. Baerends, *J. Chem. Phys.* **1996**, *105*, 6505–6516.

[30] A. V. Marenich, C. J. Cramer, D. G. Truhlar, *J. Phys. Chem. B* **2009**, *113*, 6378–6396.

[31] M. Yaacobi, A. Ben-Naim, *J. Phys. Chem.* **1974**, *78*, 175–178.

[32] A. Lannung, J. C. Gjaldbæk, S. Rundqvist, E. Varde, G. Westin, *Acta Chem. Scand.* **1960**, *14*, 1124–1128.

[33] T. Schnabel, J. Vrabec, H. Hasse, *Fluid Phase Equilib.* **2005**, *233*, 134–143.
